# Photoredox-active Cr(0) luminophores featuring photophysical properties competitive with Ru(II) and Os(II) complexes

**DOI:** 10.1038/s41557-023-01297-9

**Published:** 2023-08-14

**Authors:** Narayan Sinha, Christina Wegeberg, Daniel Häussinger, Alessandro Prescimone, Oliver S. Wenger

**Affiliations:** Department of Chemistry, University of Basel, Basel, Switzerland

**Keywords:** Inorganic chemistry, Photocatalysis, Optical spectroscopy, Electron transfer, Coordination chemistry

## Abstract

Coordination complexes of precious metals with the d^6^ valence electron configuration such as Ru(II), Os(II) and Ir(III) are used for lighting applications, solar energy conversion and photocatalysis. Until now, d^6^ complexes made from abundant first-row transition metals with competitive photophysical and photochemical properties have been elusive. While previous research efforts focused mostly on Fe(II), we disclose that isoelectronic Cr(0) gives access to higher photoluminescence quantum yields and excited-state lifetimes when compared with any other first-row d^6^ metal complex reported so far. The luminescence behaviour of the metal-to-ligand charge transfer excited states of these Cr(0) complexes is competitive with Os(II) polypyridines. With these Cr(0) complexes, the metal-to-ligand charge transfer states of first-row d^6^ metal complexes become exploitable in photoredox catalysis, and benchmark chemical reductions proceed efficiently under low-energy red illumination. Here we demonstrate that appropriate molecular design strategies open up new perspectives for photophysics and photochemistry with abundant first-row d^6^ metals.

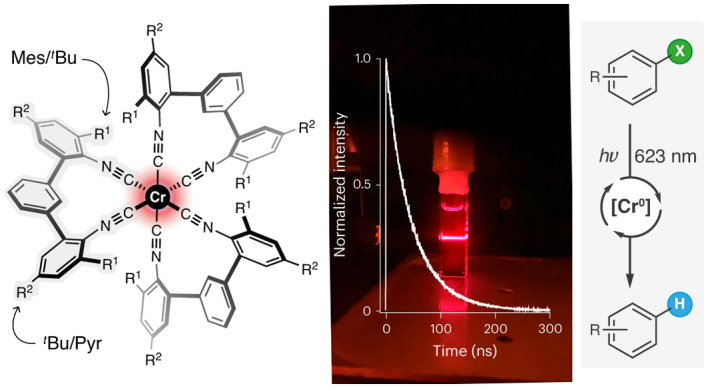

## Abstract (web summary)

Although noble metal coordination complexes typically show promising photophysical properties that enable applications in lighting, photocatalysis and solar energy conversion, first-row transition metal complexes rarely display properties as attractive. Now, two Cr(0) complexes are shown to afford excited-state lifetimes of ~50 ns and photophysical properties analogous to noble metal complexes, enabling efficient photoredox catalysis.

## Main

**Fig. 1:**
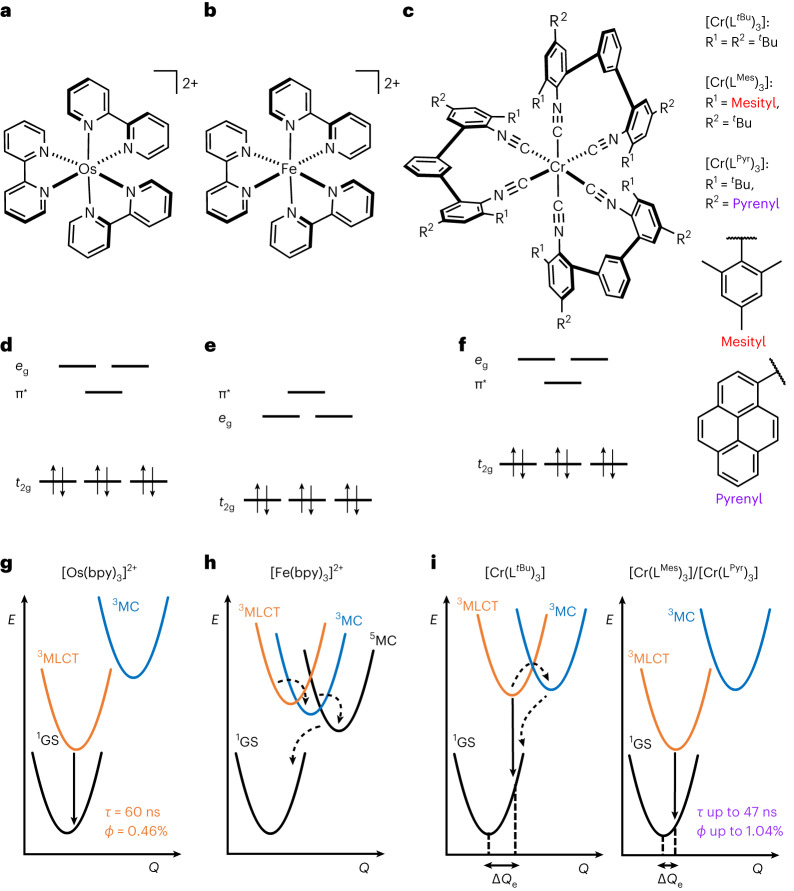
d^6^ metal complexes, their valence electron configurations and key electronic states. **a**–**c**, Molecular structures of [Os(bpy)_3_]^2+^ (**a**), [Fe(bpy)_3_]^2+^ (bpy, 2,2′-bipyridine) (**b**) and the new [Cr(L^Mes^)_3_] and [Cr(L^Pyr^)_3_] complexes (**c**). **d**–**f**, The low-spin d^6^ electron configurations in O_h_ point symmetry include a π* ligand orbital, which is the lowest unoccupied molecular orbital (LUMO) in [Os(bpy)_3_]^2+^ (**d**) but not in [Fe(bpy)_3_]^2+^ (**e**). For [Cr(L^Mes^)_3_] and [Cr(L^Pyr^)_3_], the situation is analogous to [Os(bpy)_3_]^2+^ (**f**). **g**–**i**, Potential-well energy diagrams of [Os(bpy)_3_]^2+^ (**g**), [Fe(bpy)_3_]^2+^ (**h**), and the previously explored [Cr(L^*t*Bu^)_3_] reference complex^15^, as well as the two new Cr(0) complexes (**i**). In [Cr(L^Mes^)_3_] and [Cr(L^Pyr^)_3_], the MLCT excited-state distortion (ΔQ_e_) is smaller than in [Cr(L^*t*Bu^)_3_], owing to cooperative rigidity and a π-electron density delocalization effect.

Upon photo-irradiation of a suitable metal complex, the promotion of an electron from the metal to a coordinated ligand can generate a metal-to-ligand charge transfer (MLCT) excited state with diverse applications in photophysics and photochemistry^1^. In many noble metal complexes, MLCT excited states luminesce and have lifetimes of several tens of nanoseconds or longer, which forms the basis for their use in lighting applications and photocatalysis^2–4^. Octahedral Ru(II), Os(II) and Ir(III) complexes with π-conjugated ligands are prototypical examples with a low-spin d^6^ configuration^5,6^ (Fig. 1a), in which three degenerate d-orbitals are all occupied with one electron pair, and two degenerate vacant d-orbitals are energetically above the lowest empty ligand π* orbital (Fig. 1d). In complexes with such an electronic structure, emissive and redox-active MLCT states can then emerge.

First-row transition metals experience weaker ligand fields than second- and third-row transition metals^7^, and the lowest unoccupied orbitals of 3d^6^ complexes become metal-based (Fig. 1e), which typically causes ultrafast MLCT deactivation by metal-centred (MC) states^8,9^. MLCT lifetimes in Fe(II) complexes only recently reached the pico- and nanosecond timescale^10–13^, and currently only a handful of 3d^6^ metal complexes show MLCT photoluminescence in solution at 20–25 °C^14–19^, where the highest reported quantum yield is 0.09% (ref. ^16^). This situation is very different from the d^10^ electron configuration of semiprecious Cu(I)^20^, for which luminescent charge transfer excited states are more readily obtainable^21,22^, because there are no low-lying MC states when all d-orbitals are filled^23^. Owing to their privileged 3d^10^ electron configuration, Cu(I) complexes and their photophysical properties are therefore not directly comparable to 3d^6^ metal complexes. Emissive complexes of abundant metals with other types of excited states have been reported (Zr(IV) (d^0^) (ref. ^24^), Cr(III) (d^3^) (ref. ^25^) and Fe(III) (d^5^) (ref. ^26^)), but 3d^6^ analogues of the abovementioned Ru(II), Os(II) and Ir(III) complexes with competitive photophysical and photochemical properties have been unknown^27,28^.

In this Article, we report two Cr(0) complexes with MLCT excited-state lifetimes close to 50 ns and photoluminescence quantum yields competitive with benchmark Os(II) polypyridines. These photophysical properties permit MLCT-based photoredox catalysis analogous to that known from many precious d^6^ metal complexes.

## Results and discussion

### Molecular design, synthesis and characterization

Non-radiative MLCT deactivation in d^6^ complexes decelerates in strong ligand fields, because the MC states are shifted to higher energies^29^. Isocyanide ligands create strong ligand fields^30^, which provide W(0) complexes with promising photophysics and photochemistry^31,32^. We developed isocyanide chelate ligands that provided brightly emissive Mo(0) complexes^33^, but with the first-row transition metals Cr(0) and Mn(I), the MLCT luminescence and the excited-state lifetimes remained inferior to noble metal compounds^16,18^. Two complementary molecular design principles now yield the first 3d^6^ complexes (Fig. 1c) with photophysical and photochemical behaviour competitive with precious metal-based analogues. The electronic structures of these Cr(0) complexes (Fig. 1f) resemble those of well-known noble metal analogues (Fig. 1g,i), more than those of Fe(II) polypyridines (Fig. 1h).

**Fig. 2:**
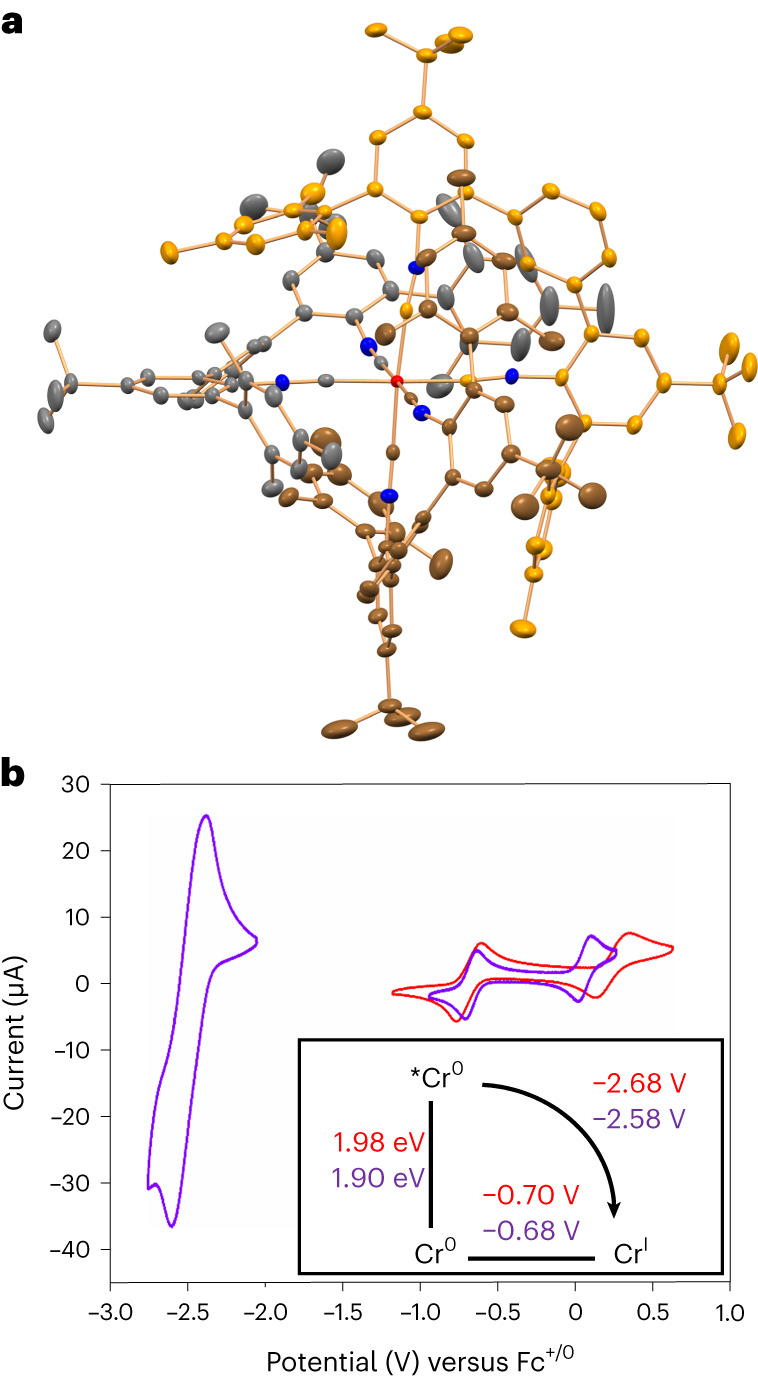
X-ray crystal structure and cyclic voltammetry. **a**, X-ray crystal structure of [Cr(L^Mes^)_3_] (50% probability ellipsoids). The carbon atoms of the three chelating bidentate isocyanide ligands are shown in a different colour coding. Hydrogen atoms and solvent molecules are omitted. **b**, Cyclic voltammograms of [Cr(L^Mes^)_3_] (red) and [Cr(L^Pyr^)_3_] (purple) in de-aerated dry THF (1 mM) containing 0.1 M ^n^Bu_4_NPF_6_. Inset: Latimer diagram to estimate the potentials for oxidation of Cr(0) in the photoactive ^3^MLCT excited state (*E_ox_(Cr(I/0))) of [Cr(L^Mes^)_3_] and [Cr(L^Pyr^)_3_]. Source data

The new complexes [Cr(L^Mes^)_3_] and [Cr(L^Pyr^)_3_] were obtained in 78% and 47% yields, respectively, by reacting the previously unknown ligands L^Mes^ and L^Pyr^ with CrCl_3_(THF)_3_ in the presence of Na/Hg in dry and de-aerated tetrahydrofuran (THF) at room temperature. Nuclear magnetic resonance (NMR) spectroscopy, mass spectrometry, combustion analysis and infra-red spectroscopy establish the identity and purity of the complexes. The key characteristics of isocyanide complexes including the ^13^C NMR resonances of the coordinating carbon atoms, as well as C≡N stretches in infra-red spectroscopy, are readily detectable (Supplementary Figs. 15, 31, 72 and 81). In the X-ray crystal structure of [Cr(L^Mes^)_3_] (Fig. 2a), the six mesityl substituents *ortho* to the isocyanide groups wedge in between the *m*-terphenyl ligand backbones, and thus impart cooperative rigidity to the overall complex while simultaneously protecting the Cr(0) atom. The absence of EXSY peaks between the two *ortho* methyl groups of the mesityl in the NOESY and ROESY NMR spectra (Supplementary Figs. 18 and 19) as well as the distinctly different NOE patterns (Supplementary Fig. 18) for the methyl pointing towards the chromium, compared with the outward-oriented methyl group, clearly demonstrate that this rigidity is also maintained in solution. Solid [Cr(L^Mes^)_3_] and [Cr(L^Pyr^)_3_] can be stored under air for several weeks without undergoing noticeable degradation, and an initially de-aerated solution of [Cr(L^Mes^)_3_] showed only 3% of decomposition over 15 days of exposure to air (Supplementary Fig. 33). The single crystal used for X-ray diffraction was grown in an NMR tube that was open to air. Both complexes remained intact for several days in de-aerated toluene-d_8_ at 115 °C (Supplementary Figs. 32 and 88). A single set of sharp ^1^H NMR resonances indicates that the three ligands in [Cr(L^Mes^)_3_] are symmetry related at 298 K, whereas for [Cr(L^Pyr^)_3_] analogous behaviour is only observed at 378 K, due to hindered rotation of the *tert*-butyl groups at lower temperatures (Supplementary Fig. 75). Thus, while the pyrene substituents on the backbone of L^Pyr^ rotate freely above 318 K as shown by variable temperature NMR (Supplementary Fig. 76) and NOE contacts between the terphenyl protons to both sides of the pyrene substituent at 378 K (Supplementary Fig. 84), the coalescence pattern of the *tert*-butyl resonances suggests that the structural rigidity of [Cr(L^Pyr^)_3_] and the steric protection of the metal centre are mainly due to inter-ligand contacts caused by the *tert*-butyl groups.

### Electrochemistry and photophysics

Oxidation of Cr(0) to Cr(I) occurs reversibly near −0.7 V versus Fc^+^/Fc in both complexes (Fig. 2b), and, along with oxidation of Cr(I) to Cr(II) at higher potentials, is typical for hexakis(arylisocyanide) complexes of Cr(0) (ref. ^34^). A reversible wave at −2.50 V versus Fc^+^/Fc observed for [Cr(L^Pyr^)_3_] is attributable to reduction of the pyrene substituents, whereas reduction of the *m*-terphenyl backbones of the diisocyanide ligands is outside the electrochemical window of suitable electrolytes^15,17^.

**Fig. 3:**
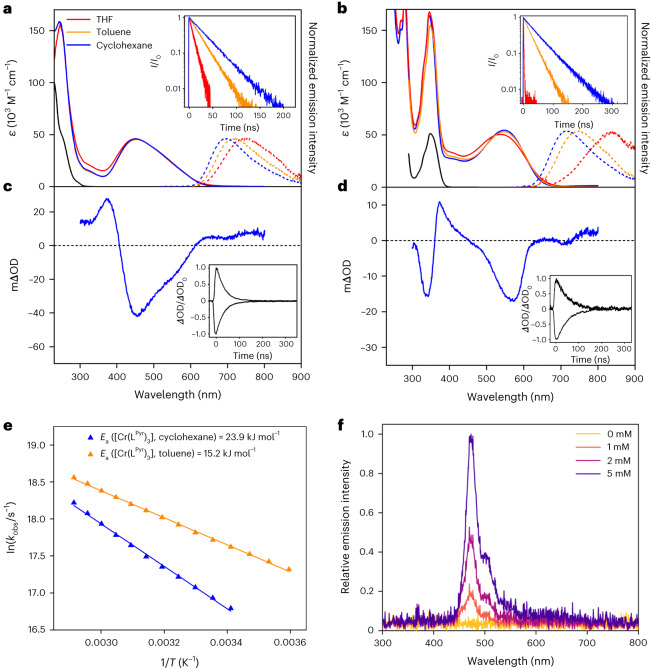
Photophysical characterization of Cr(0) tris(diisocyanide) complexes. **a**, UV–Vis absorption spectra (solid traces) of uncoordinated L^Mes^ ligand in THF (black) and of [Cr(L^Mes^)_3_] in THF, toluene and cyclohexane. Photoluminescence spectra (*λ*_exc_ = 500 nm, dotted traces) of [Cr(L^Mes^)_3_] in the same solvents. Inset: luminescence decay kinetics (*λ*_exc_ = 473 nm) of [Cr(L^Mes^)_3_]. **b**, UV–Vis absorption spectra (solid traces) of uncoordinated L^Pyr^ ligand in toluene (black) and of [Cr(L^Pyr^)_3_] in THF, toluene and cyclohexane. Photoluminescence spectra (*λ*_exc_ = 550 nm, dotted traces) of [Cr(L^Pyr^)_3_] in the same solvents. Inset: luminescence decay kinetics (*λ*_exc_ = 473 nm) of [Cr(L^Pyr^)_3_]. **c**, Transient absorption spectrum of [Cr(L^Mes^)_3_] in cyclohexane, time integrated over 200 ns after excitation at 500 nm. Inset: kinetics of excited-state absorption (ESA) decay at 375 nm and ground-state bleach (GSB) recovery at 460 nm of [Cr(L^Mes^)_3_] in cyclohexane. **d**, Transient absorption spectrum of [Cr(L^Pyr^)_3_] in cyclohexane, time integrated over 50 ns after excitation at 550 nm. Inset: ESA (374 nm) and GSB (570 nm) kinetics of [Cr(L^Pyr^)_3_] in cyclohexane. **e**, Arrhenius plots of experimental MLCT excited-state decay rate constants (*k*_obs_) for [Cr(L^Pyr^)_3_] in cyclohexane and toluene providing estimates of the activation energy (*E*_a_) for relaxation of the ^3^MLCT state via non-radiative decay into MC states. **f**, Delayed fluorescence of ^1*^perylene detected after excitation of [Cr(L^Pyr^)_3_] (10 μM) at 635 nm in de-aerated toluene at 20 °C. *I*/*I*_0_ is the luminescence intensity as a function of time versus the initial luminescence intensity at time = 0. mΔOD represents a change in optical density. *T* is the temperature. Source data

The free L^Mes^ and L^Pyr^ ligands absorb only ultraviolet light, but [Cr(L^Mes^)_3_] and [Cr(L^Pyr^)_3_] feature MLCT bands covering large parts of the visible absorption spectrum (Fig. 3a,b). The increased π-conjugation network of the pyrene-decorated ligand causes a 100 nm red shift of the MLCT absorption band maximum of [Cr(L^Pyr^)_3_] compared with [Cr(L^Mes^)_3_]. Upon photo-excitation, both complexes show broad and unstructured luminescence. Between cyclohexane and THF, the luminescence band maxima shift from 695 nm to 745 nm in [Cr(L^Mes^)_3_] and from 713 nm to 840 nm in [Cr(L^Pyr^)_3_], because the emissive MLCT state is energetically more stabilized in high polarity solvents^35^. Thus, the luminescence of the two Cr(0) complexes occurs in the same spectral range as the MLCT emission of [Os(bpy)_3_]^2+^ (Table 1). Differences in solubility between the charge-neutral Cr(0) compounds and the dicationic Os(II) complex preclude direct comparison in the same solvent, yet the energies of the emissive MLCT excited states of [Cr(L^Mes^)_3_] and [Cr(L^Pyr^)_3_] in cyclohexane and of [Os(bpy)_3_]^2+^ in acetonitrile are evidently similar. The comparison of photophysical properties between these three compounds is therefore more meaningful than comparison with [Ru(bpy)_3_]^2+^.

**Table 1:** Photophysical parameters of the new Cr(0) complexes and a few benchmark d^6^ complexes

Complex	Solvent^h^	*λ*_abs, max_ (MLCT) (nm)	*λ*_em, max_ (MLCT) (nm)	^*^E_ox_ (V versus Fc^+/0^)	*τ* (ns)	*ϕ* (%)
[Cr(L^Mes^)_3_]^a^	Cyclohexane	451	695	−2.7	31	0.36 ± 0.017
[Cr(L^Pyr^)_3_]^a^	Cyclohexane	546	713	−2.6	47	1.04 ± 0.05
[Cr(L^*t*Bu^)_3_]^b^	THF	480	630	−2.4	2.2	0.001
[Mn(L^tri^)_2_]^c^	Acetonitrile	395	525	−2.3	1.7	0.03
[Fe(pqa)_2_]^d^	Toluene	730	Non-emissive	–	2.7^i^	–
[Fe(bpy)_3_]^2+e^	Acetonitrile	521	Non-emissive	–	5 × 10^−5^	–
[Os(bpy)_3_]^2+f^	Acetonitrile	640	723	−1.4	60	0.46
[Ru(bpy)_3_]^2+g^	Acetonitrile	452	620	−1.1	855	6.20

^a^This work. ^b^From ref. ^15^. ^c^From ref. ^18^. ^d^From ref. ^11^ (pqa, (phenanthridin-4-yl)(quinoline-8-yl)amido). ^e^From ref. ^39^. ^f^From ref. ^35^. ^g^From ref. ^53^. ^h^Dry and de-aerated at 20 °C. ^i^The lowest excited state is not a classical MLCT state in this case.

Transient absorption and time-resolved luminescence experiments yield single-exponential MLCT decays for both Cr(0) complexes in de-aerated THF, toluene and cyclohexane at 20 °C (Fig. 3a–d and Supplementary Tables 6 and 10). The observable trend in decay kinetics follows the energy gap law^35^, leading to the longest MLCT lifetimes (*τ*) in the most apolar solvent, 31 ns for [Cr(L^Mes^)_3_] and 47 ns for [Cr(L^Pyr^)_3_] in cyclohexane at 20 °C. The photoluminescence quantum yields (*ϕ*) under these conditions are 0.36 ± 0.02% for [Cr(L^Mes^)_3_] and 1.04 ± 0.05% for [Cr(L^Pyr^)_3_] (Table 1). For [Os(bpy)_3_]^2+^ in de-aerated acetonitrile, *τ* is 60 ns and *ϕ* is 0.46%^35^; hence, the Cr(0) complexes exhibit competitive photophysical properties. The even longer MLCT lifetime and greater luminescence quantum yield of [Ru(bpy)_3_]^2+^ are largely due to its 0.3 eV higher MLCT energy, which further limits non-radiative relaxation following the energy gap law^35^.

The MLCT lifetimes and luminescence quantum yields of [Cr(L^Mes^)_3_] and [Cr(L^Pyr^)_3_] exceed those of previously reported 3d^6^ complexes by at least an order of magnitude^8–16,19,27^. Aside from the rigid interlocked molecular structures discussed above, the extended π-conjugation network of the new diisocyanide ligands contributes to this behaviour. In the ultraviolet (UV)–visible (Vis) transient absorption spectrum of [Cr(L^Pyr^)_3_] (Fig. 3d), the negative signal around 340 nm coincides with the lowest pyrene-localized ^1^π–π* transition in the ground state, indicating that the photoactive MLCT state has admixed pyrene character. Thus, the excited electron of the emissive MLCT state appears to be strongly delocalized, in line with the strong emission solvatochromism. Such delocalization causes weaker distortion of the MLCT excited state relative to the ground state (Δ*Q*_e_ in Fig. 1i)^36^, making non-radiative relaxation less dominant, somewhat reminiscent of the even much more weakly distorted spin-flip excited states of Cr(III) (d^3^) compounds^25,27,37^. Compared with these spin-flip MC states of d^3^ complexes, long-lived and strongly emissive MLCT excited states in first-row d^6^ complexes are far more difficult to obtain^38^.

In isoelectronic Fe(II) complexes, internal conversion from the lowest MLCT to MC states (Fig. 1h) commonly represents the dominant non-radiative relaxation pathway^7–13,27,29^, and in some cases, this is essentially barrierless^39^. Temperature-dependent studies suggest that in [Cr(L^Pyr^)_3_] the activation barrier for internal conversion to an MC state is 24 kJ mol^−1^ in cyclohexane and 15 kJ mol^−1^ in toluene (Fig. 3e). Consequently, the higher luminescence quantum yield in cyclohexane is probably the combined result of decelerated non-radiative MLCT relaxation directly to the ground state and slowed internal conversion to MC states; analogous results are obtained for [Cr(L^Mes^)_3_] (Supplementary Figs. 41 and 42).

### Triplet energy transfer, upconversion, photoredox catalysis

**Fig. 4:**
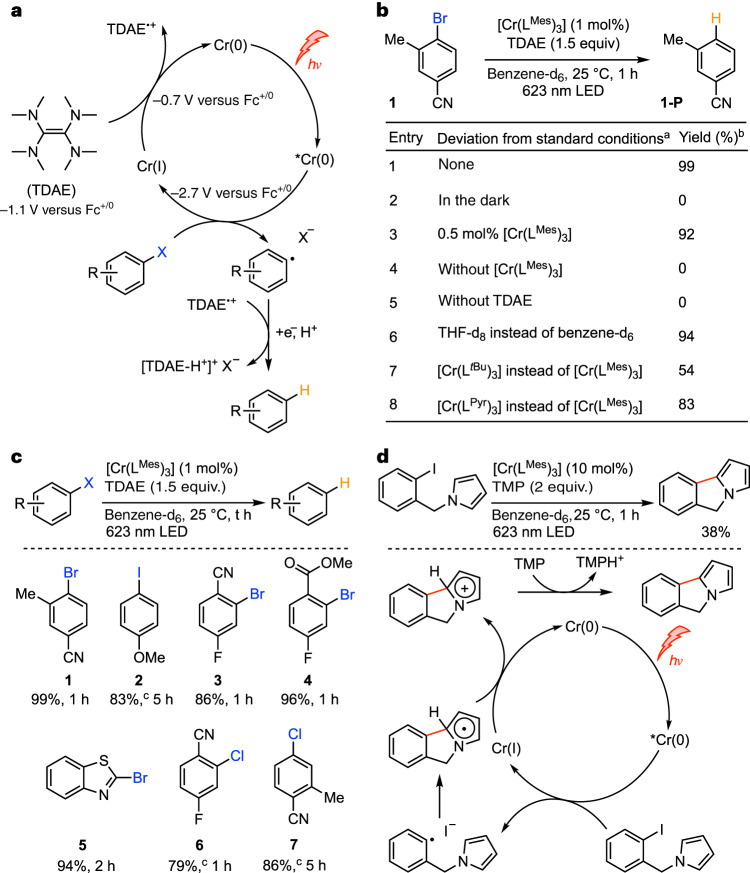
Photoredox catalysis using [Cr(L^Mes^)_3_] as a red-light absorbing sensitizer for benchmark reductive dehalogenation reactions. **a**, Proposed catalytic cycle in which the reductive dehalogenation reaction occurs directly from the ^3^MLCT excited state, and the Cr(0) resting state is subsequently regenerated by TDAE. **b**, Control experiments. ^a^Reaction conditions: aryl halide (0.06 mmol), [Cr(L^Mes^)_3_] (1 mol%), TDAE (1.5 equiv), benzene-d_6_ (0.6 mL), 623 nm LED (3.8 W) and room temperature. ^b^NMR yields obtained by using 1,4-dioxane as an internal standard. **c**, Substrate scope using the conditions listed in **b**. ^c^3 mol% [Cr(L^Mes^)_3_]. **d**, Overall redox-neutral, intramolecular base-promoted homolytic aromatic substitution (BHAS) reaction between iodobenzene and N-alkylated pyrrole using [Cr(L^Mes^)_3_] as a photocatalyst, along with a plausible mechanism for the formation of the C–C coupling product^33,44^. NMR yield obtained by using 1,4-dioxane as an internal standard.

Based on the absorption and emission data (Fig. 3a,b), the MLCT energies of [Cr(L^Mes^)_3_] and [Cr(L^Pyr^)_3_] are 1.98 eV and 1.90 eV, respectively. Triplet–triplet energy transfer to perylene (Supplementary Figs. 98 and 99) occurs with essentially diffusion-limited kinetics (9.2 × 10^9^ M^−1^ s^−1^), indicating that both Cr(0) compounds are amenable to triplet–triplet energy transfer catalysis^40^. Delayed perylene fluorescence at 480 nm is detectable upon selective excitation of [Cr(L^Pyr^)_3_] at 635 nm (Fig. 3f), illustrating that the Cr(0) complexes can sensitize triplet–triplet annihilation upconversion^41,42^. Given the abovementioned MLCT energies and the ease of Cr(0) to Cr(I) oxidation, [Cr(L^Mes^)_3_] and [Cr(L^Pyr^)_3_] become strong excited-state reductants with potentials near −2.7 V versus Fc^+^/Fc (Fig. 2b inset)^43^. W(0) arylisocyanides have a similar reducing power^32,44^, but common precious metal-based d^6^ complexes are far weaker photo-reductants^1,4,45^ (Table 1). Different aryl halides (Fig. 4) reductively quench the MLCT state of [Cr(L^Mes^)_3_]. For example, 4-iodoanisole reacts with a rate constant of 1.9 × 10^7^ M^−1^ s^−1^ in de-aerated toluene at 20 °C, in addition to showing some static quenching (Supplementary Figs. 104 and 105). Catalytic photoreductions of aryl halides become possible in the presence of tetrakis(dimethylamino)ethylene (TDAE), a commercial reductant capable of regenerating Cr(I) to Cr(0) after initial photo-induced electron transfer to the individual substrates (Fig. 4a). Similar hydrodehalogenation reactions with organic or precious metal-based photocatalysts typically require up to two blue or green photons per turnover^45–47^, whereas [Cr(L^Mes^)_3_] drives the reactions with red light, keeping photodegradation at an acceptable level (Supplementary Fig. 100). Photocatalysis with isoelectronic Fe(II) complexes usually occurs from MC states^48,49^, and there is only one single report involving a dark (non-luminescent) MLCT state, which, however, relies on consecutive ligand-to-metal charge transfer (LMCT) and MLCT excitation of an Fe(III)/Fe(II) system^50^. Aryl iodides, bromides and even an activated chloride with reduction potentials between −2.4 V and −2.7 V versus Fc^+^/Fc are reductively dehalogenated by MLCT-excited [Cr(L^Mes^)_3_] (Fig. 4c), providing the proof of concept for demanding photoreductions under red illumination that are not accomplishable in the same fashion with typical noble metal-based d^6^ complexes (Fig. 4b)^4,45,46^.

TDAE is a commercial reductant, but it is more expensive than other commonly used tertiary amine donors; hence, it seemed interesting to explore overall redox-neutral base-promoted homolytic aromatic substitution (BHAS) reactions, for which no electron donor at all needs to be added^33,44^. We chose 1-(2-iodobenzyl)-pyrrole as a substrate enabling an intramolecular variant of the BHAS reaction. Using [Cr(L^Mes^)_3_] as a photosensitizer, TMP (2,2,6,6-tetramethylpiperidine) as a base, and red light (Fig. 4d), the anticipated C–C coupled product formed in 38% yield at a catalyst loading of 10 mol%. The lower yield of the BHAS reaction and the need for higher catalyst loadings with respect to the hydrodehalogenations in Fig. 4c could have several reasons, including the following. First, the driving force for the reductive dehalogenation step in this specific substrate is only roughly 0.04 V. Second, the driving force for the regeneration of Cr(I) to Cr(0) in the catalytic cycle by the tricylic radical (Fig. 4d) is not known, but is probably in competition with nucleophilic attack of iodide anions at Cr(I), thereby leading to degradation of the sensitizer^33^. Nonetheless, the BHAS reaction in Fig. 4d provides an important proof of concept for overall redox-neutral reactions involving a thermodynamically demanding reduction step. [Ru(bpy)_3_]^2+^, [Ir(ppy)_3_] and the vast majority of their precious metal-based congeners are unable to catalyse comparable BHAS reactions^43^, because they lack sufficient reducing power in their MLCT excited states. After thousands of publications exploiting the MLCT excited states of precious 4d^6^ and 5d^6^ metal complexes for photoredox catalysis, the hydrodehalogenation and BHAS reactions demonstrated herein represent the first examples in which a luminescent MLCT excited state of a 3d^6^ metal complex has been used for photoredox catalysis.

After decades of research targeting 3d^6^ complexes emitting from the same type of MLCT excited state with competitive photophysical properties as hundreds of precious 4d^6^ and 5d^6^ metal complexes, only one single Fe(II) complex has been reported to emit from a ^3^MLCT excited state. This Fe(II) complex has an MLCT lifetime of 1 ns and a luminescence quantum yield close to the detection limit^19^. Two Mn(I) complexes exhibited ^3^MLCT lifetimes around 1 ns and luminescence quantum yields below 0.1% (refs. ^18,38^), and two Cr(0) complexes had slightly longer ^3^MLCT lifetimes (2–6 ns) and equally modest luminescence quantum yields (0.001–0.09%)^15,16^. Evidently, these previously reported MLCT-based 3d^6^ luminophores possess very short MLCT lifetimes and poor luminescence quantum yields. With the Cr(0) complexes reported herein, the MLCT phosphorescence lifetimes and quantum yields of 3d^6^ complexes finally become competitive with 4d^6^ or 5d^6^ compounds based on precious metals, and photocatalysis based on luminescent MLCT excited states is now possible using first-row d^6^ metal complexes. These findings complement recent key advances with LMCT excited states in complexes based on other abundant transition metals, in particular 3d^5^ Fe(III) LMCT luminophores with fluorescence lifetimes up to 2.0 ns and quantum yields up to 2.0% (refs. ^26,51^), in which the direction of charge transfer is opposite. Access to both LMCT and MLCT excited states with mutually complementary charge transfer directionalities is important to target the complete spectrum of photophysical and photochemical applications of first-row transition metal complexes^52^.

## Online content

Any methods, additional references, Nature Portfolio reporting summaries, source data, extended data, supplementary information, acknowledgements, peer review information; details of author contributions and competing interests; and statements of data and code availability are available at 10.1038/s41557-023-01297-9.

### Supplementary information

Supplementary Information Supplementary methods, Figs. 1–118 and Tables 1–13.

Supplementary Data 1 CIF crystal structure CCDC-2195170.

### Source data

Source Data Fig. 2 Cyclic voltammograms.

Source Data Fig. 3 UV–Vis absorption spectra, luminescence spectra, luminescence decays, UV transient absorption spectra and Arrhenius plots.

## Data Availability

Crystallographic data are available for free of charge from the Cambridge Crystallographic Data Centre under reference number CCDC-2195170 ([Cr(L^Mes^)_3_]). The data from the main paper are available from Figshare (10.6084/m9.figshare.22794431); all other data are in Supplementary Information. Source data are provided with this paper.
