# Supplementary material for: Photoredox-active Cr(0) luminophores featuring photophysical properties competitive with Ru(II) and Os(II) complexes
Source: Nat Chem. 2023 Aug 14;15(12):1730–6. doi: 10.1038/s41557-023-01297-9 (PMC10695827; doi:10.1038/s41557-023-01297-9)
Supplement: Supplementary file 1 — Supplementary methods, Figs. 1–118 and Tables 1–13. [file 41557_2023_1297_MOESM1_ESM.pdf]

nature chemistry

Article https://doi.org/10.1038/s41557-023-01297-9

# Photoredox-active Cr(0) luminophores featuring photophysical properties competitive with Ru(II) and Os(II) complexes

In the format provided by the authors and unedited

**Table of Contents**:


| | | |
|---|---|---|
| 1. | Materials and methods | 2 |
| 2. | Synthesis, characterization and photophysical details of $[Cr(L^{Mes})_3]$ | 4 |
| 2.1. | Synthesis and characterization of chelating diisocyanide ligand $L^{Mes}$ | 4 |
| 2.2. | Synthesis and characterization of $[Cr(L^{Mes})_3]$ | 7 |
| 2.3. | X-ray crystallography | 7 |
| 2.4. | NMR spectra | 9 |
| 2.5. | HR-ESI mass spectra | 21 |
| 2.6. | FTIR spectra | 24 |
| 2.7. | Temperature dependent $^1$H NMR study of $[Cr(L^{Mes})_3]$ | 25 |
| 2.8. | Air stability study of $[Cr(L^{Mes})_3]$ in solution | 26 |
| 2.9. | Cyclic voltammetry | 27 |
| 2.10. | UV-Vis Absorption, Emission spectra | 28 |
| 2.11. | Excitation spectra | 29 |
| 2.12. | Transient absorption spectra and excited state decays (absorption and emission) | 30 |
| 2.13. | Luminescence quantum yield determination | 32 |
| 2.14. | $^3$MLCT luminescence decay at different temperatures | 34 |
| 2.15. | Spectro-electrochemical experiment | 35 |
| 2.16. | Image of emission from $[Cr(L^{Mes})_3]$ in solution | 36 |
| 3. | Synthesis, characterization and photophysical details of $[Cr(L^{Pyr})_3]$ | 37 |
| 3.1. | Synthesis and characterization of chelating diisocyanide ligand $L^{Pyr}$ | 37 |
| 3.2. | Synthesis and characterization of $[Cr(L^{Pyr})_3]$ | 40 |
| 3.3. | NMR spectra | 41 |
| 3.4. | HR-ESI mass spectra | 50 |
| 3.5. | FTIR spectra | 54 |
| 3.6. | Temperature dependent $^1$H NMR study of $[Cr(L^{Pyr})_3]$ | 55 |
| 3.7. | Cyclic voltammetry | 64 |
| 3.8. | UV-Vis Absorption, Emission spectra | 65 |
| 3.9. | Excitation spectra | 66 |
| 3.10. | Transient absorption spectra and excited state decays (absorption and emission) | 67 |
| 3.11. | Luminescence quantum yield determination | 68 |
| 3.12. | $^3$MLCT luminescence decay at different temperatures | 69 |
| 3.13. | Chemical oxidation of $[Cr(L^{Pyr})_3]$ in solution | 70 |
| 3.14. | Triplet energy transfer to perylene | 71 |
| 4. | Photostability of $Cr^0$ complexes | 72 |
| 4.1. | Photostability of $[Cr(L^{Mes})_3]$ and $[Cr(L^{Pyr})_3]$ with respect to $[Ru(bpy)_3]^{2+}$ | 72 |
| 4.2. | Photostability study of $[Cr(L^{Mes})_3]$ under photoredox catalysis condition | 73 |
| 5. | Additional spectroscopic data | 74 |
| 5.1. | Steady-state emission spectra of $[Cr(L^{tBu})_3]$, $[Cr(L^{Mes})_3]$, $[Cr(L^{Pyr})_3]$ | 74 |
| 6. | Red light driven photoredox catalysis using $[Cr(L^{Mes})_3]$ as a photosensitizer | 75 |
| 6.1. | UV-Vis spectra of a representative substrate, electron donor and photosensitizer, and emission profile of 623 nm LED | 75 |
| 6.2. | Image of the photoredox reaction set up | 75 |
| 6.3. | Stern-Volmer quenching study | 76 |
| 6.4. | Red light driven hydrodehalogenation reactions | 76 |
| 6.5. | NMR spectra of hydrodehalogenation reactions | 78 |
| 6.6. | Red-light induced BHAS reaction | 87 |
| 7. | References | 89 |

**1. Materials and methods**. All experiments were carried out under a nitrogen or an argon atmosphere using standard Schlenk or Glovebox techniques. Glassware was oven-dried at 130 °C. Solvents were distilled by standard procedures prior to use. NMR spectra were recorded at 298 K (or as mentioned separately at various other temperatures) on Bruker AVANCE III 600, Bruker AVANCE III 500, Bruker AVANCE III 400, and Bruker AVANCE III 250 spectrometers. Variable temperature NMR experiments were recorded at 600 MHz using an indirect BBI probe head. All probes were equipped with self-shielded z-gradients. The temperature was calibrated using a methanol sample and showed an accuracy of ± 0.2 K. Routine gradient selected 2D NMR experiments were used (COSY, NOESY, HSQC, HMBC). Chemical shifts ($\delta$) are denoted relative to the residual solvent peak ($CDCl_3$: $\delta_H$ = 7.26 ppm, $\delta_C$ = 77.16 ppm. $C_6D_6$: $\delta_H$ = 7.16 ppm, $\delta_C$ = 128.39 ppm. Toluene-$d_8$: $\delta_H$ = 2.09 ppm, $\delta_C$ = 20.4 ppm). For the variable temperature NMR measurements, the solvent peaks of toluene-$d_8$ were kept at the values given above. The coupling constants ($J$) are given in Hz and these are described by the following abbreviations: s (singlet), d (doublet), t (triplet) and m (multiplet). All coupling constants are given in Hertz and are only reported for $^1H$-$^1H$ couplings. Mass spectra were acquired on Bruker esquire 3000 plus and Bruker maxis 4G QTOF EDI spectrometers. Elemental analysis was carried out on a Vario Micro Cube instrument. Solid-state IR spectroscopy (FTIR) was performed on a Bruker Alpha Platinum-ATR instrument, and the following abbreviations are used to describe the intensity of the vibrational bands: s (strong), m (medium) and w (weak).

Unless otherwise noted all chemicals were purchased from commercial suppliers and used without further purification. The synthetic reactions were monitored by thin layer chromatography using TLC Silica gel 60 plates coated with fluorescence indicator F254 (Merck). The compounds were visualized by UV absorption (254/366 nm). Purification by standard silica gel column chromatography was performed using SiliaFlash® P60, Silicycle. (Pore size: 40-63 μm).

$[Cr(L^{Mes})_3]$ and $[Cr(L^{Pyr})_3]$ can be handled as a solid under ambient atmosphere, but upon dissolution the chromium(0) complexes are unstable towards dissolved oxygen, hence solvents were deoxygenated using at least three cycles of the freeze-pump-thaw method prior to dissolution. For storage, the $Cr^0$ complexes were kept under an inert atmosphere (Ar or $N_2$) in the fridge. Selected samples were stored at room temperature under ambient atmosphere for up to one year and did not undergo noticeable degradation.

Cyclic voltammetry was performed in a MBraun Glovebox under an argon atmosphere with a VersaSTAT 4 potentiostat from Princeton Applied Research. A glassy carbon disk electrode served as a working electrode, the counter electrode and quasi-reference electrode were two silver wires. Internal potential calibration occurred by addition of small amounts of ferrocene. The solvent was dry deaerated THF containing 0.1 M ($^nBu_4N)PF_6$ (tetra-*n*-butylammonium hexafluorophosphate) as an electrolyte.

All photophysical measurements were performed in dry and deaerated solutions. Optical absorption spectroscopy was performed using a Cary 5000 instrument from Varian. Steady-state luminescence spectra were measured on a Fluorolog-322 from Horiba Jobin-Yvon, equipped with iHR320, a Xenon lamp 450 Watt Illuminator (FL-1039A/40A) and a water-cooled photomultiplier tube (PMT Hamamatsu R2658 or R928). The luminescence spectra were corrected for the spectral response of the system. An LP920-KS instrument from Edinburgh Instruments was employed for nanosecond transient absorption spectroscopy. The frequency-doubled output of a Quantel Brilliant b laser served as an excitation source. The laser pulse duration was ~10 ns and the pulse frequency was 10 Hz. The typical pulse energy used for the nanosecond transient absorption studies was 10 mJ. Detection of transient UV-Vis absorption spectra occurred on an iCCD camera from Andor. Picosecond pump-probe (transient) absorption studies with sub-nanosecond time resolution were performed using a

TRASS instrument from Hamamatsu and a mode-locked picosecond Nd:$YVO_4$/YAG laser (model PL2251B-20-SH/TH/FH with PRETRIG option) as an excitation source. The laser pulse duration was ~30 ps and the pulse frequency was 10 Hz. The laser pulse energy of 8 mJ at 355 nm powered an Ekspla PG402-264 OPA with an energy output of 1 mJ at 530 nm. Temperature dependent photoluminescence lifetime studies were performed on an FLS1000 spectrometer (time-correlated single photon counting (TCSPC) technique) from Edinburgh Instruments using a pulsed LED for excitation at 472.8 nm (Edinburgh Instruments, pulse width: 75.5 ps, linewidth <4.5 nm) or for excitation at 635.2 nm (Edinburgh Instruments, pulse width: 61.4 ps, linewidth <2.5 nm), where the cuvette holder was equipped with a temperature controller (TC 1, Quantum Northwest). Photostability studies were performed with a continuous-wave laser with excitation at 532 nm (Roithner LaserTechnik).

**Quantum yield determination**: The luminescence quantum yields of $[Cr(L^{Mes})_3]$ and $[Cr(L^{Pyr})_3]$ in deaerated solutions of cyclohexane, toluene and tetrahydrofuran ($\phi_{[Cr(0)\ complex],solvent}$) were determined relative to that of $[Ru(bpy)_3](PF_6)_2$ in aerated acetonitrile ($\phi_{[Ru(bpy)3]2+}$= 0.018)[1]. The x-axis of the emission spectra was converted from wavelength (nm) to wavenumber ($cm^{-1}$) following equation 1.

$$x\ \mathrm{nm} = \frac{10000000}{x}\ \mathrm{cm}^{-1} \qquad \text{(equation 1)}$$

When converting from wavelength (nm) scale to wavenumber ($cm^{-1}$) scale, the intensity of the emission spectra was adjusted following equation 2[2], to determine the corrected integrated emission intensities for the chromium(0) complexes and $[Ru(bpy)_3](PF_6)_2$.

$$I(\bar{\nu}) = \lambda^2 I(\lambda) \qquad \text{(equation 2)}$$

Equation 3 was used for the photoluminescence quantum yield determination, where I is the integrated emission intensity, A is the absorbance at 450 nm (the excitation wavelength) and η is the refractive index of the solvent ($\eta_{acetonitrile}$ = 1.344, $\eta_{cyclohexane}$= 1.436, $\eta_{toluene}$ = 1.497, $\eta_{tetrahydrofuran}$ = 1.407)[3]. Measurements involving at least six different concentrations were performed ($0.04 \leq A \leq 0.12$ at 450 nm). Neutral density filters with optical densities of 1.0 (OD 1.0, in the case of $[Cr(L^{Mes})_3]$) and 0.5 (OD 0.5, in the case of $[Cr(L^{Pyr})_3]$) were employed to attenuate the $[Ru(bpy)_3]^{2+}$ luminescence signal, in order to obtain comparable emission intensities under the same instrument settings for $[Ru(bpy)_3]^{2+}$, $[Cr(L^{Mes})_3]$ and $[Cr(L^{Pyr})_3]$. When determining the integrated emission intensity of $[Ru(bpy)_3]^{2+}$ from the emission spectra (i.e. “$I_{[Ru(bpy)3]2+}$“ in eq. 3), the use of the OD 1.0 and OD 0.5 filters was afterwards taken into account.

$$\phi_{\mathrm{Cr(0)complex,\ solvent}} = \frac{\frac{I_{\mathrm{Cr(0)complex}}}{A_{\mathrm{Cr(0)complex}}}}{\frac{I_{[\mathrm{Ru(bpy)_3}]^{2+}}}{A_{[\mathrm{Ru(bpy)_3}]^{2+}}}} \cdot \phi_{[\mathrm{Ru(bpy)_3}]^{2+}} \cdot \frac{\eta^2_{\mathrm{solvent}}}{\eta^2_{\mathrm{acetonitrile}}} \qquad \text{(equation 3)}$$

## 2. Synthesis, characterization and photophysical details of [Cr($L^{Mes}$)$_3$]

### 2.1. Synthesis and characterization of chelating diisocyanide ligand $L^{Mes}$

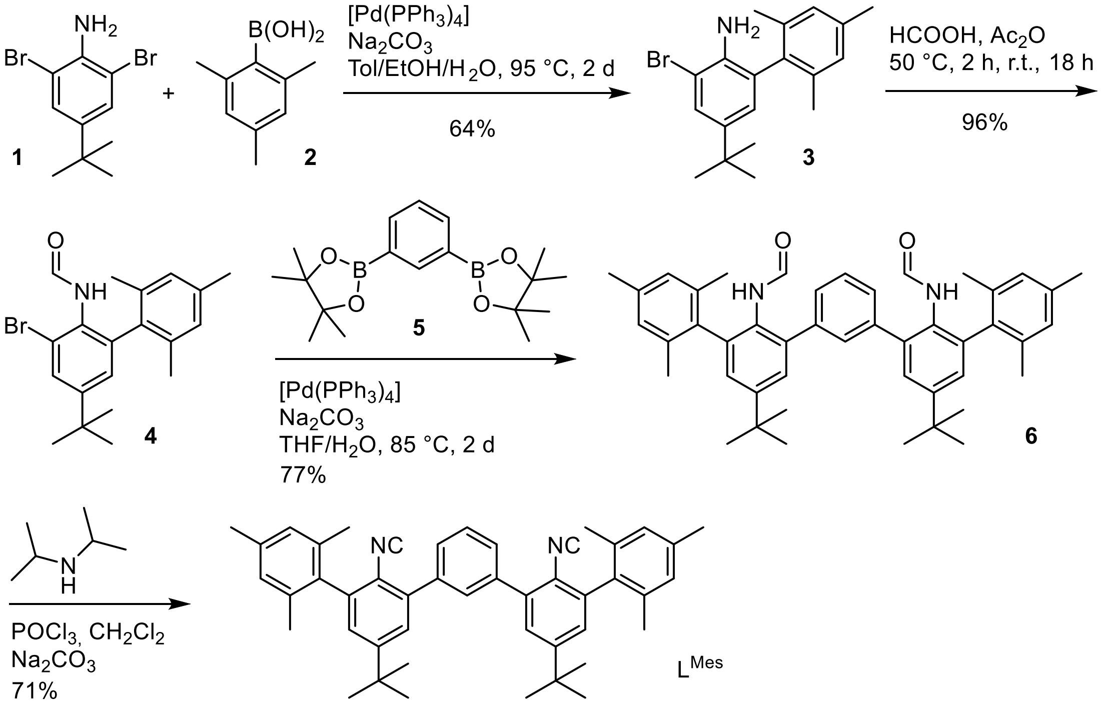


**Supplementary Fig. 1.** Synthesis of chelating bidentate arylisocyanide ligand, $L^{Mes}$.

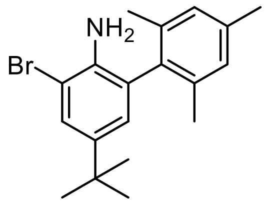

**Synthesis of compound 3**. A 250 mL Schlenk flask was charged with 2,6-dibromo-4-(*tert*-butyl)aniline (3.0 g, 9.771 mmol, 1.0 equiv.), 2,4,6-trimethylphenyl boronic acid (1.762 g, 10.748 mmol, 1.1 equiv.), $Na_2CO_3$ (4.142 g, 39.084 mmol, 4.0 equiv.) and [Pd($PPh_3$)$_4$] (0.564 g, 0.488 mmol, 0.05 equiv.). A mixture of toluene/ethanol/water (40 mL : 20 mL : 20 mL) was added and the resulting reaction mixture was degassed by bubbling $N_2$ for 15 min, and subsequently refluxed at 95 °C for 48 h. After cooling to ambient temperature, water (100 mL) was added. The organic phase was separated, and aqueous phase was extracted with dichloromethane (2 × 50 mL). The combined organic phases were dried over anhydrous $Na_2SO_4$, and evaporated under reduced pressure. The crude product was purified by silica gel column chromatography using petroleum ether/dichloromethane (7:3) as an eluent to obtain an off-white solid, compound **3** (2.160 g, 6.237 mmol, 64%). $^{1}H$ NMR (500 MHz, $CDCl_3$): $\delta$ 7.43 (d, $J$ = 2.2 Hz, 1H), 7.00 (br s, 2H), 6.92 (d, $J$ = 2.2 Hz, 1H), 3.72 (br s, 2H), 2.36 (s, 3H), 2.03 (s, 6H), 1.29 (s, 9H) ppm. $^{13}C\{^{1}H\}$ NMR (125 MHz, $CDCl_3$): $\delta$ 142.4, 139.0, 137.5, 137.1, 135.1, 128.7, 128.0, 126.9, 126.6, 109.2, 34.2, 31.6, 21.2, 20.2 ppm. $R_f$ = 0.66 (petroleum ether:dichloromethane 7:3). HRMS (ESI, positive ions): *m/z* 346.1169 (calculated for [**3**+H]$^+$ 346.1165). FTIR: ν 3454 (w), 3364 (m), 2955 (s), 2919 (m), 2865 (w), 1748 (w), 1608 (s), 1577 (s), 1553 (m), 1475 (s), 1438 (s), 1360 (s), 1304 (m), 1289 (m), 1247 (s), 1069 (s), 1034 (w), 1020 (w), 876 (s), 857 (s), 755 (s), 747 (m), 707 (m), 644 (w), 603 (w), 560 (w) cm$^{-1}$.

**Synthesis of compound 4**. In a 100 mL Schlenk flask, formic acid (10 mL) was slowly added

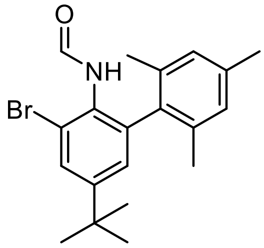

to acetic anhydride (20 mL) at 0 °C. The mixture was degassed by purging of $N_2$ and subsequently heated to 50 °C for 2 h. After cooling to ambient temperature, compound **3** (0.600 g, 1.733 mmol) was added under $N_2$ and the resulting reaction mixture was stirred for 18 h. The reaction mixture was added to cold water (200 mL), and the resulting mixture was extracted with dichloromethane (2 × 50 mL). The combined organic phases were washed with water (4 × 50 mL), dried over anhydrous $Na_2SO_4$, and evaporated under reduced pressure to yield a light-brown solid, compound **4** (0.620 g, 1.656 mmol, 96%). Two different (formamide) rotamers were observed in the $^1$H NMR spectrum, which is consistent with previously reported $^1$H NMR spectra of related compounds [4]. $^1$H NMR (500 MHz, $CDCl_3$): $\delta$ 8.05-7.93 (m, 1H), 7.66-7.62 (m, 1H), 7.14-7.13 (m, 1H), 6.96-6.90 (m, 3H), 2.32 (3H), 1.96 (s, 6H), 1.31 (s, 9H) ppm. HRMS (ESI, positive ions): *m/z* 374.1109 (calculated for [**4**+H]$^+$ 374.1114). Anal. Calcd. for **4** ($C_{20}H_{24}BrNO$) (%): C, 64.07; H, 6.33; N, 3.59 (observed); C, 64.17; H, 6.17; N, 3.74 (calculated). FTIR: ν 3216 (w), 3117 (w), 2963 (w), 2910 (w), 2862 (w), 1687 (s), 1658 (s), 1598 (w), 1498 (s), 1393 (m), 1363 (m), 1290 (m), 1239 (w), 1158 (w), 1104 (w), 1016 (w), 875 (m), 853 (s), 761 (s), 694 (w), 653 (w), 604 (m), 535 (w), 465 (w) cm$^{-1}$.

**Synthesis of compound 5**. The following procedure was adapted from a previously published

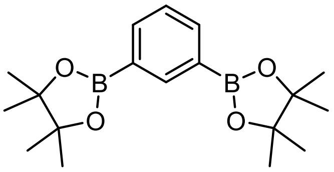

protocol[5]. In a Schlenk flask, 1,3-Dibromobenzene (2.03 g, 8.61 mmol, 1.0 equiv.), bis(pinacolato)diboron (6.56 g, 25.8 mmol, 3.0 equiv.), and KOAc (5.07 g, 51.6 mmol, 6.0 equiv.) were added to isopropanol (40 ml), and the suspension was purged with $N_2$ for 30 min. [$PdCl_2(PPh_3)_2$] (306 mg, 0.44 mmol, 0.05 equiv.) was added and the reaction mixture was purged with $N_2$ for another 10 min. The mixture was refluxed overnight under $N_2$. The reaction mixture was cooled to room temperature, and a saturated aqueous solution of $NH_4Cl$ (100 ml) was added. The aqueous phase was extracted with dichloromethane (3 × 50 ml), and the combined organic phases were dried over anhydrous $Na_2SO_4$, filtered and concentrated under reduced pressure. Purification by chromatography on a silica gel column with petroleum ether/ethyl acetate 5:1 (v/v) yielded a white solid (mixture of compound **5** and bis(pinacolato)diboron). The crude product was recrystallized from hot methanol/water (v/v 10:1, 55 mL) to give compound **5** as a white crystalline solid. Yield: 1.51 g, 4.58 mmol, 53 %. NMR data was in agreement with literature [5,6]. $^1$H NMR (400 MHz, $CDCl_3$) $\delta$ 8.28 (dd, *J* = 0.7, 1.4 Hz, 1H), 7.90 (dd, *J* = 7.4, 1.4 Hz, 2H), 7.37 (td, *J* = 7.4, 0.7 Hz, 1H), 1.34 (s, 24H) ppm.

**Synthesis of compound 6**. A 100 mL Schlenk flask was charged with compound **5** (0.250 g,

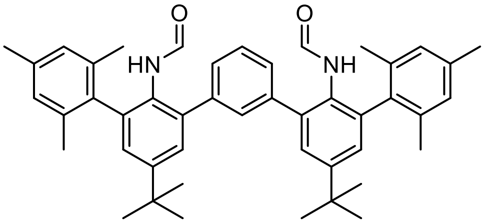

0.757 mmol, 1.0 equiv.), compound **4** (0.623 g, 1.665 mmol, 2.2 equiv.), $Na_2CO_3$ (0.481 g, 4.542 mmol, 6.0 equiv.) and [$Pd(PPh_3)_4$] (0.087 g, 0.076 mmol, 0.1 equiv.) THF (28 mL) and water (7 mL) were added, and the mixture was degassed by bubbling $N_2$ for 15 min, and heated under reflux at 85 °C for 48 h. After cooling to ambient temperature, water (100 mL) was added, and the product was extracted with dichloromethane (2 × 50 mL). The combined organic phases were dried over $Na_2SO_4$ and evaporated under reduced pressure to obtain the crude product, which was purified by silica gel column chromatography using petroleum ether/ethyl acetate (4:1) as an eluent to obtain the neat compound **6** (0.386 g, 0.581 mmol, 77%). Due to the presence of two (formamide) rotamers of compound **4**, the compound **6** formed as a mixture

of several isomers. $^{1}H$ NMR (250 MHz, $CDCl_3$): 7.69-7.30 (m, 9H), 7.21-7.16 (m, 2H), 6.93 (s, 4H), 6.84-6.62 (m, 1H), 2.30 (br s, 6H), 2.00 (br s, 12H), 1.36-1.33 (m, 18H) ppm. $R_f$ (petroleum ether/ethyl acetate (4:1) = 0.25. HRMS (ESI, positive ions): *m/z* 665.4098 (calculated for [**6**+H]$^+$ 665.4102); 687.3922 (calculated for [**6**+Na]$^+$ 687.3921). Anal. Calcd. for **6**·0.5($H_2O$) [($C_{46}H_{52}N_2O_2$)·0.5($H_2O$)] (%): C, 81.80; H, 8.10; N, 3.97 (observed); C, 81.98; H, 7.93; N, 4.16 (calculated). FTIR: ν 3262 (w), 2961 (m), 2915 (w), 2866 (w), 1681 (s), 1612 (w), 1476 (m), 1362 (m), 1276 (w), 1242 (m), 1030 (w), 883 (m), 849 (m), 801 (m), 710 (m), 655 (w), 592 (w), 560 (w), 541 (w) cm$^{-1}$.

**Synthesis of $L^{Mes}$**. In a Schlenk flask, compound **6** (0.386 g, 0.581 mmol, 1.0 equiv.) was dissolved in dry dichloromethane (20 mL), and diisopropylamine (0.412 g, 4.067 mmol, 7.0 equiv.) was added under $N_2$. After cooling to 0 °C, $POCl_3$ (0.498 g, 3.254 mmol, 5.6 equiv.) was added slowly, and the resulting reaction mixture was stirred at ambient temperature for 5 h. An aqueous solution of $Na_2CO_3$ (1.5 M, 20 mL) was added and the resulting biphasic mixture was stirred for 18 h. Water (20 mL) was added and the entire mixture was extracted with dichloromethane (2 × 50 mL). The combined organic phases were washed with saturated $NaHCO_3$ solution (3 × 50 mL), dried over anhydrous $Na_2SO_4$ and evaporated under reduced pressure. The crude product was purified by silica gel column chromatography using dichloromethane as an eluent to obtain the bidentate arylisocyanide ligand, $L^{Mes}$ (0.260 g, 0.413 mmol, 71%) as an off-white solid. $^{1}H$ NMR (500 MHz, $CDCl_3$): $\delta$ 7.79 (s, 1H), 7.66-7.59 (m, 3H), 7.53 (d, *J* = 2.2 Hz, 2H), 7.26 (d, *J* = 1.3 Hz, 2H), 7.01 (s, 4H), 2.36 (s, 6H), 2.07 (s, 12H), 1.36 (s, 18H) ppm. $^{13}C\{^{1}H\}$ NMR (125 MHz, $CDCl_3$): $\delta$ 167.9, 153.2, 139.5, 138.5, 138.3, 137.8, 135.9, 134.9, 130.1, 129.1, 128.8, 128.6, 127.2, 126.6, 121.5, 35.3, 31.3, 21.3, 20.4 ppm. $R_f$ (dichloromethane) = 0.9. HRMS (ESI, positive ions): *m/z* 651.3712 (calculated for [$L^{Mes}$+Na]$^+$ 651.3710). Anal. Calcd. for $L^{Mes}$ ($C_{46}H_{48}N_2$) (%): C, 87.89; H, 7.72; N, 4.50 (observed); C, 87.85%; H, 7.69; N, 4.45 (calculated). FTIR: 2961 (w), 2911 (w), 2868 (w), 2115 (s, $\nu_{C\equiv N}$), 1576 (w), 1457 (m), 1363 (m), 1243 (m), 1195 (w), 1027 (w), 887 (m), 849 (m), 800 (m), 762 (w), 710 (s), 659 (w), 626 (m), 560 (w), 543 (w), 441 (m), 419 (m) cm$^{-1}$.

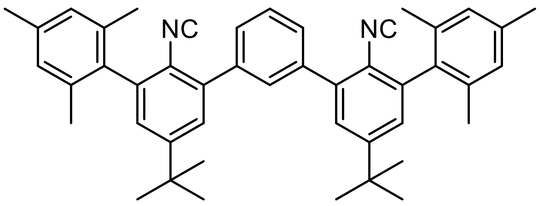

## 2.2. Synthesis of [Cr($L^{Mes}$)$_3$]

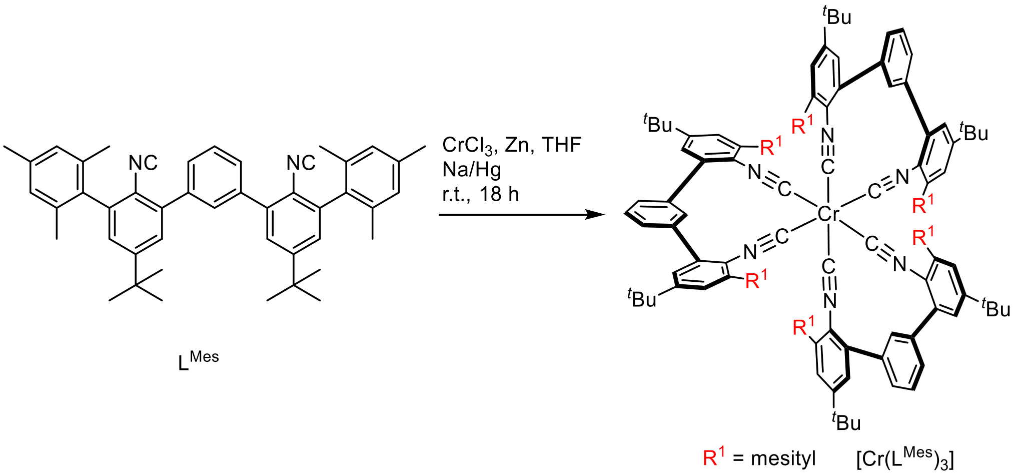


[Cr($L^{Mes}$)$_3$] was prepared following a previously published method for $Cr^0$ arylisocyanide complexes[7,8]. Anhydrous $CrCl_3$ (17.0 mg, 0.107 mmol) was stirred under argon in dry THF (5 mL) in the presence of Zn dust (3 mg) to form a green colored solution of [$CrCl_3$(THF)$_3$]. In a separate Schlenk flask, Na/Hg was prepared under argon by adding small pieces of Na (100.0 mg) in Hg (1.5 mL), and thereafter dry THF (2 mL) was added, and this mixture was stirred for 5 min. To this, a solution of $L^{Mes}$ (200.0 mg, 0.318 mmol) in dry THF (10 mL) was added. The resulting mixture was stirred for 10 min before the freshly prepared solution of [$CrCl_3$(THF)$_3$] was added dropwise, whereupon the reaction mixture immediately turned dark red. Stirring was continued for 18 h in the dark. Thereafter, the reaction mixture was filtered under argon through a small pad of celite. The dark red filtrate was evaporated to dryness to obtain [Cr($L^{Mes}$)$_3$] (160 mg, 0.083 mmol, 78%). $^1$H NMR (600 MHz, $CDCl_3$): $\delta$ 8.16 (s, 3H), 7.66-7.63 (m, 12H), 7.52 (t, $J$ = 7.7 Hz, 3H), 7.02 (s, 6H), 6.70 (s, 6H), 6.48 (s, 6H), 2.20 (s, 18H), 1.85 (s, 18H), 1.76 (s, 18H), 1.23 (s, 54H) ppm. $^{13}$C{$^1$H} NMR (125 MHz, $CDCl_3$): $\delta$ 196.0, 146.7, 140.3, 138.0, 137.1, 136.7, 136.6, 136.5, 136.3, 131.7, 129.9, 129.3, 129.2, 129.1, 128.8, 127.9, 125.6, 34.8, 31.7, 21.8, 21.7, 21.0 ppm. HRMS (ESI, positive ions): $m/z$ 969.0445 (calcd for [Cr($L^{Mes}$)$_3$]$^{2+}$ 969.0440). Anal. Calcd. for [Cr($L^{Mes}$)$_3$]·2(THF) [($C_{138}H_{144}N_6Cr$)·2($C_4H_8O$)] (%): C, 84.01; H, 8.12; N, 3.88 (observed); C, 84.19; H, 7.74; N, 4.04 (calculated). FTIR: ν 2952 (m), 2911 (m), 2853 (w), 1930 (s, $\nu_{C\equiv N}$), 1591 (w), 1426 (m), 1379 (m), 1361 (w), 1240 (w), 1220 (w), 1040 (w), 913 (w), 885 (m), 847 (m), 800 (m), 749 (m), 722 (m), 696 (s), 649 (w), 586 (s, $\nu_{Cr\text{-}C}$), 511 (m), 491 (w), 443 (m) cm$^{-1}$.

**2.3. X-ray crystallography.** A suitable red block-shaped single crystal of [Cr($L^{Mes}$)$_3$]·2$C_6H_6$ with dimensions 0.16×0.13×0.10 mm$^3$ was selected and mounted on a mylar loop in perfluoroether oil on a STOE STADIVARI diffractometer. The crystal was kept at a steady $T$ = 150 K during data collection. The structure was solved with the ShelXT 2018 solution program and by using Olex2 as the graphical interface[9,10]. The model was refined with ShelXL 2018 using full matrix least squares minimization on $\boldsymbol{F}^2$ [11]. All non-hydrogen atoms were refined anisotropically. Hydrogen atom positions were calculated geometrically and refined using the riding model. The asymmetric unit contains one formula unit of [Cr($L^{Mes}$)$_3$] and two molecules of benzene. Two *tert*-butyl groups in the complex are disordered and they are modelled over several positions. CCDC 2195170 ([Cr($L^{Mes}$)$_3$]·2$C_6H_6$) contains the supplementary crystallographic data for this paper. These data can be obtained free of charge from The Cambridge Crystallographic Data Centre.

**Supplementary Table 1**. Crystallographic parameters for the structure of $[Cr(L^{Mes})_3]\cdot 2C_6H_6$.

| **Compound** | $[Cr(L^{Mes})_3]\cdot 2C_6H_6$ |
|---|---|
| Formula | $C_{150}H_{156}CrN_6$ |
| $D_{calc.}$/ g cm$^{-3}$ | 1.140 |
| *m*/mm$^{-1}$ | 0.790 |
| Formula Weight | 2094.80 |
| Colour | red |
| Shape | block-shaped |
| Size/mm$^3$ | 0.16×0.13×0.10 |
| *T*/K | 150 |
| Crystal System | triclinic |
| Space Group | *P*-1 |
| *a*/Å | 14.9318(4) |
| *b*/Å | 15.8909(4) |
| *c*/Å | 26.7375(6) |
| $\alpha$/° | 92.110(2) |
| $\beta$/° | 93.775(2) |
| $\gamma$/° | 105.082(2) |
| V/Å$^3$ | 6102.9(3) |
| *Z* | 2 |
| *Z'* | 1 |
| Wavelength/Å | 1.34143 |
| Radiation type | 1.3401270, 1.34 Ga $K_{\alpha}$ |
| $\Theta_{min}$/° | 2.509 |
| $\Theta_{max}$/° | 55.732 |
| Measured Refl's. | 88118 |
| Indep't Refl's | 23461 |
| Refl's I≥2 *s*(I) | 18292 |
| $R_{int}$ | 0.0342 |
| Parameters | 1436 |
| Restraints | 90 |
| Largest Peak | 1.069 |
| Deepest Hole | -0.792 |
| GooF | 1.039 |
| $wR_2$ (all data) | 0.1672 |
| $wR_2$ | 0.1496 |
| $R_1$ (all data) | 0.0833 |
| $R_1$ | 0.0606 |

## 2.4. NMR spectra

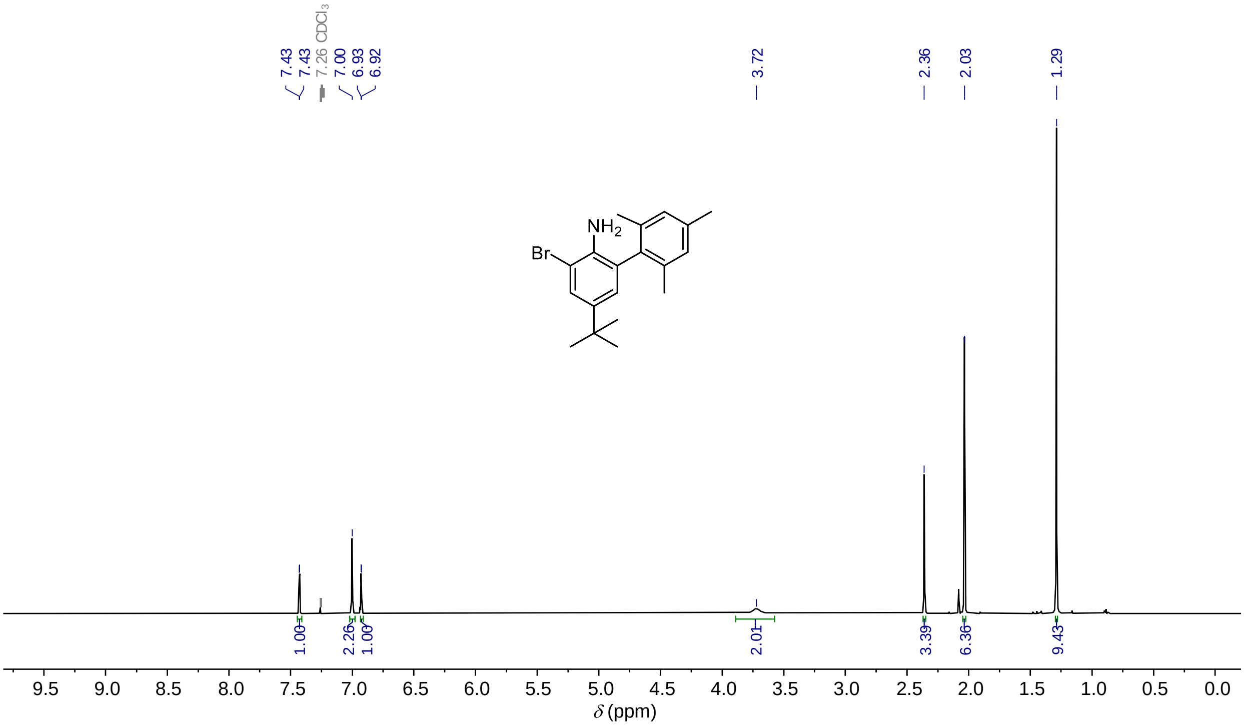


**Supplementary Fig. 2.** $^{1}H$ NMR spectrum of compound **3** (500 MHz, $CDCl_3$).

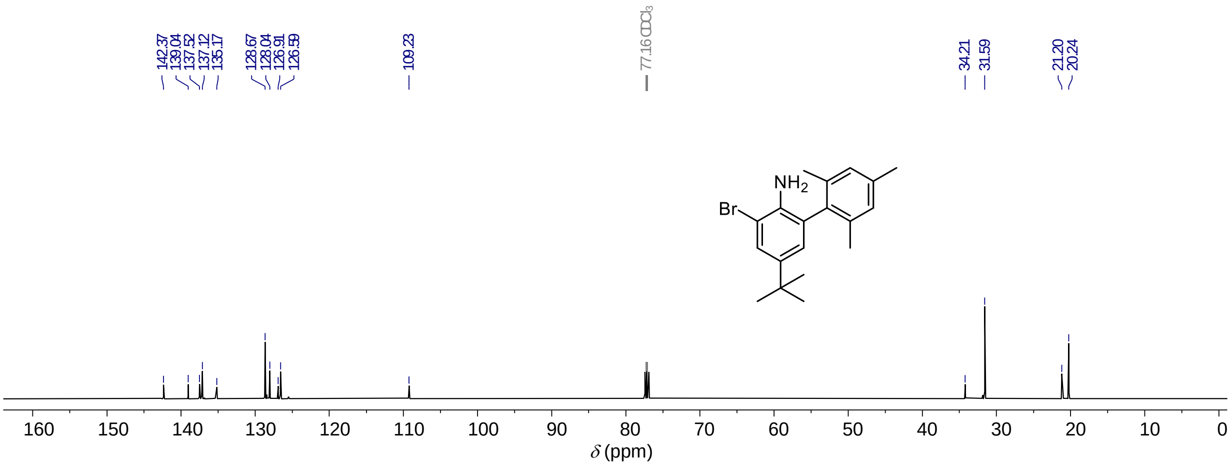


**Supplementary Fig. 3.** $^{13}C\{^{1}H\}$ NMR spectrum of compound **3** (125 MHz, $CDCl_3$).

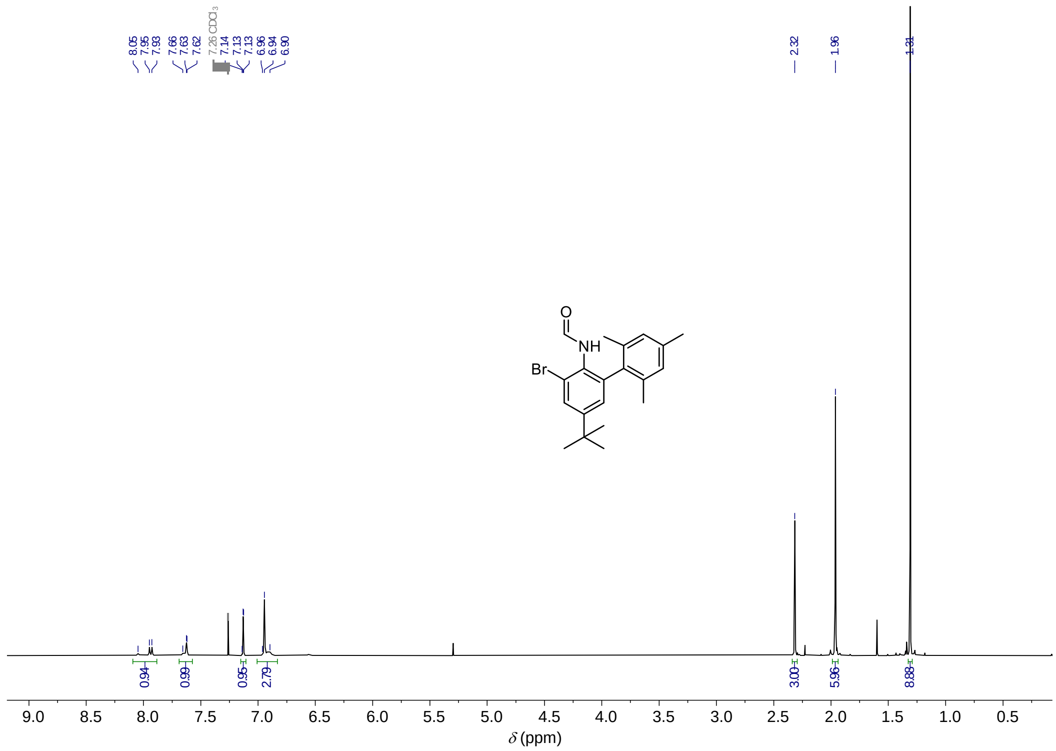

**Supplementary Fig. 4.** [1]H NMR spectrum of compound **4** (250 MHz, $CDCl_3$).

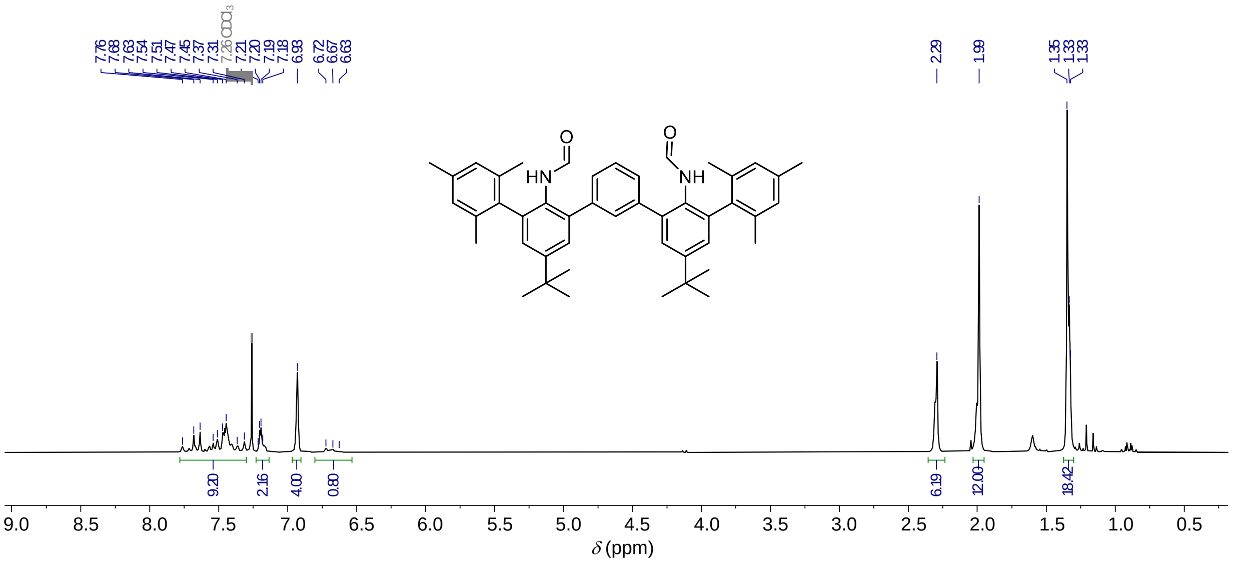

**Supplementary Fig. 5.** [1]H NMR spectrum of compound **6** (250 MHz, $CDCl_3$).

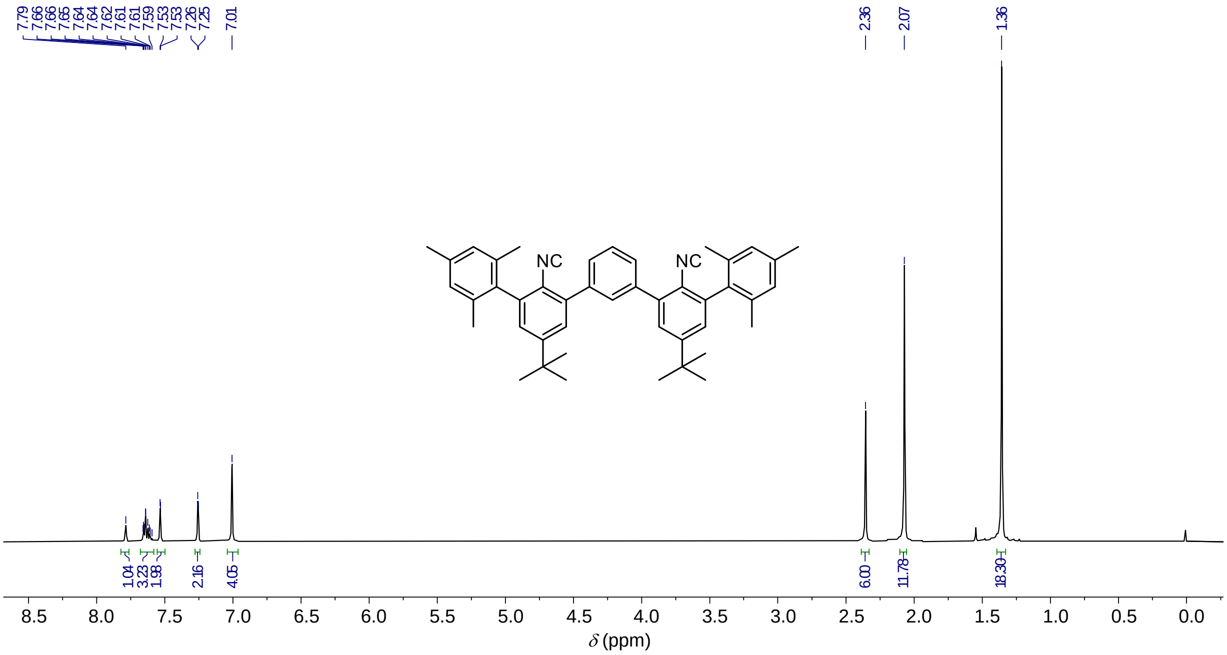

**Supplementary Fig. 6.** $^{1}$H NMR spectrum of compound L$^{Mes}$ (500 MHz, $CDCl_3$).

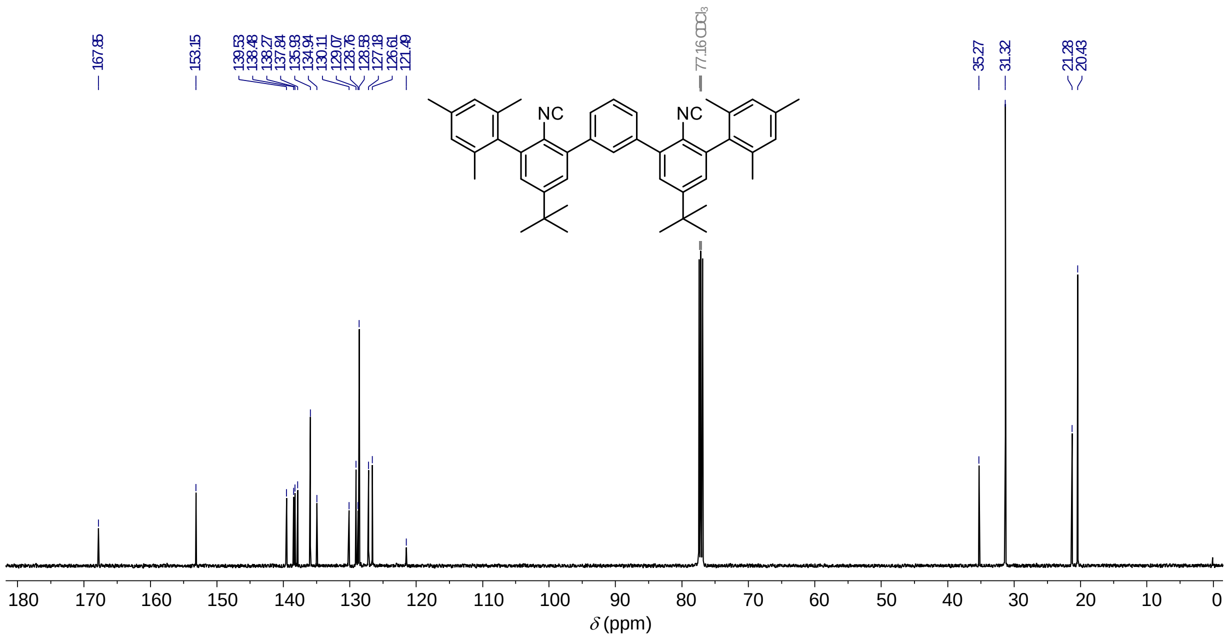

**Supplementary Fig. 7.** $^{13}$C{$^{1}$H} NMR spectrum of compound L$^{Mes}$ (125 MHz, $CDCl_3$).

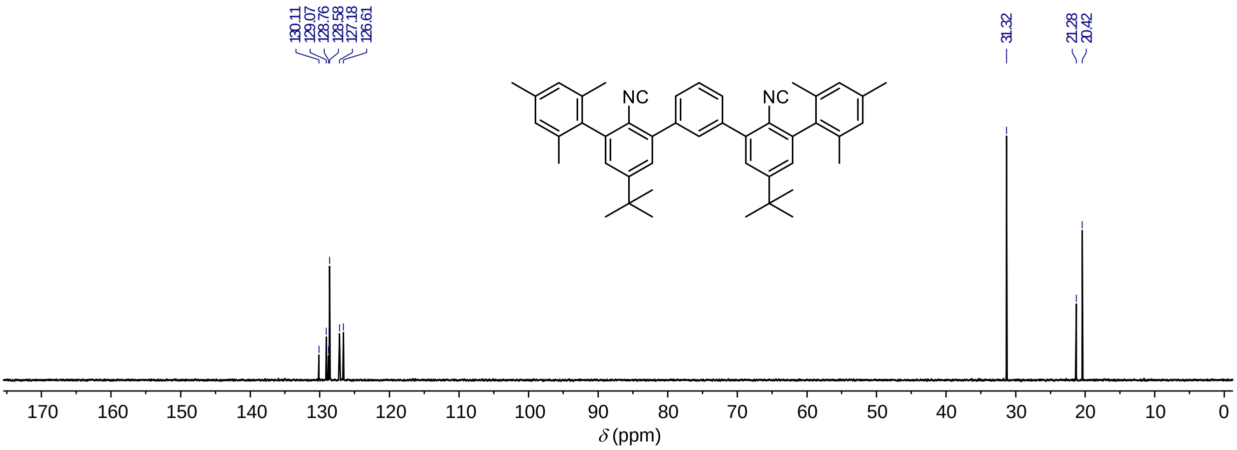


**Supplementary Fig. 8.** $^{13}C\{^{1}H\}$ DEPT-135 NMR spectrum of compound $L^{Mes}$ (125 MHz, $CDCl_3$).

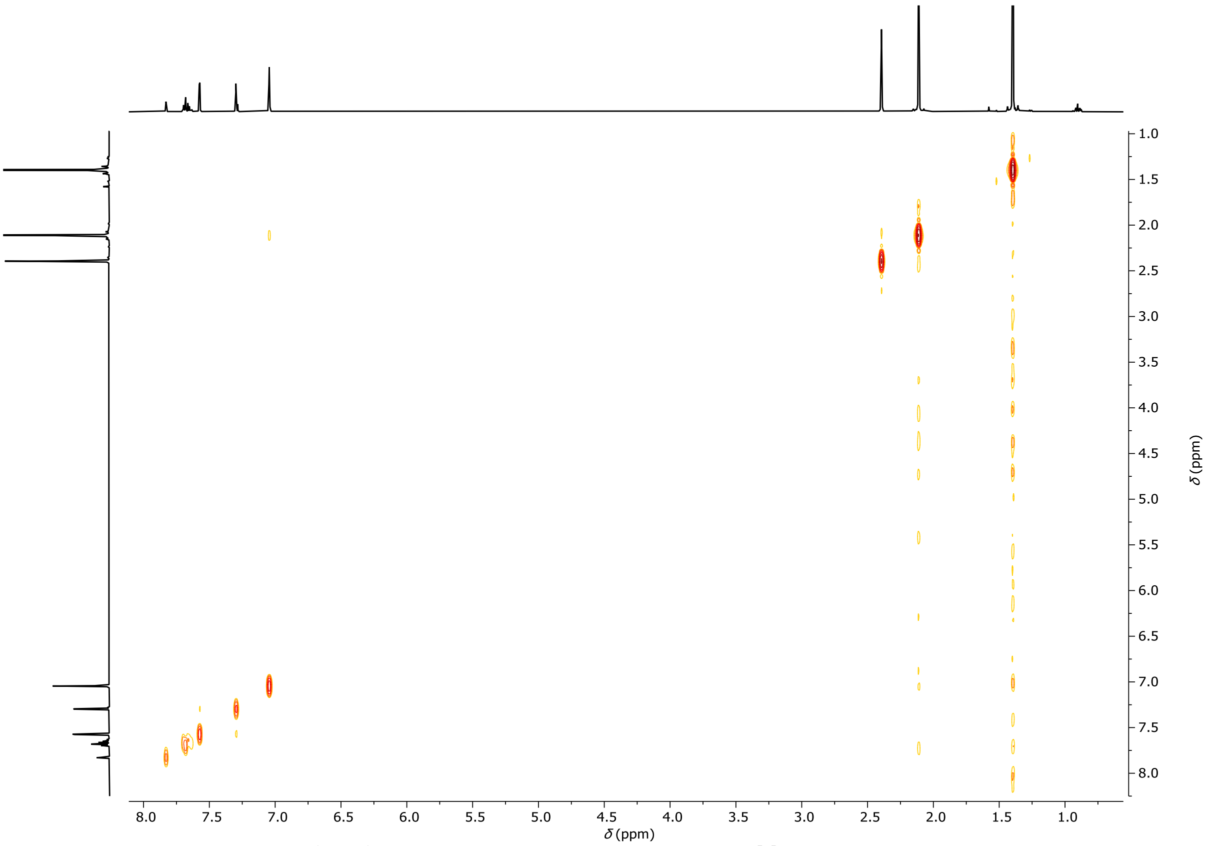


**Supplementary Fig. 9.** $^{1}H$, $^{1}H$ COSY NMR spectrum of $L^{Mes}$ (500MHz, 500 MHz, $CDCl_3$).

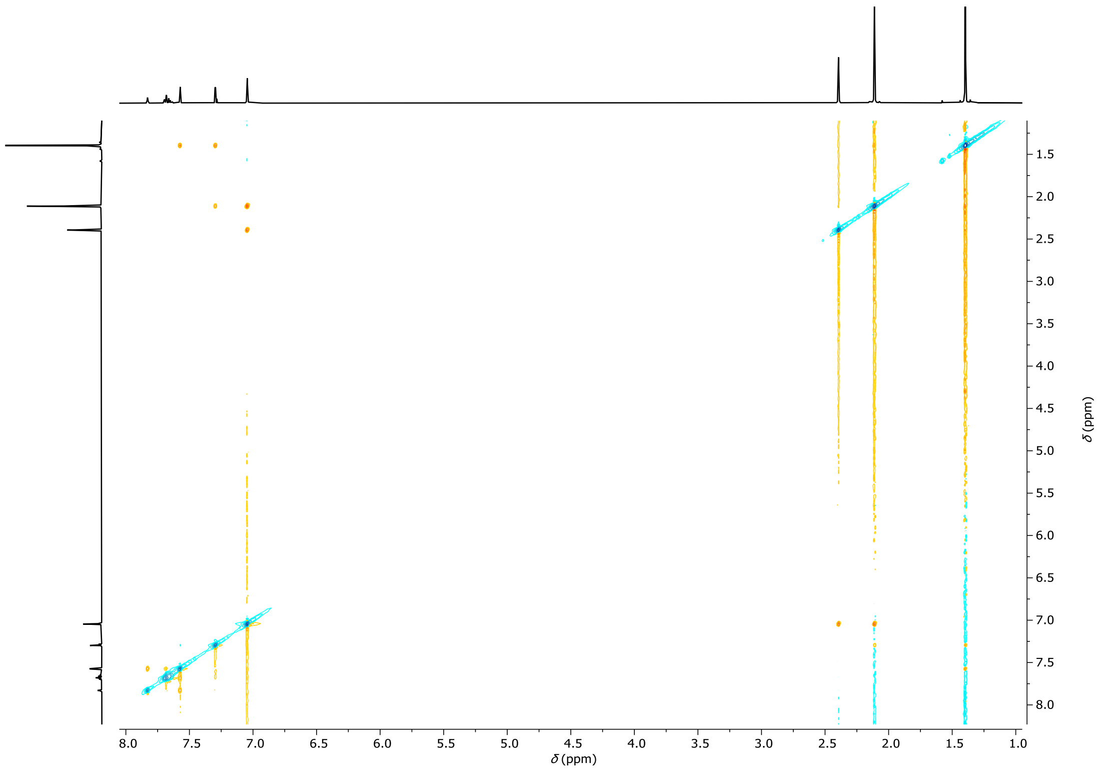


**Supplementary Fig. 10.** $^{1}H$, $^{1}H$ NOESY NMR spectrum of L$^{Mes}$ (500MHz, 500 MHz, $CDCl_3$).

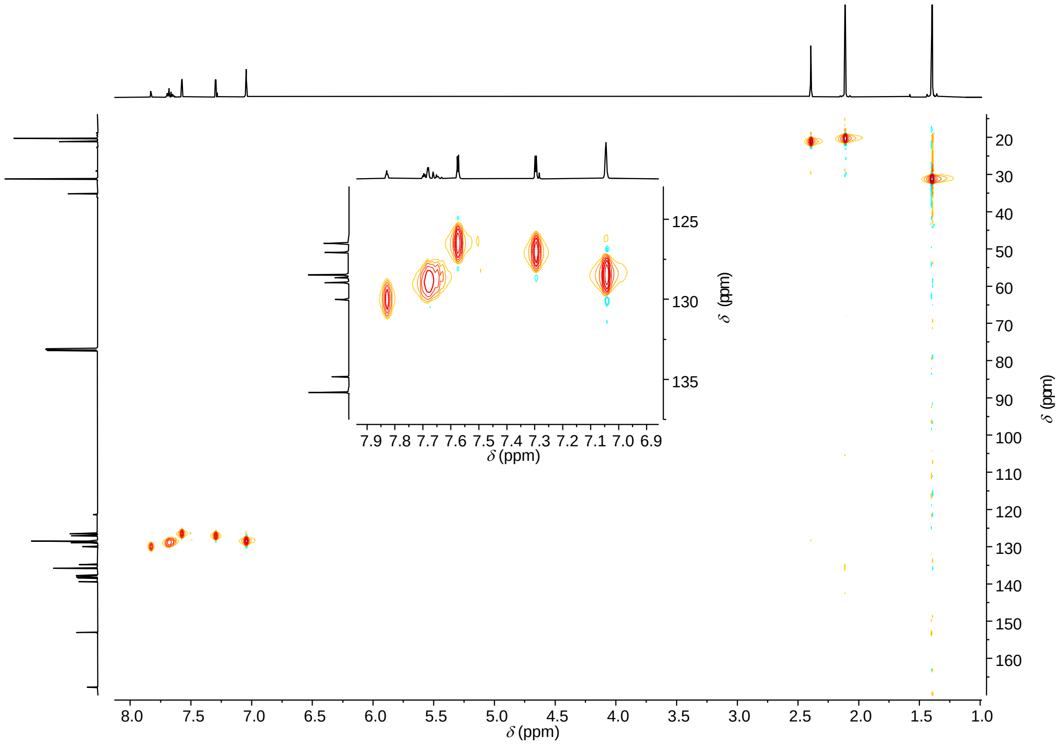


**Supplementary Fig. 11.** HMQC NMR spectrum of compound L$^{Mes}$ (500 MHz, 125 MHz, $CDCl_3$). Inset: Zoom portion of the aromatic region of HMQC spectrum.

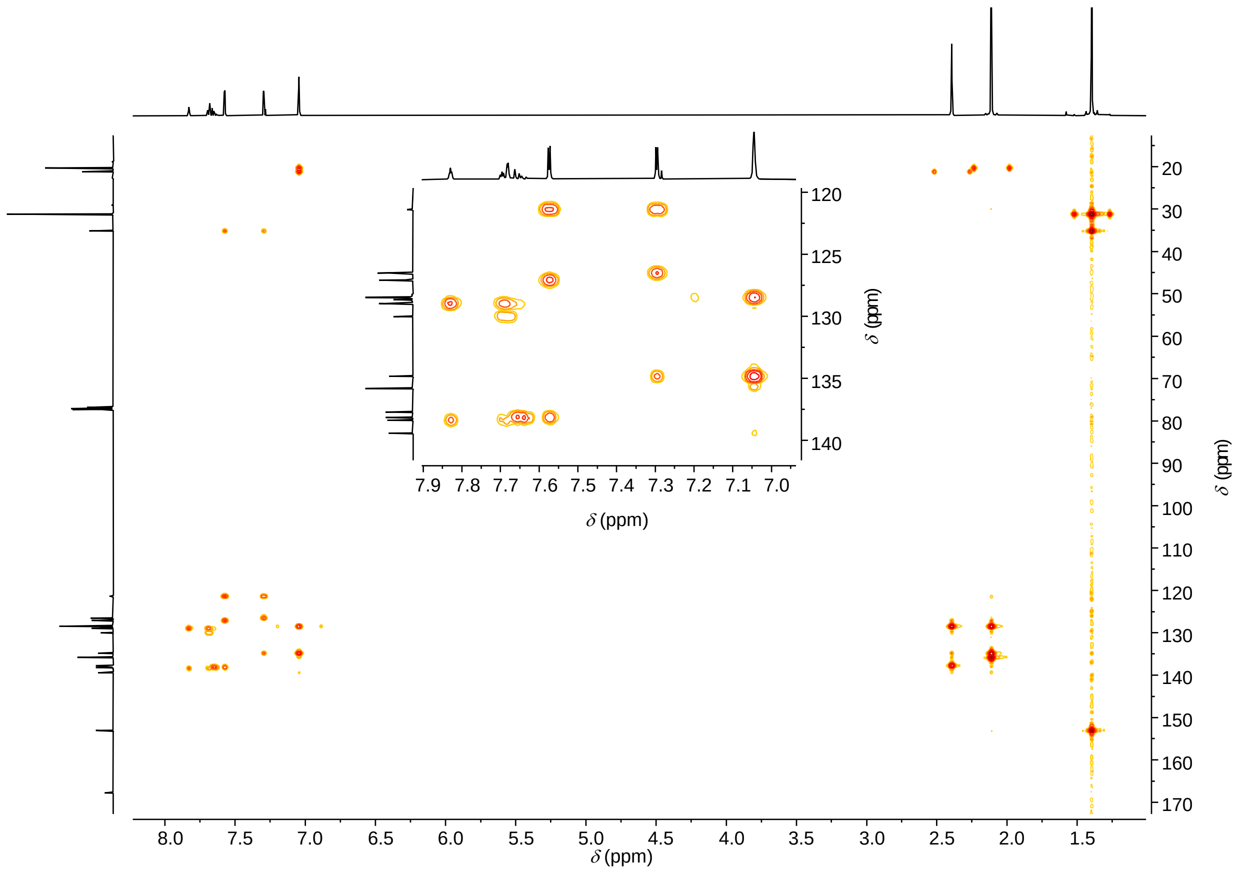

**Supplementary Fig. 12.** HMBC NMR spectrum of compound $L^{Mes}$ (500 MHz, 125 MHz, $CDCl_3$). Inset: Zoom portion of the aromatic region of HMBC spectrum.

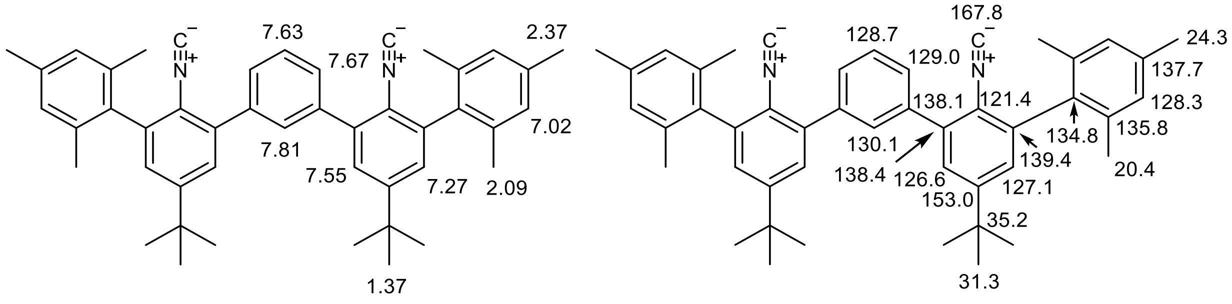

**Supplementary Fig. 13.** Assignment of the measured $^1$H-NMR (left) and $^{13}$C-NMR (right) resonances of $L^{Mes}$ in $CDCl_3$ in Supplementary Fig. 6 and 7, respectively, based on DEPT-135 and 2D NMR measurements (Supplementary Fig. 8-12).

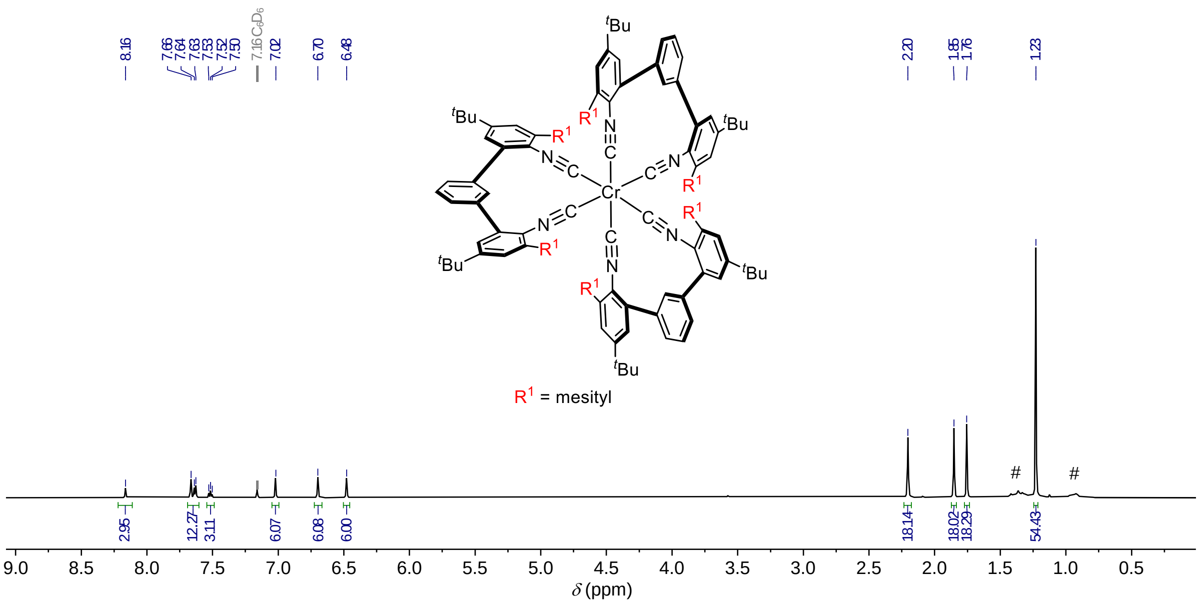


**Supplementary Fig. 14.** $^{1}$H NMR spectrum of [Cr(L$^{Mes}$)$_3$] (600 MHz, $C_6D_6$). (The # symbol designates proton resonances caused by grease used for the Schlenk line).

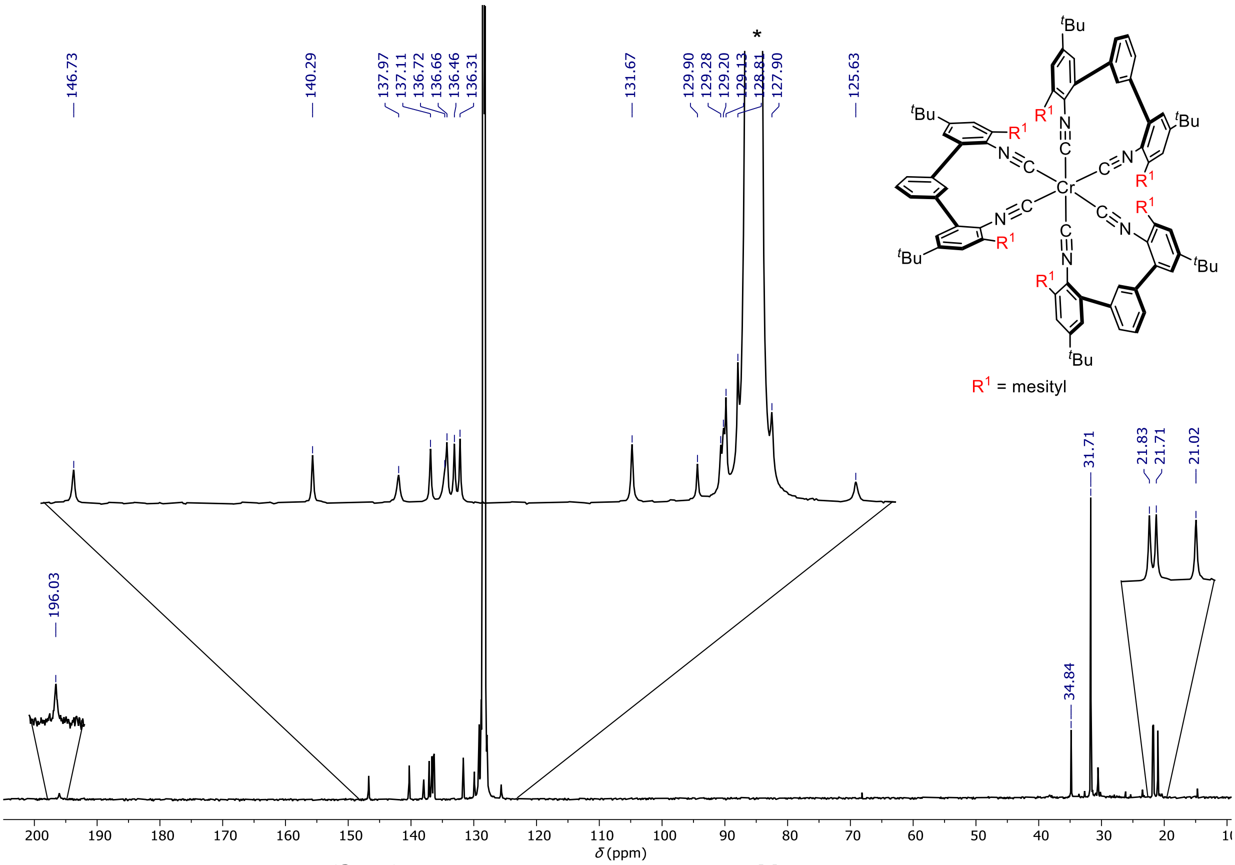


**Supplementary Fig. 15.** $^{13}$C{$^{1}$H} NMR spectrum of [Cr(L$^{Mes}$)$_3$] (150 MHz, $C_6D_6$ (*)).

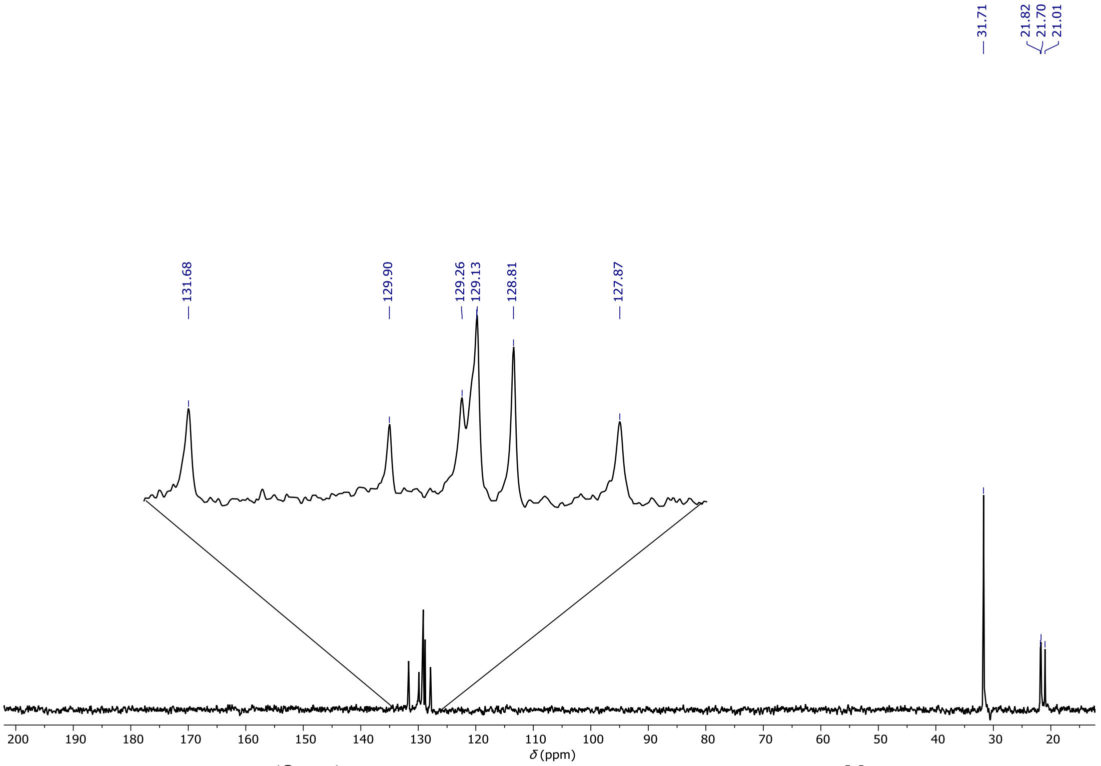


**Supplementary Fig. 16.** $^{13}C\{^{1}H\}$ DEPT-135 NMR spectrum of $[Cr(L^{Mes})_3]$ (150 MHz, $C_6D_6$).

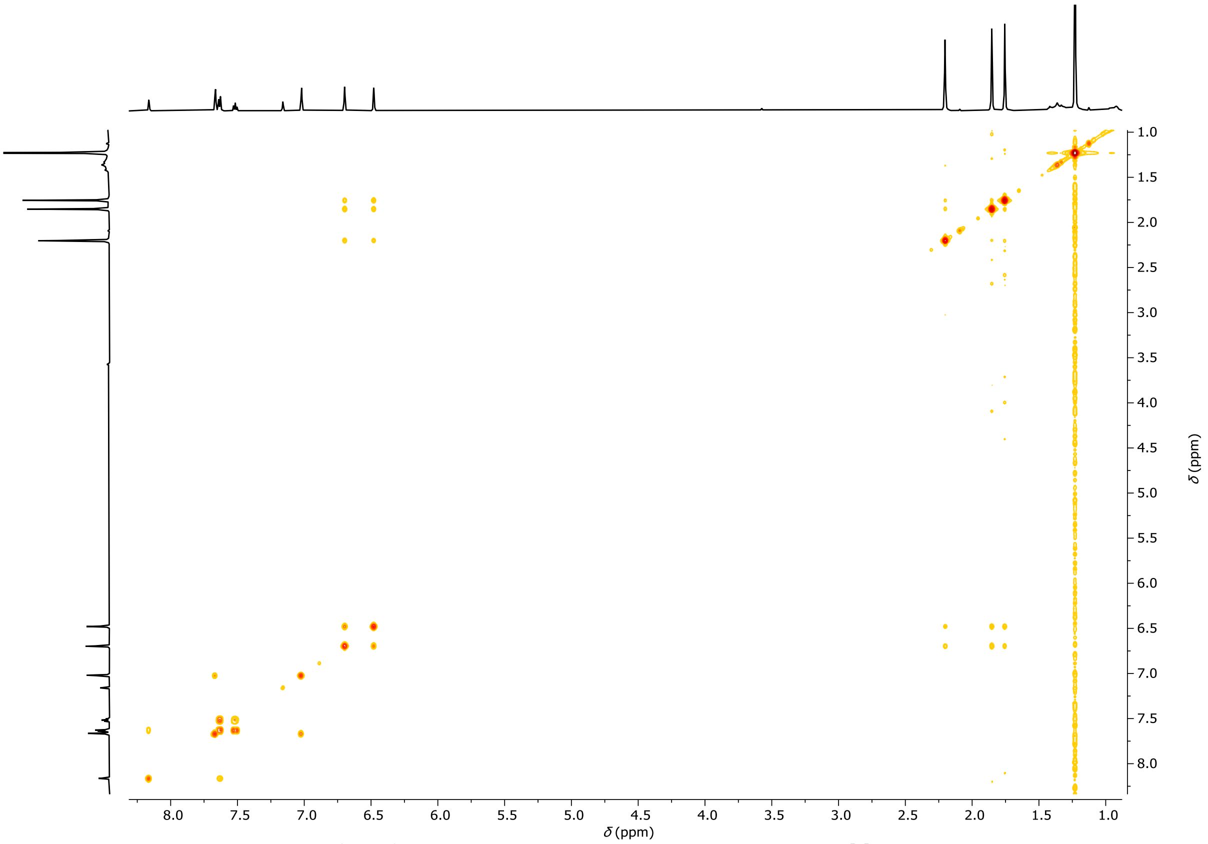


**Supplementary Fig. 17.** $^{1}H$, $^{1}H$ COSY NMR spectrum of $[Cr(L^{Mes})_3]$ (600MHz, 600 MHz, $C_6D_6$).

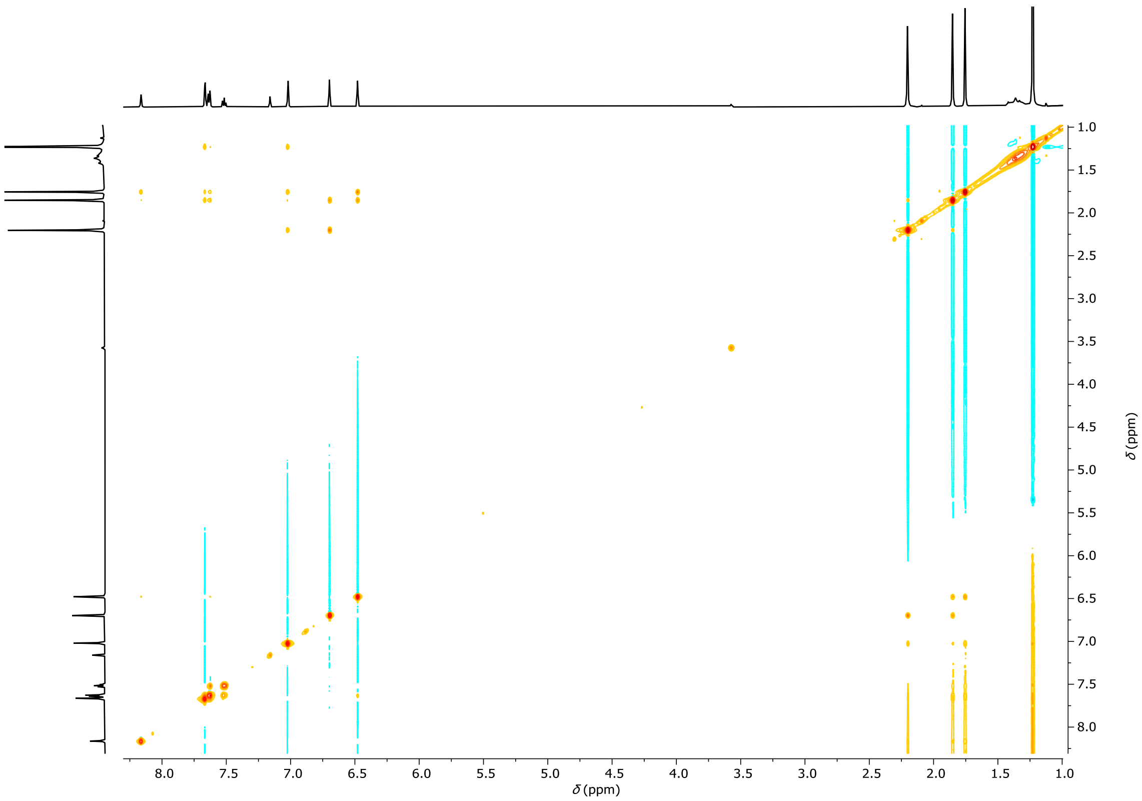


**Supplementary Fig. 18.** $^{1}H$, $^{1}H$ NOESY NMR spectrum of $[Cr(L^{Mes})_3]$ (600MHz, 600 MHz, $C_6D_6$).

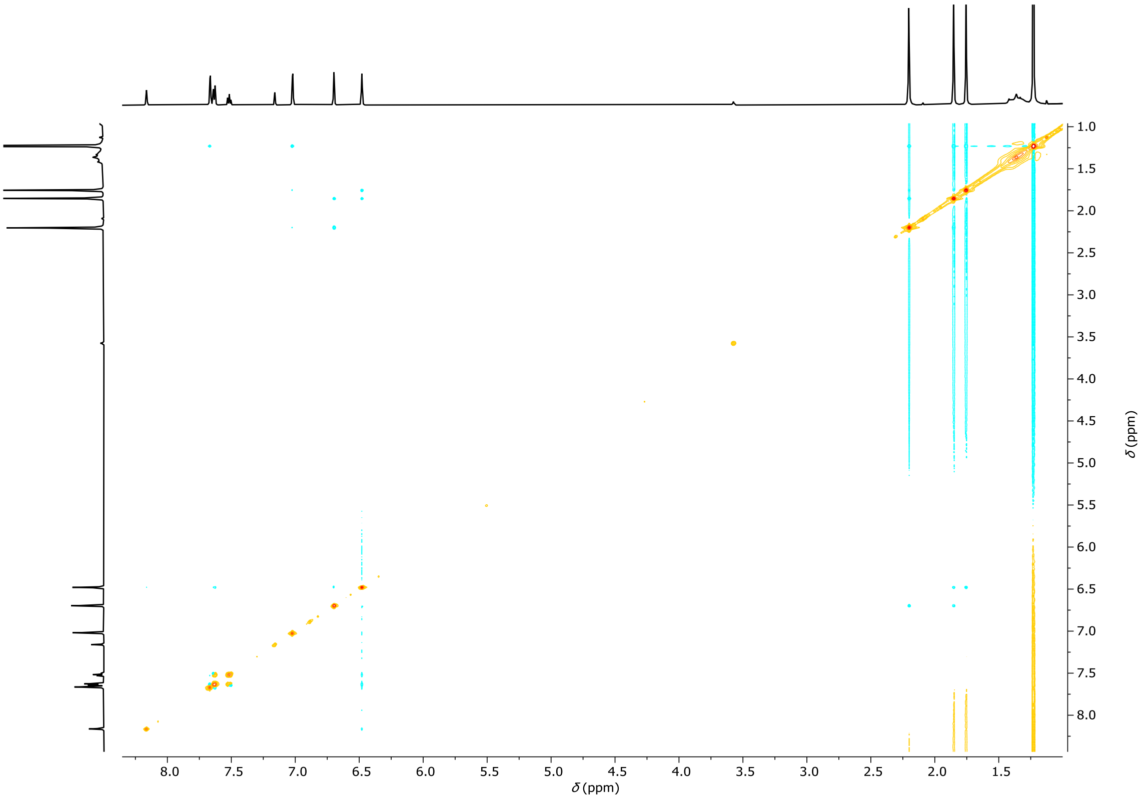


**Supplementary Fig. 19.** $^{1}H$, $^{1}H$ ROESY NMR spectrum of $[Cr(L^{Mes})_3]$ (600MHz, 600 MHz, $C_6D_6$).

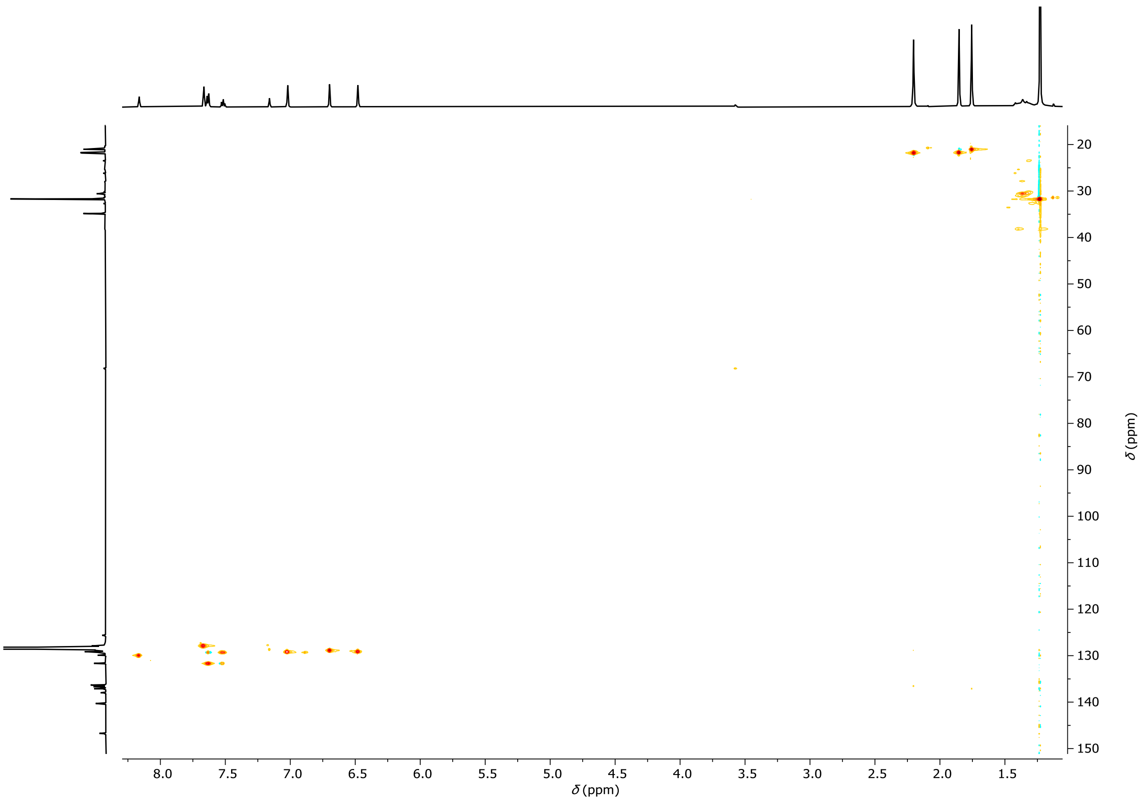


**Supplementary Fig. 20.** HSQC NMR spectrum of [Cr(L$^{Mes}$)$_3$] (600MHz, 150 MHz, $C_6D_6$).

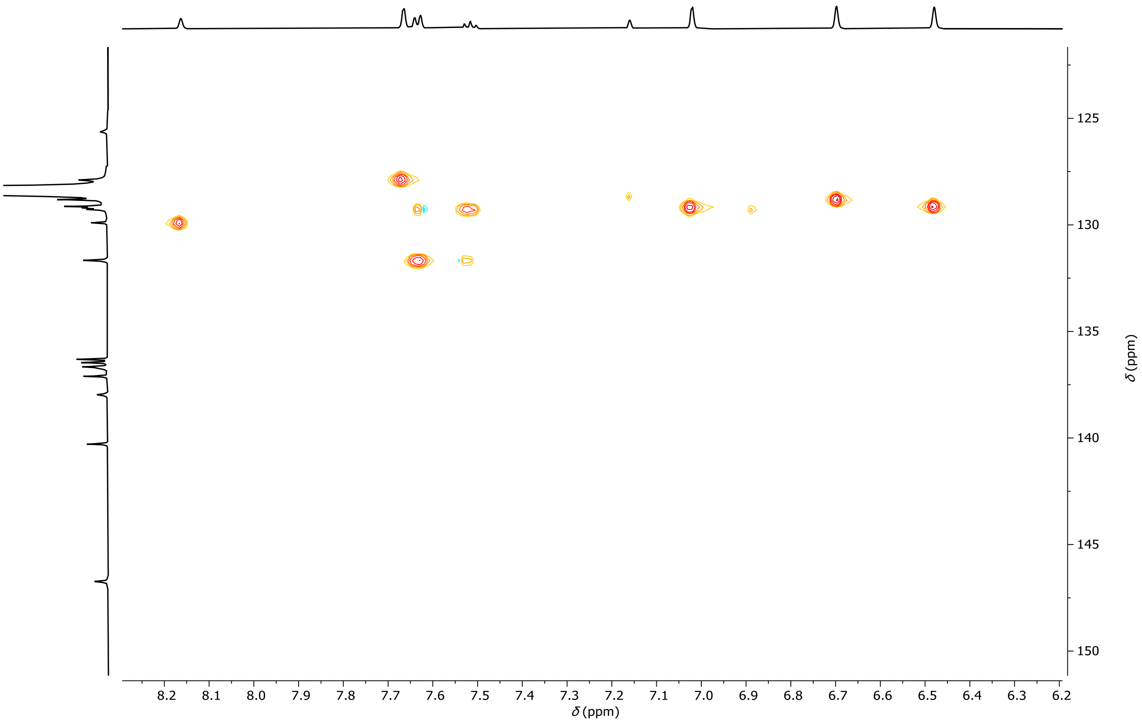


**Supplementary Fig. 21.** Zoom portion of the aromatic region of HSQC spectrum in Supplementary Fig. 20.

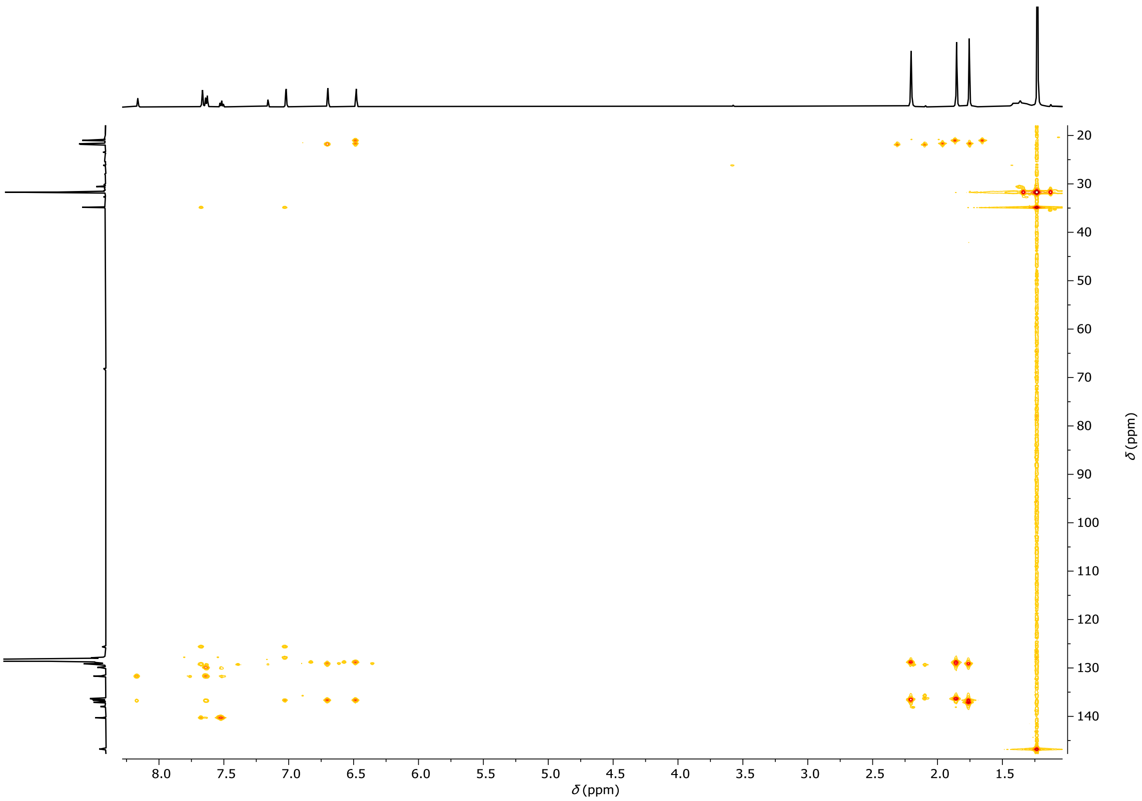


**Supplementary Fig. 22.** HMBC NMR spectrum of [Cr(L$^{Mes}$)$_3$] (600MHz, 150 MHz, $C_6D_6$).

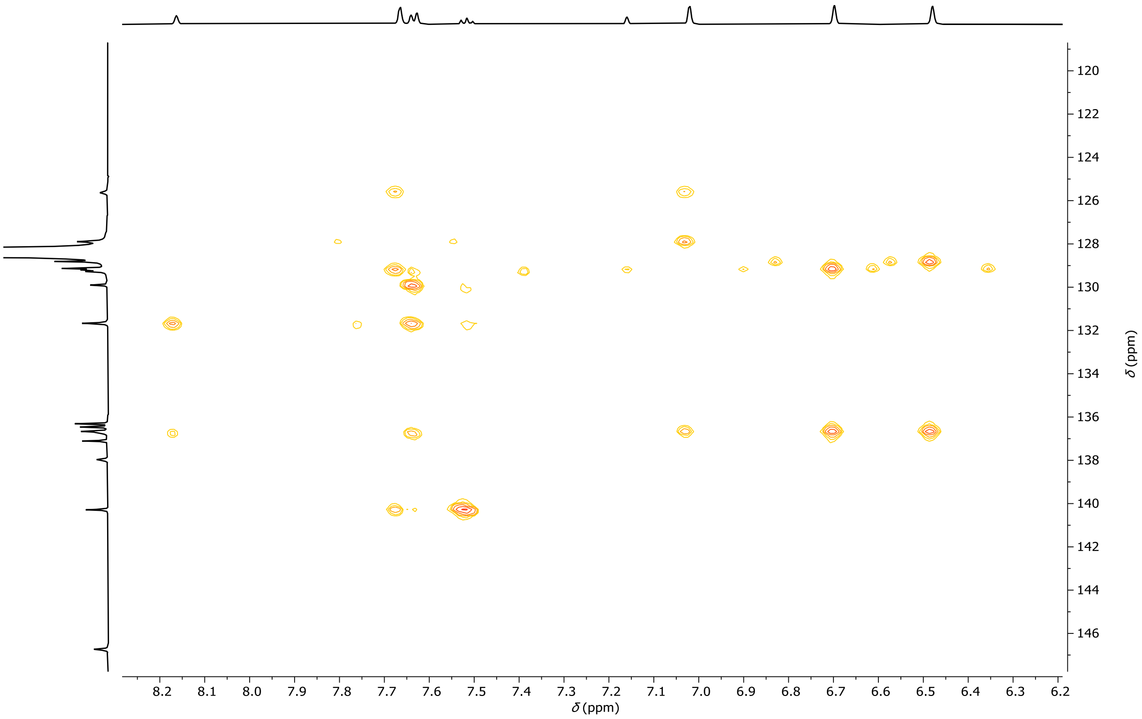


**Supplementary Fig. 23.** Zoom portion of the aromatic region of HMBC spectrum in Supplementary Fig. 22.

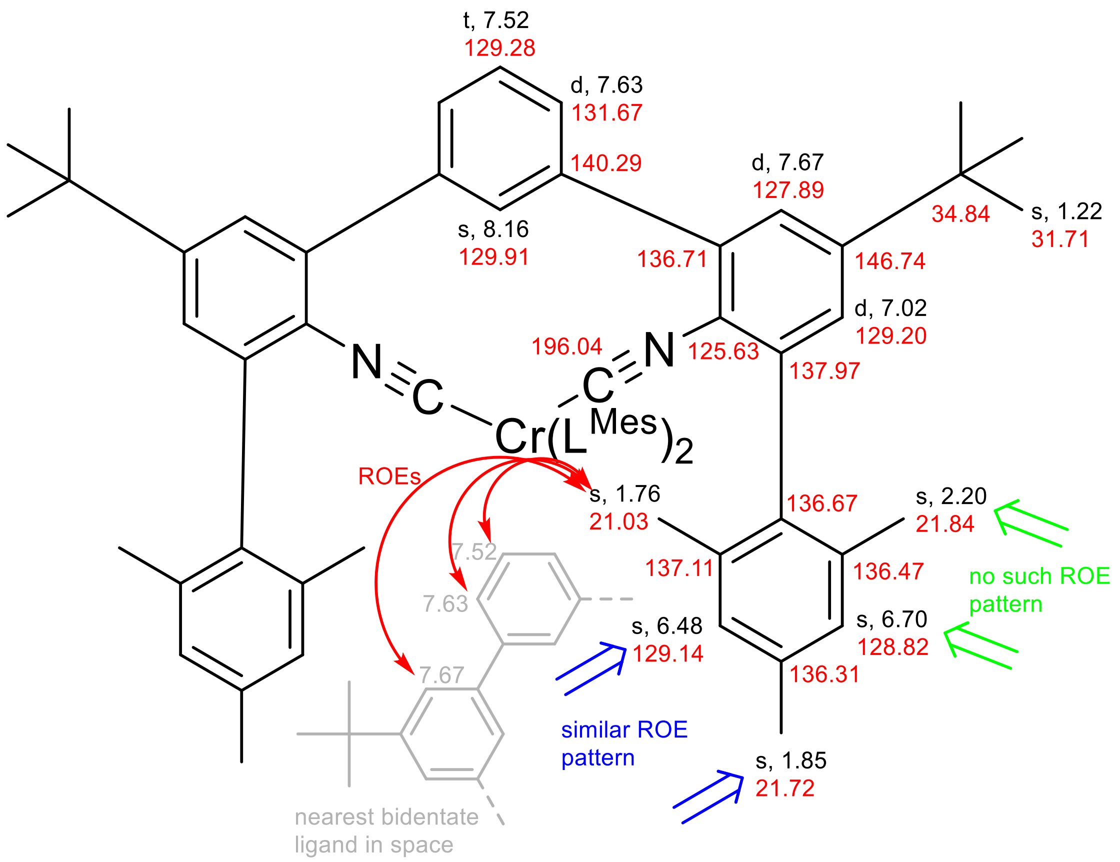


**Supplementary Fig. 24.** Assignment of the measured $^{1}$H-NMR (black) and $^{13}$C-NMR (red) resonances of [Cr(L$^{Mes}$)$_3$] in $C_6D_6$ in Supplementary Fig. 14 and 15, respectively, based on DEPT-135 and 2D NMR measurements (Supplementary Fig. 16-23).

## 2.5. HR-ESI mass spectra

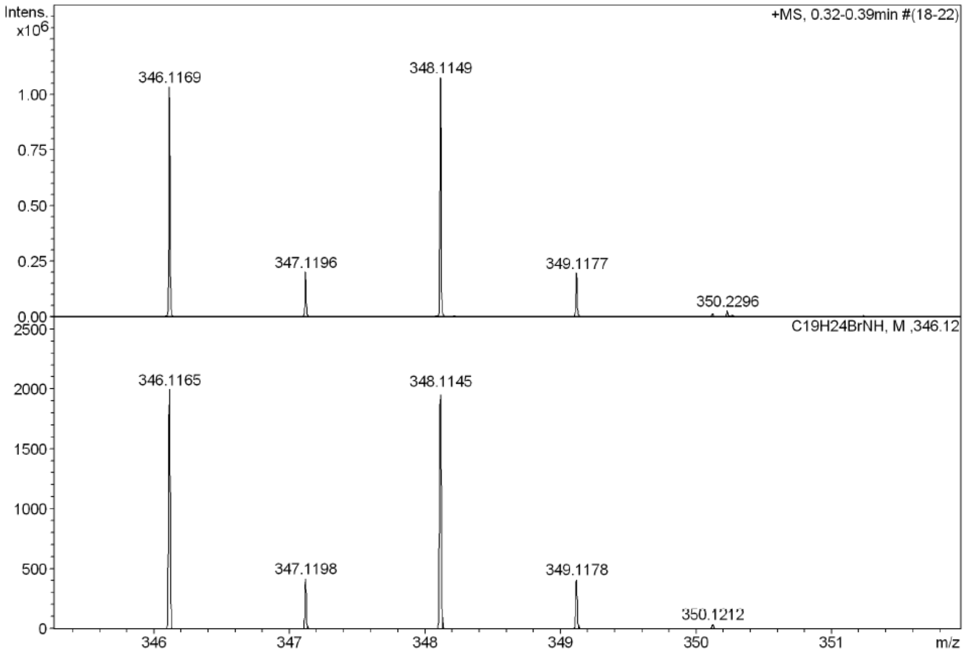


**Supplementary Fig. 25.** Top: HRMS-ESI (positive ions) mass spectrum of compound **3**. Bottom: Simulated mass spectrum of [**3**+H]$^+$.

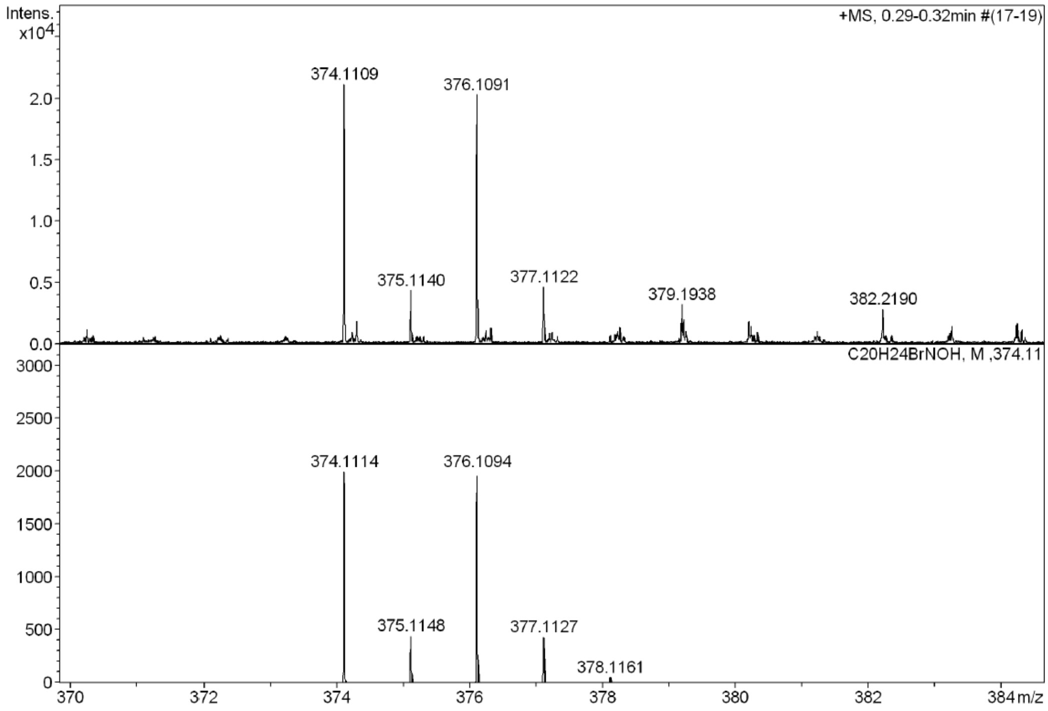


**Supplementary Fig. 26.** Top: HRMS-ESI (positive ions) mass spectrum of compound **4**. Bottom: Simulated mass spectrum of [**4**+H]$^+$.

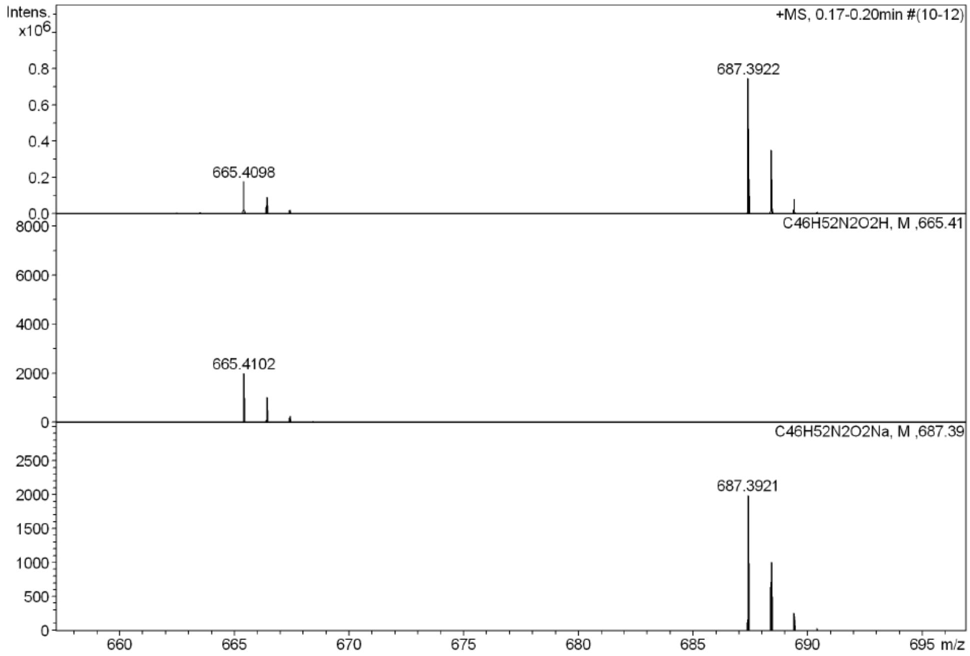


**Supplementary Fig. 27.** Top: HRMS-ESI (positive ions) mass spectrum of compound **6**. Middle, Bottom: Simulated mass spectra of [**6**+H]$^+$ and [**6**+Na]$^+$, respectively.

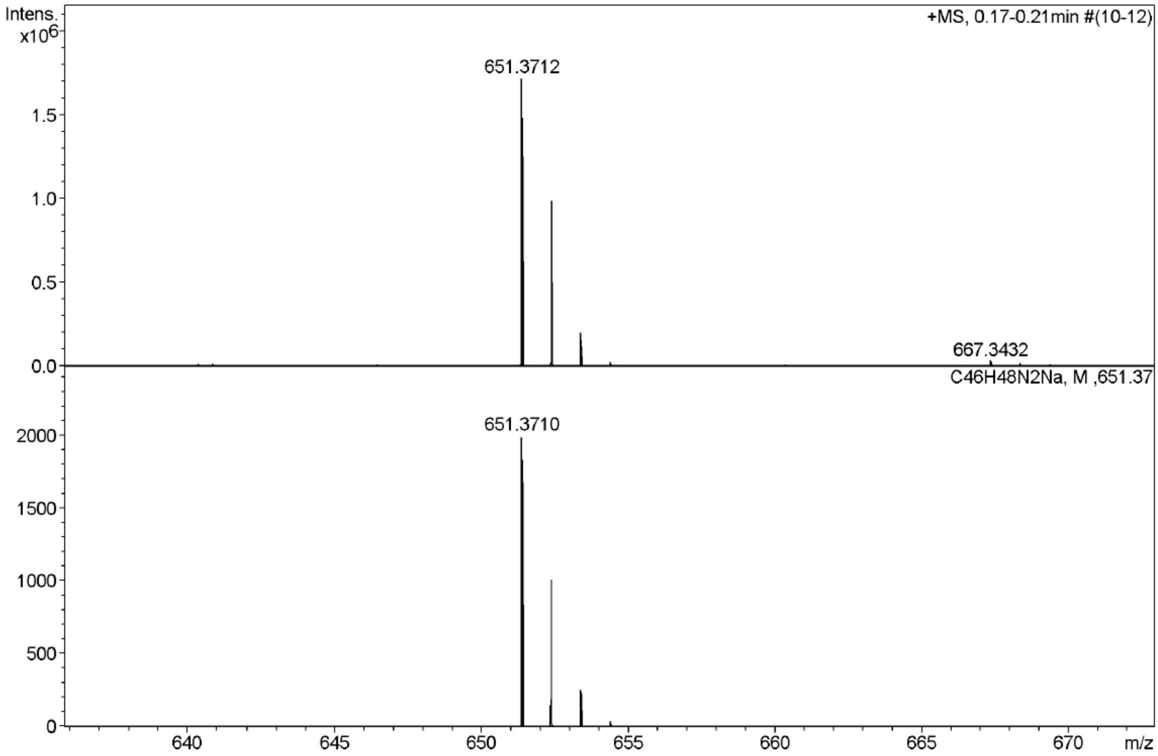


**Supplementary Fig. 28.** Top: HRMS-ESI (positive ions) mass spectrum of compound L$^{Mes}$. Bottom: Simulated mass spectrum of [L$^{Mes}$+Na]$^+$.

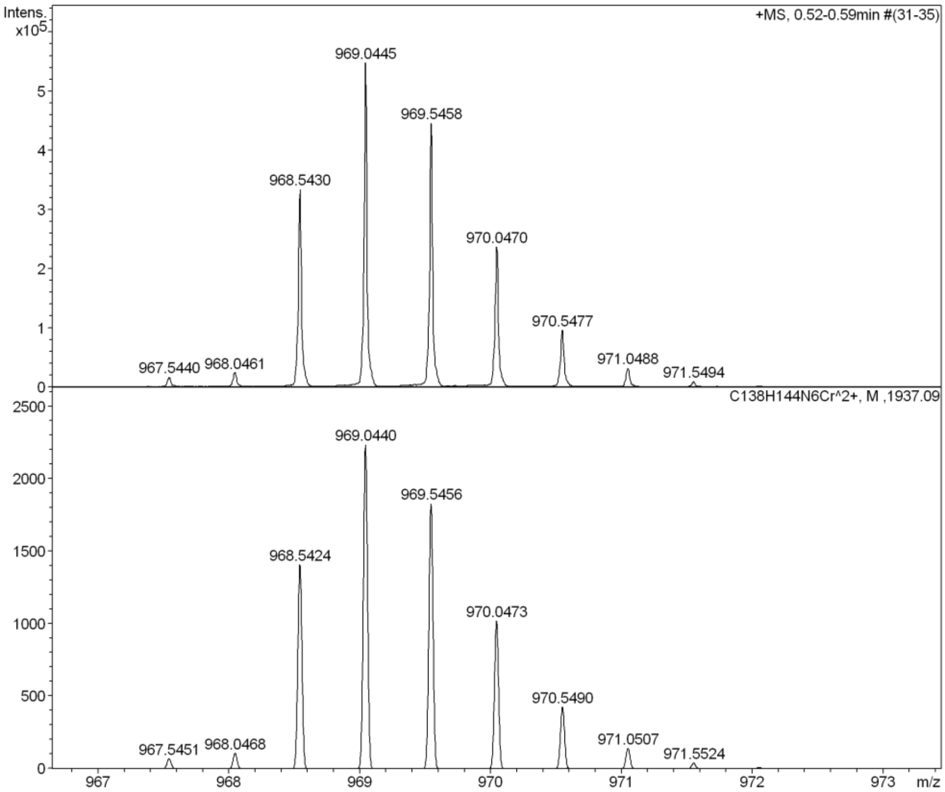


**Supplementary Fig. 29.** Top: HRMS-ESI (positive ions) mass spectrum of compound $[Cr(L^{Mes})_3]$. Bottom: Simulated mass spectrum of $[Cr(L^{Mes})_3]^{2+}$.

## 2.6. FTIR spectra

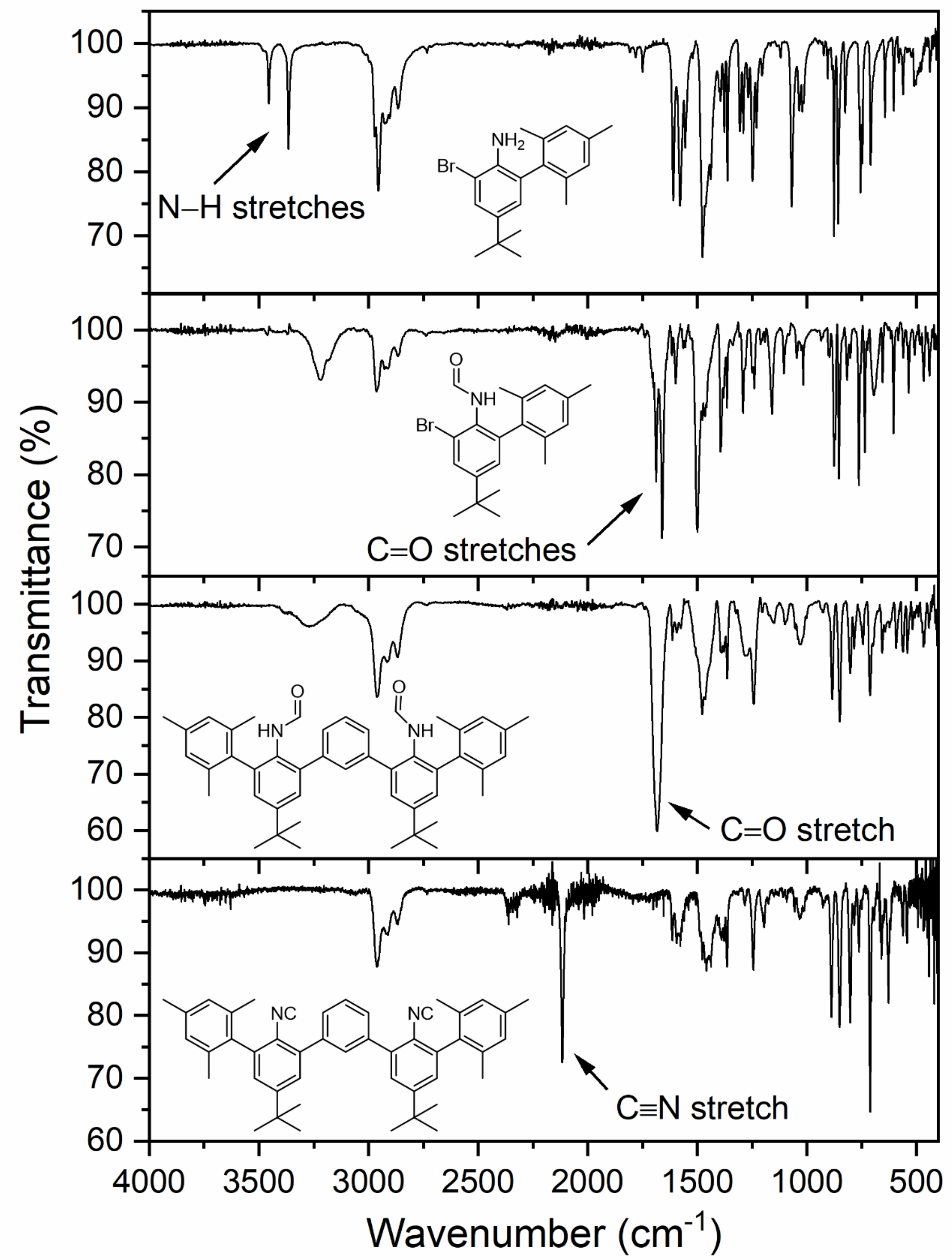


**Supplementary Fig. 30.** Stacked solid state IR spectra of **3**, **4**, **6** and $L^{Mes}$, respectively. The vibrational modes of the respective functional groups of the compounds are indicated.

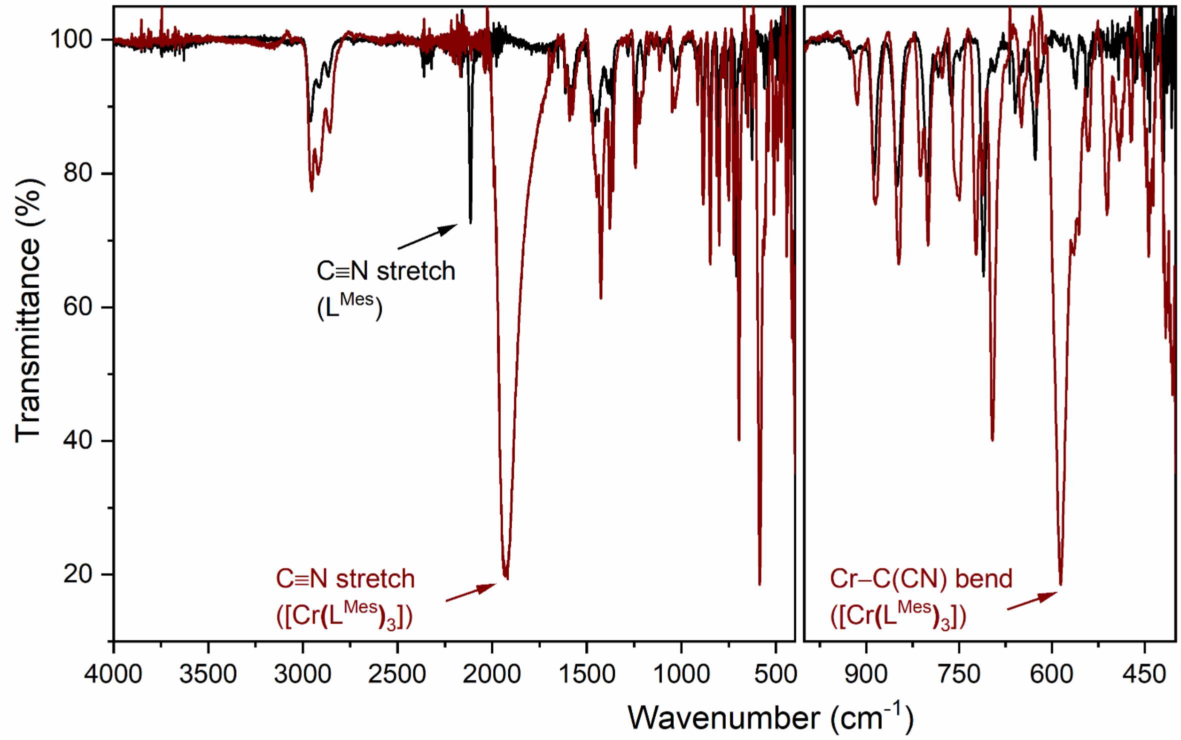


**Supplementary Fig. 31.** Solid state IR spectra of $L^{Mes}$ (black) and $[Cr(L^{Mes})_3]$ (brown). Left: Full spectrum, right: Zoom of the region between 1000 $cm^{-1}$ and 400 $cm^{-1}$ to highlight the Cr–C bending frequency at 586 $cm^{-1}$. The C≡N stretch frequencies of $L^{Mes}$ and $[Cr(L^{Mes})_3]$ are found at 2115 and 1930 $cm^{-1}$, respectively.

**2.7. Temperature dependent $^1$H NMR study of $[Cr(L^{Mes})_3]$**: To check the thermal stability of the $Cr^0$ complex in solution and also to investigate the potential rotation of the mesityl groups by monitoring the methyl signals at 2.14, 1.80 and 1.68 ppm, respectively, a $^1$H NMR study of $[Cr(L^{Mes})_3]$ in toluene-$d_8$ was carried out at elevated temperatures up to 388 K, as noted in Supplementary Fig. 32 below. The $Cr^0$ complex proved to be very stable even at 115 °C, and no free rotation of the mesityl groups was observed as no significant changes of the chemical shifts and line shapes were observed. This indicates the unsymmetric nature of the mesityl groups due to steric demands around the chromium center, as seen in the X-ray structure. The rigid, interlocked arrangement of the mesityl groups that was demonstrated in solution at 298 K by the distinctly different NOE patterns of their methyl groups (Supplementary Fig. 18, 19 and 24) as well as by the absence of EXSY peaks between the methyl resonances at 2.20 and 1.76 ppm in $C_6D_6$ is, therefore, also maintained at elevated temperatures.

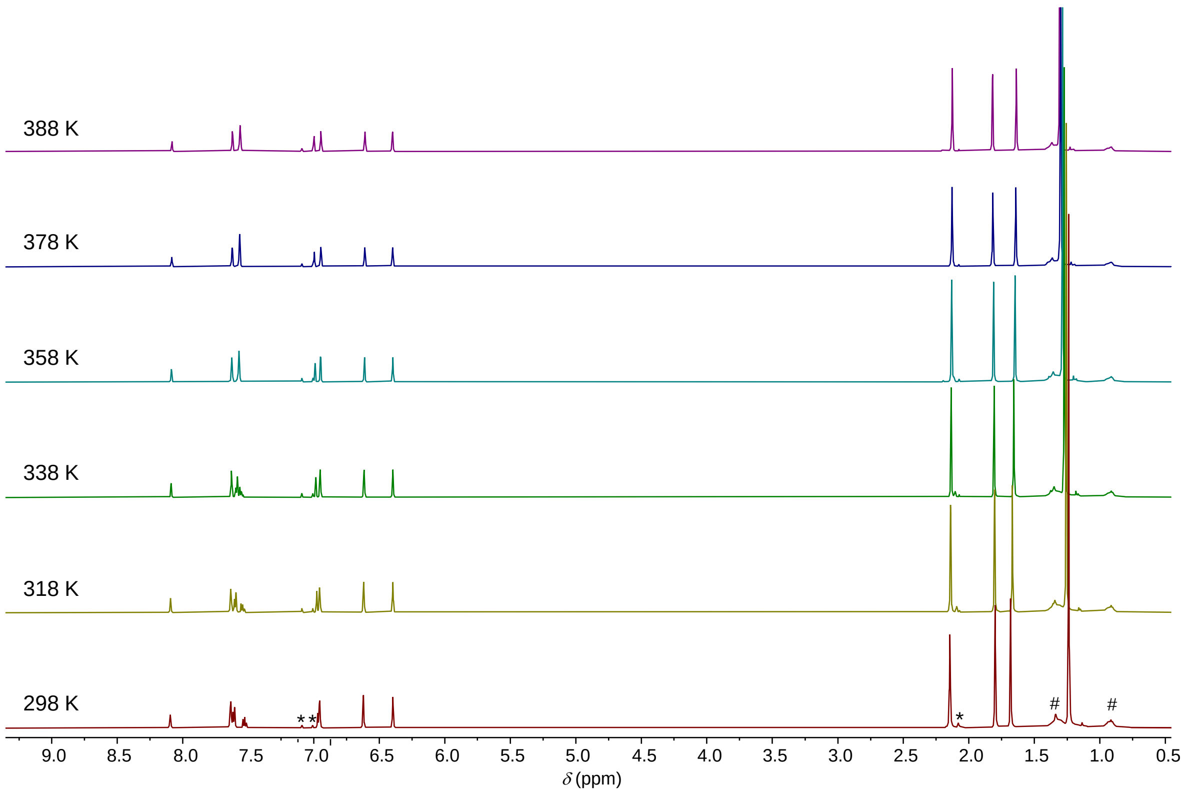


**Supplementary Fig. 32.** $^1$H NMR spectra (600 MHz) of [Cr(L$^{Mes}$)$_3$] in toluene-d$_8$ (*) at different temperatures as indicated in the figure, showing the high thermal stability of the complex. (The # symbol designates proton resonances caused by grease used for the Schlenk line).

**2.8. Air stability study of [Cr(L$^{Mes}$)$_3$] in solution**: Generally, Cr$^0$ arylisocyanide complexes are quite stable in the solid state but very unstable in solution in the presence of small amounts of air/moisture, which cause rapid decomposition. Due to the presence of bulky mesityl groups in the *ortho*-positions of the isocyanide donors, the metal center is well shielded as seen the X-ray structure of [Cr(L$^{Mes}$)$_3$], and hence we anticipated that the new Cr$^0$ complex is more stable in aerated solution. To test this hypothesis, a $^1$H NMR spectrum was recorded in dry and degassed benzene-d$_6$ initially under argon, and then the NMR tube was exposed to air by putting a needle through the cap, such that air could readily diffuse into the NMR tube while at the same time the solvent evaporation was kept minimal. $^1$H NMR spectra at different time interval were then collected, and only 3% decomposition to free ligand (considering that one equivalent of complex decomposes to three equivalents of ligands) was observed after 15 days (Supplementary Fig. 33).

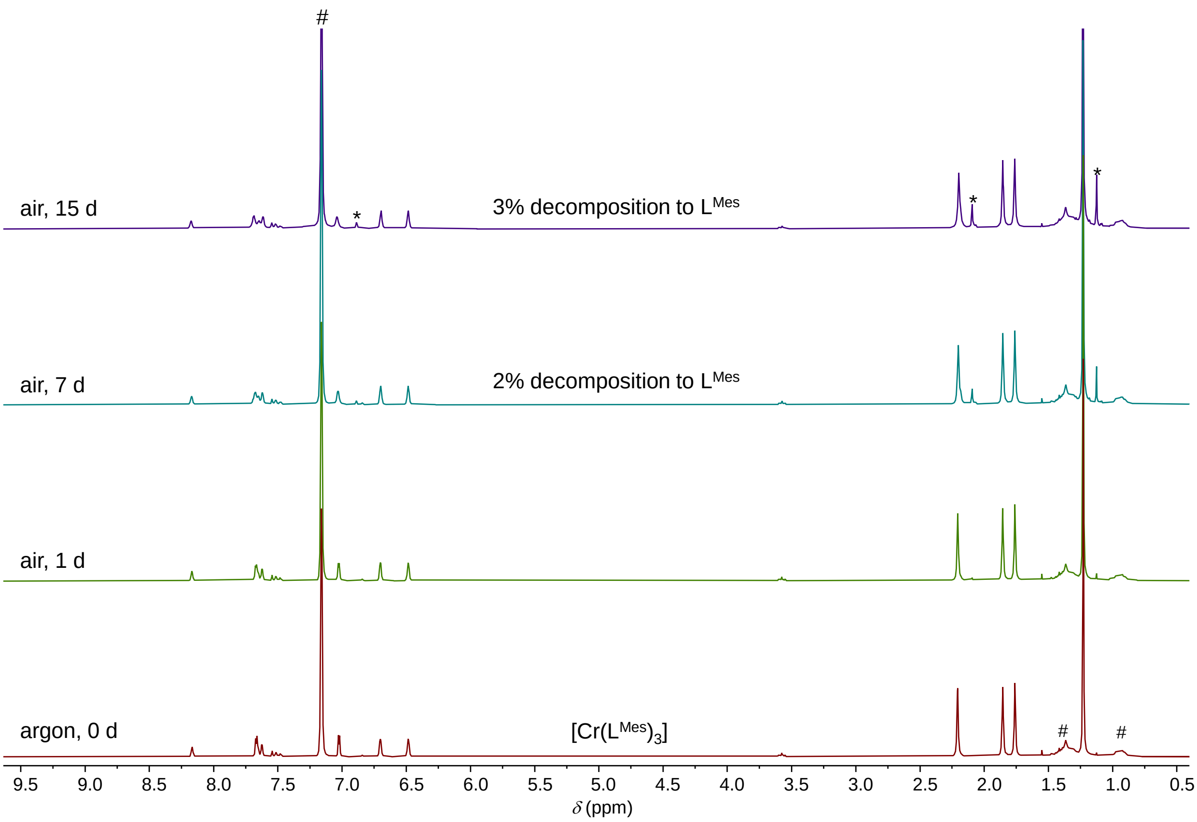


**Supplementary Fig. 33.** $^{1}H$ NMR spectra (250 MHz) of $[Cr(L^{Mes})_3]$ in benzene-$d_6$ (#) at different time intervals in the presence of air, showing the high insensitivity towards air in solution. * denotes the decomposition of the complex to free ligand (* pertinent signals from uncoordinated ligand). (The # symbol designates proton resonances caused by grease used for the Schlenk line).

## 2.9. Cyclic voltammetry

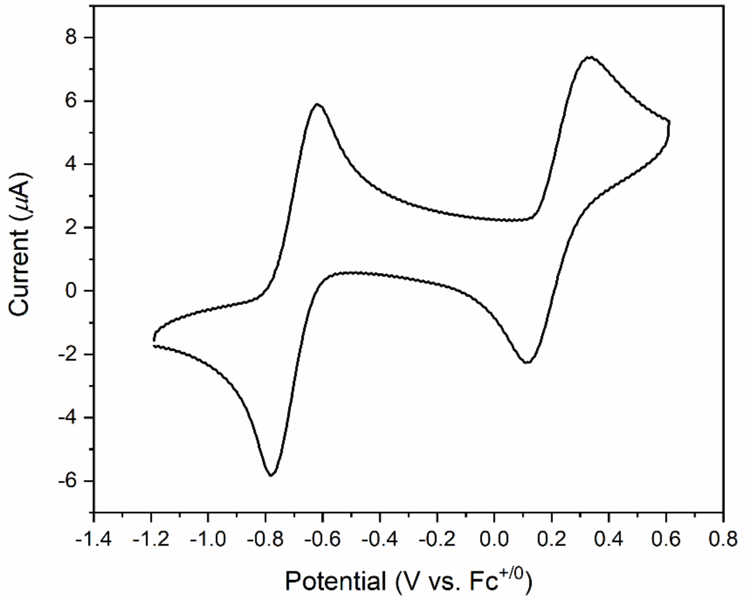


**Supplementary Fig. 34.** Cyclic voltammogram of 1 mM $[Cr(L^{Mes})_3]$ in tetrahydrofuran (THF) with 0.1 M of $(^{n}Bu_4N)(PF_6)$ as supporting electrolyte, recorded at a scan rate of 0.1 V/s. $E_{1/2}$ = -0.70 and 0.22 V vs $Fc^{+/0}$.

## 2.10. UV-Vis absorption and emission spectra

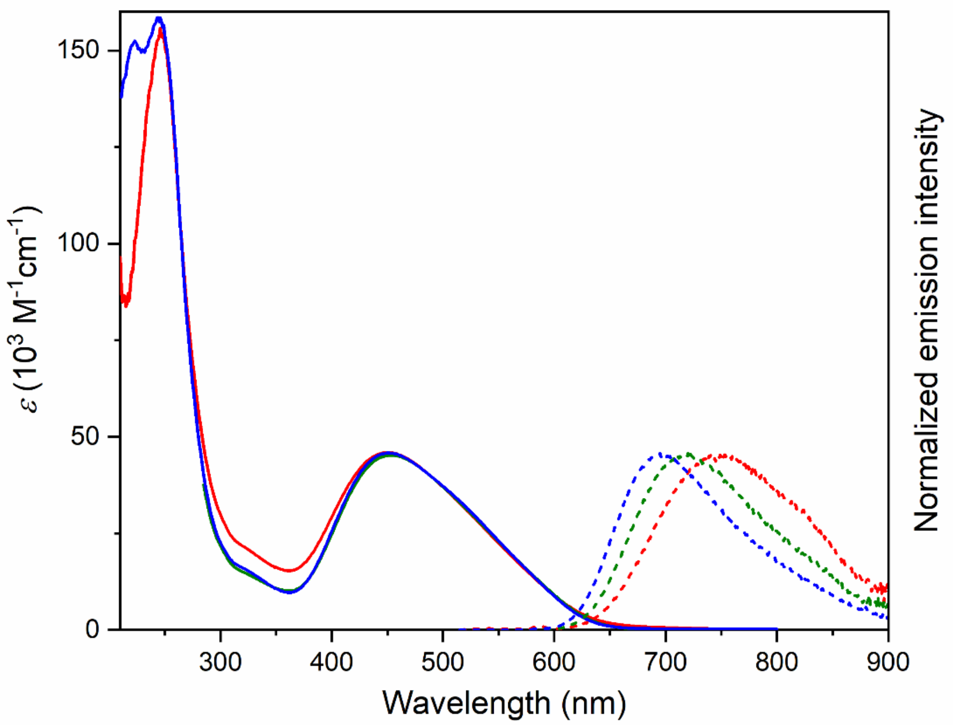


**Supplementary Fig. 35.** UV-Vis absorption (solid lines) and emission spectra (dotted lines, $\lambda_{exc}$ = 500 nm) of [Cr(L$^{Mes}$)$_3$] in deaerated THF (red), toluene (green) and cyclohexane (blue) solutions, respectively, at 20 °C.

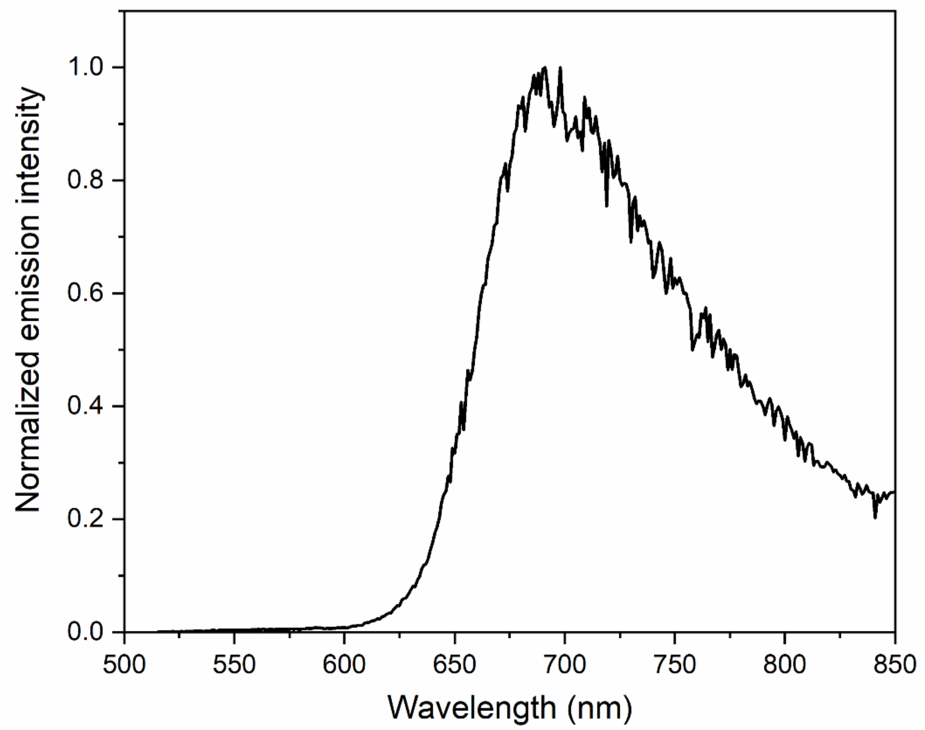


**Supplementary Fig. 36.** Emission spectrum ($\lambda_{exc}$ = 500 nm) of [Cr(L$^{Mes}$)$_3$] in 2-MeTHF at 77 K.

## 2.11. Excitation spectra

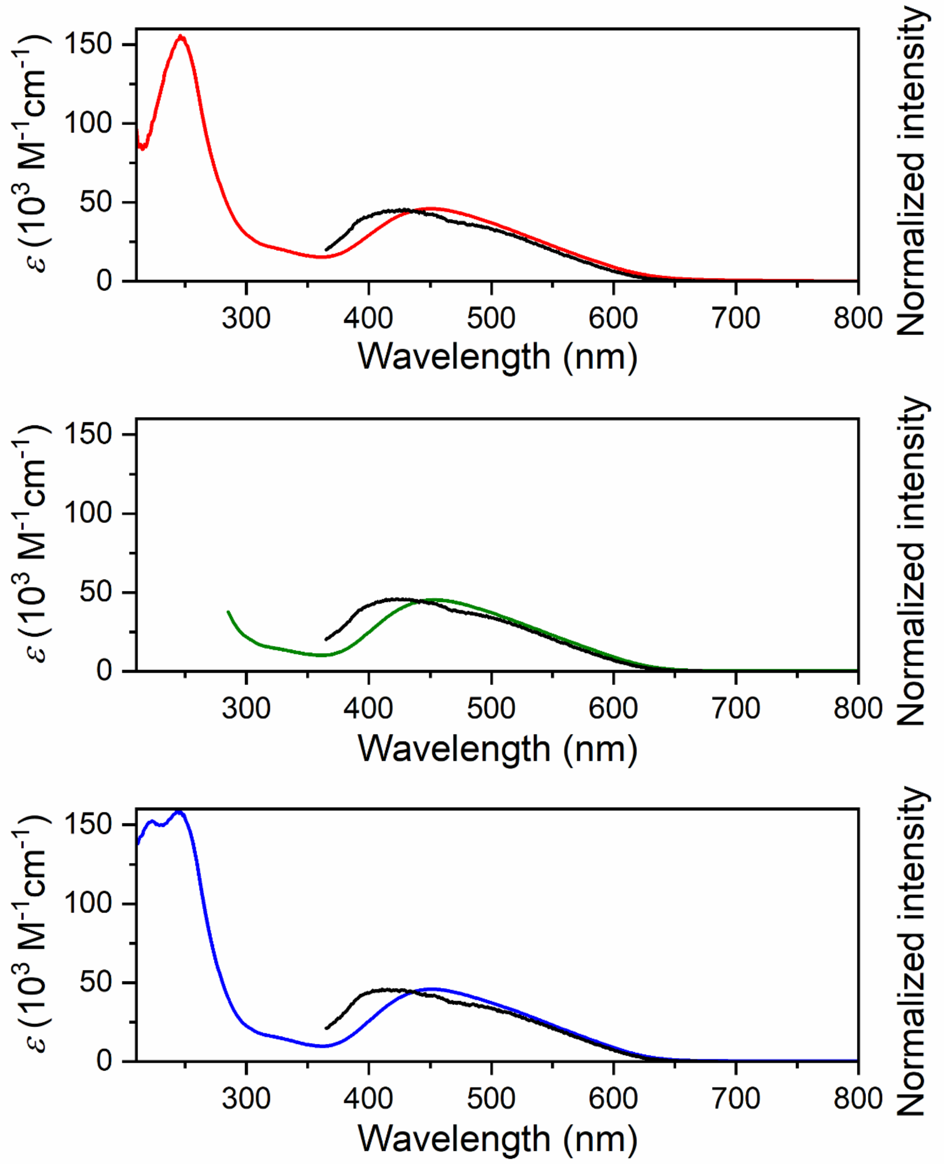


**Supplementary Fig. 37.** Excitation spectra (black, $\lambda_{det}$ = 700 nm, for all three cases) and UV–Vis absorption spectra in deaerated THF (red), toluene (green) and cyclohexane (blue), respectively, of [Cr($L^{Mes}$)$_3$] at 20 °C.

## 2.12. Transient absorption spectra and excited state decays (absorption and emission)

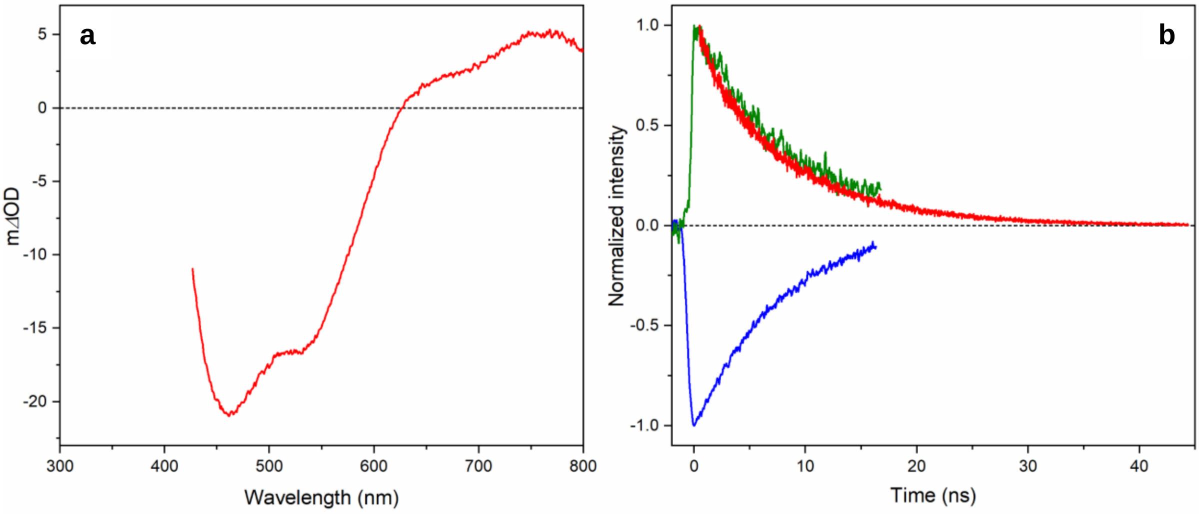


**Supplementary Fig. 38.** (**a**) Picosecond transient absorption spectrum of [Cr(L$^{Mes}$)$_3$] (20 μM) in deaerated THF solution at 20 °C, integrated over 2 ns following excitation at 530 nm with 30 ps laser pulses. (**b**) Kinetic decays obtained from the transient absorption spectrum of [Cr(L$^{Mes}$)$_3$] in deaerated THF at 750 (excited state absorption, green line) and 450 nm (MLCT bleach, blue line), respectively, with lifetime ($\tau$) of 7.8 ns. Luminescence decay of [Cr(L$^{Mes}$)$_3$] in THF at 750 nm (red line), $\lambda_{exc}$ = 473 nm, τ = 7.8 ns. The decays of the transient absorption signals of [Cr(L$^{Mes}$)$_3$] (blue and green traces in panel b) were measured on a transient absorption setup, which only permitted data collection over a time window of 20 ns.

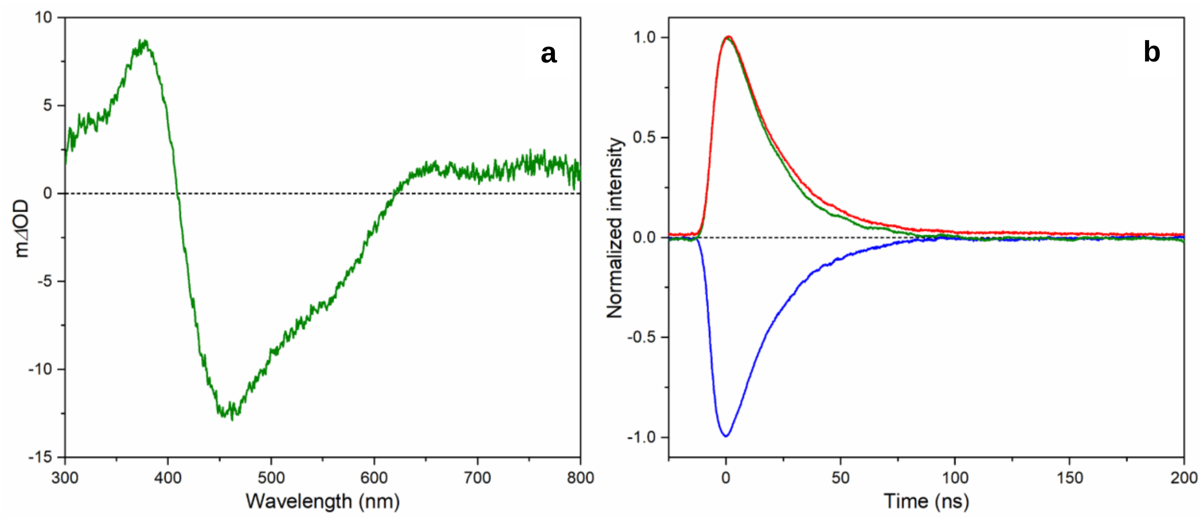


**Supplementary Fig. 39.** (**a**) Transient absorption spectrum of [Cr(L$^{Mes}$)$_3$] (20 μM) in deaerated toluene solution at 20 °C following excitation at 500 nm with laser pulses of ~10 ns. The signals were time-integrated over 200 ns immediately after excitation. (**b**) Kinetic decays obtained from the transient absorption spectrum of [Cr(L$^{Mes}$)$_3$] in deaerated toluene at 375 (excited state absorption, green line) and 460 nm (MLCT bleach, blue line), respectively, with lifetime ($\tau$) of 20.0 ns. Luminescence decay of [Cr(L$^{Mes}$)$_3$] in deaerated toluene at 715 nm (red line), τ = 20.0 ns.

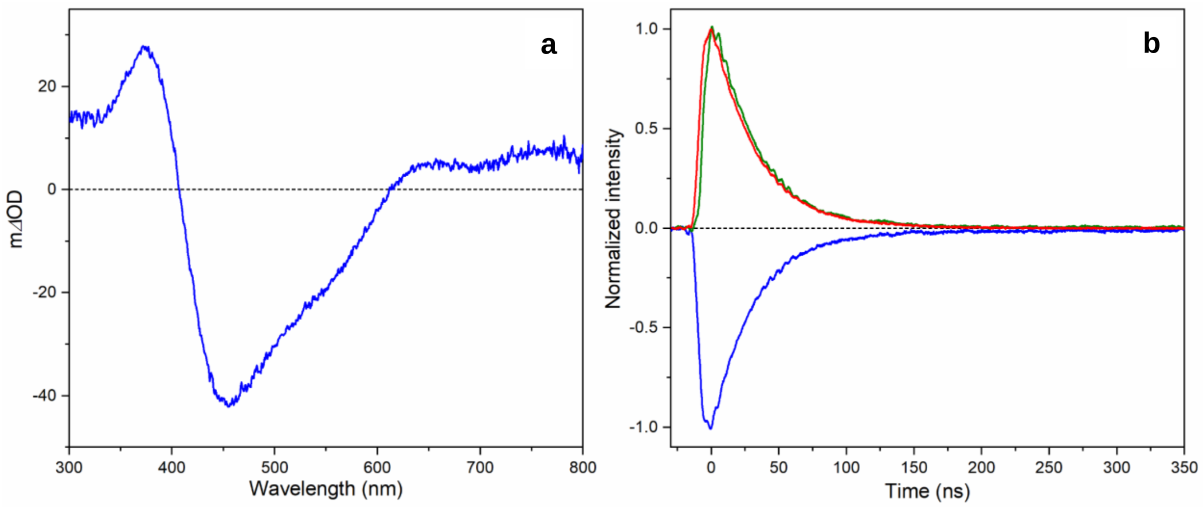


**Supplementary Fig. 40.** (**a**) Transient absorption spectrum of [Cr($L^{Mes}$)$_3$] (20 μM) in deaerated cyclohexane solution at 20 °C following excitation at 500 nm with laser pulses of ~10 ns. The signals were time-integrated over 200 ns immediately after excitation. (**b**) Kinetic decays obtained from the transient absorption spectrum of [Cr($L^{Mes}$)$_3$] in deaerated cyclohexane at 375 (excited state absorption, green line) and 460 nm (MLCT bleach, blue line), respectively, with lifetime ($\tau$) of 31.0 ns. Luminescence decay of [Cr($L^{Mes}$)$_3$] in deaerated toluene at 695 nm (red line), τ = 31.0 ns.

## 2.13. Luminescence quantum yield determination

**Supplementary Table 2. Raw data for quantum yield determination: [Cr(L$^{Mes}$)$_3$] in deaerated cyclohexane solution**

| UV-Vis absorption spectra | Emission spectra | Linear correlation |
|---|---|---|
| 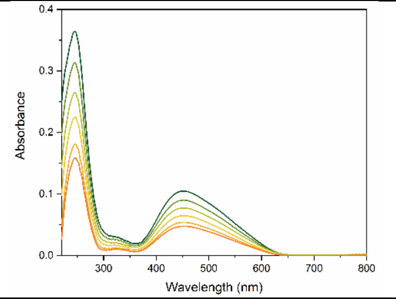 | 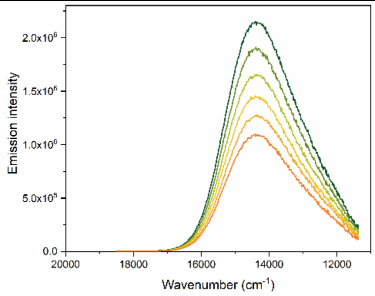 | 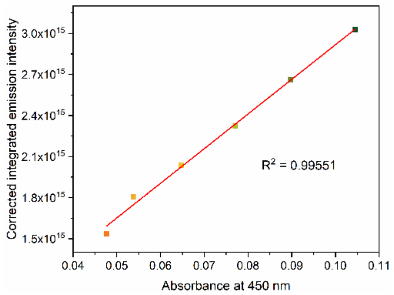 |

**Supplementary Table 3. Raw data for quantum yield determination: [Cr(L$^{Mes}$)$_3$] in deaerated toluene solution**

| UV-Vis absorption spectra | Emission spectra | Linear correlation |
|---|---|---|
| 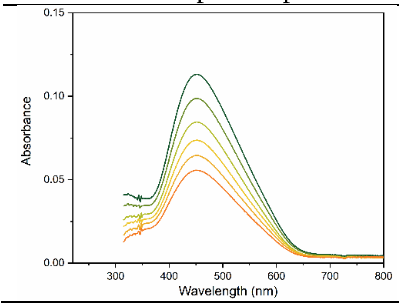 | 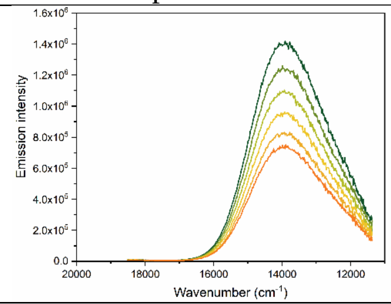 | 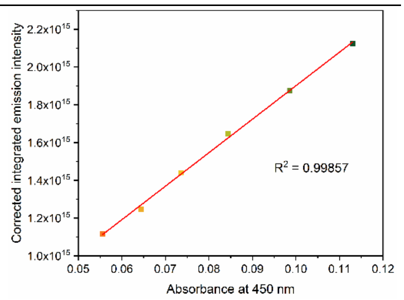 |

**Supplementary Table 4. Raw data for quantum yield determination: [Cr(L$^{Mes}$)$_3$] in deaerated tetrahydrofuran solution**

| UV-Vis Absorption Spectra | Emission Spectra | Linear correlation |
|---|---|---|
| 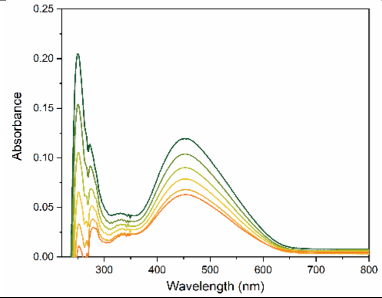 | 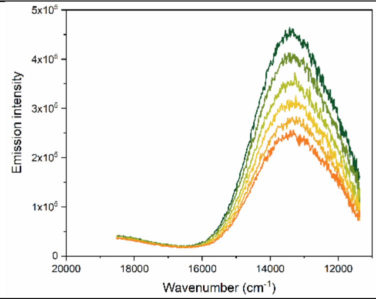 | 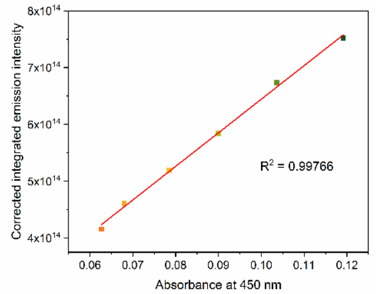 |

**Supplementary Table 5. Raw data for quantum yield determination: $[Ru(bpy)_3]^{2+}$ in aerated acetonitrile solution**

| UV-Vis absorption Spectra | Emission spectra | Linear correlation |
|---|---|---|
| 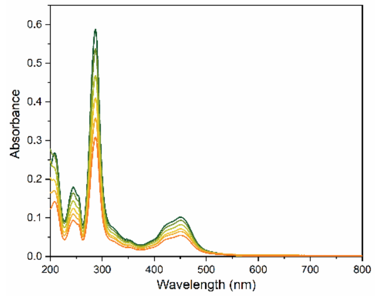 | 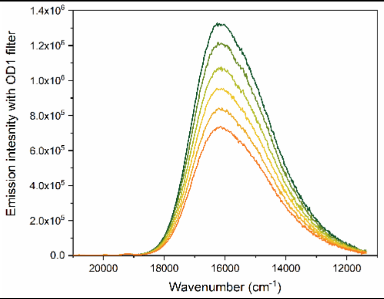 | 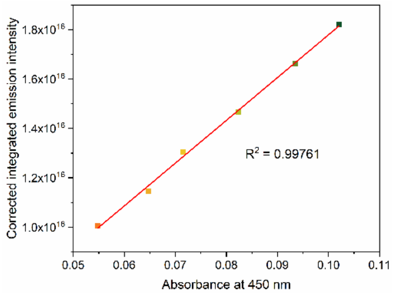 |



**Supplementary Table 6**. Key photophysical data for $[Cr(L^{Mes})_3]$ in solution at 20 °C

| | solvent | $\lambda_{abs}$ (MLCT) (nm) | $\lambda_{em, max}$ (MLCT) (nm) | $\tau$ (ns) | $\phi$ (%) |
|---|---|---|---|---|---|
| $[Cr(L^{Mes})_3]$ | cyclohexane | 350-650 | 695 | 31 | 0.36 ± 0.017 |
| | toluene | 350-650 | 715 | 20 | 0.24 ± 0.003 |
| | tetrahydrofuran | 350-650 | 745 | 7.8 | 0.072 ± 0.001 |

## 2.14. $^3$MLCT luminescence decay at different temperatures

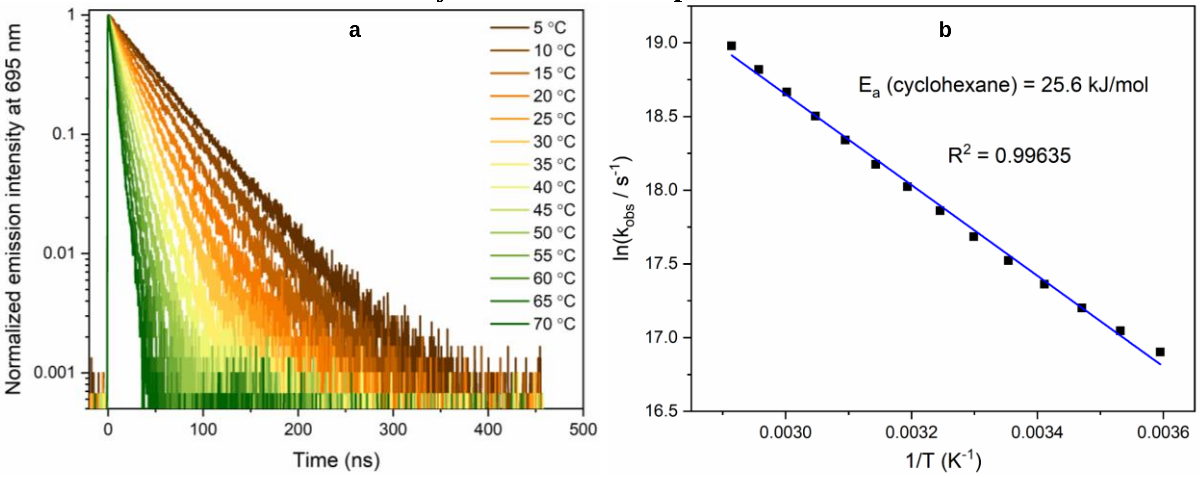


**Supplementary Fig. 41.** (**a**) Temperature-dependent luminescence decay of 20 µM [Cr(L$^{Mes}$)$_3$] in deaerated cyclohexane solution, $\lambda_{exc}$ = 473 nm; detection was at 695 nm. (**b**) Arrhenius analysis of the datasets in **(a)**.

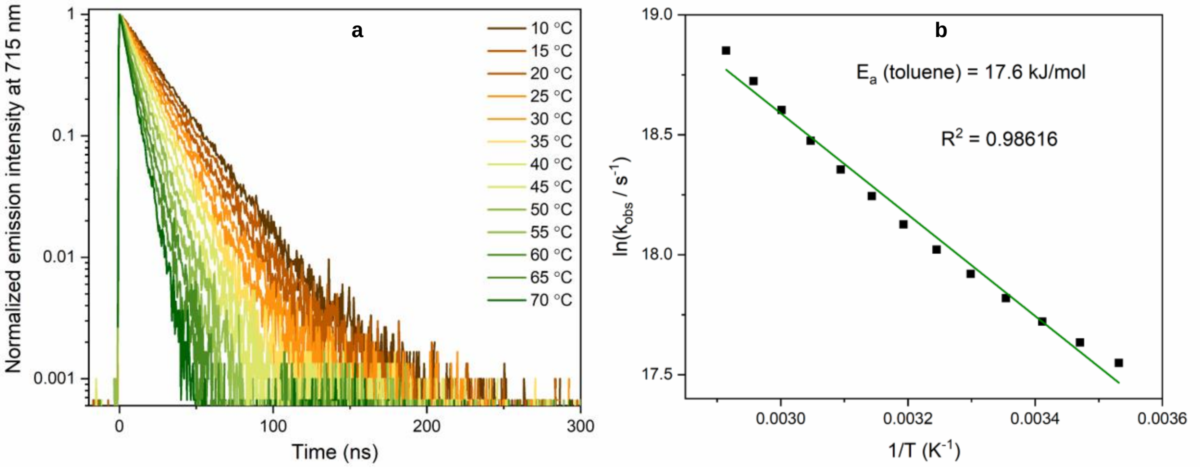


**Supplementary Fig. 42.** (**a**) Temperature-dependent luminescence decay of 20 µM [Cr(L$^{Mes}$)$_3$] in deaerated toluene solution, $\lambda_{exc}$ = 473 nm; detection was at 715 nm. (**b**) Arrhenius analysis of the datasets in **(a)**.

## 2.15. Spectro-electrochemical experiment

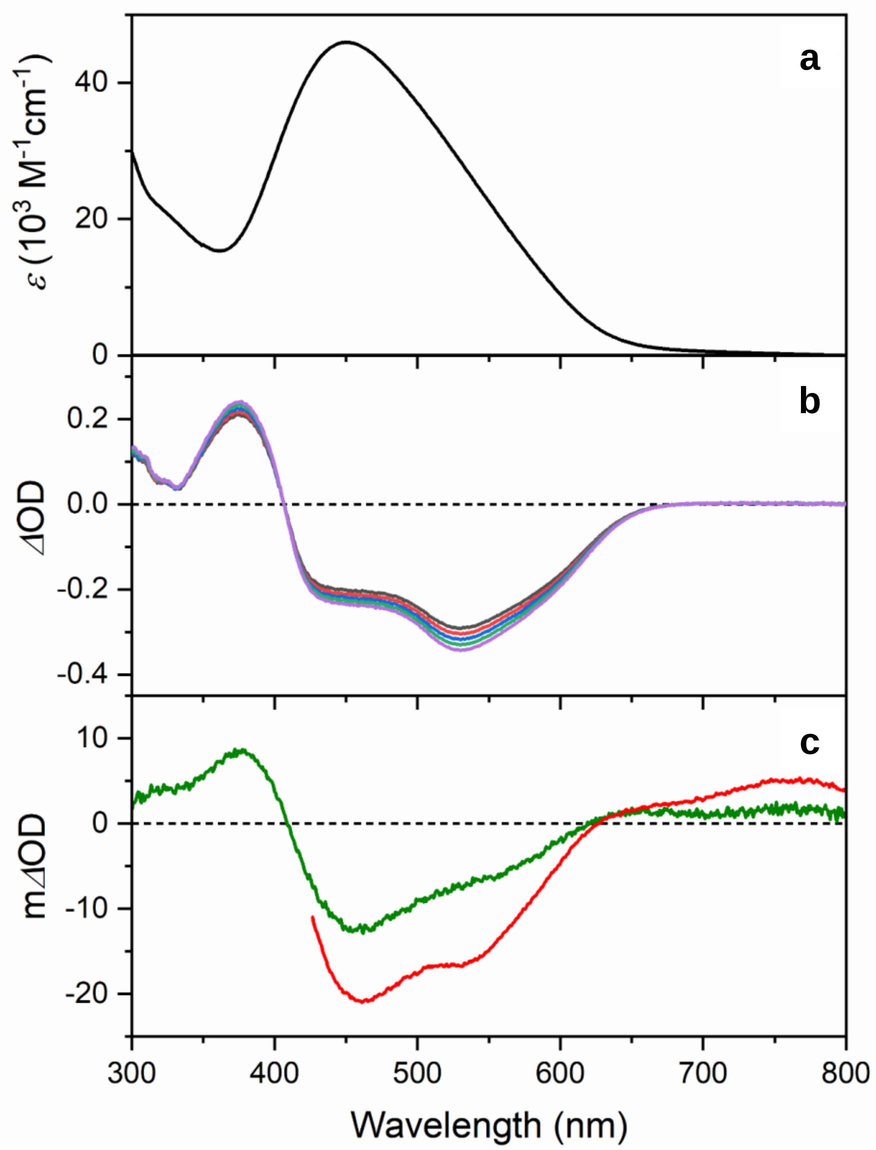


**Supplementary Fig. 43.** (**a**) UV–Vis absorption spectrum of [Cr($L^{Mes}$)$_3$] in THF. (**b**) UV−vis absorption changes following metal-based oxidation of [Cr($L^{Mes}$)$_3$] at -0.70 V versus $Fc^{+/0}$ [= $E_{1/2}$($Cr^{I/0}$)] in THF at 22 °C. The electrolyte was 0.1 M ($^{n}Bu_4N$)($PF_6$) in the spectro-electrochemical experiment. (**c**) Transient absorption spectra of [Cr($L^{Mes}$)$_3$] in dry and deaerated THF (red) and toluene (green), respectively.

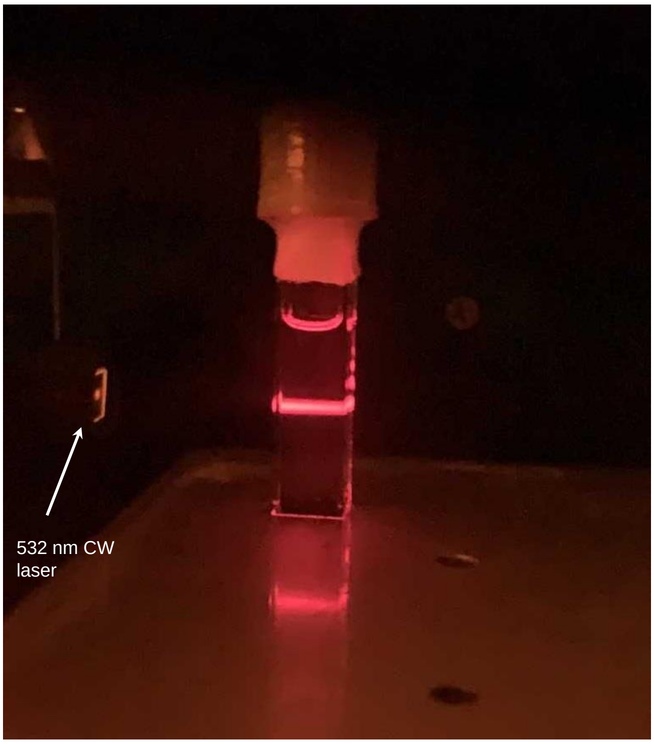


**Supplementary Fig. 44.** Image showing the red emission from a dry and deaerated toluene solution (20 μM) of [Cr($L^{Mes}$)$_3$] upon excitation with a 25 mW 532 nm CW laser. The image was taken using a green light filter in front of the camera.

## 3. Synthesis, characterization and photophysical details of [Cr(L$^{Pyr}$)$_3$]
### 3.1. Synthesis and characterization of chelating diisocyanide ligand L$^{Pyr}$

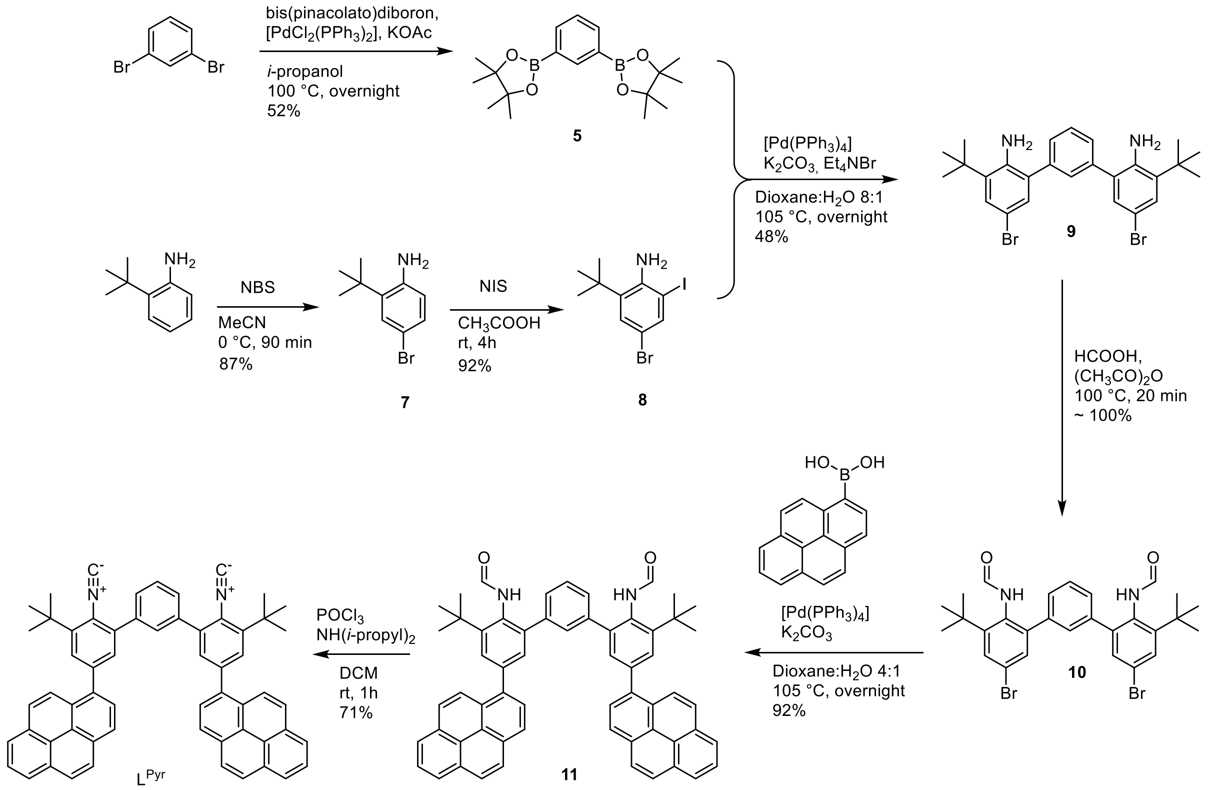


**Supplementary Fig. 45.** Synthesis of chelating bidentate arylisocyanide ligand, L$^{Pyr}$.

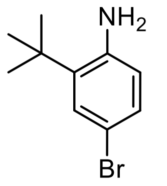

**Synthesis of compound 7**. *N*-Bromosuccinimide (NBS, 7.61 g, 42.8 mmol, 1.1 equiv.) was added to a solution of 2-*tert*-butyl-phenylamine (6 mL, 38.9 mmol, 1.0 equiv.) in dry acetonitrile (30 mL) at 0 °C, and the reaction mixture was stirred for 90 min. The reaction mixture was poured into $H_2O$ (50 mL) and extracted with dichloromethane (3 × 50 mL). The combined organic phases were washed with saturated aqueous solutions of $Na_2S_2O_5$ (50 mL) and $Na_2CO_3$ (50 mL), then dried over anhydrous $Na_2SO_4$, filtered and concentrated under reduced pressure. The product was purified with column chromatography ($SiO_2$, pentane:ethyl acetate 40:1 → 20:1) and concentrated to give compound **7** as a brown oil (7.70 g, 33.8 mmol, 87%). NMR data was in agreement with previous literature[4]. $^1$H NMR (400 MHz, $CDCl_3$): $\delta$ 7.32 (d, $J$ = 2.4 Hz, 1H), 7.13 (dd, $J$ = 8.4, 2.3 Hz, 1H), 6.58 (d, $J$ = 8.4 Hz, 1H), 4.42 (br s, 2H) 1.40 (s, 9H) ppm.

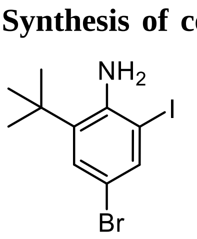

**Synthesis of compound 8**. Compound **7** (4.49 g, 19.7 mmol, 1.0 equiv.) was dissolved in glacial acetic acid (25 mL) and the solution was purged with $N_2$ for 20 min. *N*-Iodosuccinimide (NIS, 4.43 g, 19.7 mmol, 1.0 equiv.) was added, and the reaction mixture was stirred for 4 h at room temperature. The reaction mixture was poured into $H_2O$ (50 mL) and extracted with ethyl acetate (3 × 50 mL). The combined organic phases were washed with a saturated aqueous solution of $Na_2S_2O_5$ (50 mL) and brine (50 mL), then dried over anhydrous $Na_2SO_4$, filtered and concentrated under reduced pressure to give compound **8** as a black oil (6.39 g, 18.1 mmol, 92%). $^1$H NMR (400 MHz, $CDCl_3$): $\delta$ 7.69 (d, $J$ = 2.3 Hz, 1H), 7.30 (d, $J$ = 2.3 Hz, 1H), 4.35 (s, 2H), 1.39 (s, 9H) ppm. $^{13}$C{$^1$H} NMR (101 MHz, $CDCl_3$): $\delta$ 143.5, 138.8, 135.6,

130.1, 110.3, 88.4, 35.3, 29.4 ppm. $R_f$ (petroleum ether:ethyl acetate 20:1) = 0.5. HRMS (ESI, positive ions): *m/z* 353.9353 (calculated for [**8** + H]$^+$ 353.9349).

**Synthesis of compound 9**. A mixture of compound **5** (1.42 g, 4.30 mmol, 1.0 equiv.), compound **8** (3.35 g, 9.47 mmol, 2.2 equiv.), $Et_4NBr$ (139 mg, 0.43 mmol, 0.1 equiv.) and $K_2CO_3$ (2.97 g, 21.5 mmol, 5.0 equiv.) in 1,4-dioxane:$H_2O$ (6:1, 35 mL) was purged for 20 min with $N_2$. [Pd($PPh_3$)$_4$] (497 mg, 0.43 mmol, 0.1 equiv.) was added, and the purging with $N_2$ was continued for additional 10 minutes. Subsequently, the reaction mixture was refluxed under $N_2$ overnight. After cooling to room temperature, the reaction mixture was extracted with dichloromethane (3 × 50 mL). The combined organic phases were dried over anhydrous $Na_2SO_4$, filtered, and concentrated to dryness. The product was purified by column chromatography ($SiO_2$, pentane:dichloromethane 8:1 to 2:1) and concentrated to give compound **9** as a pale brown solid (1.10 g, 2.07 mmol, 48 %). $^1$H NMR (400 MHz, $CDCl_3$): *δ* 7.52 (t, *J* = 8.2 Hz, 1H), 7.45 – 7.39 (m, 3H), 7.34 (d, *J* = 2.4 Hz, 2H), 7.14 (d, *J* = 2.3 Hz, 2H), 3.97 (s, 4H), 1.44 (s, 18H) ppm. $^{13}$C{$^1$H} NMR (101 MHz, $CDCl_3$): *δ* 141.0, 139.8, 135.9, 132.5, 130.8, 130.6, 129.7, 129.1, 128.9, 110.3, 34.8, 29.7 ppm. $R_f$ (dichloromethane:pentane 1:1) = 0.6. Anal. Calcd. for **9** ($C_{26}H_{30}Br_2N_2$) (%): C, 58.86; H, 5.67; N, 5.09 (observed); C, 58.88; H, 5.70; N, 5.28 (calculated). HRMS (ESI, positive ions): *m/z* 531.0823 (calculated for [**9** + H]$^+$ 531.0828). FTIR: ν 3506 (w, $\nu_{N\text{-}H}$), 3400 (w, $\nu_{N\text{-}H}$), 2965 (m), 2913 (w), 2874 (w), 1751 (w), 1690 (w), 1610 (s, $\nu_{N\text{-}H}$), 1568 (m), 1482 (m), 1464 (m), 1437 (s), 1426 (s), 1412 (s), 1392 (s), 1369 (m), 1307 (w), 1256 (m), 1245 (s), 1195 (w), 1091 (m), 1043 (w), 1020 (w), 906 (w), 869 (s), 806 (m), 767 (w), 717 (s), 693 (m), 661 (w),640 (m), 630 (m), 614 (m), 587 (w), 550 (m), 517 (m), 455 (w) cm$^{-1}$.

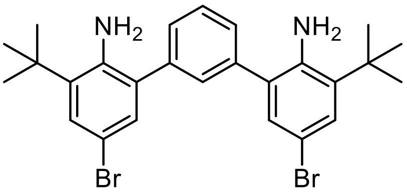


**Synthesis of compound 10**. Formic acid (3.5 mL, 83.1 mmol) was added dropwise to acetic anhydride (8 mL, 95.1 mmol) at 0°C. The mixture was allowed to warm to room temperature, whereupon it was heated to 50 °C for 2h. Compound **9** (1.05 g, 1.98 mmol) was added after cooling the reaction mixture back to 0 °C, and the reaction mixture was stirred at 100 °C for 20 min. The reaction mixture was poured over ice-cold water (20 mL), whereupon a solid precipitated. The solid was isolated by vacuum filtration, washed with ice-cold water, and dried under vacuum to give compound **10** as a pale brown powder (1.16 g, 1.98 mmol, ~100 %). The product is a mixture of different (formamide) rotamers, which complicates the $^1$H NMR spectrum. $^1$H NMR (400 MHz, $CDCl_3$): *δ* 8.18-8.15 (m, 0.5H), 7.99-7.95 (m, 0.5H), 7.85-7.57 (m, 6H), 7.53-7.18 (m, 10H), 7.14-7.10 (m, 1H), 1.44-1.39 (m, 18H) ppm. $R_f$ (dichloromethane) = 0.2. HRMS (ESI, positive ions): *m/z* 609.0549 (calculated for [**10** + Na]$^+$). Anal. Calcd. for **10**·0.5($H_2O$) [($C_{28}H_{30}Br_2N_2O_2$)·0.5($H_2O$)] (%): C, 56.65; H, 5.23; N, 4.59 (observed); C, 56.49; H, 5.25, N, 4.71 (calculated). FTIR: ν 2965 (w), 2873 (w), 1674 (s, $\nu_{C=O}$), 1566 (m), 1482 (m), 1388 (m), 1363 (m), 1260 (m), 1102 (w), 1079 (w), 1069 (w), 1000 (w), 889 (w), 869 (s), 801 (m), 738 (m), 738 (m), 711 (m), 692 (m), 638 (w), 612 (w), 556 (w), 512 (w), 453 (w) cm$^{-1}$.

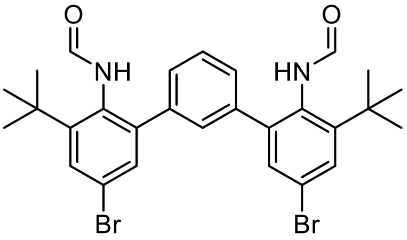

**Synthesis of compound 11**. A mixture of compound **10** (1.20 g, 2.05 mmol, 1.0 equiv.), pyrene-1-boronic acid (1.51 g, 6.14 mmol, 3.0 equiv.) and $K_2CO_3$ (2.28 g, 16.5 mmol, 8.0 equiv.) in 1,4-dioxane:$H_2O$ (4:1, 60 mL) was purged for 30 min with $N_2$. [Pd($PPh_3$)$_4$] (477 mg, 0.412 mmol, 0.2 equiv.) was added, and the purge with $N_2$ was continued for additional 10 minutes. Subsequently, the reaction mixture was refluxed under $N_2$ overnight. Brine (50 mL) was added to the reaction mixture after cooling it to room temperature, and the aqueous phase was extracted with ethyl acetate (3 × 50 mL). The combined organic phases were dried over anhydrous $Na_2SO_4$, filtered, and concentrated to dryness. The product was purified by column chromatography ($SiO_2$, petroleum ether:ethyl acetate 2:1 → 1:2) and concentrated to give **11** as a pale brown solid (1.56 g, 1.88 mmol, 92 %). The product is a mixture of different (formamide) rotamers, which complicates the $^1$H NMR spectrum. $^1$H NMR (400 MHz, $CDCl_3$): $\delta$ 8.31-7.98 (m, 18H), 7.93 (d, *J* = 11.8 Hz, 0.5H), 7.83 (d, *J* = 2.0 Hz, 0.5H), 7.80 (d, *J* = 2.1 Hz, 0.5H), 7.78 (d, *J* = 2.0 Hz, 0.5H), 7.76 (d, *J* = 2.0 Hz, 0.5H), 7.70 (d, *J* = 2.0 Hz, 0.5H), 7.66 (d, *J* = 2.0 Hz, 0.5H), 7.60 (t, *J* = 1.8 Hz, 0.5H), 7.55 (d, *J* = 1.9 Hz, 0.5H), 7.52 (d, *J* = 2.0 Hz, 0.5H), 7.50 – 7.40 (m, 5H), 1.58 – 1.53 (m, 18H) ppm. $R_f$ (ethyl acetate) = 0.6. HRMS (ESI, positive ions): *m/z* 851.3596 (calculated for [**11** + Na]$^+$ 851.3608). Anal. Calcd. for **11**·1.5($C_4H_8O_2$) [($C_{60}H_{44}N_2O_2$)·1.5($C_4H_8O_2$): C, 82.54; H, 6.30; N, 2.75 (observed); C, 82.47; H, 6.29; N, 2.91 (calculated). FTIR: ν 3039 (w), 2959 (w), 2867 (w), 1731 (w), 1675 (s, $\nu_{C=O}$), 1589 (w), 1486 (m), 1456 (m), 1432 (w), 1385 (w), 1362 (m), 1240 (m), 1187 (w), 1042 (w), 879 (w), 844 (s), 819 (w), 797 (w), 757 (w), 741 (w), 723 (m), 694 (m), 495 (w) cm$^{-1}$.

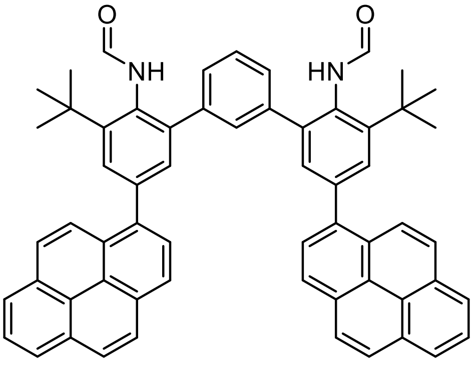


**Synthesis of $L^{Pyr}$**. Diisopropylamine (2.1 mL, 15.0 mmol, 8.0 equiv.) was added to a solution of compound **11** (1.56 g, 1.87 mmol, 1.0 equiv.) in dry dichloromethane (50 mL) under $N_2$. The mixture was cooled to 0 °C, and $POCl_3$ (1.1 mL, 11.3 mmol, 6.0 equiv.) was added dropwise. The cooling bath was removed after complete addition of $POCl_3$, and the reaction mixture was stirred for 30 min at rt. A saturated aqueous $Na_2CO_3$ solution was added dropwise (50 mL) to the resulting yellow solution, and the aqueous phase was extracted with dichloromethane (3 × 50 mL). The combined organic phases were dried over anhydrous $Na_2SO_4$, filtered, and concentrated to dryness. The crude was purified by column chromatography ($SiO_2$, petroleum ether:ethyl acetate 20:1) to give $L^{Pyr}$ as an pale brown solid (1.06 g, 1.34 mmol, 71%). $^1$H NMR (500 MHz, 298 K, $C_6D_6$): $\delta$ 8.27 (d, *J* = 9.2 Hz, 2H), 7.96 (dd, *J* = 7.6, 1.1 Hz, 2H), 7.95 (d, *J* = 7.8 Hz, 2H), 7.89 (dd, *J* = 7.7, 1.1 Hz, 2H), 7.87 (d, *J* = 0.6 Hz, 2 x 2H), 7.84 (d, J = 7.8 Hz, 2H), 7.81 (t, J = 1.8 Hz, 1H), 7.80 – 7.75 (m, 4H), (2H), 7.74 (d, *J* = 1.9 Hz, 2H), 7.64 (d, *J* = 1.9 Hz, 2H), 7.61 (dd, *J* = 7.8, 1.8 Hz, 2H), 7.37 (t, *J* = 7.8 Hz, 1H), 1.51 (s, 18H) ppm. $^{13}$C{$^1$H} NMR (126 MHz, $C_6D_6$, 298K) δ 176.8, 146.7, 142.5, 142.3, 139.0, 136.5, 132.0, 131.6, 131.4, 131.1, 131.0, 129.7, 129.02, 128.97, 128.9, 128.5, 128.4, 128.0, 127.7, 126.5, 125.8, 125.7, 125.53, 125.47, 125.2, 124.9, 123.3, 35.7, 29.5 ppm. $R_f$ (petroleum ether:ethyl acetate 5:1) = 0.6. HRMS (ESI, positive ions): *m/z* 815.3410 (calculated for [$L^{Pyr}$ + Na]$^+$ 815.3397). Anal. Calcd. for $L^{Pyr}$·3($H_2O$) [($C_{60}H_{44}N_2$)·3($H_2O$)] (%): C, 84.88; H, 6.04; N, 3.13 (observed); C, 85.06; H, 5.95; N, 3.31 (calculated). FTIR: ν 3041 (w), 2964 (m), 2874 (w), 2112 (s, $\nu_{C≡N}$), 1594 (m), 1482 (w), 1420

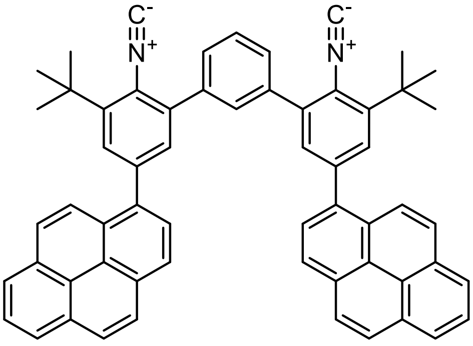

(w), 1396 (w), 1370 (m), 1273 (w), 1242 (w), 1189 (w), 896 (m), 845 (s), 817 (m), 802 (m), 759 (m), 723 (s), 710 (m), 683 (m) $cm^{-1}$.

**3.2. Synthesis of** [Cr($L^{Pyr}$)$_3$].

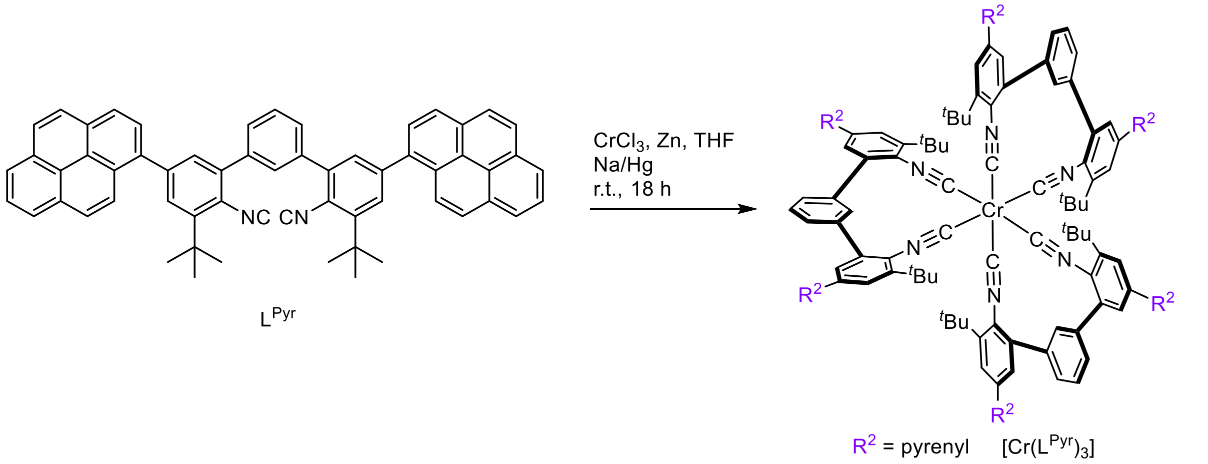


[Cr($L^{Pyr}$)$_3$] was synthesized according to the method previously published for $Cr^0$ tris(aryldiisocyanide) complexes, where [$CrCl_3$(THF)$_3$] was prepared *in situ* from $CrCl_3$ and then reacted with the bidentate isocyanide ligand[7,8]. Anhydrous $CrCl_3$ (4.4 mg, 0.03 mmol) was stirred in dry THF (1 mL) under argon at room temperature in the presence of Zn powder (1.2 mg). Over the course of 2 h, the solution changed color from purple to green. In a separate Schlenk flask, sodium amalgam was prepared by adding small pieces of Na (36 mg) to Hg (0.4 mL). A solution of $L^{Pyr}$ (66.1 mg, 0.083 mmol) in dry THF (2 mL) was then added to the sodium amalgam, followed by the freshly prepared solution of [$CrCl_3$(THF)$_3$], whereupon the color of the reaction mixture immediately turned dark purple. Additional dry THF (2 mL) was added to the reaction mixture, and it was stirred overnight at room temperature. The reaction mixture was transferred to another Schlenk flask with a syringe through a filter of celite under an inert atmosphere of argon, and all volatiles were removed from the reaction mixture under reduced pressure. At this stage, the crude typically contained 10-20% of free ligand. Pure samples of [Cr($L^{Pyr}$)$_3$] were obtained by slow evaporation of *n*-pentane into concentrated THF solutions of the crude under inert conditions (glovebox). The precipitate of [Cr($L^{Pyr}$)$_3$] was isolated by vacuum filtration under ambient (air) conditions. Remaining silicon grease was removed by transferring the filtered residue of [Cr($L^{Pyr}$)$_3$] to a Schlenk flask containing *n*-pentane under inert conditions and sonication for 5 min. Isolation by vacuum filtration under ambient (air) conditions afforded [Cr($L^{Pyr}$)$_3$] as a purple solid (32 mg, 0.013 mmol, 47 %). [Cr($L^{Pyr}$)$_3$] is sensitive to oxygen when in solution, hence the purification of [Cr($L^{Pyr}$)$_3$] by precipitation has to be carried out under strictly inert conditions to avoid paramagnetic impurities in the final sample.

$^1$H NMR (600 MHz, toluene-$d_8$, 378 K): $\delta$ 8.81 (s, 1H), 8.35 (d, $J$ = 9.3 Hz, 2H), 7.95 (d, $J$ = 2.0 Hz, 4H), 7.90 (dd, $J$ = 7.6, 1.1 Hz, 2H), 7.84 – 7.79 (m, 8H), 7.77 (d, $J$ = 9.3 Hz, 2H), 7.70 (t, $J$ = 7.6 Hz, 2H), 7.60 (d, $J$ = 2.0 Hz, 2H), 7.47 (dd, $J$ = 7.7, 1.8 Hz, 2H), 7.35 (t, $J$ = 7.7 Hz, 1H), 1.82 (s, 18H) ppm. $^{13}$C NMR (151 MHz, toluene-d8, 378 K): $\delta$ 199.2 (333K), 144.8, 140.5, 139.6, 137.7, 137.3, 131.7, 131.24, 131.22, 131.1, 130.8, 130.1, 129.6, 128.8, 128.7, 128.3, 127.5, 127.3, 127.23, 127.21, 125.6, 125.4, 125.2, 125.0, 124.9, 124.6, 124.5, 35.9, 31.0 ppm. HRMS (ESI, positive ions): *m/z* 1214.9949 (calculated for [Cr($L^{Pyr}$)$_3$]$^{2+}$ 1214.9971). Anal. Calcd. for [Cr($L^{Pyr}$)$_3$]·2($C_4H_8O$)·2($C_5H_{12}$) [($C_{198}H_{172}CrN_6O_2$)·2($C_4H_8O$)·2($C_5H_{12}$)] (%): C, 87.63; H, 6.79; N, 2.67 (observed); C, 87.40; H, 6.45; N, 3.07 (calculated). FTIR: ν 3039 (w), 2951 (w), 2921 (w), 2851 (w), 1945 (s, $\nu_{C\equiv N}$), 1586 (w), 1453 (w), 1420 (m), 1404 (m), 1361

(w), 1243 (w), 1066 (m), 891 (m), 842 (s), 818 (m), 808 (m), 796 (m), 755 (m), 723 (m), 712 (m), 692 (m), 683 (m), 659 (w), 580 (s), 504 (w), 454 (w), 438 (m) $cm^{-1}$.

### 3.3. NMR spectra

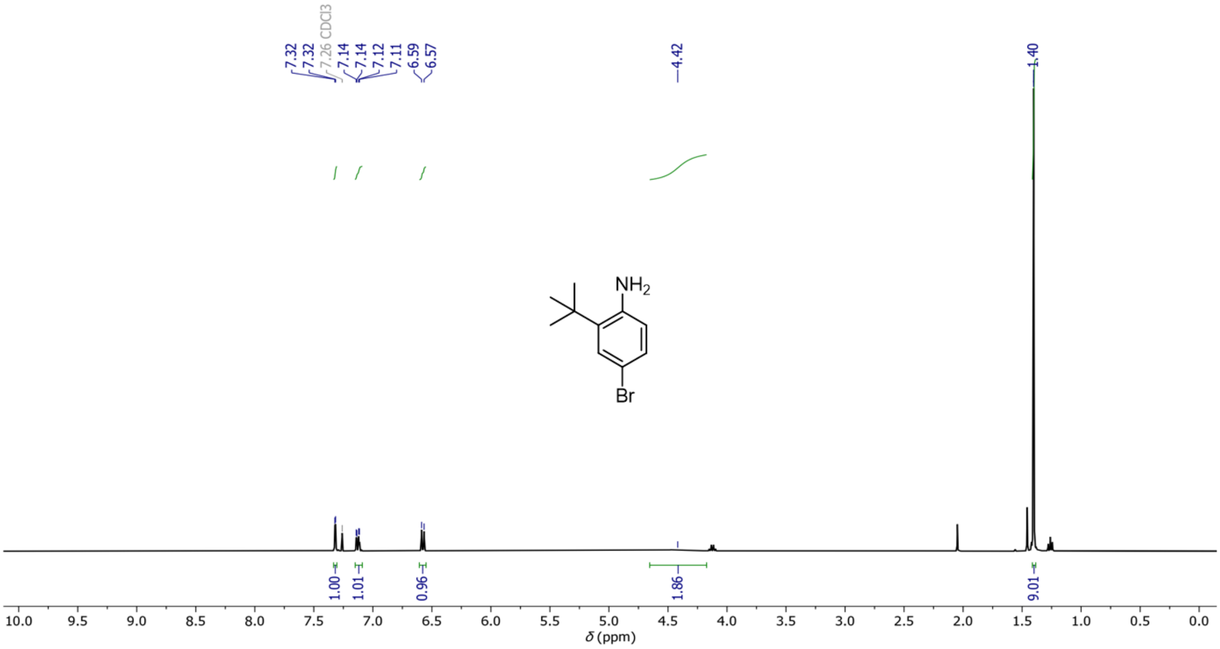


**Supplementary Fig. 46.** $^1$H NMR spectrum of compound **7** (400 MHz, $CDCl_3$).

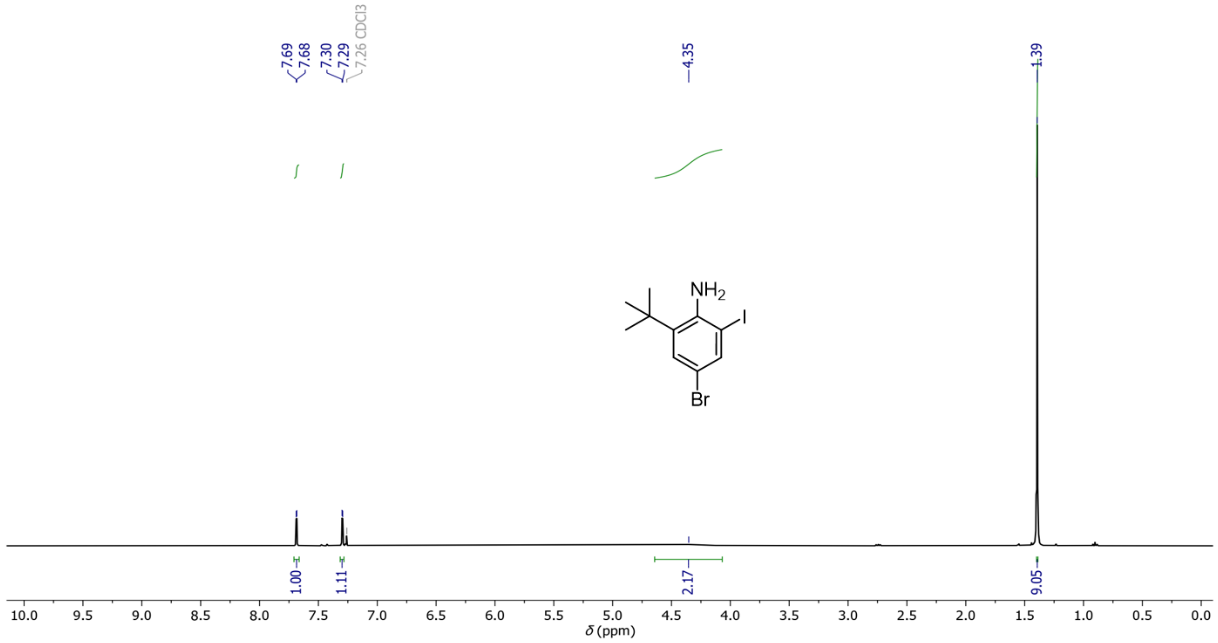


**Supplementary Fig. 47.** $^1$H NMR spectrum of compound **8** (400 MHz, $CDCl_3$).

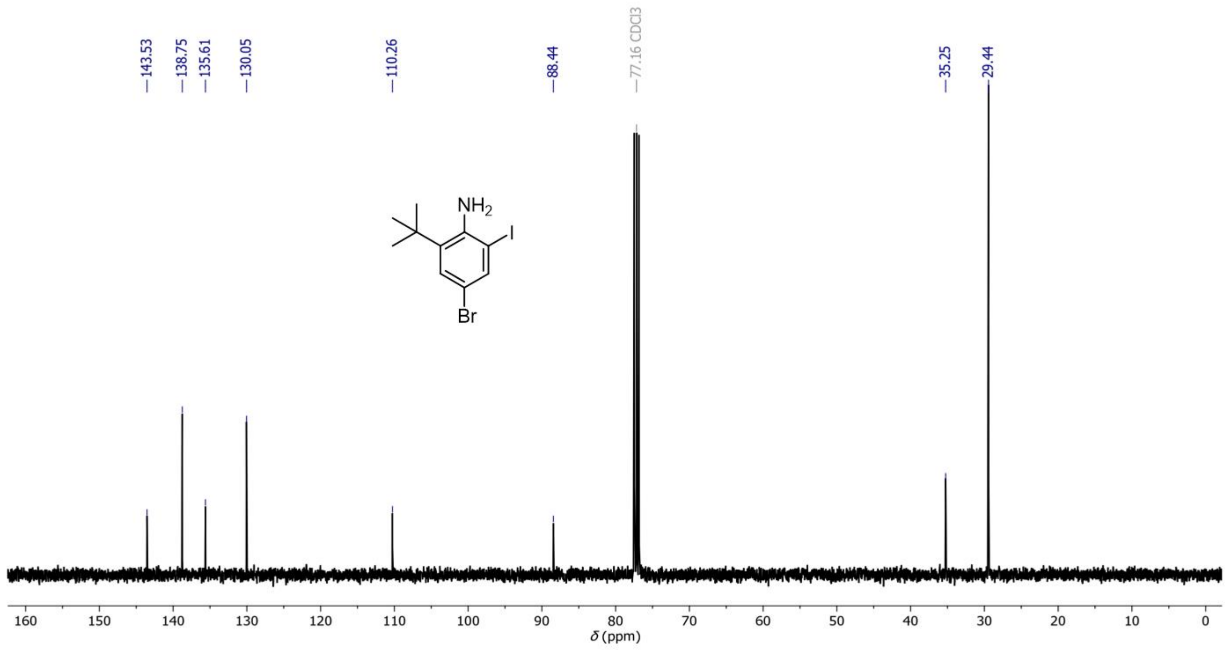


**Supplementary Fig. 48.** $^{13}C\{^{1}H\}$ NMR spectrum of compound **8** (101 MHz, $CDCl_3$).

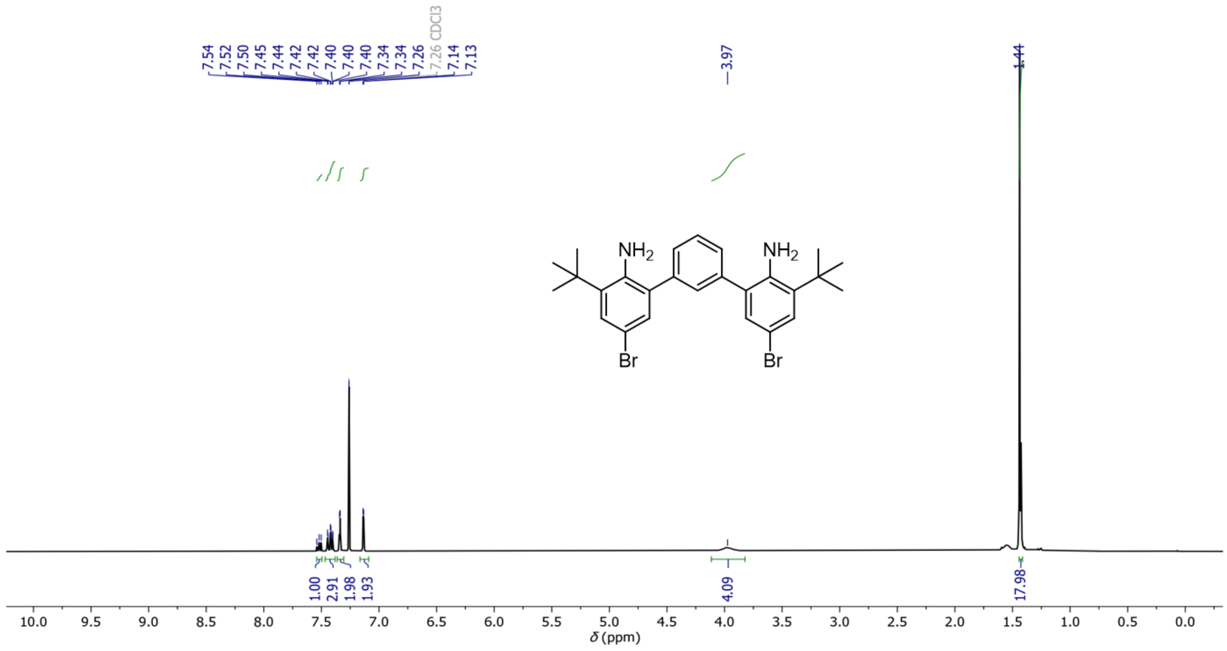


**Supplementary Fig. 49.** $^{1}H$ NMR spectrum of compound **9** (400 MHz, $CDCl_3$).

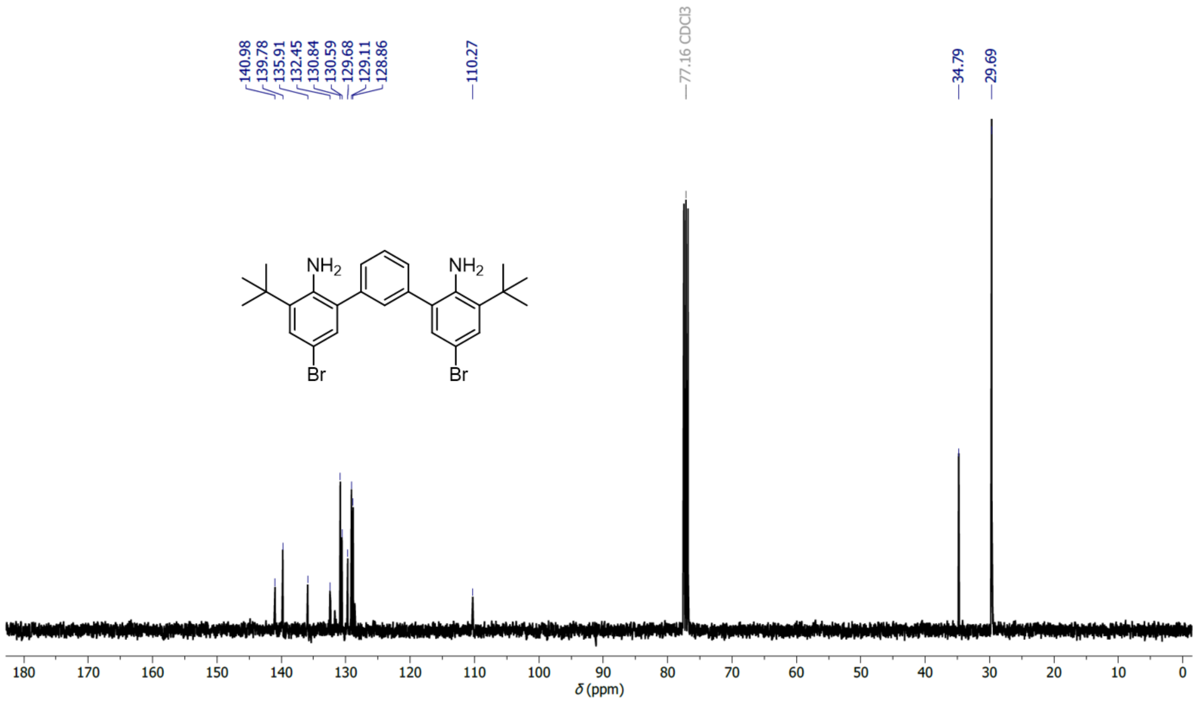

**Supplementary Fig. 50.** $^{13}C\{^{1}H\}$ NMR spectrum of compound **9** (101 MHz, $CDCl_3$).

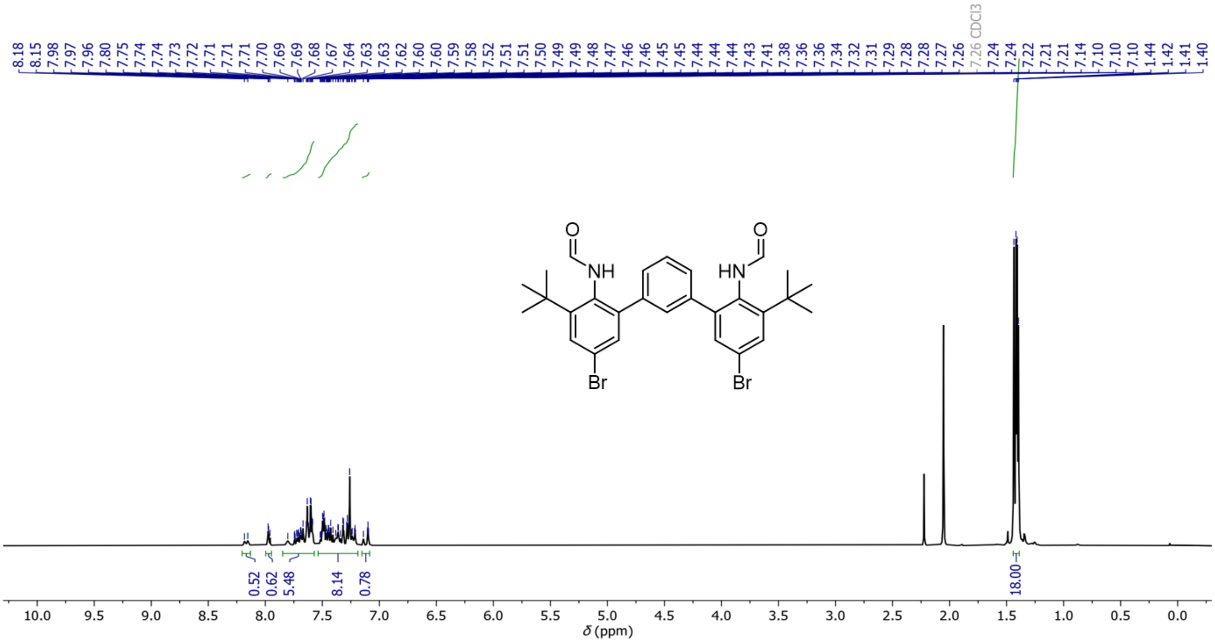

**Supplementary Fig. 51.** $^{1}H$ NMR spectrum of compound **10** (400 MHz, $CDCl_3$). The resonances appearing at 2.05 and 2.22 ppm are due to traces of acetic acid and acetone, respectively.

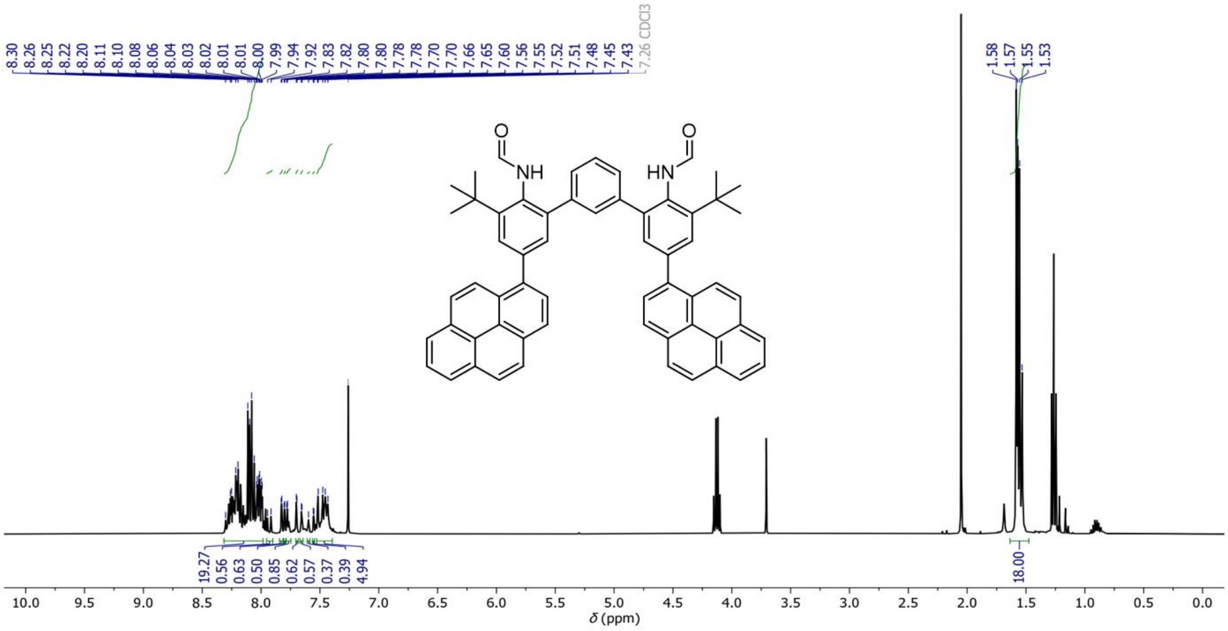


**Supplementary Fig. 52.** $^{1}H$ NMR spectrum of compound **11** (400 MHz, $CDCl_3$). The resonances appearing at 4.13, 2.05 and 1.26 ppm are due to traces of ethyl acetate and the resonance appearing at 3.71 ppm is due to a trace of 1,4-dioxane.

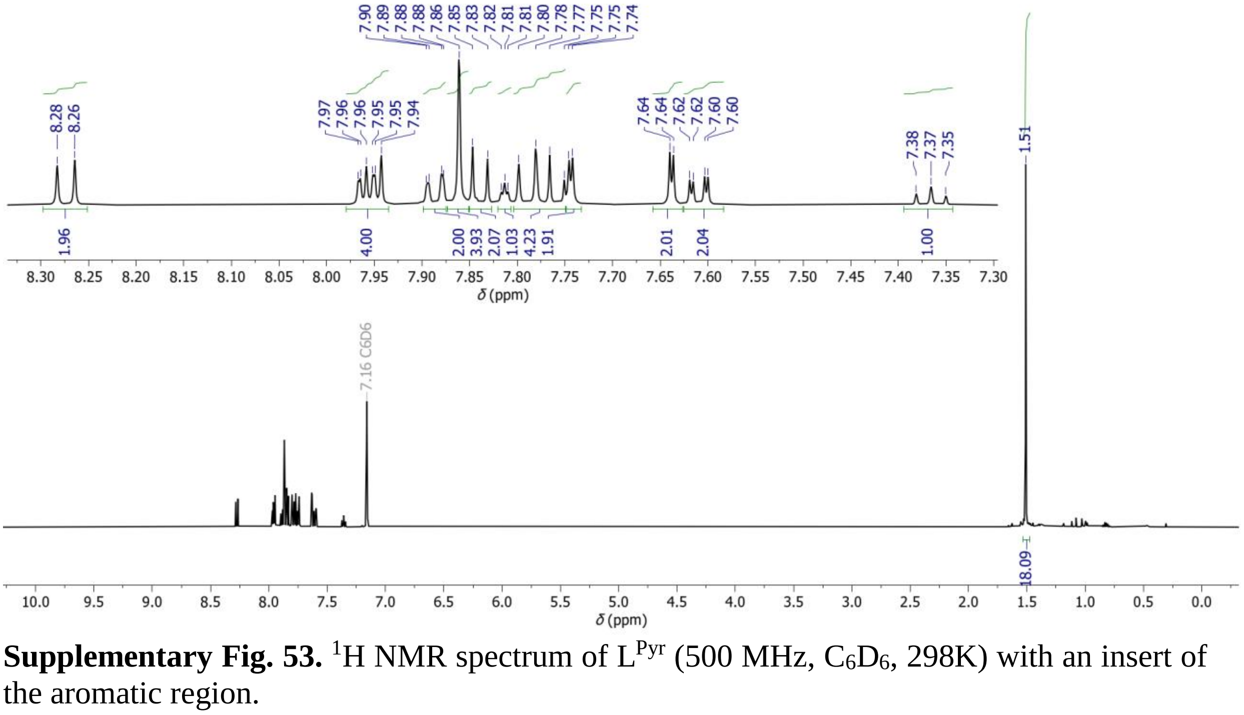


**Supplementary Fig. 53.** $^{1}H$ NMR spectrum of $L^{Pyr}$ (500 MHz, $C_6D_6$, 298K) with an insert of the aromatic region.

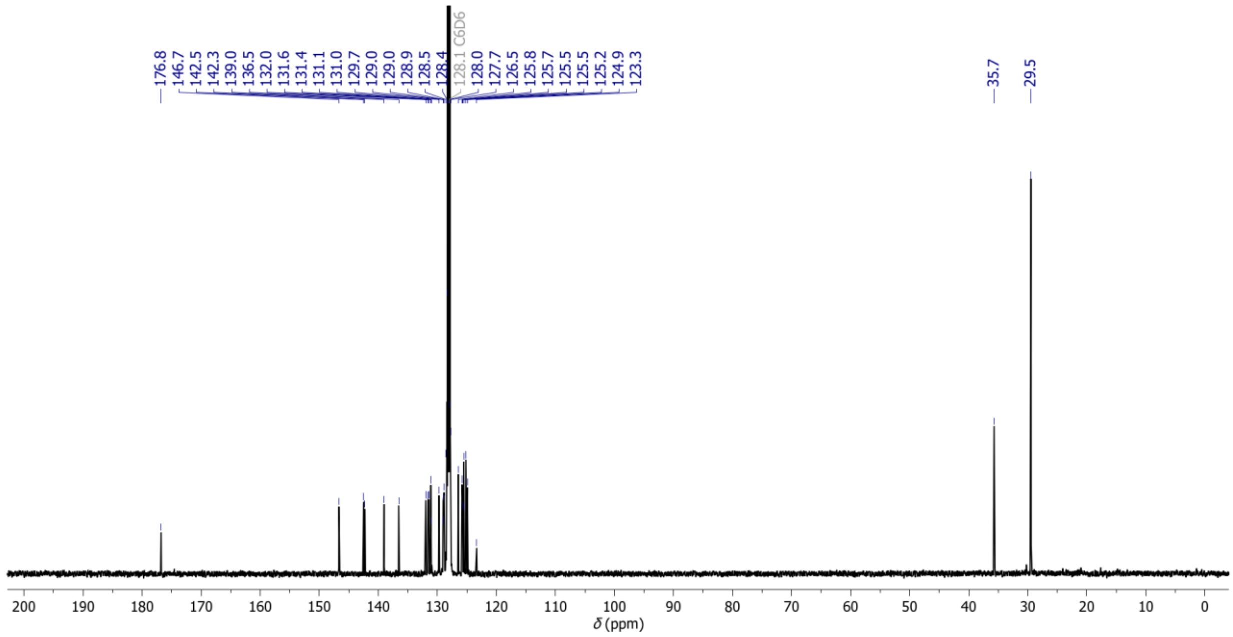


**Supplementary Fig. 54.** $^{13}C\{^{1}H\}$ NMR spectrum of $L^{Pyr}$ (126 MHz, $C_6D_6$, 298K). The resonance at 176.8 ppm originates from the isocyanide carbon.

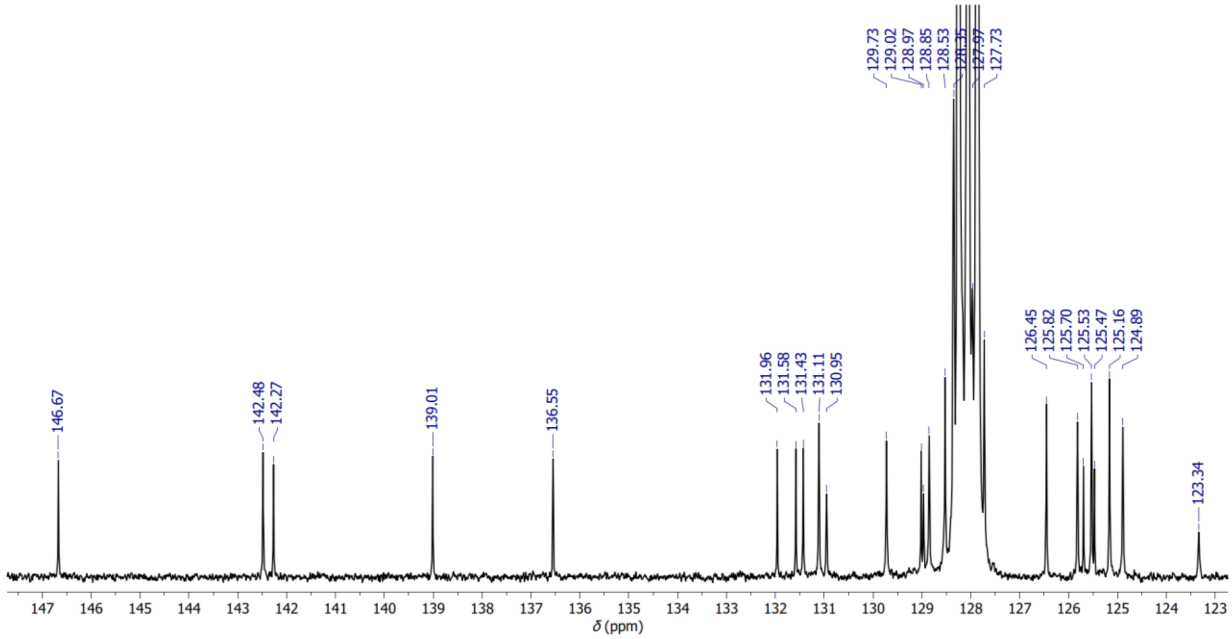


**Supplementary Fig. 55.** Zoom of the aromatic region between 123 and 148 ppm of the $^{13}C\{^{1}H\}$ NMR spectrum of $L^{Pyr}$ (126 MHz, $C_6D_6$, 298K).

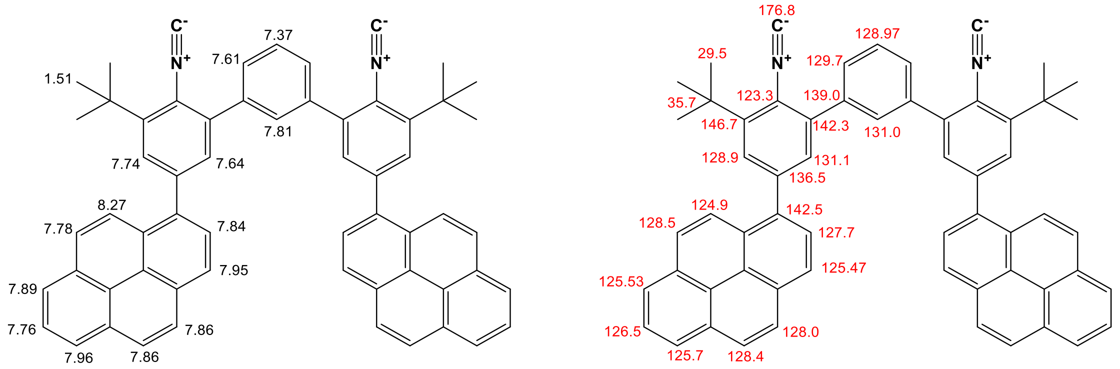


**Supplementary Fig. 56.** Assignment of the measured $^{1}H$-NMR (left) and $^{13}C$-NMR (right) resonances of $L^{Pyr}$ in $C_6D_6$ in Supplementary Fig. 53 and 54, respectively. The 2D-NMR techniques COSY (Supplementary Fig. 57) and NOESY (Supplementary Fig. 58) were used to assign the protons, and HSQC (Supplementary Fig. 60) and HMBC (Supplementary Fig. 62) were used to establish the coupling between $^{1}H$ and $^{13}C$.

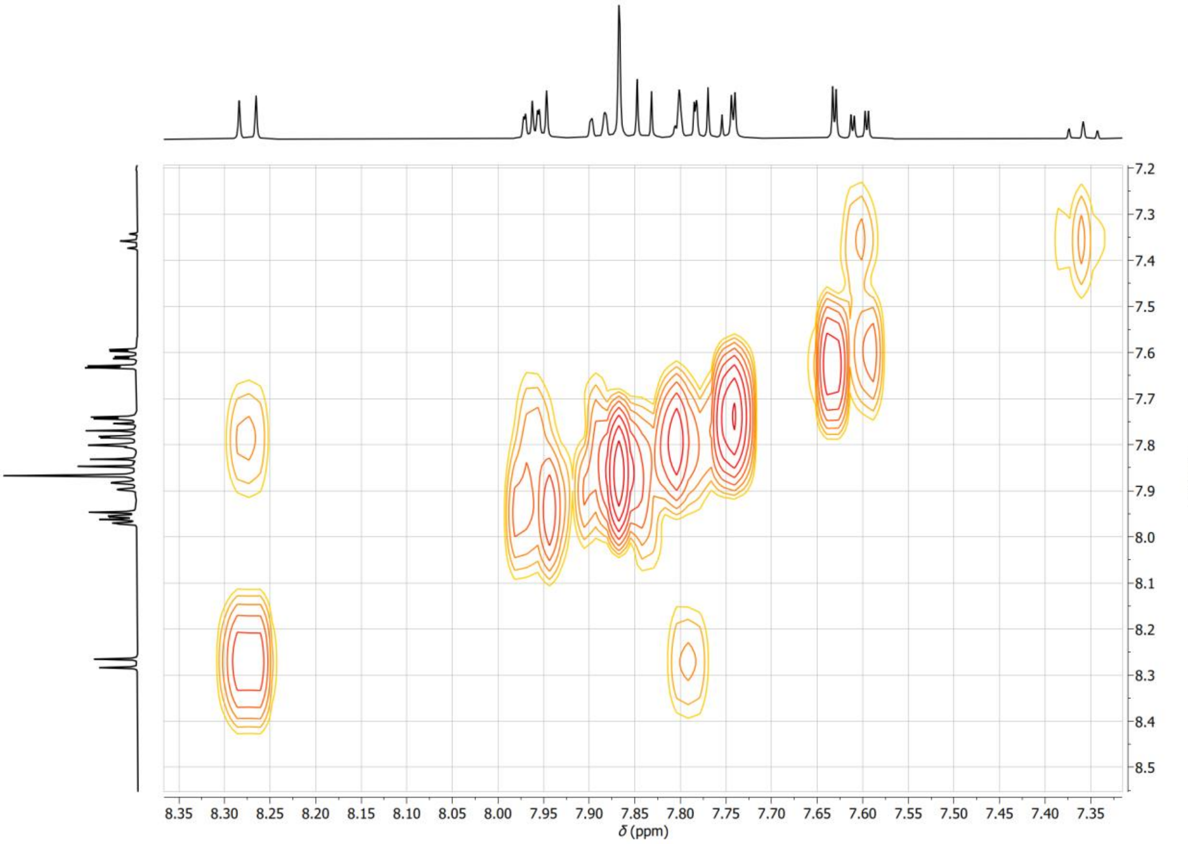


**Supplementary Fig. 57.** COSY spectrum (298 K, 500 MHz) of the aromatic region of $L^{Pyr}$ in $C_6D_6$.

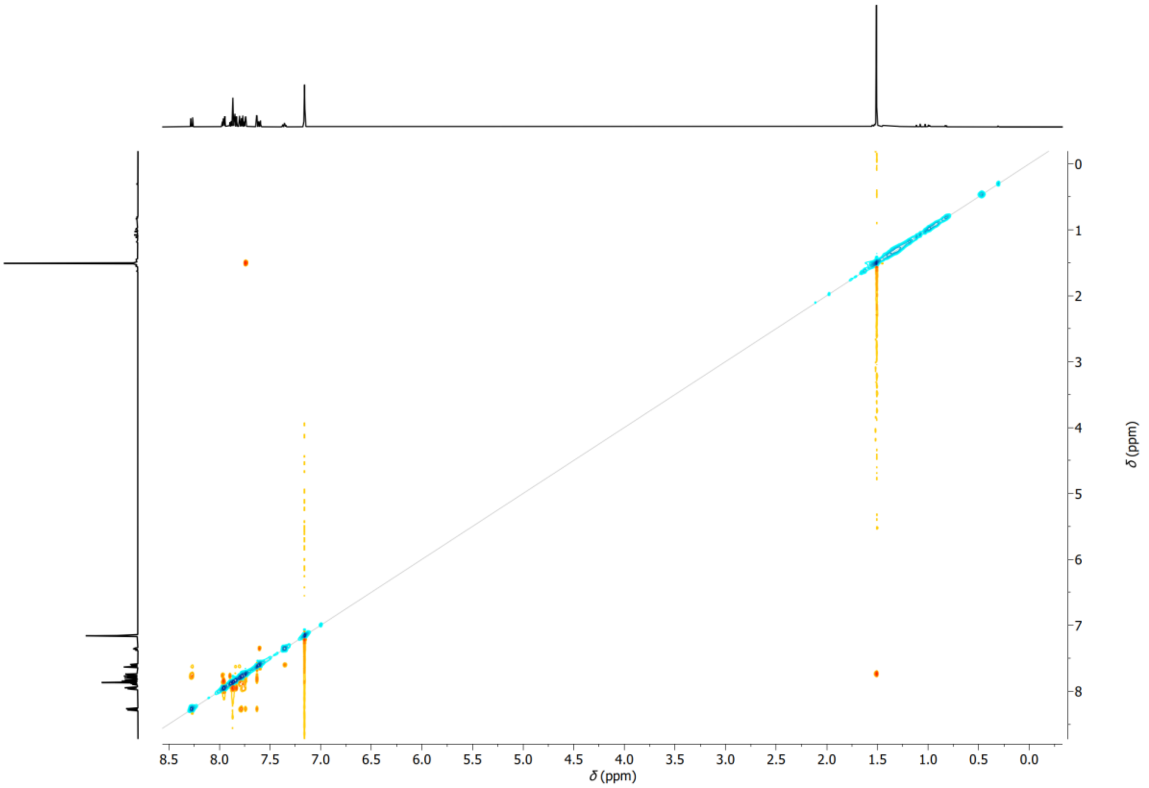


**Supplementary Fig. 58.** NOESY spectrum (298 K, 500 MHz) of L$^{Pyr}$ in $C_6D_6$.

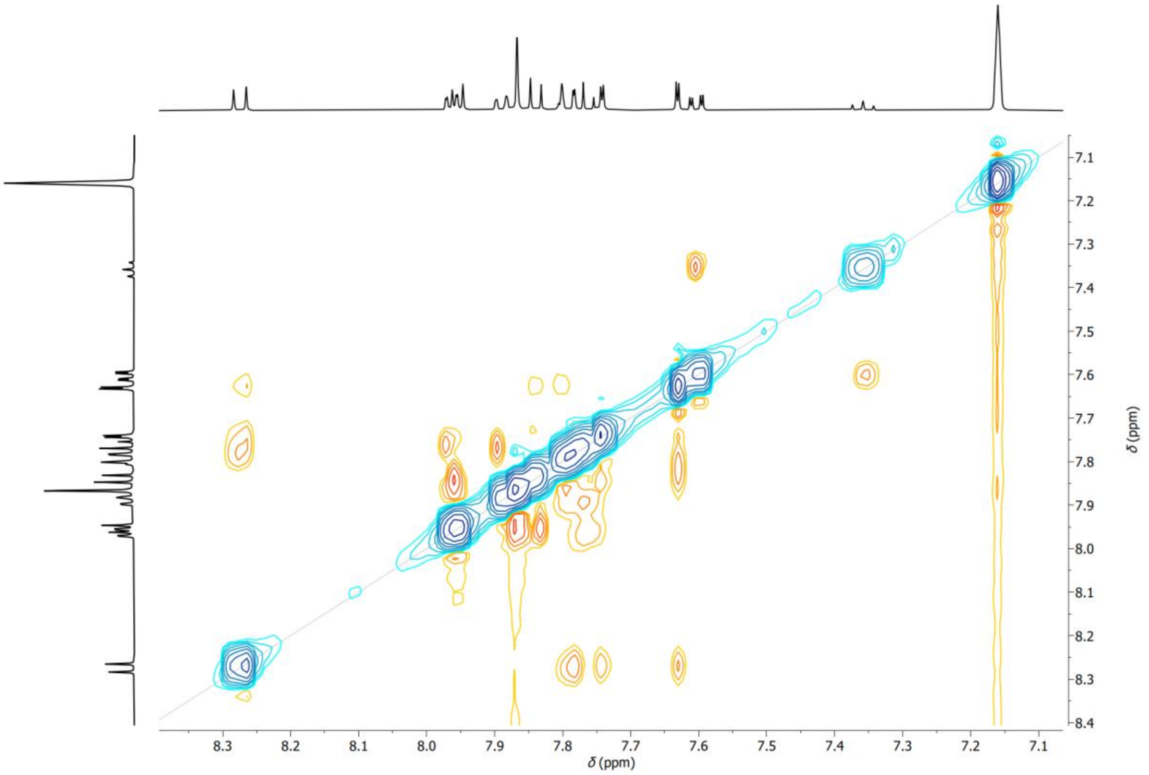


**Supplementary Fig. 59.** Zoom of the aromatic region of the NOESY spectrum (298 K, 500 MHz) of L$^{Pyr}$ in $C_6D_6$.

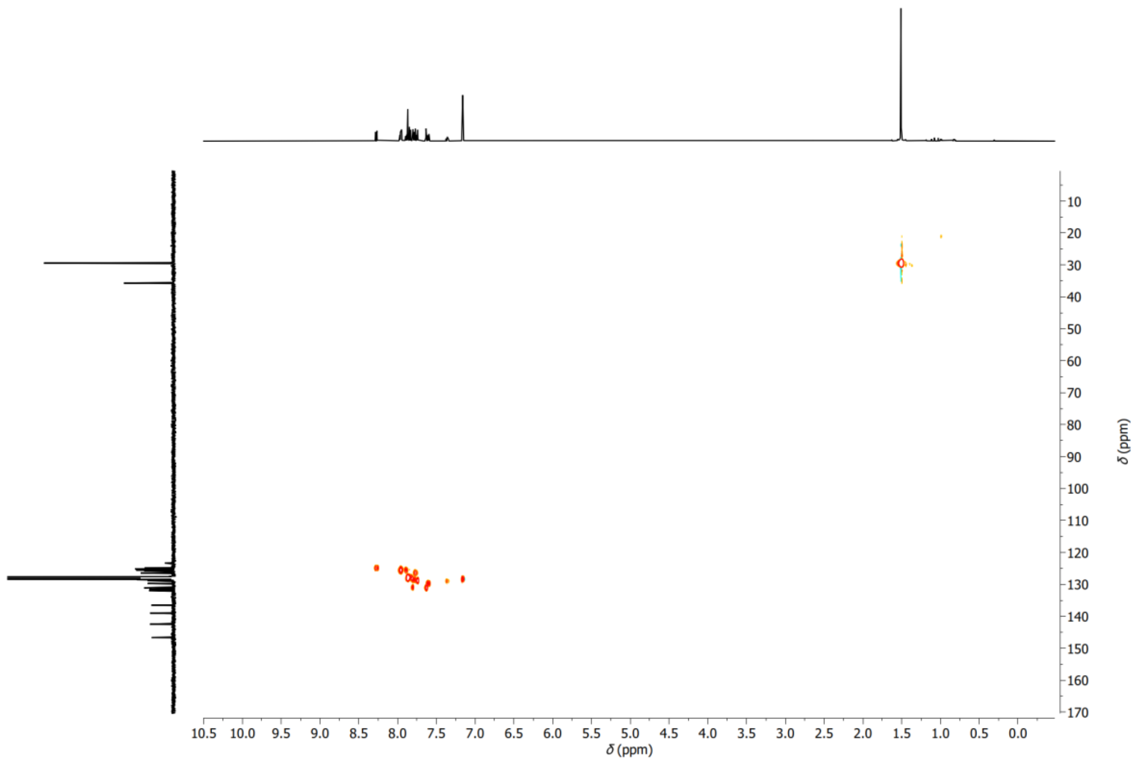


**Supplementary Fig. 60.** HMQC spectrum (298 K, 500 MHz) of $L^{Pyr}$ in $C_6D_6$.

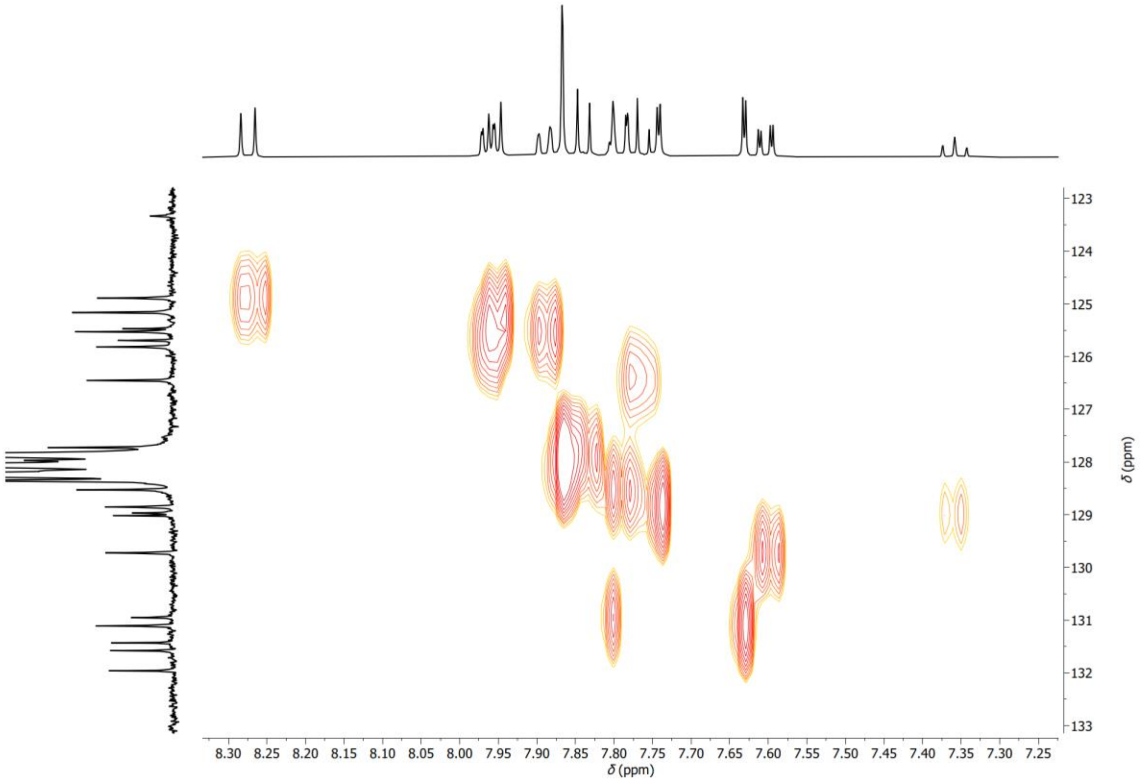


**Supplementary Fig. 61.** Zoom of the aromatic region of the HMQC spectrum (298 K, 500 MHz) of $L^{Pyr}$ in $C_6D_6$.

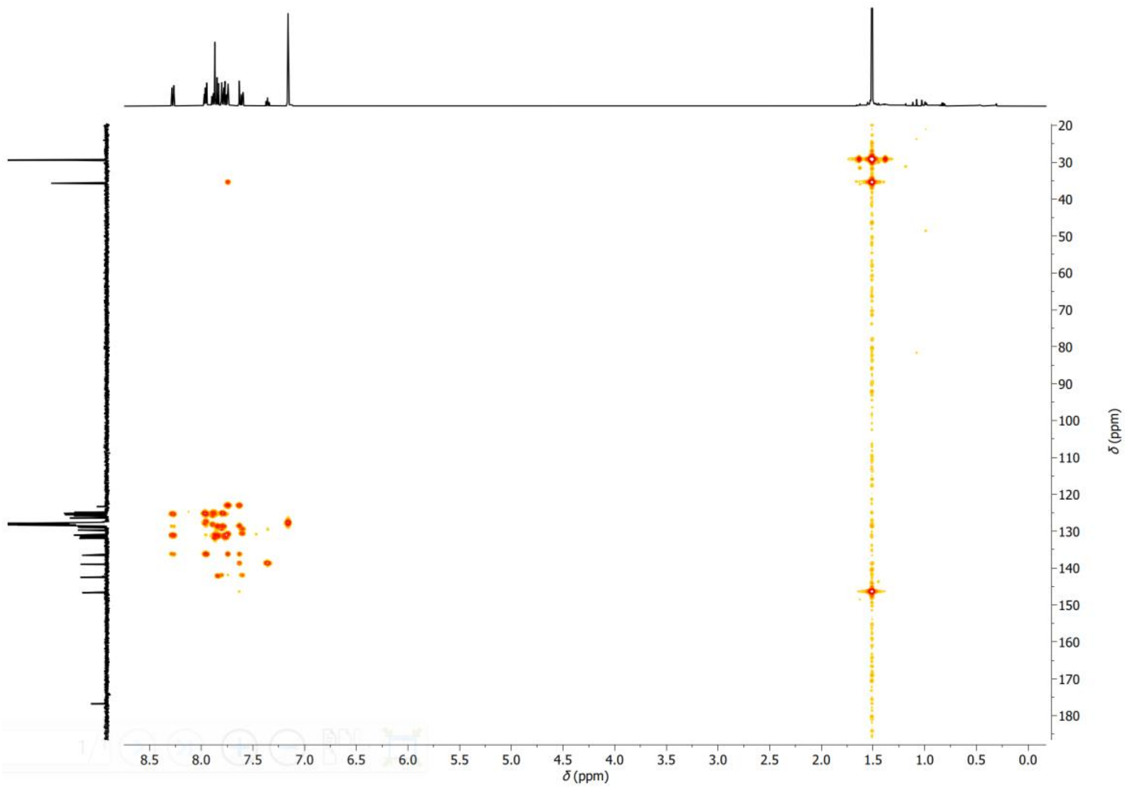


**Supplementary Fig. 62.** HMBC spectrum (298 K, 500 MHz) of $L^{Pyr}$ in $C_6D_6$.

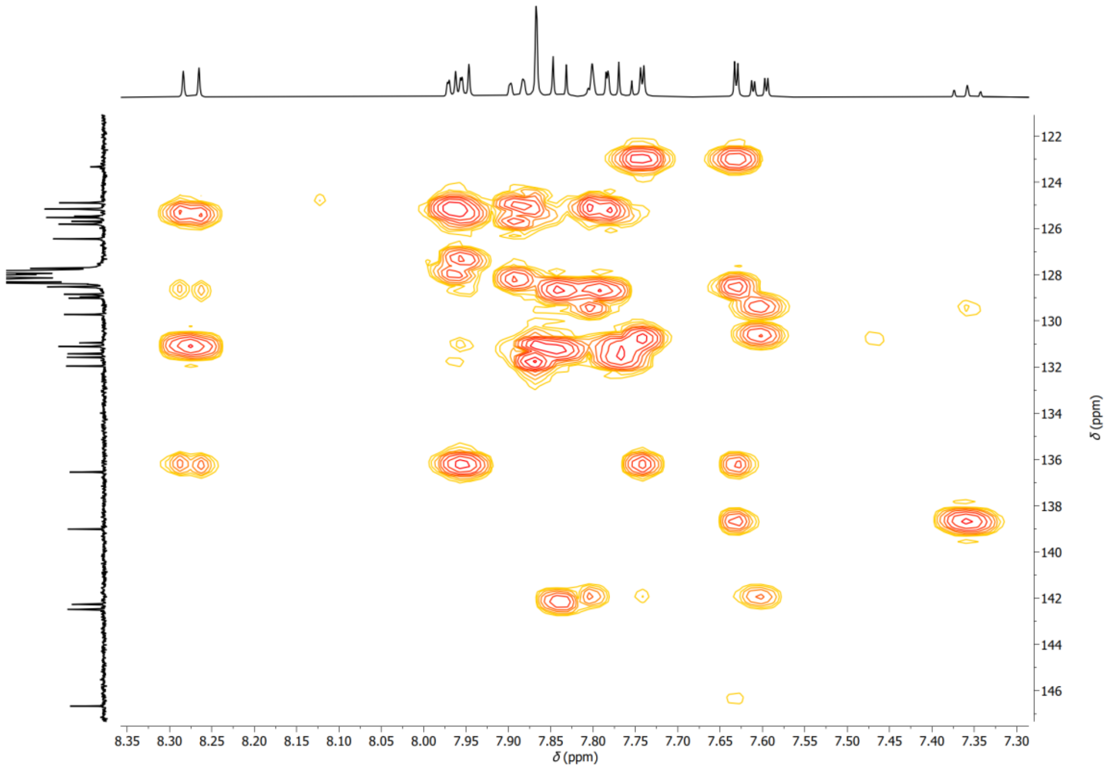


**Supplementary Fig. 63.** Zoom of the aromatic region of the HMBC spectrum (298 K, 500 MHz) of $L^{Pyr}$ in $C_6D_6$.

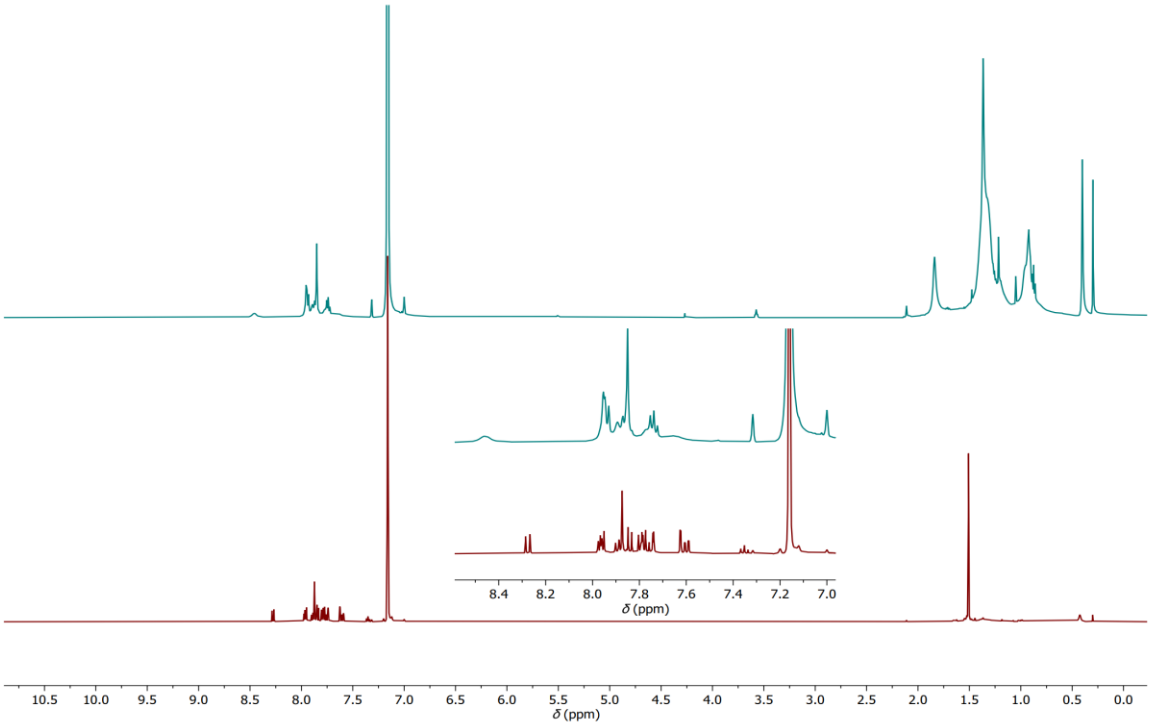


**Supplementary Fig. 64.** $^{1}H$ NMR spectra of [Cr(L$^{Pyr}$)$_3$] (top) and L$^{Pyr}$ (bottom) in $C_6D_6$ (298 K, 500 MHz). The solvent residual peak is observed at 7.16 ppm. The resonance at 1.84 ppm for [Cr(L$^{Pyr}$)$_3$] originates from the *tert*-butyl group, and the remaining aliphatic resonances originate from either silicon grease or *n*-pentane. Insert: Zoom of the aromatic region. See variable-temperature NMR studies presented further below for additional details.

### 3.4. HR-ESI mass spectra

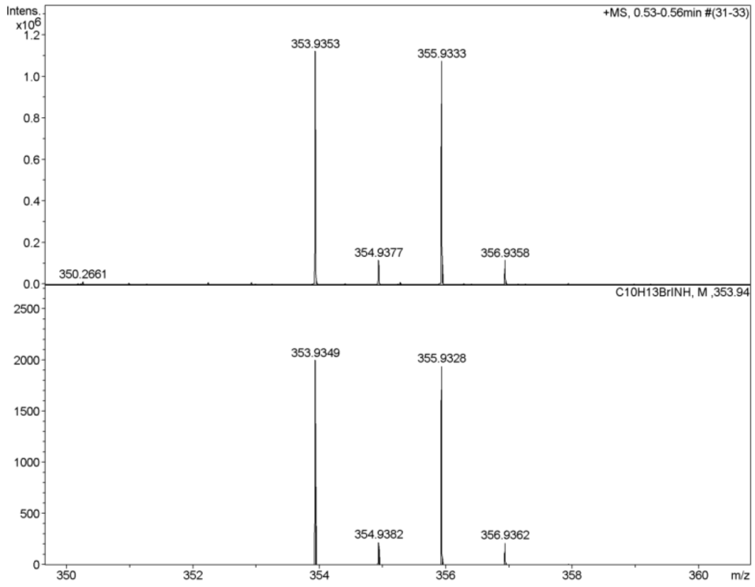


**Supplementary Fig. 65.** High-resolution ESI-MS spectra of the found (top) and calculated (bottom) isotope pattern of the ion [**8** + H]$^{+}$.

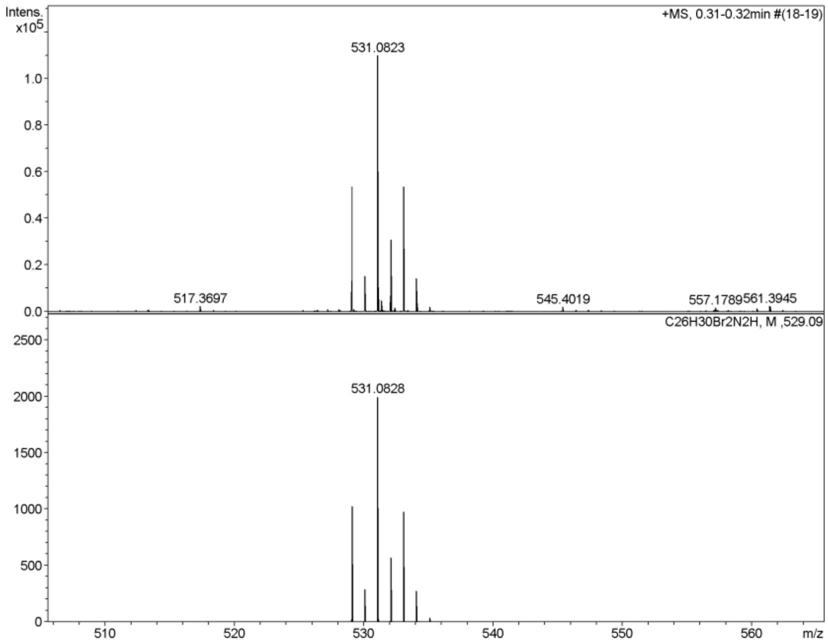


**Supplementary Fig. 66.** High-resolution ESI-MS spectra of the found (top) and calculated (bottom) isotope pattern of the ion [**9** + H]$^+$.

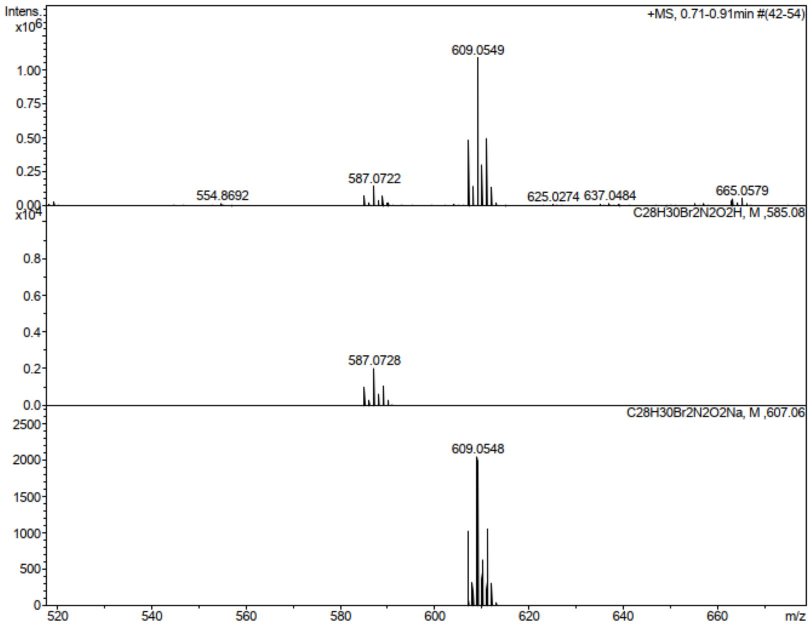


**Supplementary Fig. 67.** High-resolution ESI-MS spectra of the found (top) and calculated isotope pattern of the ions [**10** + H]$^+$ (middle) and [**10** + Na]$^+$ (bottom).

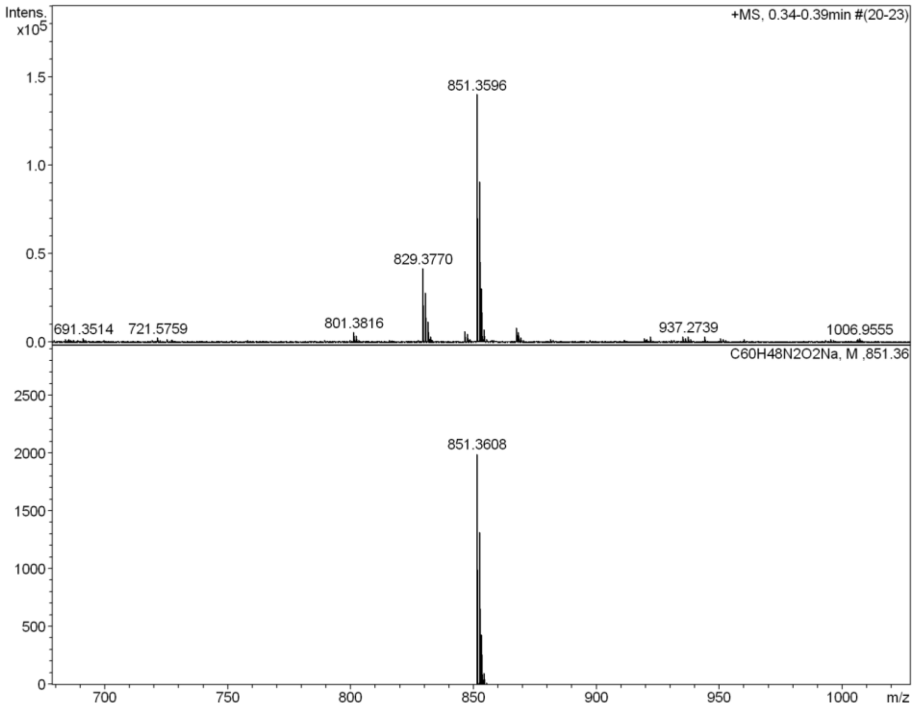


**Supplementary Fig. 68.** High-resolution ESI-MS spectra of the found (top) and calculated isotope pattern of the ion [**11** + Na]$^+$ (bottom).

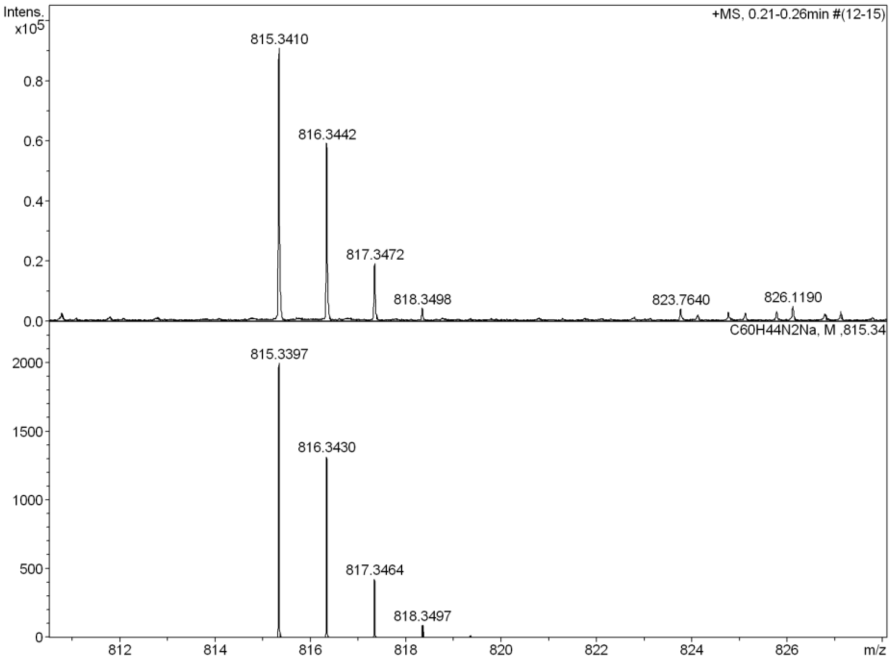


**Supplementary Fig. 69.** High-resolution ESI-MS spectra of the found (top) and calculated isotope pattern of the ion [L$^{Pyr}$ + Na]$^+$ (bottom).

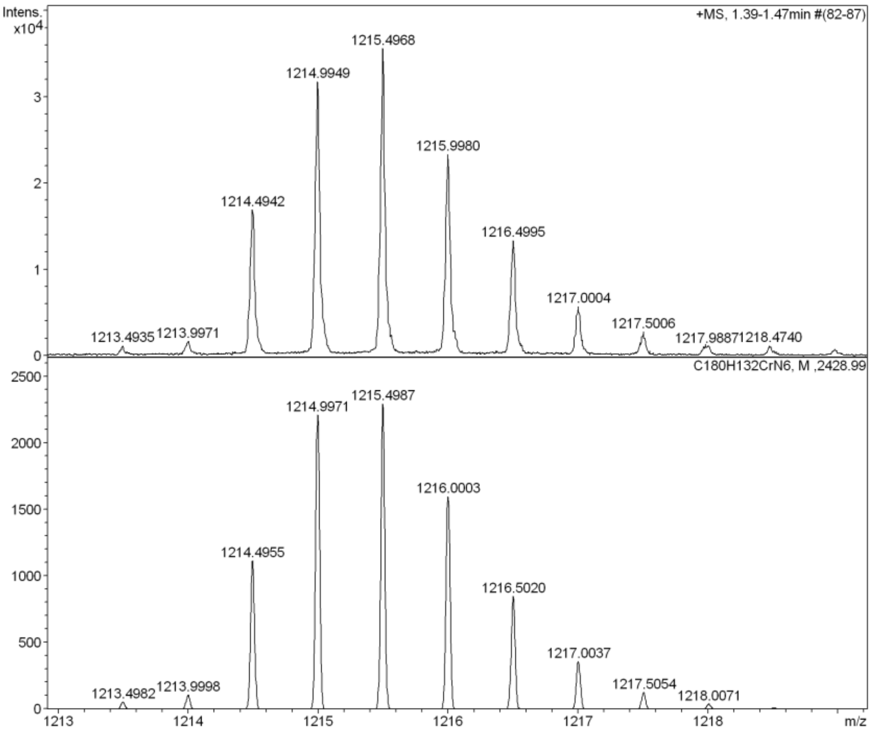


**Supplementary Fig. 70.** High-resolution ESI-MS spectra of the found (top) and calculated (bottom) isotope pattern of the ion $[Cr(L^{Pyr})_3]^{2+}$.

## 3.5. FT-IR spectra

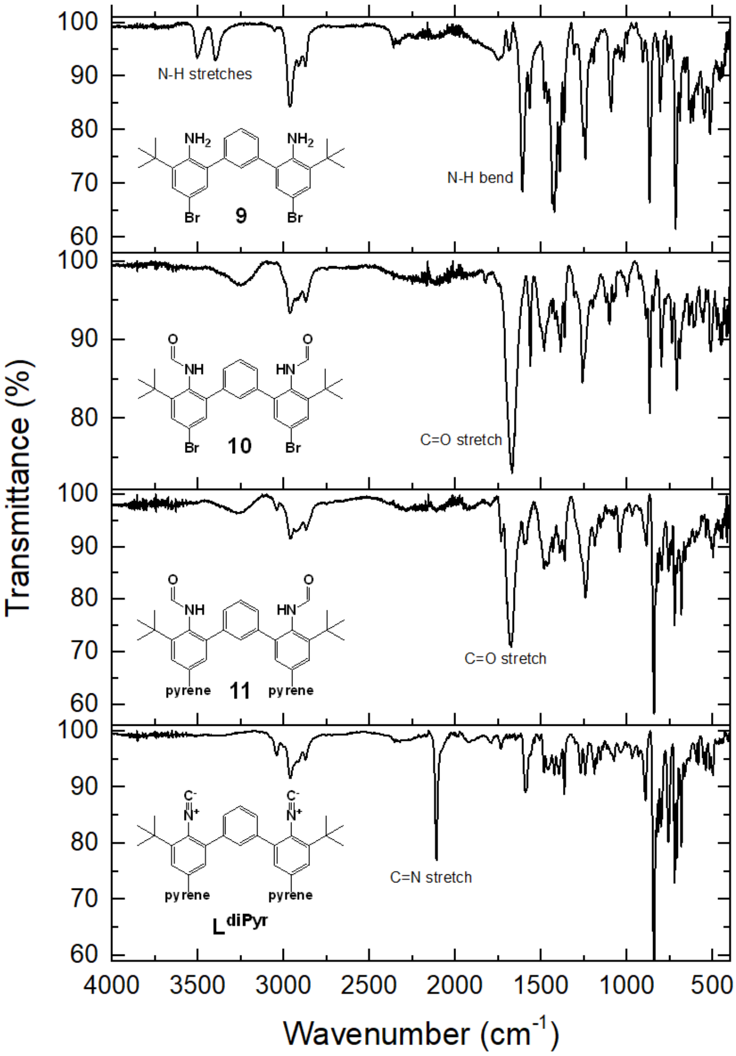


**Supplementary Fig. 71.** Stacked solid state IR spectra of compounds **9**, **10**, **11** and L$^{Pyr}$. The vibrational modes of the functional groups of the compounds are indicated in the various spectra.

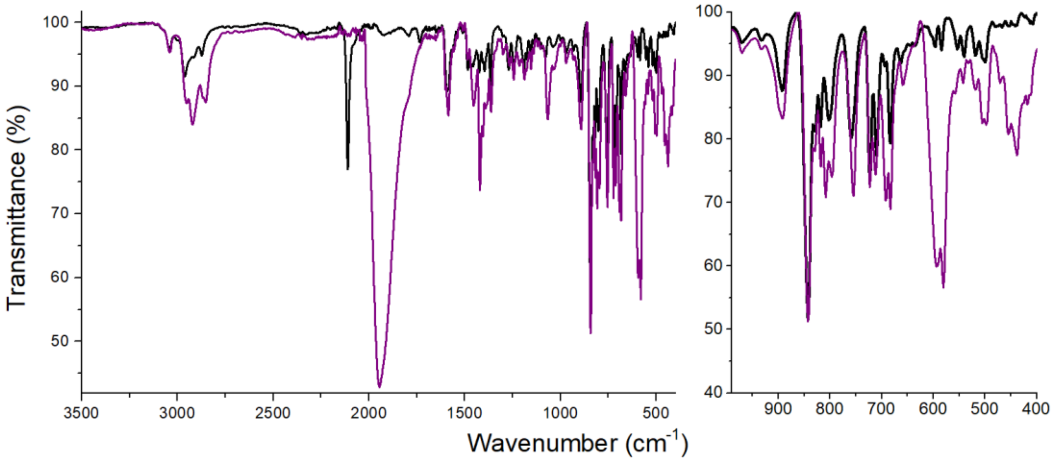


**Supplementary Fig. 72.** Solid state IR spectra of L$^{Pyr}$ (black) and [Cr(L$^{Pyr}$)$_3$] (purple). Left: Full spectrum, right: Zoom of the region between 1000 cm$^{-1}$ and 400 cm$^{-1}$ to highlight the Cr–C bending frequency at 590 – 580 cm$^{-1}$. The C≡N stretch frequencies for of L$^{Pyr}$ and [Cr(L$^{Pyr}$)$_3$] are found at 2111 and 1945 cm$^{-1}$, respectively.

### 3.6 Temperature-dependent $^{1}H$ NMR study of $[Cr(L^{Pyr})_3]$

Temperature-dependent NMR studies (198 K to 378 K) were performed with $[Cr(L^{Pyr})_3]$ in toluene-$d_8$ to investigate the dynamic behavior of this $Cr^0$ complex. At room temperature, the $^{1}H$ NMR spectrum of $[Cr(L^{Pyr})_3]$ shows broad resonances, however, upon heating to 378 K the resonances sharpen into well-resolved resonances making full assignment ($^{1}H$ and $^{13}C$) of $[Cr(L^{Pyr})_3]$ possible.

The line width of the *tert*-butyl proton resonances at 198 K is 60 Hz, compared to a line width of 10 Hz for the aromatic protons, is evidence for a relatively lower coalescence temperature for the *tert*-butyl protons. This strongly suggests that the underlying dynamic processes of $[Cr(L^{Pyr})_3]$ are centered on the *tert*-butyl groups.

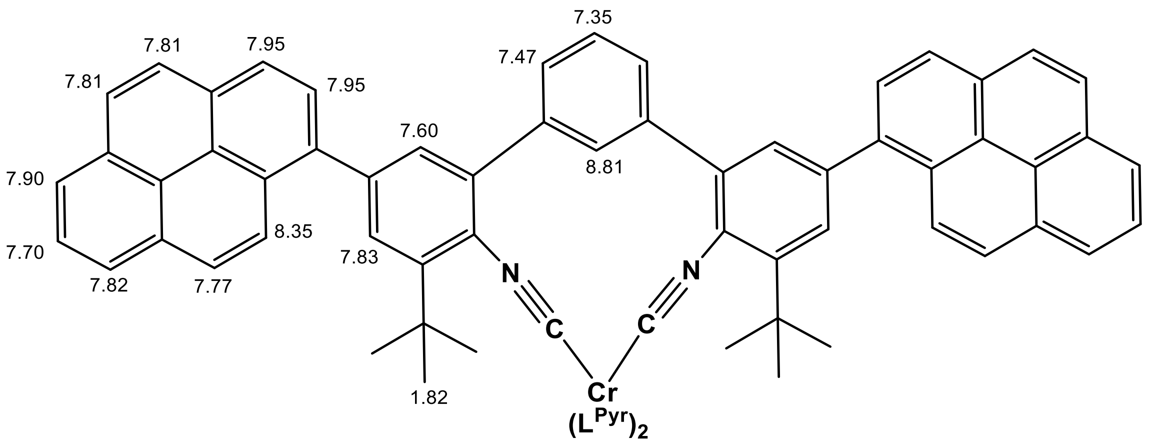


**Supplementary Fig. 73.** Assignment of the measured $^{1}H$-NMR resonances of $[Cr(L^{Pyr})_3]$ in toluene-$d_8$ at 378 K. The 2D NMR techniques COSY (Supplementary Fig. 82) and NOESY (Supplementary Fig. 84) were used to assign the protons.

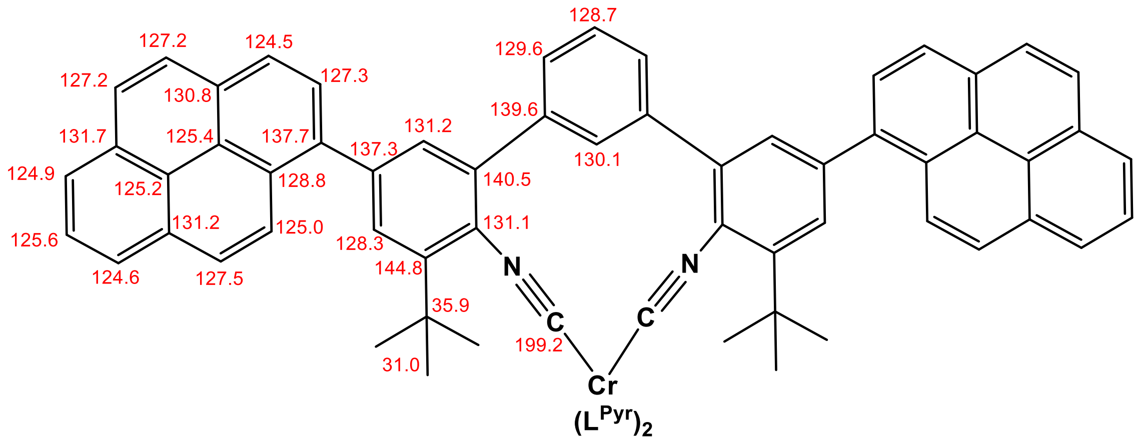


**Supplementary Fig. 74.** Assignment of the measured $^{13}C$-NMR resonances of $[Cr(L^{Pyr})_3]$ in toluene-$d_8$ at 378 K. A combination of $^{13}C$ NMR (Supplementary Fig. 78) and DEPT-45 (Supplementary Fig. 79) experiments were used to record all $^{13}C$-NMR resonances (Supplementary Fig. 80). The chemical shift of the isocyanide carbon was recorded at 333 K (Supplementary Fig. 81). The 2D NMR techniques HSQC (Supplementary Fig. 85, 86) and HMBC (Supplementary Fig. 87) were used to establish the coupling between $^{1}H$ and $^{13}C$.

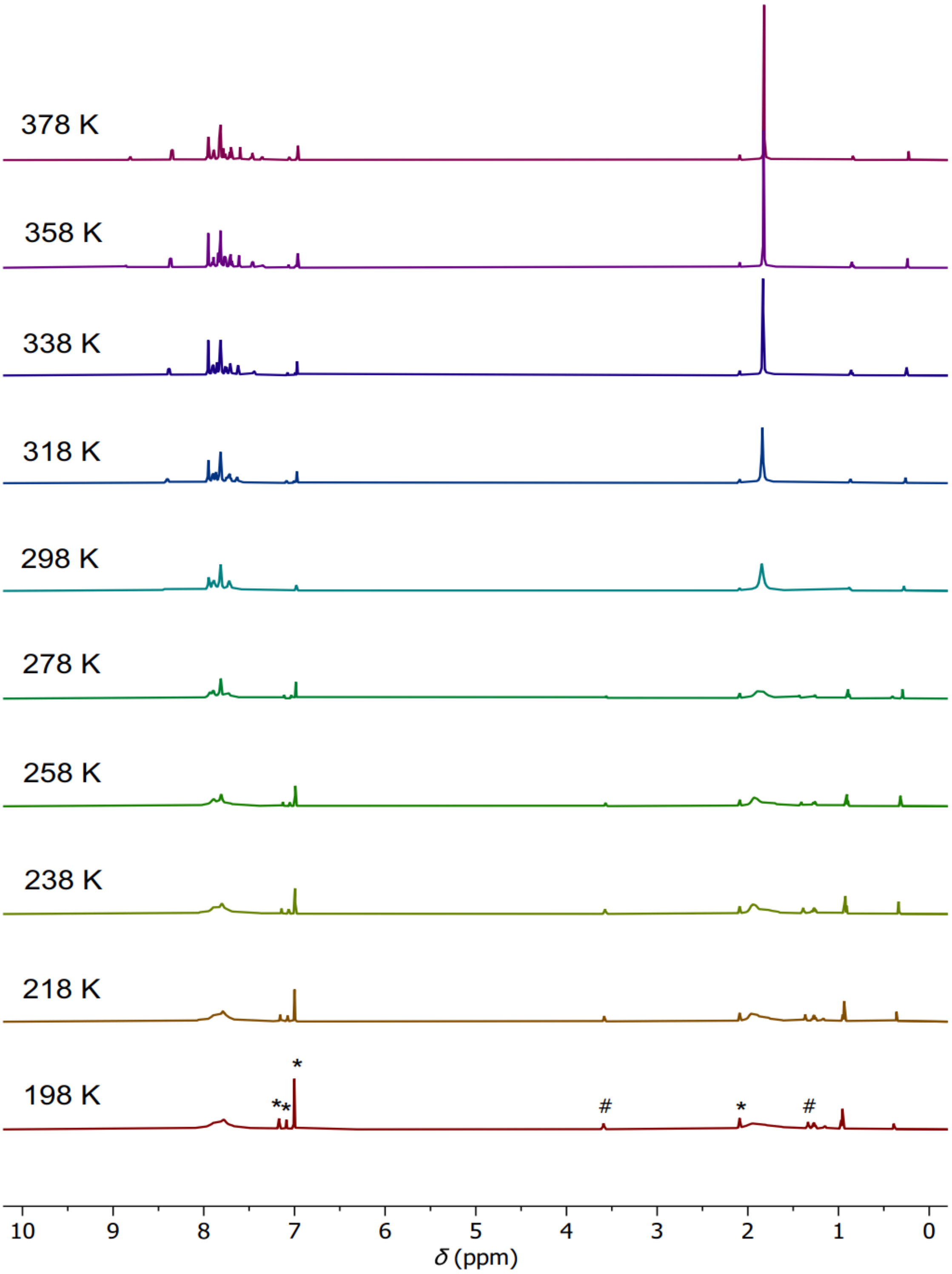


**Supplementary Fig. 75.** Stacked $^{1}H$ NMR spectra (600 MHz) of $[Cr(L^{Pyr})_3]$ in toluene-$d_8$ (*) at different temperatures as indicated in the figure. # = THF.

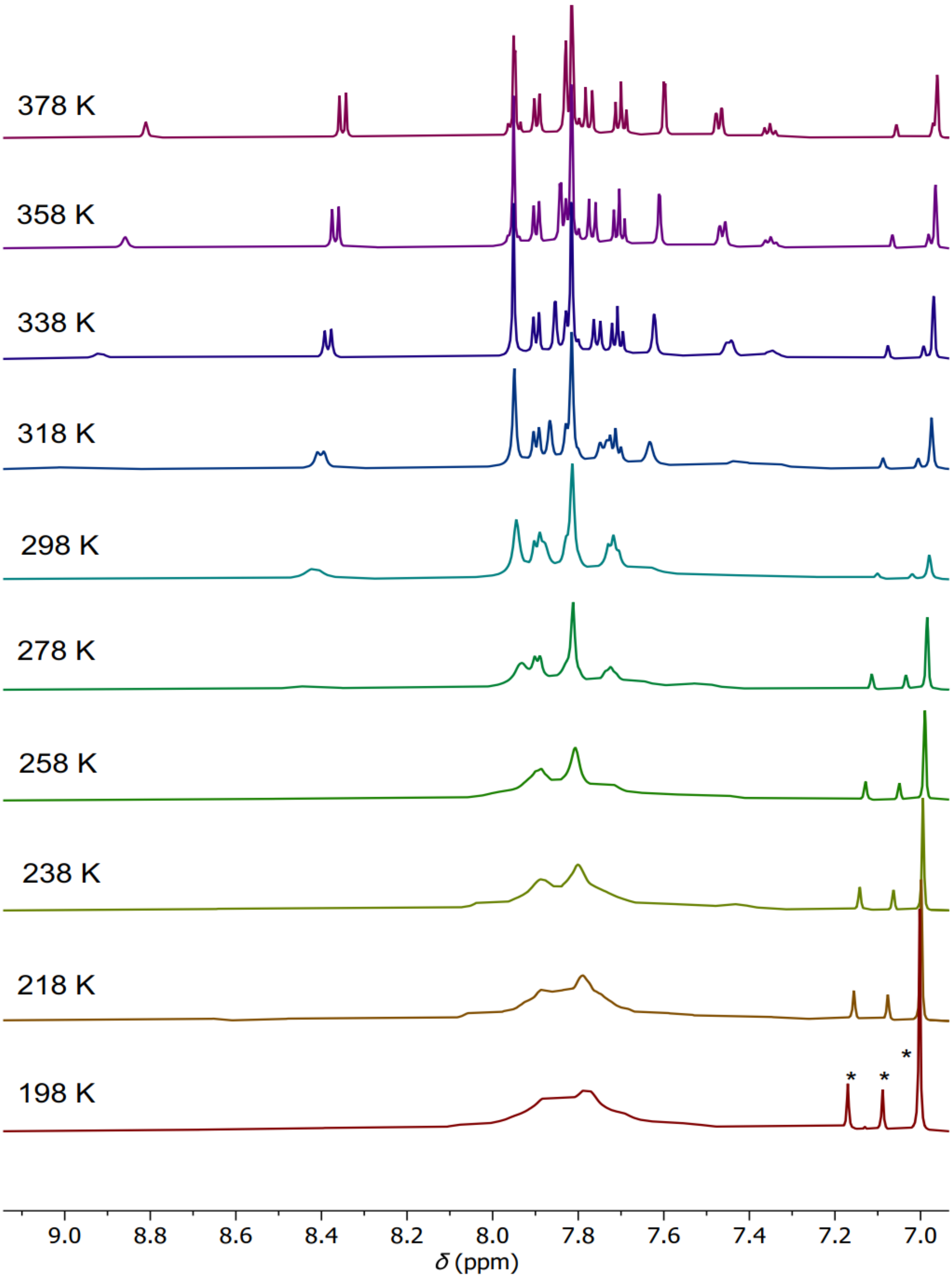


**Supplementary Fig. 76.** Zoom of the aromatic region of stacked $^{1}$H NMR spectra (600 MHz) of [Cr(L$^{Pyr}$)$_3$] in toluene-d$_8$ (*) at different temperatures as indicated in the figure.

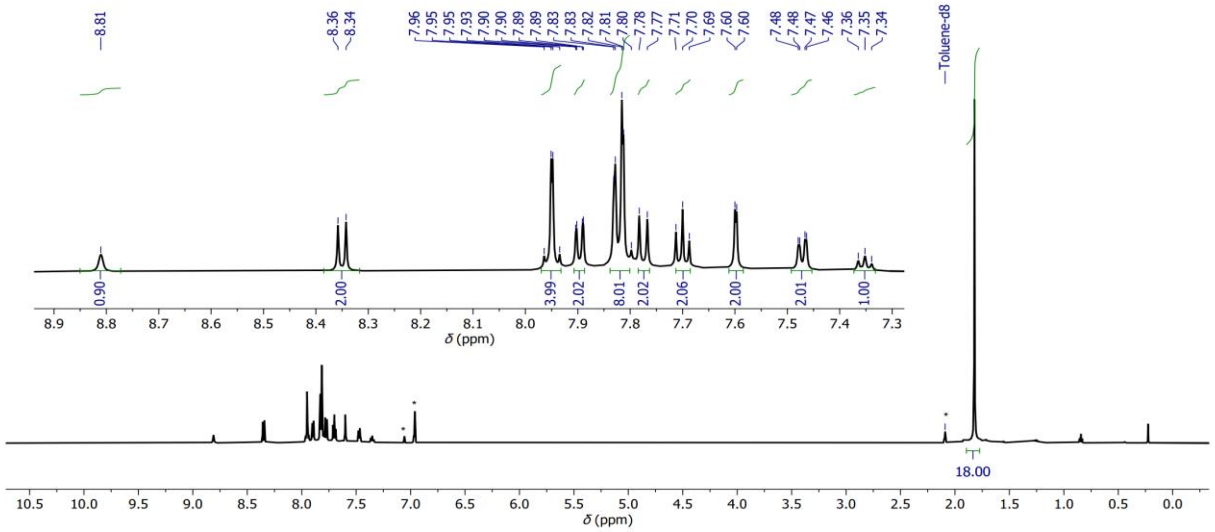


**Supplementary Fig. 77.** $^{1}H$ NMR spectra (600 MHz) of $[Cr(L^{Pyr})_3]$ in toluene-d$_8$ (*) at 378 K. Insert: Zoom of the aromatic region.

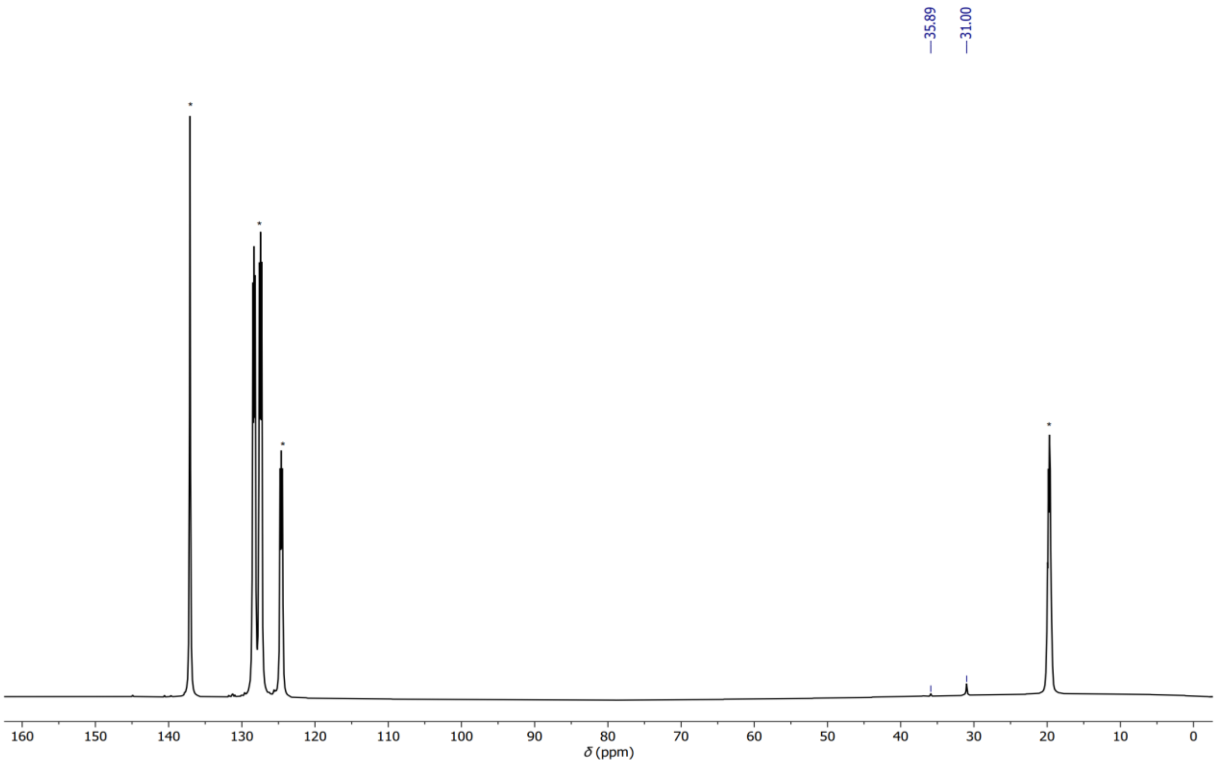


**Supplementary Fig. 78.** $^{13}C\{^{1}H\}$ NMR spectrum (151 MHz) of $[Cr(L^{Pyr})_3]$ in toluene-d$_8$ (*) at 378 K.

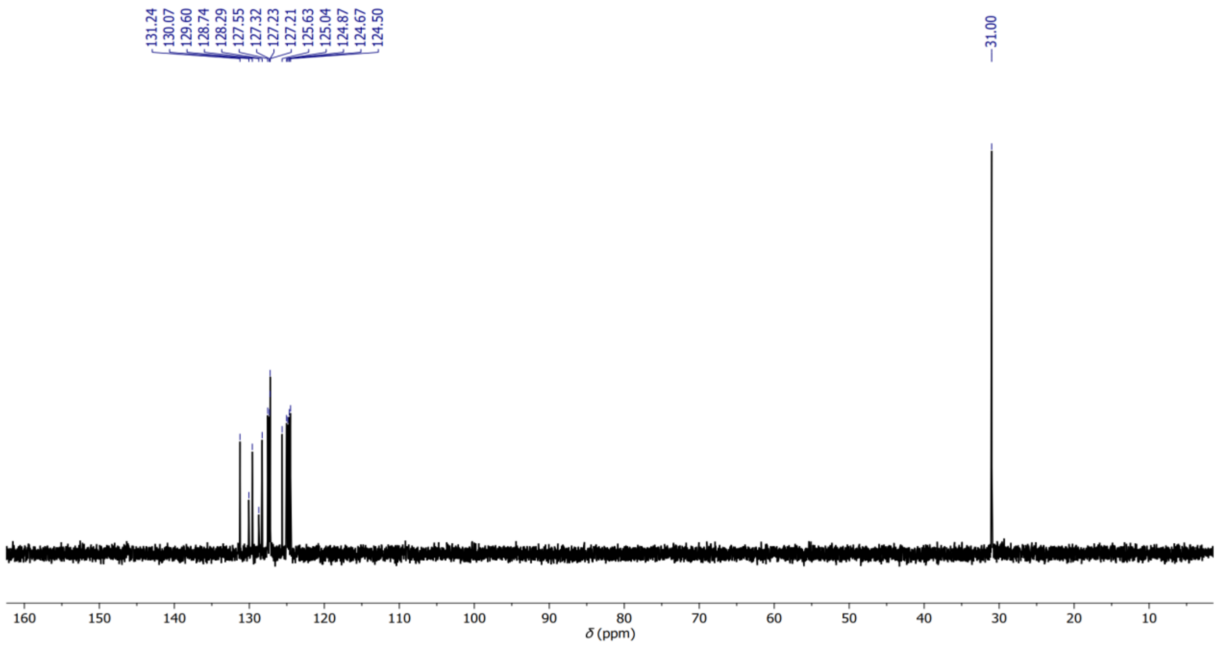


**Supplementary Fig. 79.** DEPT-45 spectrum (151 MHz) of [Cr($L^{Pyr}$)$_3$] in toluene-d$_8$ at 378 K.

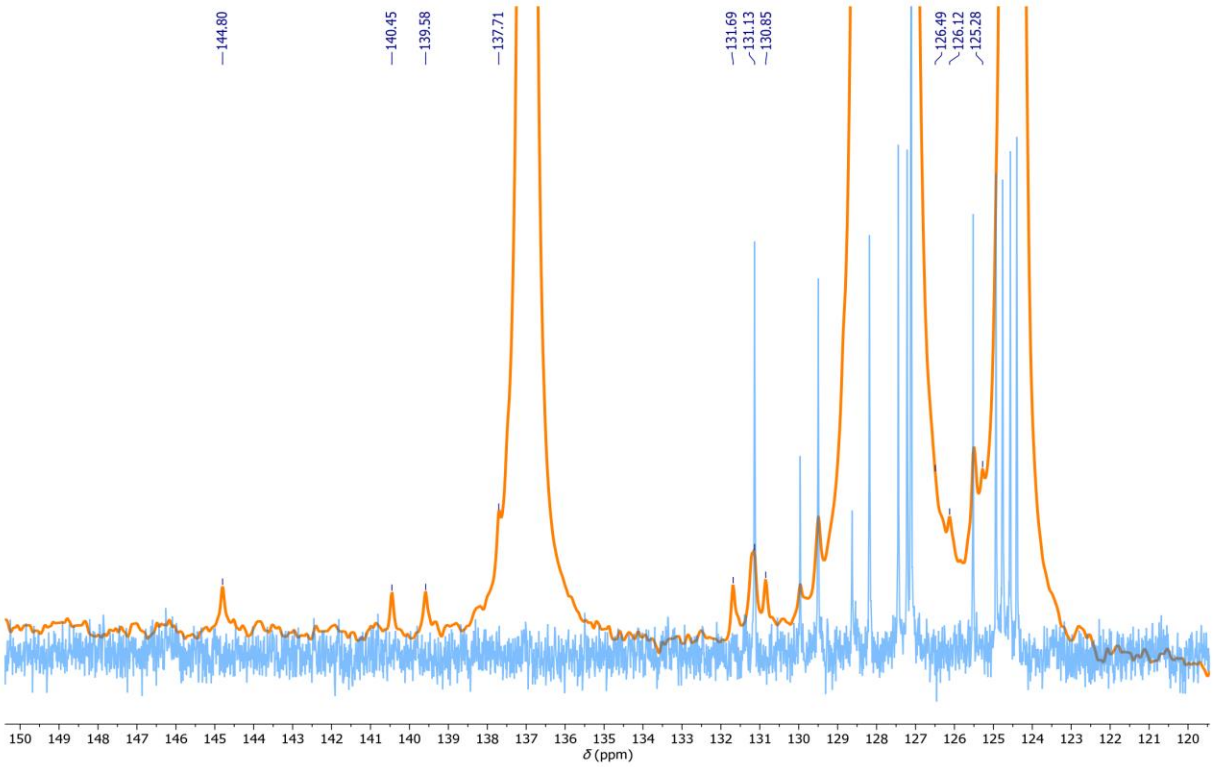


**Supplementary Fig. 80.** Overlaying $^{13}C\{^1H\}$ NMR spectra (orange, 151 MHz) and DEPT-45 (blue, 151 MHz) of [Cr($L^{Pyr}$)$_3$] in toluene-d$_8$ at 378 K.

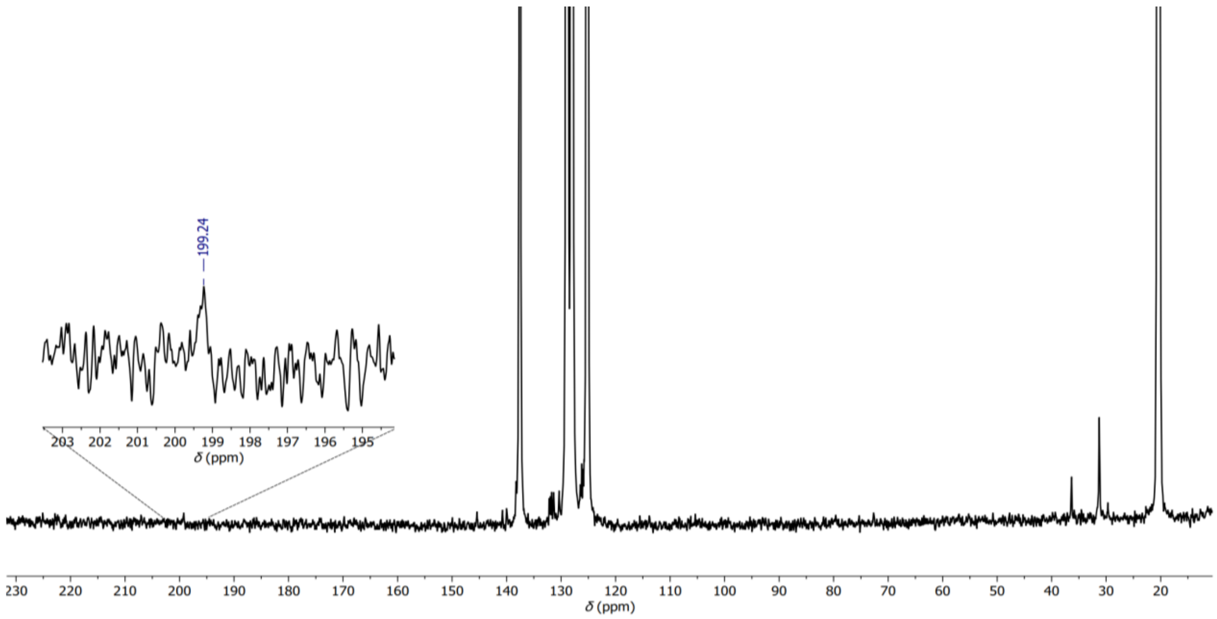


**Supplementary Fig. 81.** $^{13}C\{^{1}H\}$ NMR spectrum (151 MHz) of $[Cr(L^{Pyr})_3]$ in toluene-$d_8$ at 333 K. The spectrum was recorded over 4 days. The isocyanide carbon resonance is found at 199.2 ppm.

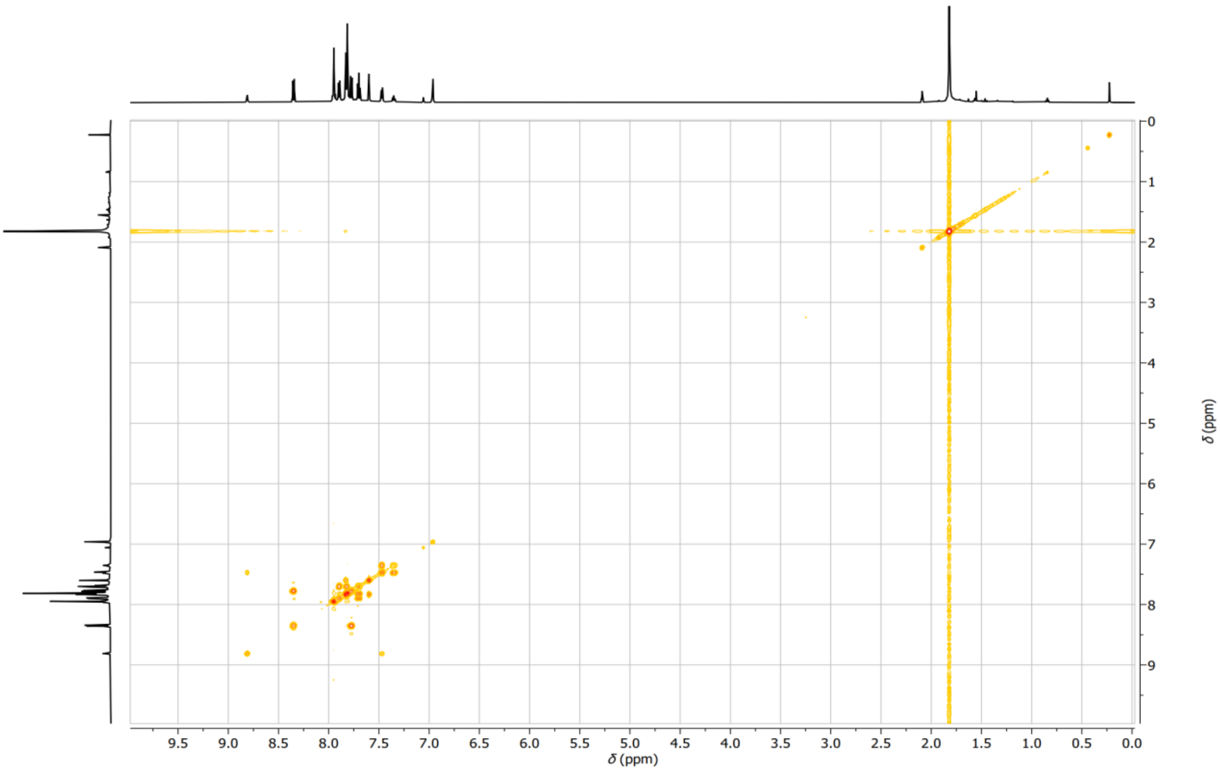


**Supplementary Fig. 82.** COSY spectrum (600 MHz) of $[Cr(L^{Pyr})_3]$ in toluene-$d_8$ at 378 K.

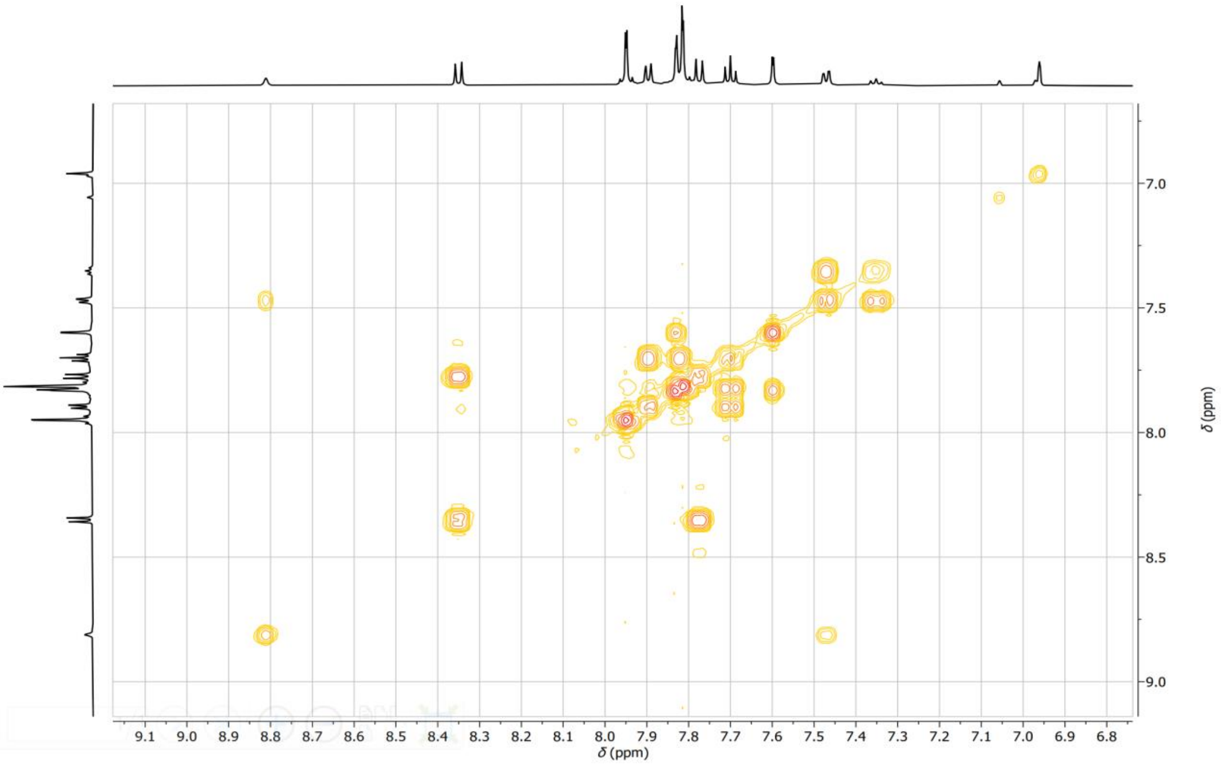


**Supplementary Fig. 83.** Zoom of the aromatic region of the COSY spectrum (600 MHz) of $[Cr(L^{Pyr})_3]$ in toluene-$d_8$ at 378 K.

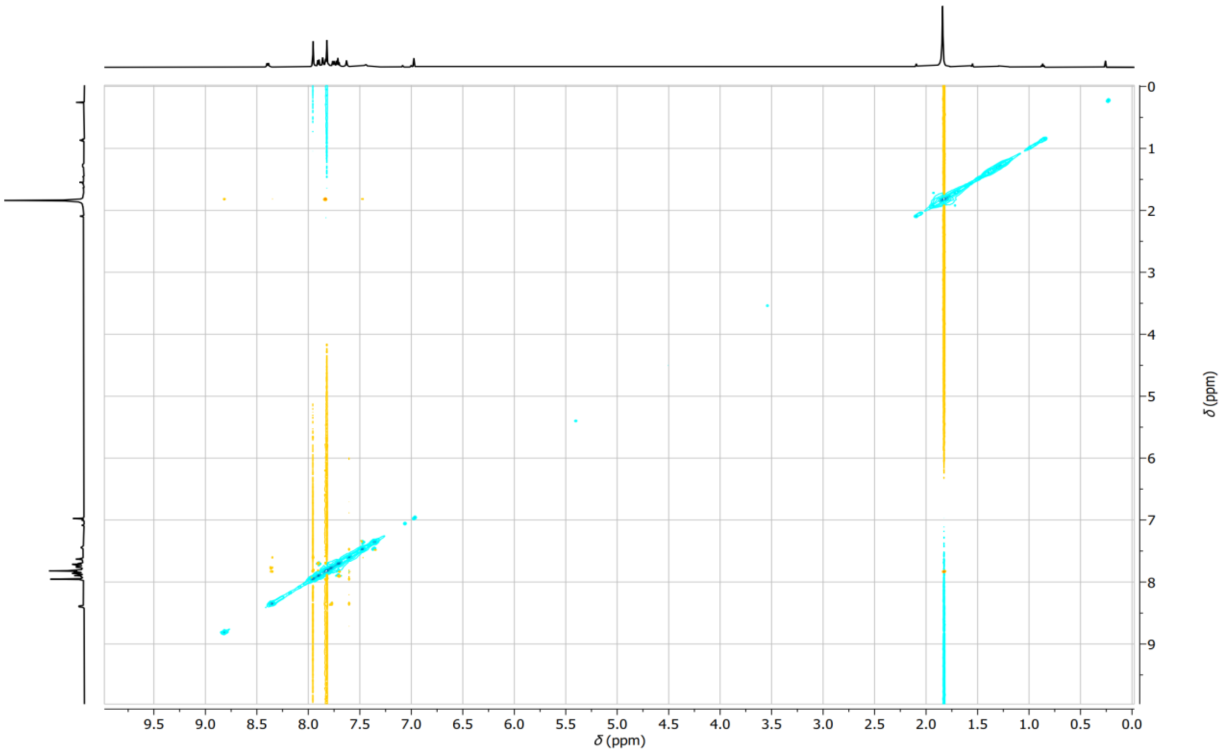


**Supplementary Fig. 84.** NOESY spectrum (600 MHz) of $[Cr(L^{Pyr})_3]$ in toluene-$d_8$ at 378 K.

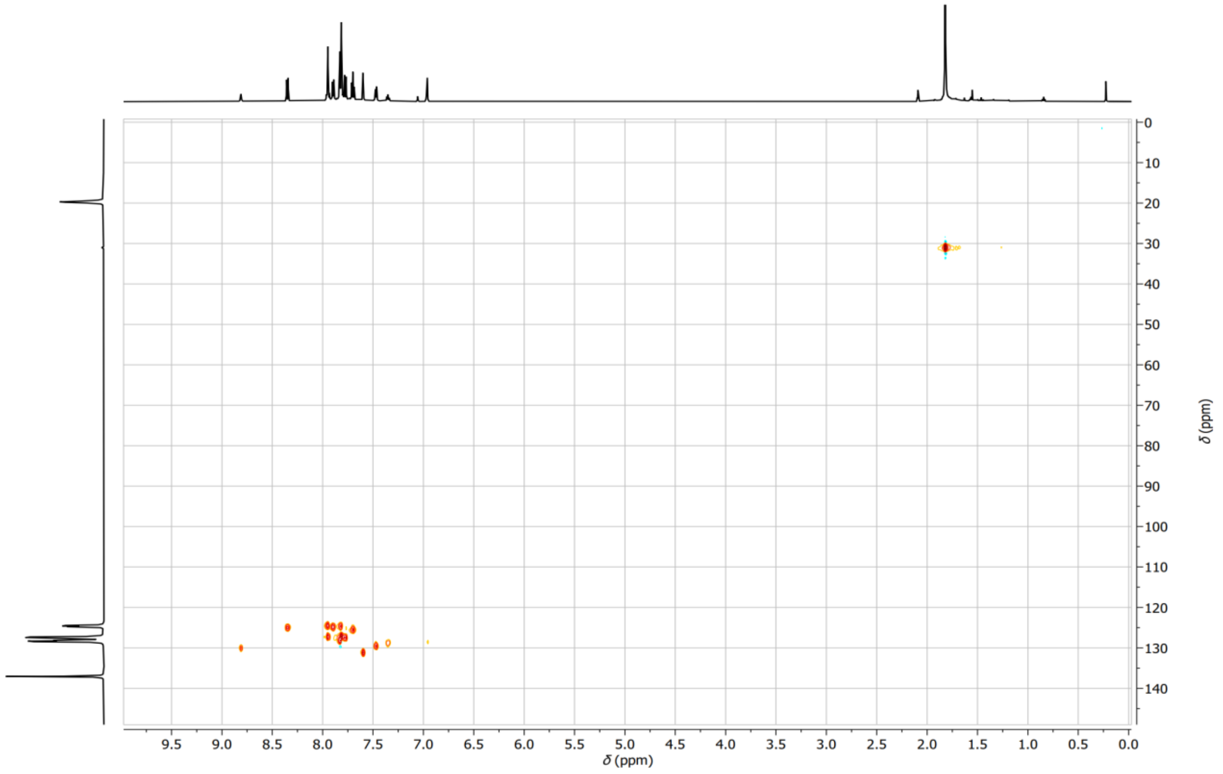


**Supplementary Fig. 85.** HSQC spectrum (600 MHz) of [Cr($L^{Pyr}$)$_3$] in toluene-$d_8$ at 378 K.

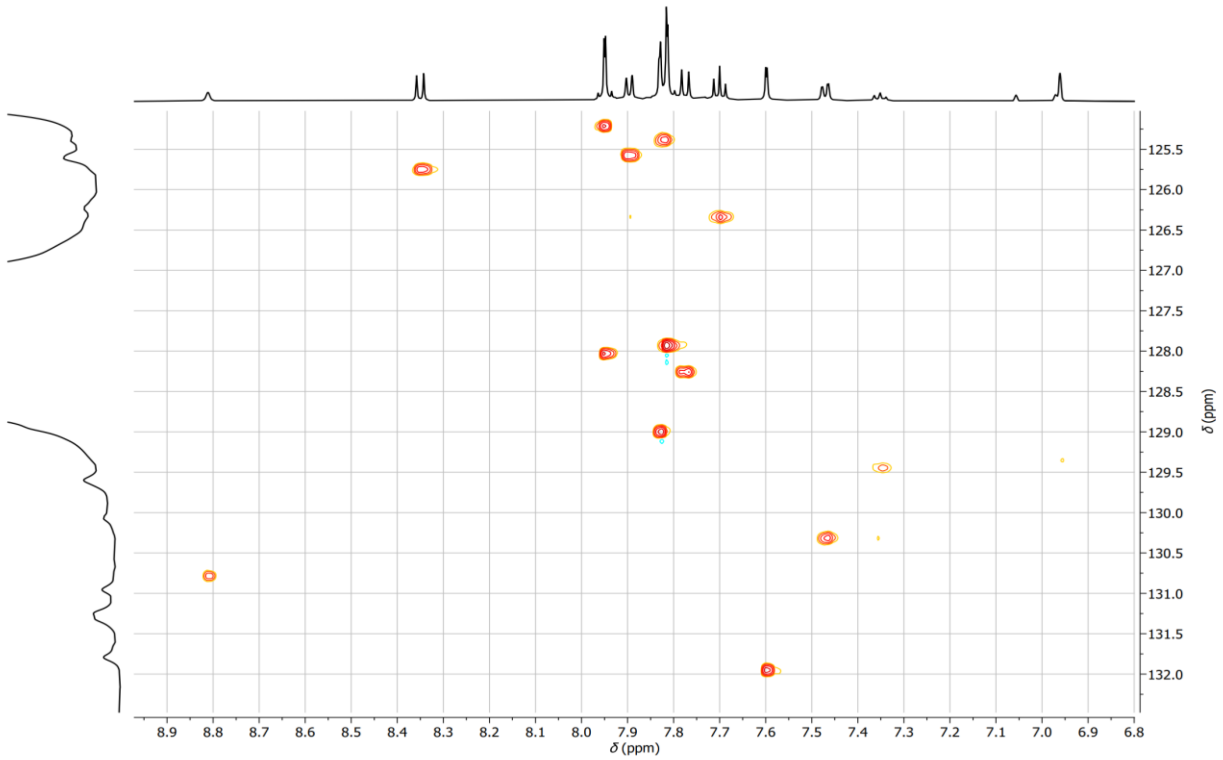


**Supplementary Fig. 86.** Zoom of the aromatic region of the HSQC spectrum (600 MHz) of [Cr($L^{Pyr}$)$_3$] in toluene-$d_8$ at 378 K.

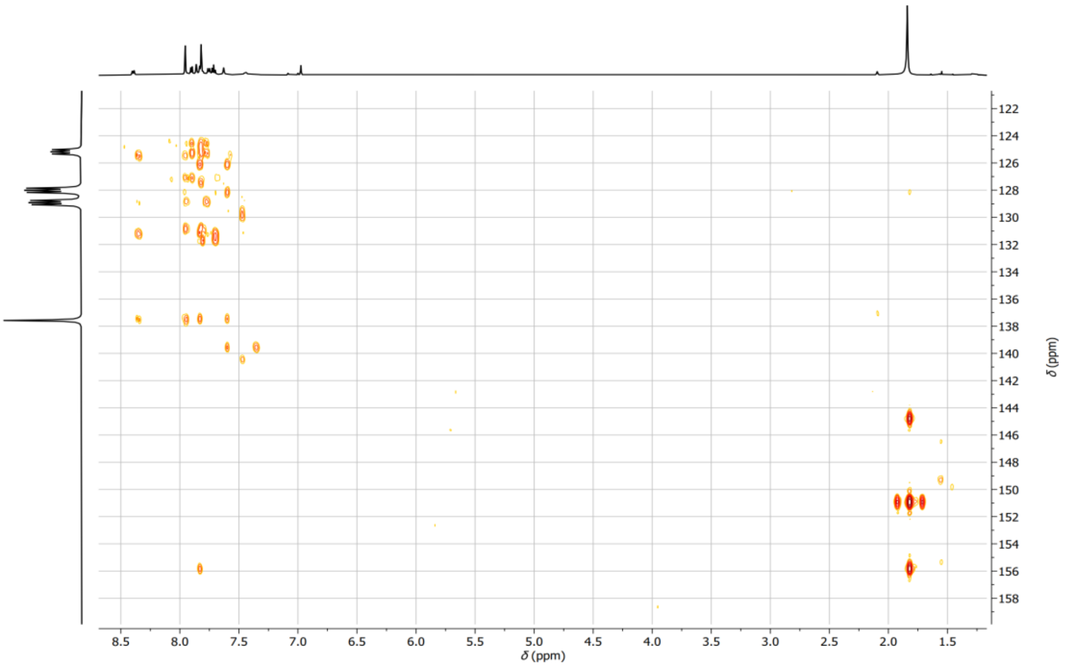


**Supplementary Fig. 87.** HMBC spectrum (600 MHz) of [Cr($L^{Pyr}$)$_3$] in toluene-d$_8$ at 378 K. For the sake of the resolution in the carbon dimension, the sweep width was limited to 60 ppm and, thus, the *tert*-butyl resonances appear aliased at multiples of 60 ppm.

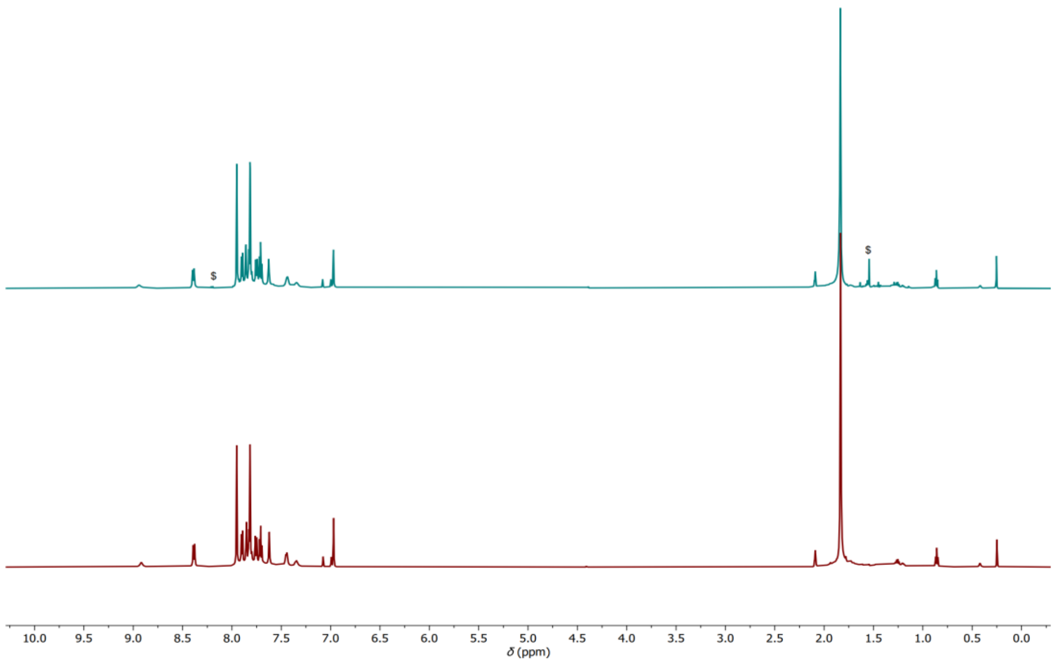


**Supplementary Fig. 88.** $^1$H NMR spectra (600 MHz, 333K) of [Cr($L^{Pyr}$)$_3$] in toluene-d$_8$. Initial spectrum (bottom) and spectrum recorded of the exact same sample after heating for three days at 378 K and additional four days at 333 K (top). Only 1% of free $L^{Pyr}$ ($) (considering one complex decomposes to three ligands) was observed after the total of one week at elevated temperatures, demonstrating the high thermal stability of [Cr($L^{Pyr}$)$_3$].

## 3.7. Cyclic voltammetry

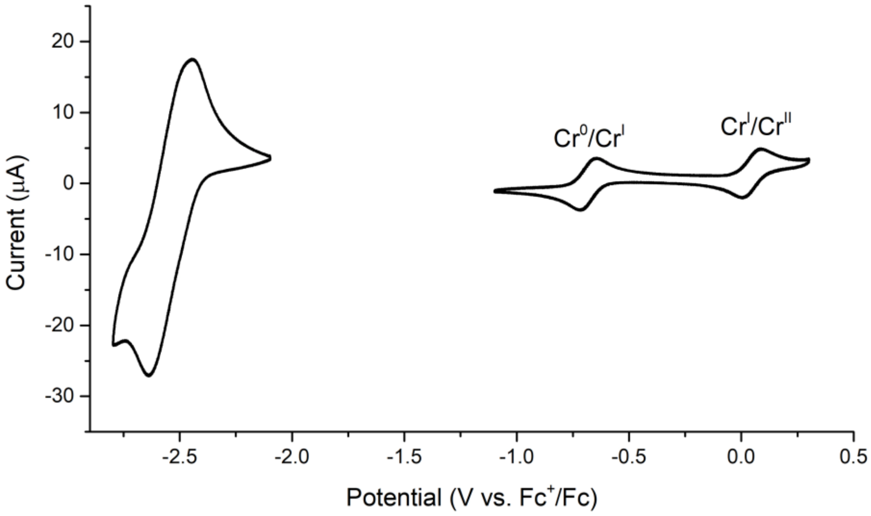


**Supplementary Fig. 89.** Cyclic voltammogram of $[Cr(L^{Pyr})_3]$ (1 mM) in THF with 0.2 M $TBAPF_6$ at 20 °C. The potential scan rate was 50 $mVs^{-1}$.

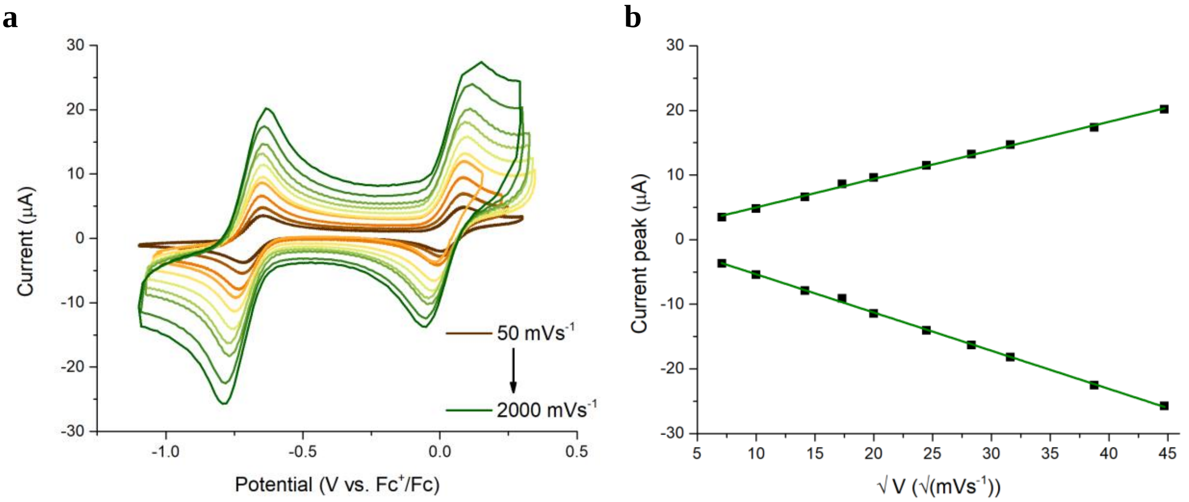


**Supplementary Fig. 90.** (**a**) Cyclic voltammogram of the $Cr^0/Cr^I$ and $Cr^I/Cr^{II}$ redox couples for $[Cr(L^{Pyr})_3]$ in deaerated THF containing 0.2 M $TBAPF_6$ at 20 °C. The scan rate was varied between 50 mV $s^{-1}$ and 2000 mV $s^{-1}$. (**b**) Randles−Sevcik plot for the $Cr^0/Cr^I$ redox couple from (**a**).

### 3.8. UV-Vis absorption and emission spectra

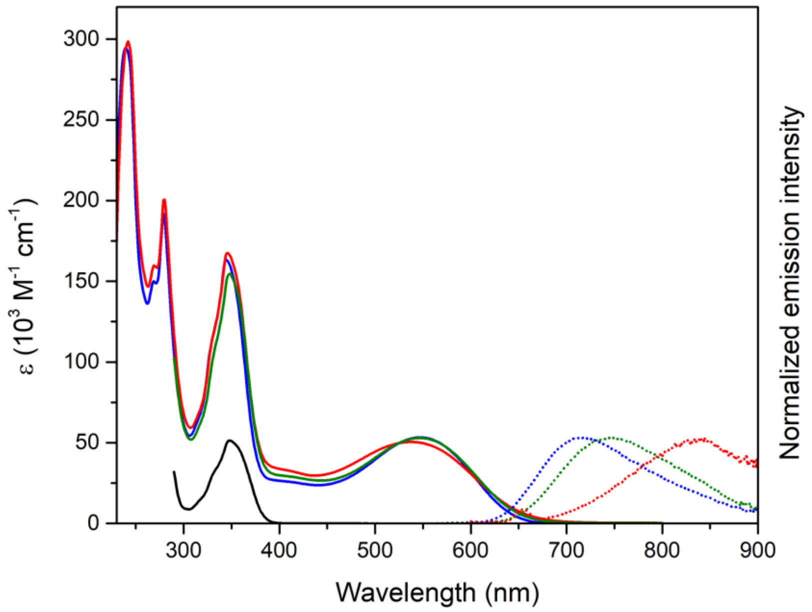


**Supplementary Fig. 91.** UV–Vis absorption (solid lines) and emission spectra (dotted lines, $\lambda_{exc}$ = 550 nm) of [Cr($L^{Pyr}$)$_3$] in deaerated THF (red), toluene (green) and cyclohexane (blue) solutions, respectively, at 20 °C. The absorption spectrum of $L^{Pyr}$ (toluene) is represented in black.

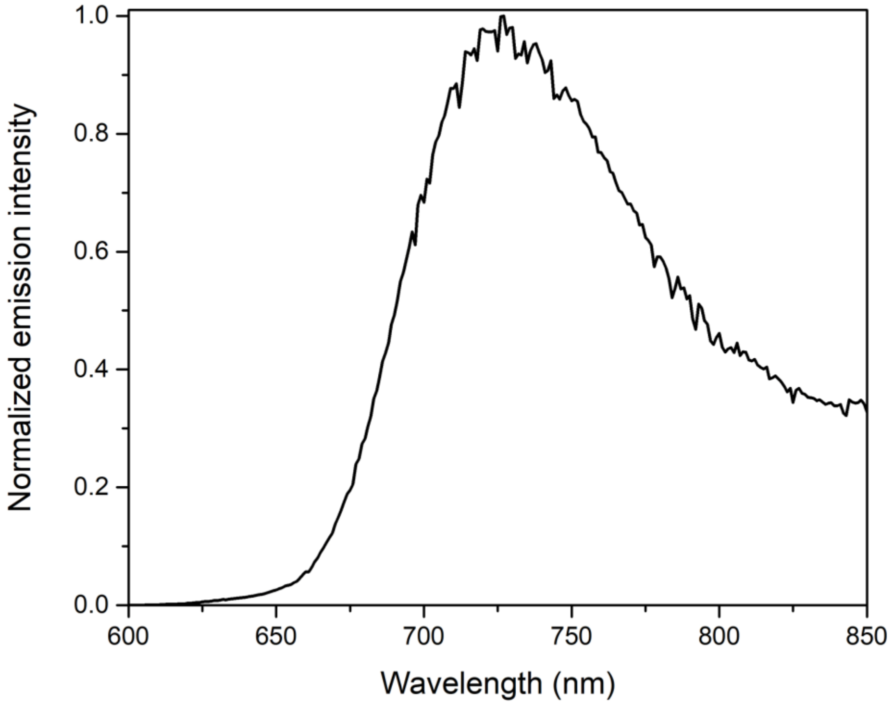


**Supplementary Fig. 92.** Emission spectrum ($\lambda_{exc}$ = 550 nm) of [Cr($L^{Pyr}$)$_3$] in 2-MeTHF at 77 K. The emission maximum is found at 727 nm.

## 3.9. Excitation spectra

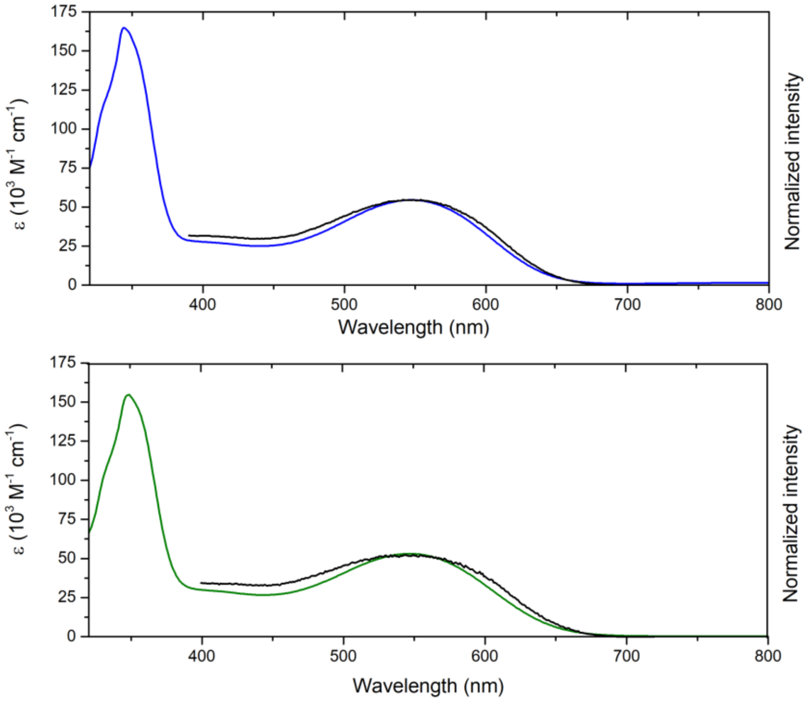


**Supplementary Fig. 93.** Excitation spectra (black, $\lambda_{det,cyclohexane}$ = 713 nm, $\lambda_{det,toluene}$ = 740 nm) and UV–Vis absorption spectra in deaerated cyclohexane (blue) and toluene (green), respectively, of $[Cr(L^{Pyr})_3]$ at 20 °C.

## 3.10. Transient absorption spectra and excited state decays (absorption and emission)

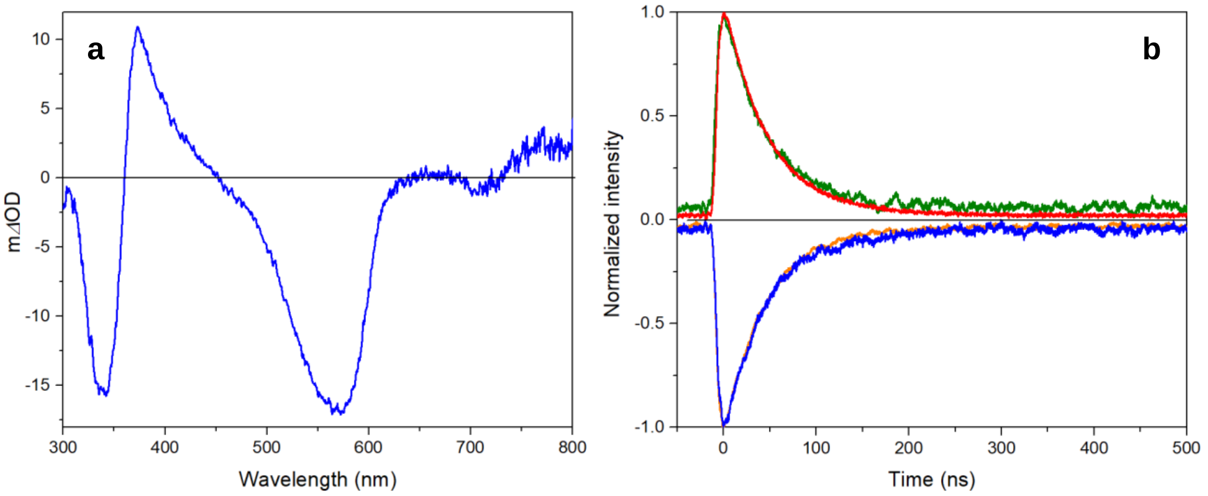


**Supplementary Fig. 94. (a)** Transient absorption spectrum of [Cr(L$^{Pyr}$)$_3$] (10 μM) in deaerated cyclohexane at 20 °C following excitation at 550 nm with laser pulses of ~10 ns. The signal was time-integrated over 50 ns immediately after excitation. **(b)** Kinetic decays obtained from the transient absorption spectrum of [Cr(L$^{Pyr}$)$_3$] in deaerated cyclohexane at 375 (excited state absorption, green line), 570 (MLCT bleach, blue line) and 340 nm (pyrene bleach, orange line), respectively, with a lifetime of 47 ns. Luminescence decay of [Cr(L$^{Pyr}$)$_3$] in deaerated cyclohexane at 713 nm (red line).

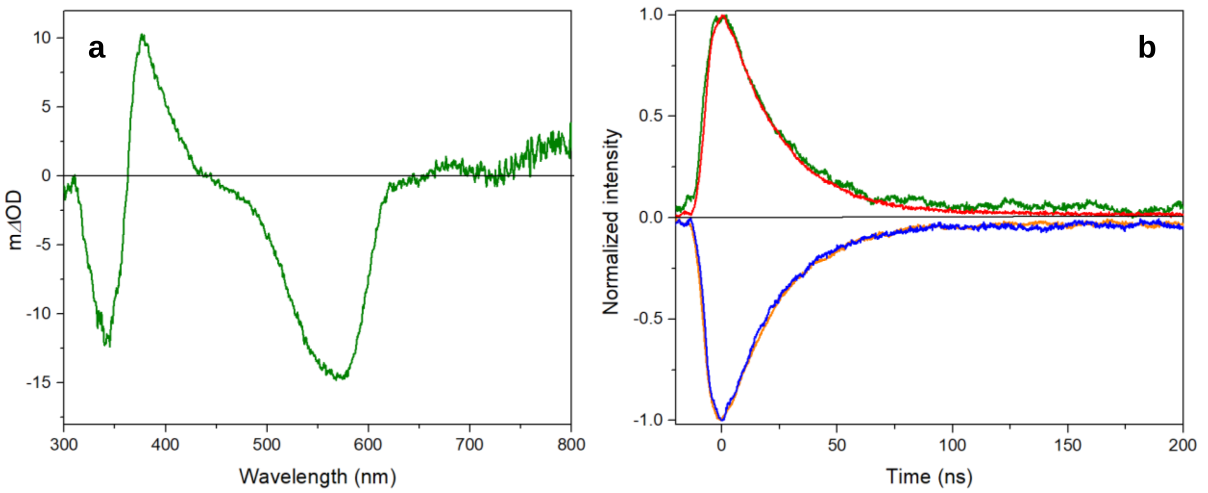


**Supplementary Fig. 95. (a)** Transient absorption spectrum of [Cr(L$^{Pyr}$)$_3$] (10 μM) in deaerated toluene at 20 °C following excitation at 550 nm with laser pulses of ~10 ns. The signal was time-integrated over 50 ns immediately after excitation. **(b)** Kinetic decays obtained from the transient absorption spectrum of [Cr(L$^{Pyr}$)$_3$] in deaerated toluene at 380 (excited state absorption, green line), 565 (MLCT bleach, blue line) and 340 nm (pyrene bleach, orange line), respectively, with a lifetime of 24 ns. Luminescence decay of [Cr(L$^{Pyr}$)$_3$] in deaerated toluene at 744 nm (red line).

## 3.11. Luminescence quantum yield determination

**Supplementary Table 7. Raw data for quantum yield determination: [Cr(L$^{Pyr}$)$_3$] in deaerated cyclohexane solution**

| UV-Vis absorption spectra | Emission spectra | Linear correlation |
|---|---|---|
| 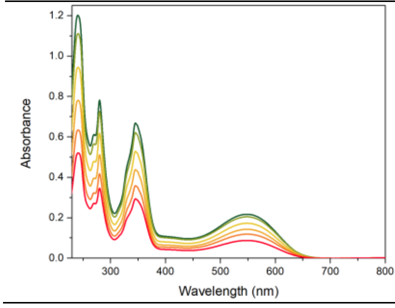 | 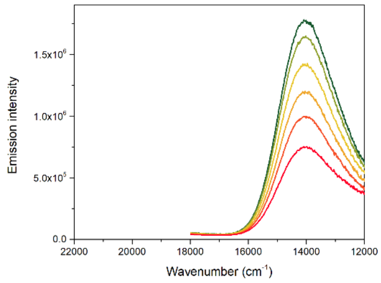 | 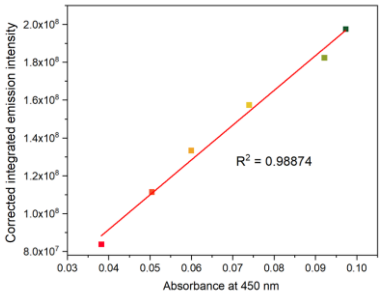 |



**Supplementary Table 8. Raw data for quantum yield determination: [Cr(L$^{Pyr}$)$_3$] in deaerated toluene solution**

| UV-Vis absorption spectra | Emission spectra | Linear correlation |
|---|---|---|
| 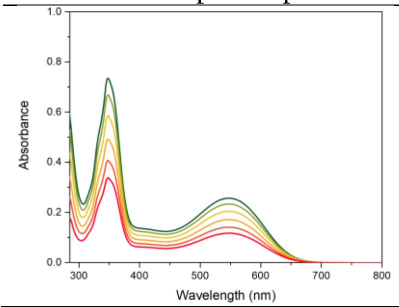 | 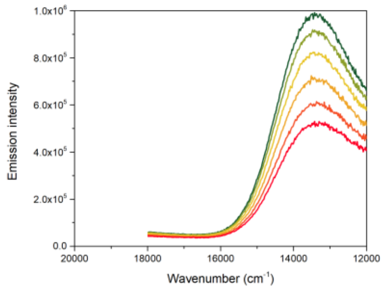 | 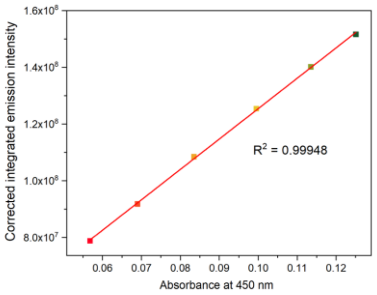 |



**Supplementary Table 9. Raw data for quantum yield determination: [Ru(bpy)$_3$]$^{2+}$ in aerated acetonitrile solution**

| UV-Vis absorption spectra | Emission spectra | Linear correlation |
|---|---|---|
| 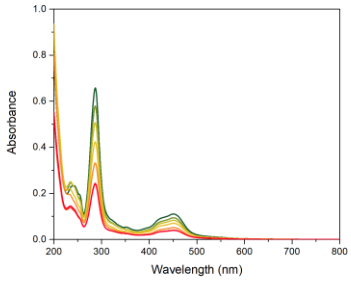 | 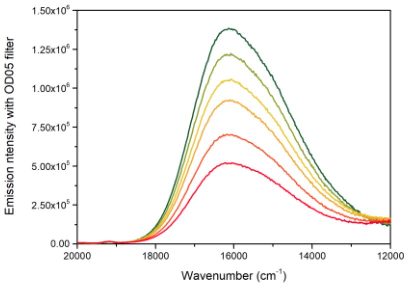 | 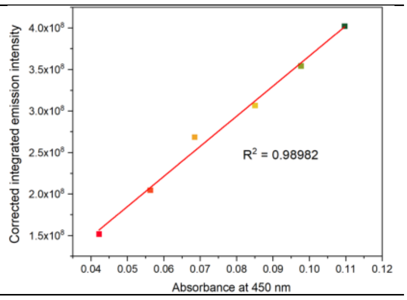 |



The luminescence quantum yield of [Cr(L$^{Pyr}$)$_3$] in THF was not determined for two main reasons: (1) The employed Fluorolog 322 instrument has a detection limit of 900 nm, making a reliable determination of the luminescence quantum yields in THF challenging; (2) an excitation wavelength of 450 nm needs to be used for relative determination to [Ru(bpy)$_3$]$^{2+}$, and as a consequence the second order diffraction at the monochromator grating leads to an intense signal at 900 nm, which is overlapping with the emission at 840 nm of [Cr(L$^{Pyr}$)$_3$] in THF. However, from a quantitative estimation it can be stated that the luminescence quantum yield of [Cr(L$^{Pyr}$)$_3$] in THF is significantly lower than in the other two investigated solvents.

**Supplementary Table 10**. Key photophysical data for [Cr($L^{Pyr}$)$_3$] in solution at 20 °C

| | solvent | $\lambda_{abs}$ (MLCT) (nm) | $\lambda_{em, max}$ (MLCT) (nm) | $\tau$ (ns) | $\phi$ (%) |
|---|---|---|---|---|---|
| **[Cr($L^{Pyr}$)$_3$]** | cyclohexane | 450 - 700 | 713 | 47 | 1.04 ± 0.05 |
| | toluene | 450 - 700 | 744 | 24 | 0.66 ± 0.006 |
| | tetrahydrofuran | 450 - 700 | 840 | 1.1 | - |

### 3.12. $^3$MLCT luminescence decay at different temperatures

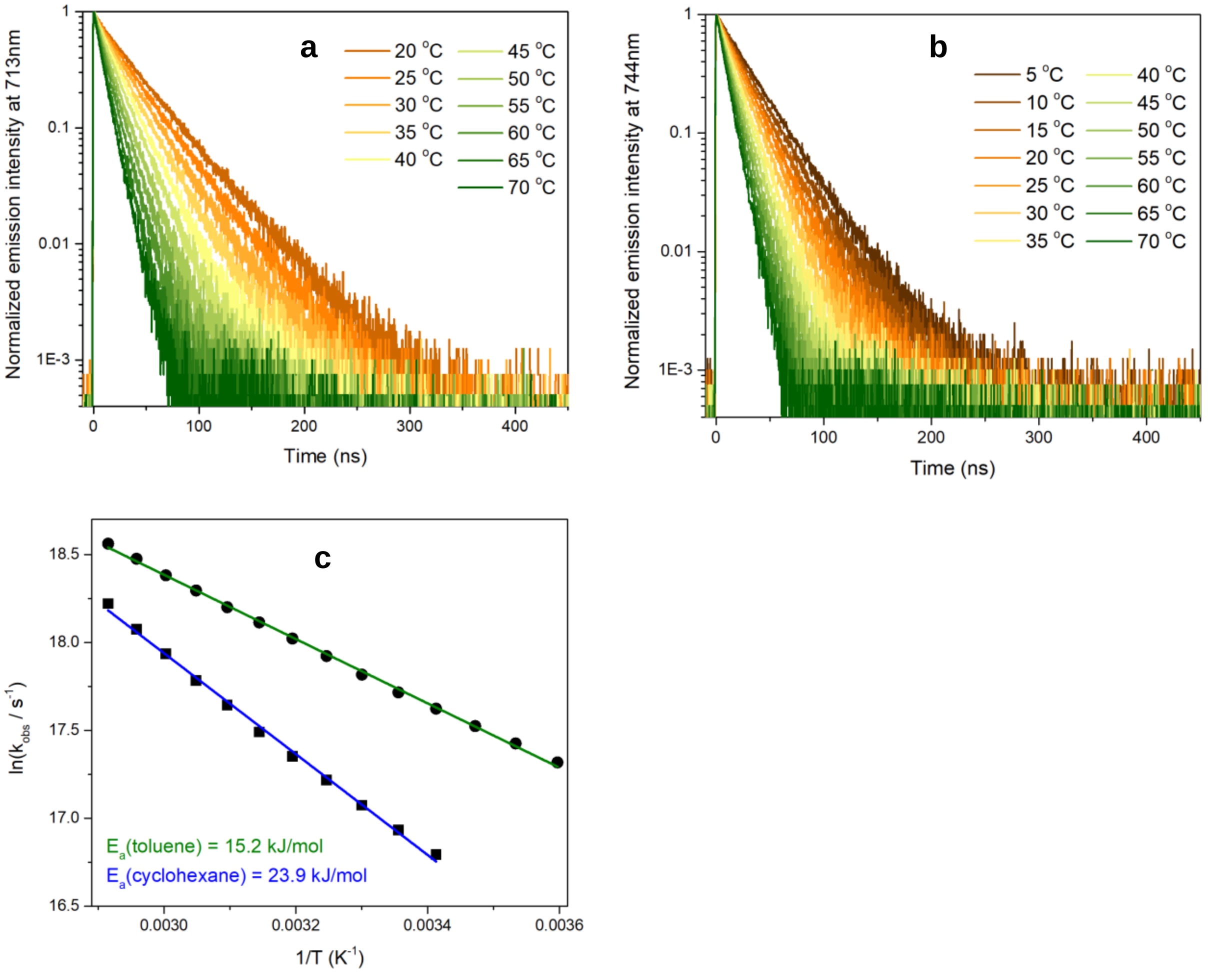


**Supplementary Fig. 96.** Temperature-dependent luminescence decay of [Cr($L^{Pyr}$)$_3$] (10 μM) in deaerated solutions of (**a**) cyclohexane and (**b**) toluene. Excitation occurred at 635 nm, detection was at 713 and 744 nm, respectively. (**c**) Arrhenius analysis of the datasets in (**a**) and (**b**).

### 3.13. Chemical oxidation of [Cr($L^{Pyr}$)$_3$] in solution

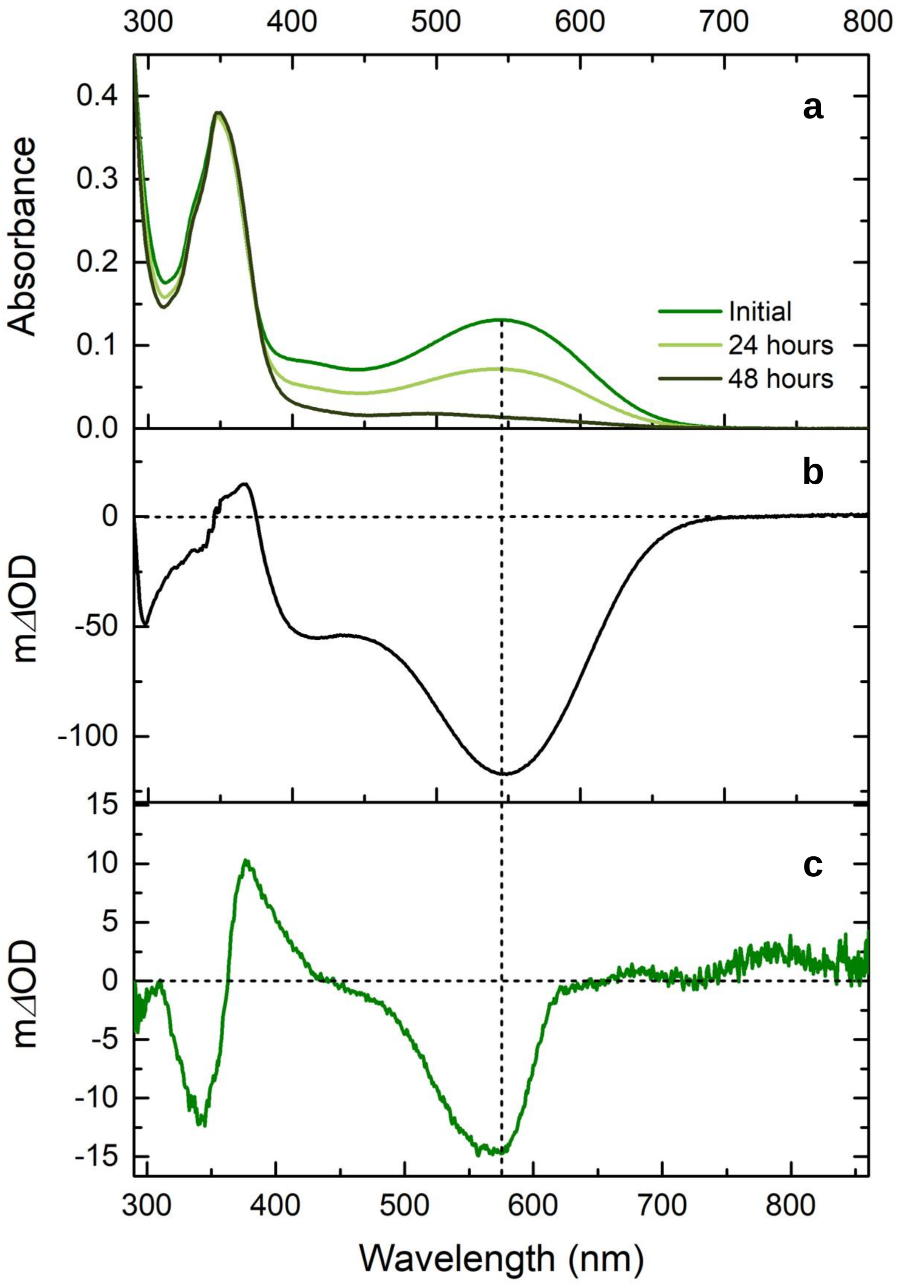


**Supplementary Fig. 97.** (**a**) UV–Vis absorption spectra of [Cr($L^{Pyr}$)$_3$] in an aerated solution of toluene at 20 °C. The spectra were recorded on the same sample at different time intervals as indicated in the figure. (**b**) Difference between the UV–Vis absorption spectrum recorded initially and that recorded after 48 hours. (**c**) Transient absorption spectrum of [Cr($L^{Pyr}$)$_3$] (10 μM) in deaerated toluene at 20 °C following excitation at 550 nm with laser pulses of ~10 ns. The signal was time-integrated over 50 ns immediately after excitation.

### 3.14. Triplet-triplet energy transfer to perylene

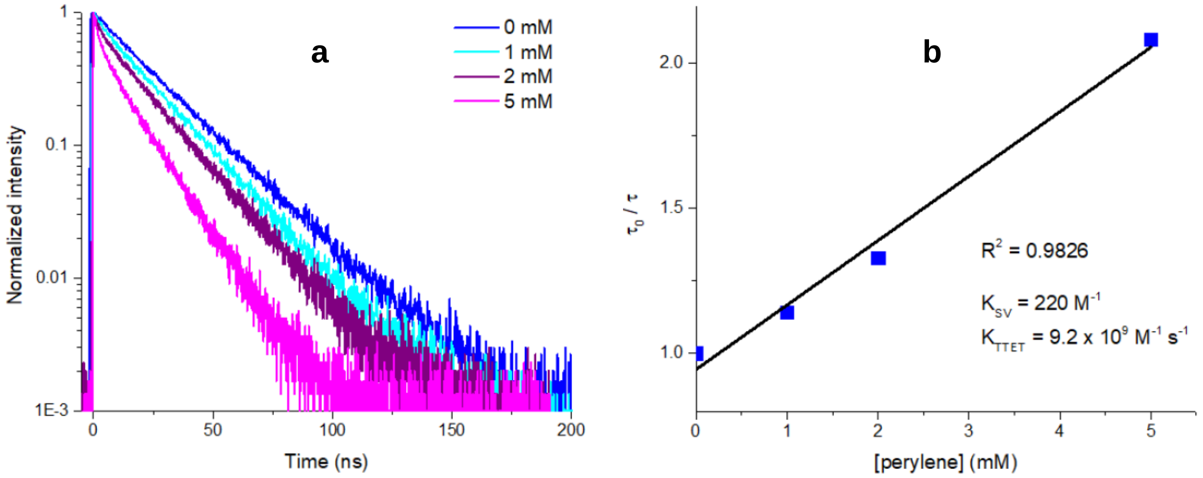


**Supplementary Fig. 98. (a)** $^3$MLCT luminescence quenching of [Cr($L^{Pyr}$)$_3$] (10 μM) by perylene in deaerated toluene at 20 °C. Excitation occurred at 635 nm in the presence of different concentrations of perylene (0, 1, 2, 5 mM, see color coding in figure). **(b)** Stern-Volmer analysis of the data from (**a**).

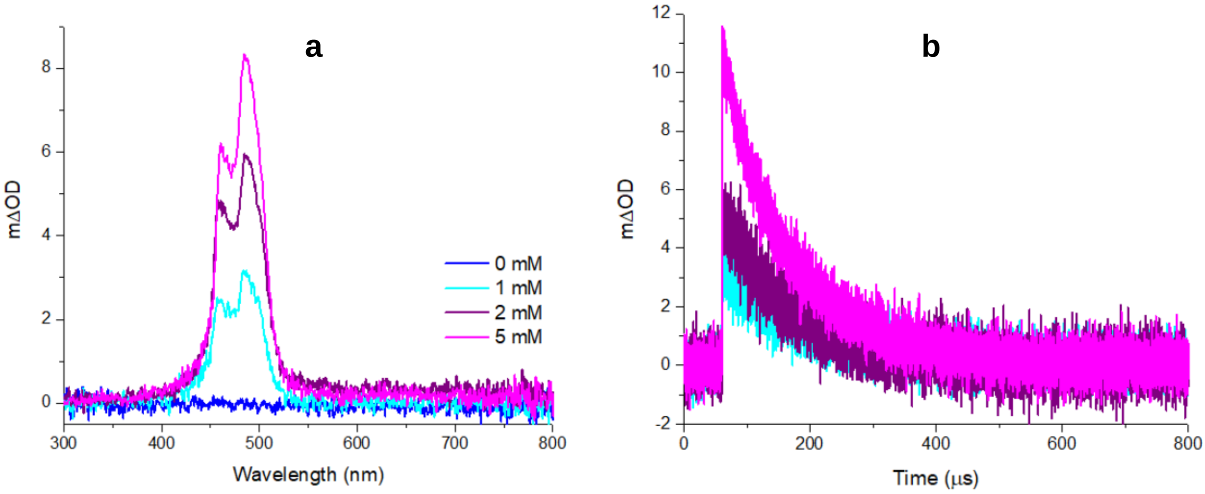


**Supplementary Fig. 99. (a)** Transient absorption spectra of $^3$perylene. The spectra were recorded in deaerated toluene, after complete decay of the $^3$MLCT luminescence emitted by [Cr($L^{Pyr}$)$_3$] (10 μM) at 744 nm. The concentrations of perylene and their respective color codes are specified in the insert. All spectra were recorded with an integration time of 200 μs, and the following delay times were used: 350 ns (0 mM), 300 ns (1 mM), 250 ns (2 mM) and 150 ns (5 mM). Excitation occurred at 635 nm. **(b)** Decay traces of $^3$perylene monitored at 488 nm for the spectra shown in (**a**).

## 4. Photostability of $Cr^0$ complexes

### 4.1. Photostability of [Cr($L^{Mes}$)$_3$] and [Cr($L^{Pyr}$)$_3$] in comparison to [Ru(bpy)$_3$]$^{2+}$

The photostability studies were performed with a green continuous wave (CW) laser with excitation at 532nm (25mW). Deaerated acetonitrile solution of [Ru(bpy)$_3$]$^{2+}$ and deaerated cyclohexane solutions of [Cr($L^{Mes}$)$_3$] and [Cr($L^{Pyr}$)$_3$] with optical density around 0.10 – 0.13 were irradiated with 25 mW 532 nm CW laser and the corresponding emission intensities were monitored at 620, 695, and 713 nm, respectively. Both $Cr^0$ complexes are less photostable when compared to [Ru(bpy)$_3$]$^{2+}$.

**Supplementary Table 11. [Ru(bpy)$_3$]$^{2+}$ in acetonitrile**

| UV-Vis absorption spectra | Emission spectra | Decay of emission |
|---|---|---|
| 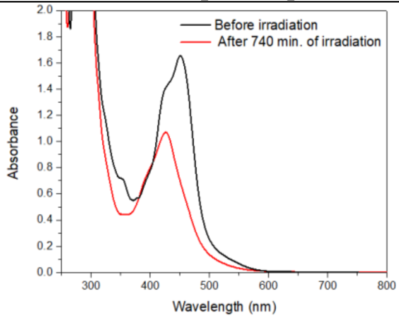 | 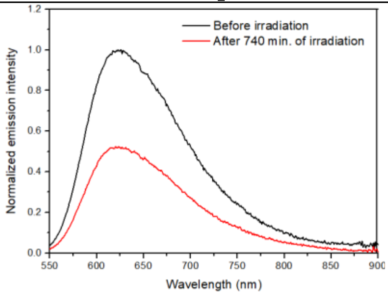 | 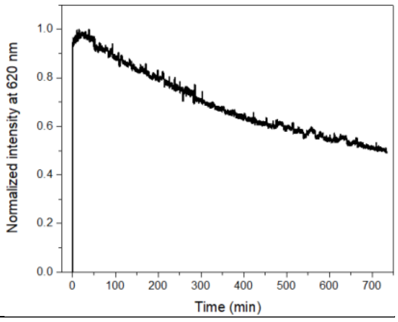 |



**Supplementary Table 12. [Cr($L^{Mes}$)$_3$] in cyclohexane**

| UV-Vis Absorption spectra | Emission spectra | Decay of emission |
|---|---|---|
| 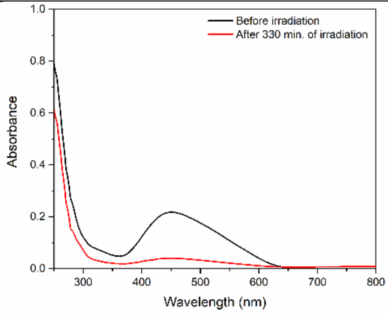 | 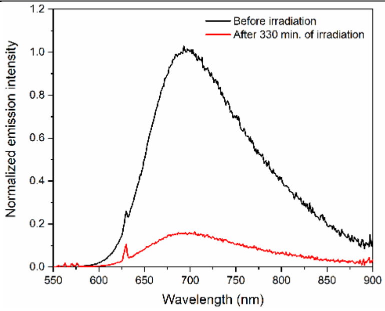 | 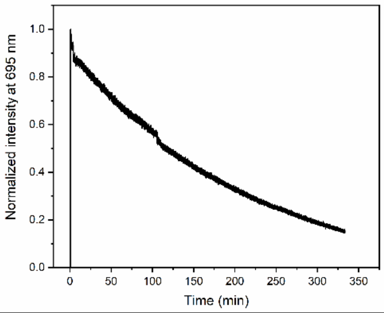 |



**Supplementary Table 13. [Cr($L^{Pyr}$)$_3$] in cyclohexane**

| UV-Vis absorption spectra | Emission spectra | Decay of emission |
|---|---|---|
| 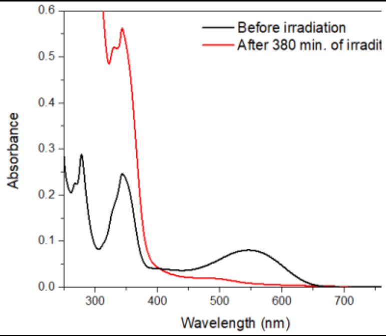 | 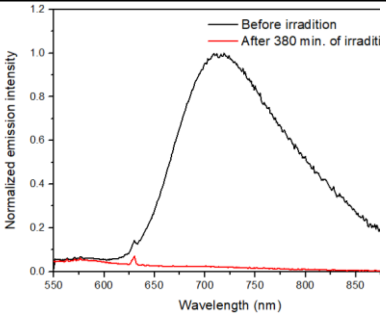 | 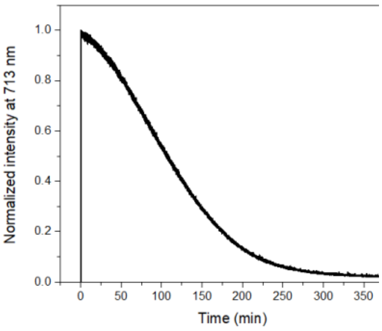 |

**4.2. Photostability study of [Cr($L^{Mes}$)$_3$] under photoredox catalysis condition with 623 nm (3.8 W) LED**

3 mL dry and degassed benzene was added to a mixture of 4-bromo-3-methylbenzonitrile (5.9 mg, 0.03 mmol, 1 equiv.), TDAE (10.0 µL, 0.045 mmol, 1.5 equiv.) and [Cr($L^{Mes}$)$_3$] (0.6 mg, 0.3 µmol, 0.01 equiv.) in a Schlenk tube under inert conditions. Then the resulting mixture was degassed by freeze-pump-thaw (3 times) cycles, and transferred to a 1 cm Schlenk cuvette under inert atmosphere. The reaction mixture was irradiated with 623 nm (3.8 W) LED (Thorlab, Solis-623), and the MLCT absorption band of the photosensitizer ([Cr($L^{Mes}$)$_3$]) was monitored at different time intervals to study the photostability of the new photosensitizer during the reductive dehalogenation reaction. Due to the formation of [TDAE-$H^+$]$^+$$Br^-$ salt, the baselines of the absorption spectra were usually in the range of 0.1-0.4 OD, thus they were manually set to 0 OD at 800 nm. The data in Supplementary Fig. 100 indicates that the photosensitizer ([Cr($L^{Mes}$)$_3$]) is largely stable under these conditions, and over 12 h of irradiation about 60% photocatalyst decomposed. The irradiation times used for the photochemical reactions in the main paper were between 1 and 5 hours, and 1 – 3 mol% catalyst was used. Only 1 mol% catalyst was used for this photostability study.

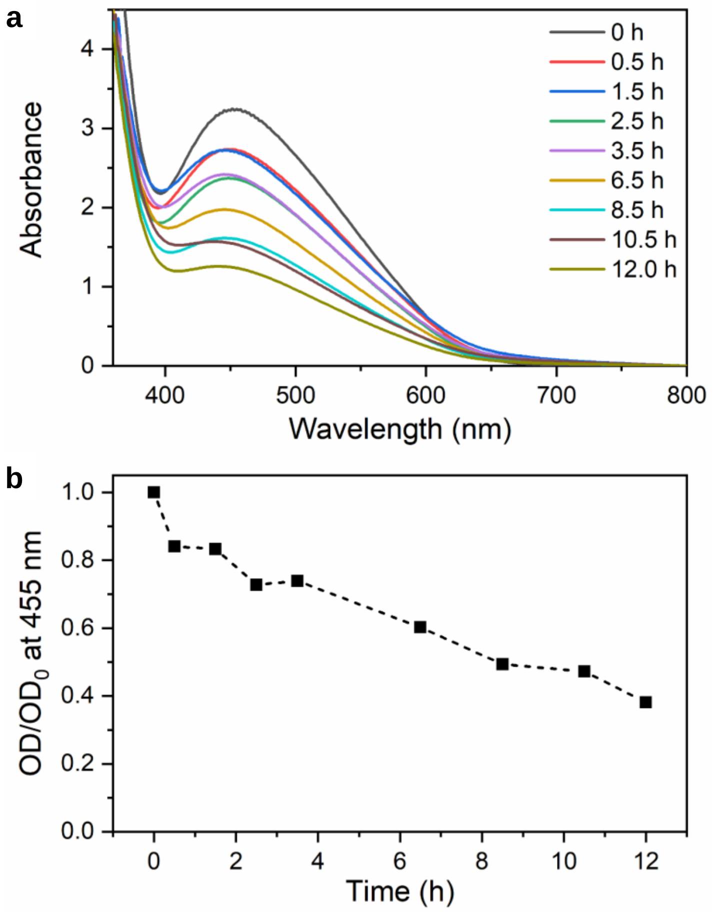


**Supplementary Fig. 100.** (**a**) UV-Vis absorption spectra of the reaction mixture described in the above paragraph upon continuous photoirradiation with a 3.8 W 623 nm LED after different time intervals. (**b**) Absorbance of the reaction mixture at 455 nm as a function of time upon continuous photoirradiation.

## 5. Additional spectroscopic data

### 5.1. Steady-state emission spectra of [Cr($L^{tBu}$)$_3$], [Cr($L^{Mes}$)$_3$] and [Cr($L^{Pyr}$)$_3$]

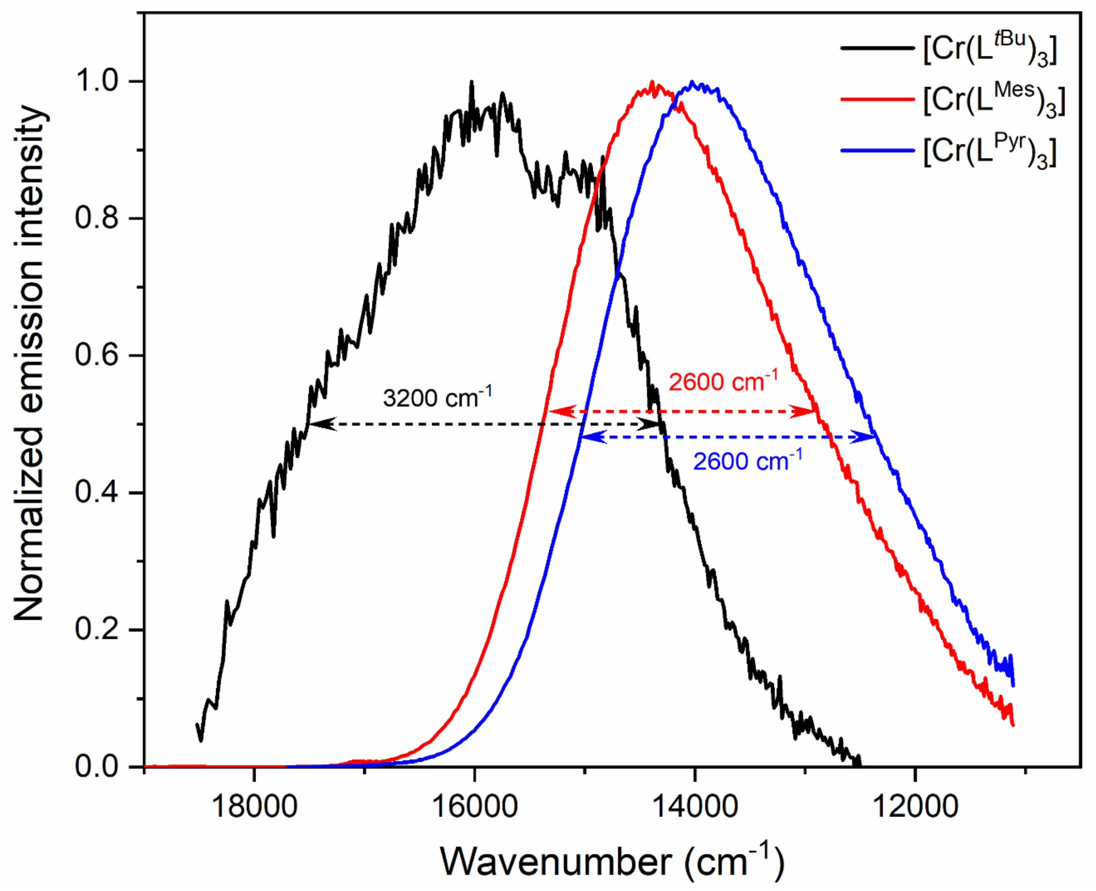


**Supplementary Fig. 101.** Normalized steady-state emission spectra at 20°C of [Cr($L^{tBu}$)$_3$] (THF), [Cr($L^{Mes}$)$_3$] (cyclohexane), [Cr($L^{Pyr}$)$_3$] (cyclohexane) in deaerated solutions. Excitation occurred at 450, 500 and 550 nm, respectively. The full widths at half maxima (fwhm, dashed horizontal lines) for the three emission bands are indicated, and these fwhm values can be interpreted as a measure for the distortion of the MLCT excited state relative to the electronic ground state. For each $Cr^0$ complex, the solvent in which the individual emitters performed best is considered here.

## 6. Red light driven photoredox catalysis using [Cr($L^{Mes}$)$_3$] as a photosensitizer

### 6.1. UV-Vis absorption spectra of a representative substrate, electron donor and photosensitizer, and emission profile of 623 nm LED

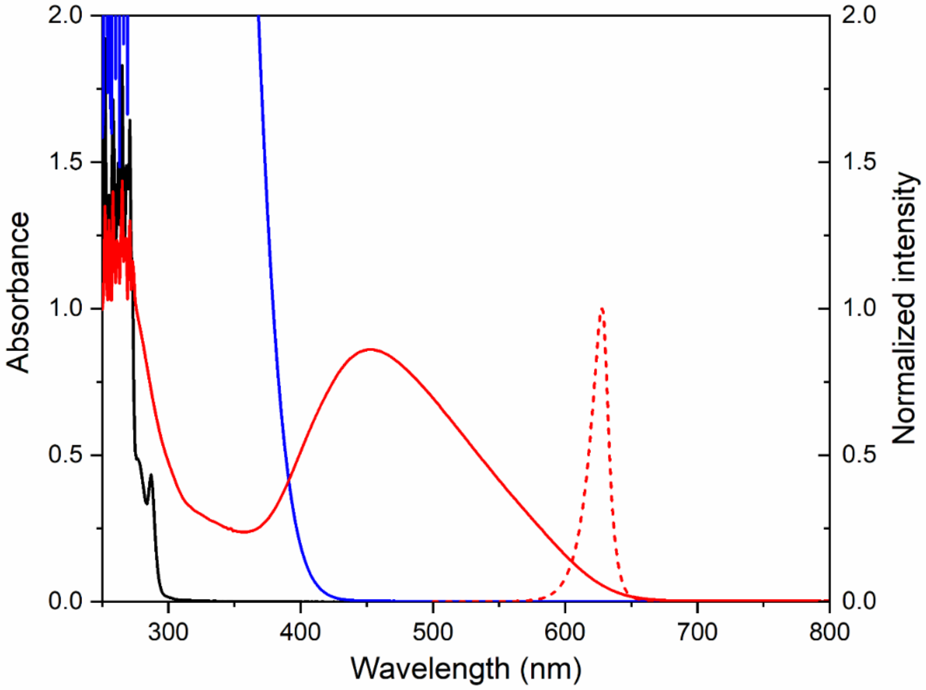


**Supplementary Fig. 102.** UV–Vis absorption spectra (solid traces) of 3-methyl-4-bromobenzonitrile (0.41 mM, black trace), tetrakis(dimethylamino)ethylene (TDAE, blue trace, 7.16 mM) and [Cr($L^{Mes}$)$_3$] (red trace, 20.63 µM) in benzene. Emission spectrum (red dotted trace) of the 623 nm LED used in this study.

### 6.2. Image of the photoredox reaction set up

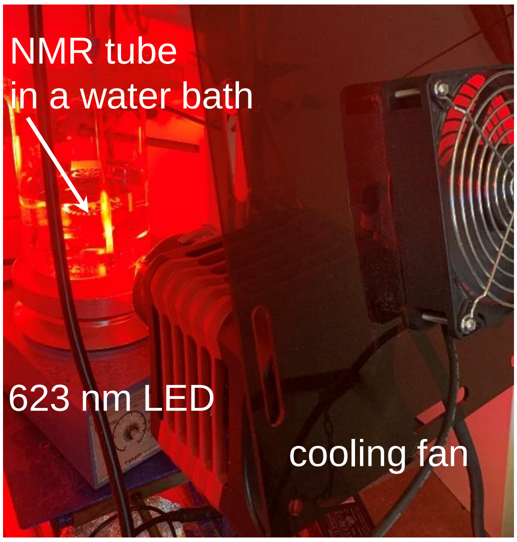


**Supplementary Fig. 103.** Irradiation setup for photoredox reactions with 623 nm LED.

## 6.3. Stern-Volmer quenching study

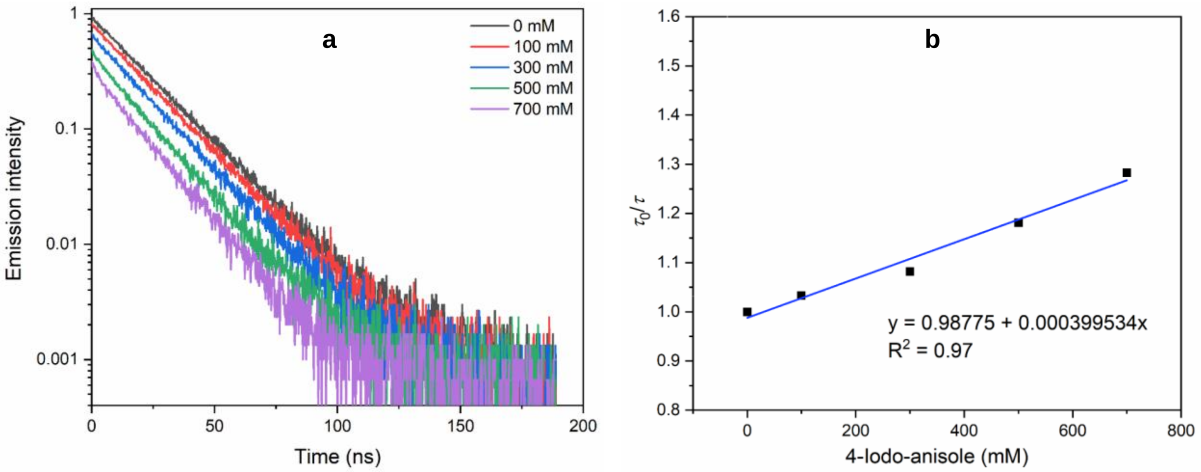


**Supplementary Fig. 104.** (**a**) Luminescence lifetime quenching of 20 $\mu$M [Cr($L^{Mes}$)$_3$] by 4-iodo-anisole (0, 100, 300, 500, 700 mM, respectively) in deaerated toluene at 20 °C. (**b**) Plot of ($\tau_0/\tau$) versus 4-iodo-anisole concentration for [Cr($L^{Mes}$)$_3$]. From the slope of the linear fit, the bimolecular quenching constant was determined to be $k_q = 1.9 \times 10^7$ $M^{-1}s^{-1}$ using $\tau_0 = 20.11$ ns. The initial luminescence intensity at t = 0 ns in (**a**) decreases with increasing 4-iodo-anisole concentration, which is indicative of some static quenching in addition to the dynamic quenching evaluated in (**b**).

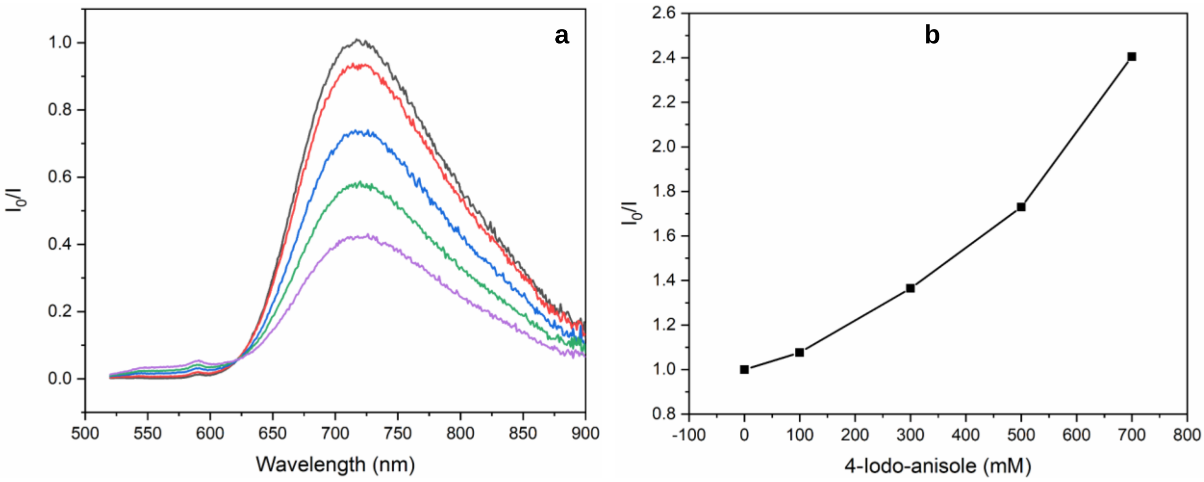


**Supplementary Fig. 105.** (**a**) Steady-state luminescence quenching of 20 $\mu$M [Cr($L^{Mes}$)$_3$] by 4-iodo-anisole (0, 100, 300, 500, 700 mM, respectively) in deaerated toluene at 20 °C. (**b**) Plot of ($I_0$/I) versus 4-iodo-anisole concentration for [Cr($L^{Mes}$)$_3$]. The data in panel B confirms the simultaneous presence of both dynamic and static quenching. Similar observations were recently made for a $^2$LMCT emissive iron(III) photosensitizer[12].

## 6.4. Red-light induced hydrodehalogenation reactions

A Schlenk flask was charged with aryl halide (0.06 mmol) and chromium(0) photosensitizer (1-3 mol%). Then the flask was evacuated and back filled with argon/nitrogen (3 times). Tetrakis(dimethylamino)ethylene (TDAE, 1.5 equiv), dry and deaerated $C_6D_6$ or THF-$d_8$ (0.6 mL) and 1,4-dioxane (internal standard, 0.06 mmol) were added subsequently. Then the reaction mixture was transferred to an NMR tube under inert gas conditions and sealed. The

NMR tube was irradiated with a 623 nm LED (Thorlabs Solis-623C, 3.8 W, with 550 nm long-pass filter). Yields were determined by NMR spectroscopy against the internal standard.

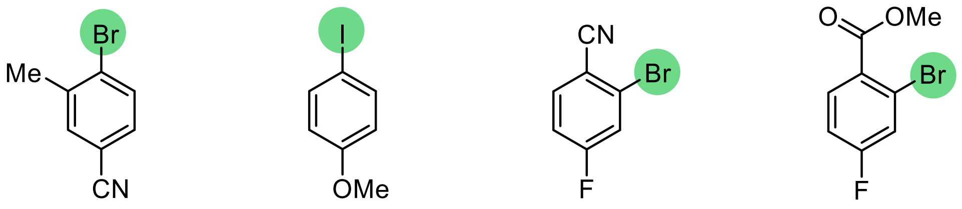


**1** -2.51 V vs $Fc^{+/0}$ | **2** -2.59 V vs $Fc^{+/0}$ | **3** -2.53 V vs $Fc^{+/0}$ | **4** -2.53 V vs $Fc^{+/0}$

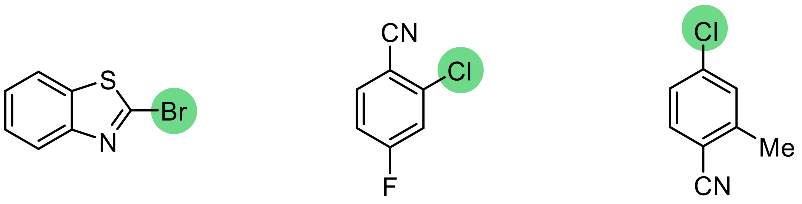


**5** -2.52 V vs $Fc^{+/0}$ | **6** -2.50 V vs $Fc^{+/0}$ | **7** -2.63 V vs $Fc^{+/0}$

**Supplementary Fig. 106.** Reduction potentials of substrates used in this study. Potentials for compounds **2** – **6** have been reported previously. Potentials reported in V vs SCE were converted to V vs $Fc^{+/0}$ by subtracting 0.38 V[13,14].

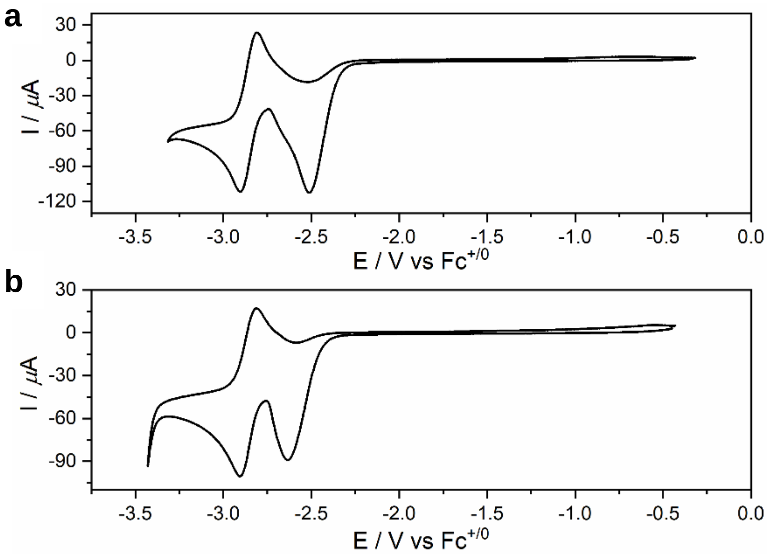


**Supplementary Fig. 107.** Cyclic voltammograms of 2 mM substrates **1** and **7** (see Supplementary Fig. 106), respectively, in acetonitrile with 0.1 M of $(^{n}Bu_4N)(PF_6)$ as supporting electrolyte, recorded at a scan rate of 0.1 V/s.

## 6.5. NMR spectra for the red-light driven hydrodehalogenation reactions

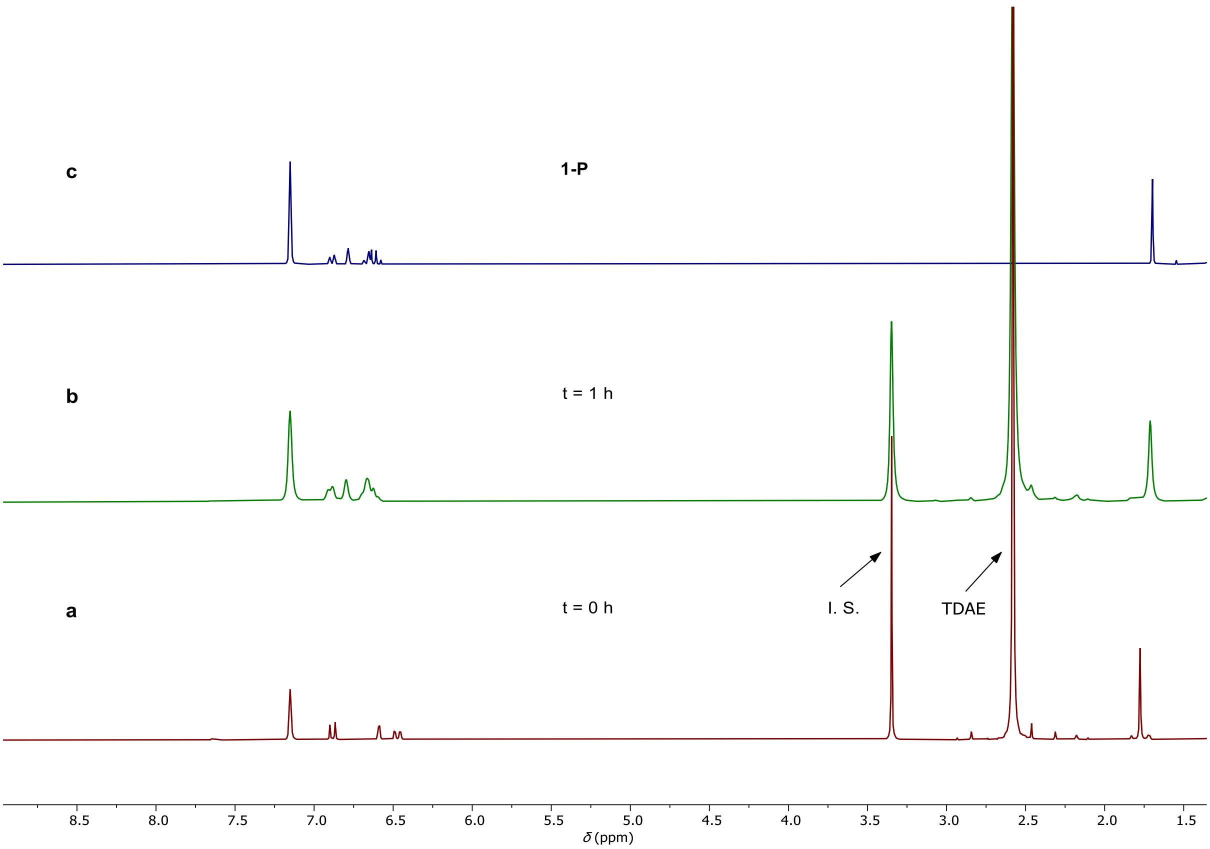


**Supplementary Fig. 108.** $^1$H NMR spectra monitoring the red-light driven dehalogenation of substrate **1** to product **1-P**. (**a**) $^1$H NMR spectrum of the reaction mixture containing substrate **1** (4-bromo-3-methyl-benzonitrile), [Cr(L$^{Mes}$)$_3$], TDAE, 1,4-dioxane (internal standard, I. S.) in $C_6D_6$. (**b**) $^1$H NMR spectrum of the reaction mixture after 1 h photoirradiation with 623 nm LED. (**c**) $^1$H NMR spectrum of the commercially available product **1-P** in $C_6D_6$. The NMR yield of **1-P** from (**b**) is 99%.

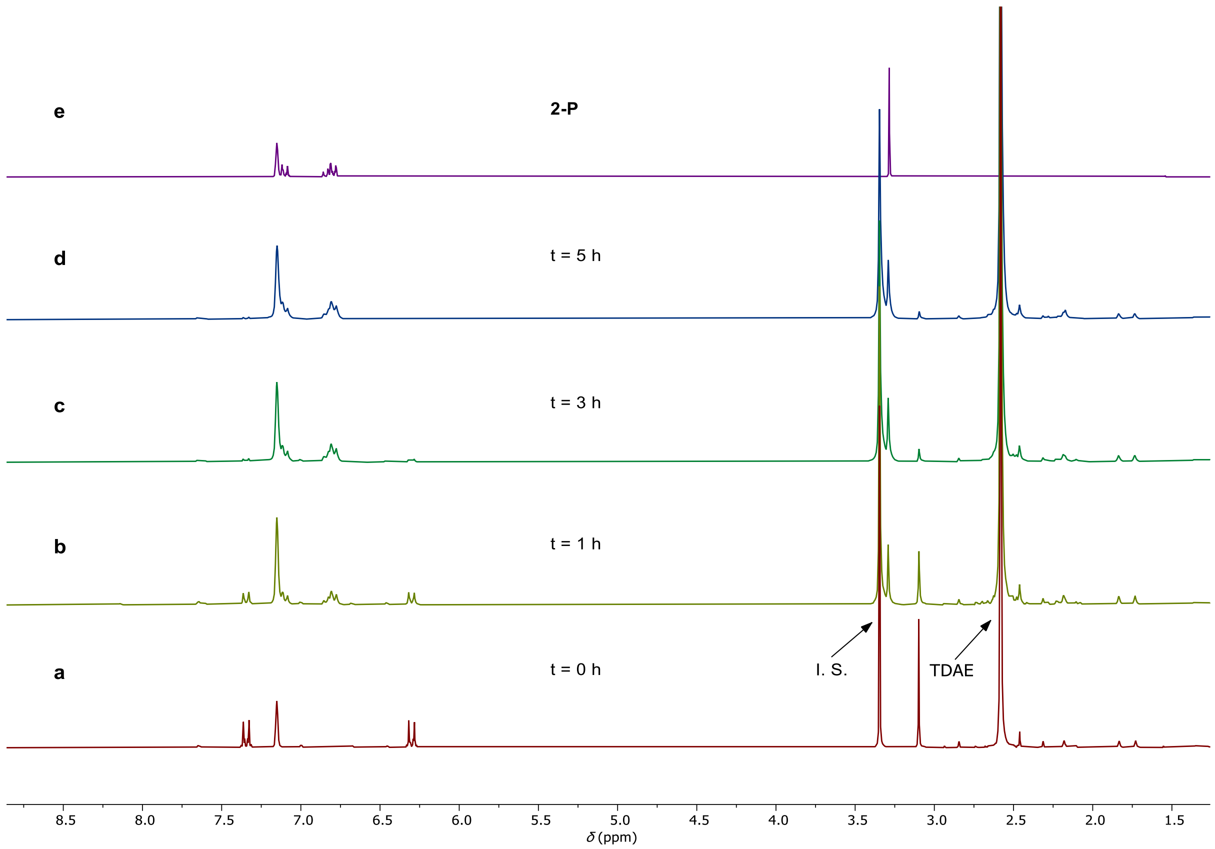


**Supplementary Fig. 109.** $^1$H NMR spectra monitoring the red-light driven dehalogenation of substrate **2** to product **2-P**. (**a**) $^1$H NMR spectrum of the reaction mixture containing substrate **2** (4-bromo-anisole), [Cr(L$^{Mes}$)$_3$], TDAE, 1,4-dioxane (internal standard, I. S.) in $C_6D_6$. $^1$H NMR spectrum of the reaction mixture after 1 h (**b**), 3 h (**c**) and 5 h (**d**) photoirradiation with 623 nm LED, respectively. (**e**) $^1$H NMR spectrum of the commercially available product **2-P** in $C_6D_6$. The NMR yield of **2-P** from (**d**) is 83%.

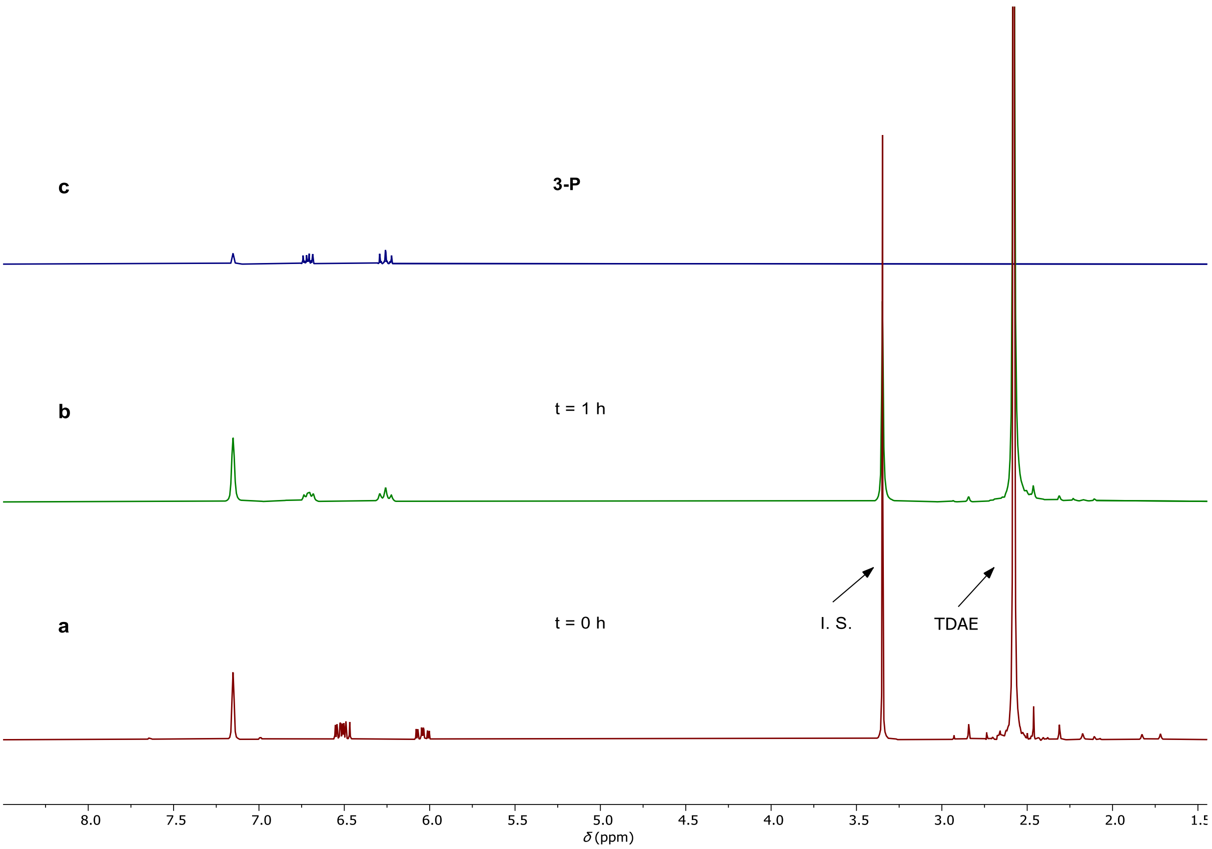


**Supplementary Fig. 110.** $^1$H NMR spectra monitoring the red-light driven dehalogenation of substrate **3** to product **3-P**. (**a**) $^1$H NMR spectrum of the reaction mixture containing substrate **3** (2-bromo-4-fluoro-benzonitrile), [Cr(L$^{Mes}$)$_3$], TDAE, 1,4-dioxane (internal standard, I. S.) in $C_6D_6$. (**b**) $^1$H NMR spectrum of the reaction mixture after 1 h photoirradiation with 623 nm LED. (**c**) $^1$H NMR spectrum of the commercially available product **3-P** in $C_6D_6$. The NMR yield of **3-P** from (**b**) is 86%.

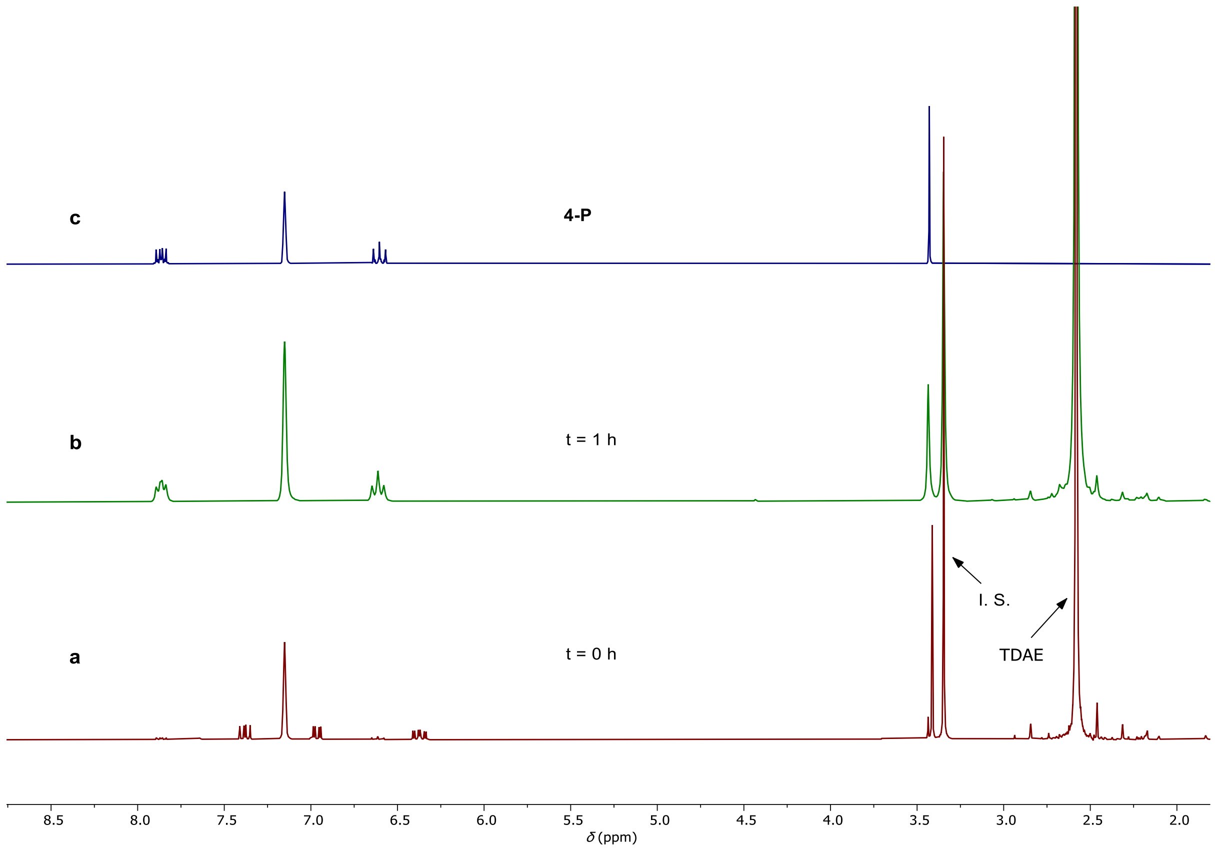


**Supplementary Fig. 111.** $^1$H NMR spectra monitoring the red-light driven dehalogenation of substrate **4** to product **4-P**. (**a**) $^1$H NMR spectrum of the reaction mixture containing substrate **4** (2-bromo-4-fluoro-methylbenzoate), [Cr(L$^{Mes}$)$_3$], TDAE, 1,4-dioxane (internal standard, I. S.) in $C_6D_6$. (**b**) $^1$H NMR spectrum of the reaction mixture after 1 h photoirradiation with 623 nm LED. (**c**) $^1$H NMR spectrum of the commercially available product **4-P** in $C_6D_6$. The NMR yield of **4-P** from (**b**) is 96%.

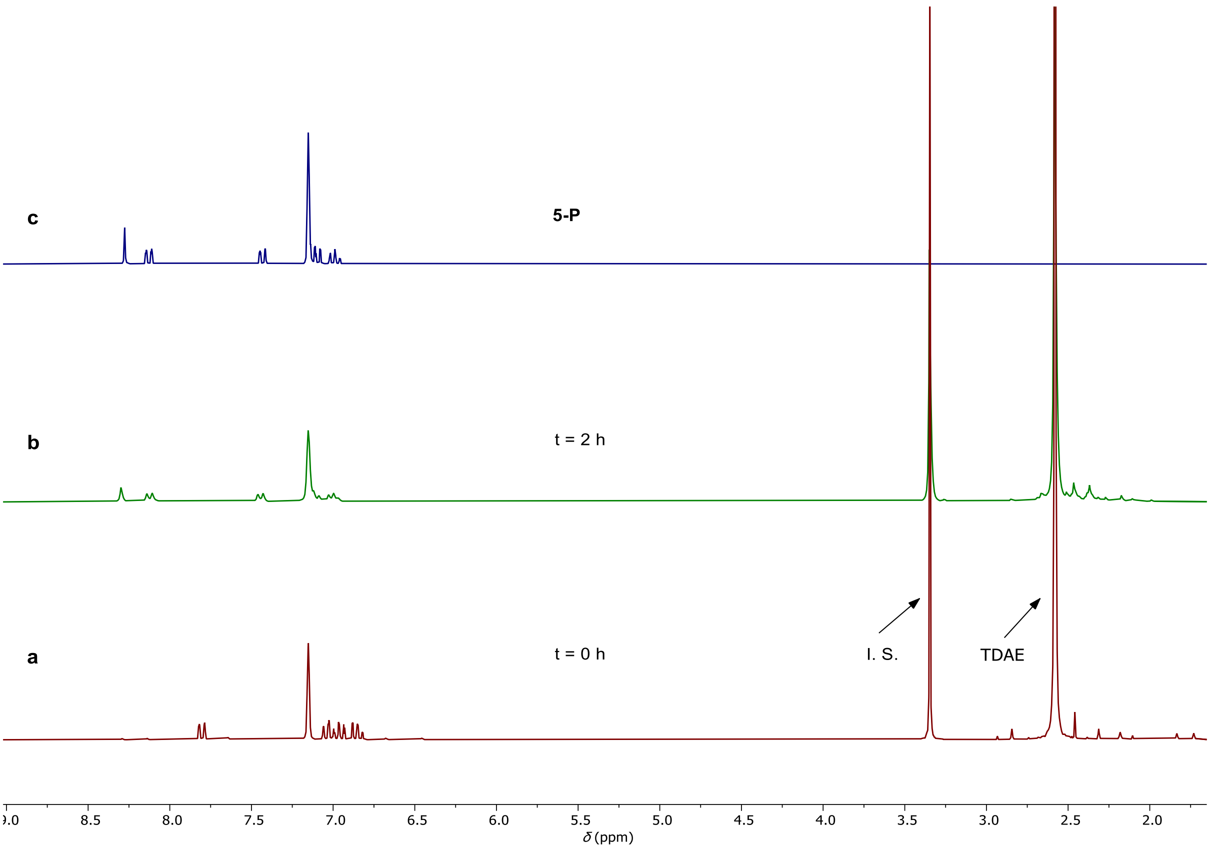


**Supplementary Fig. 112.** $^{1}$H NMR spectra monitoring the red-light driven dehalogenation of substrate **5** to product **5-P**. (**a**) $^{1}$H NMR spectrum of the reaction mixture containing substrate **5** (2-bromo-4-fluoro-methylbenzoate), [Cr(L$^{Mes}$)$_3$], TDAE, 1,4-dioxane (internal standard, I. S.) in $C_6D_6$. (**b**) $^{1}$H NMR spectrum of the reaction mixture after 2 h photoirradiation with 623 nm LED. (**c**) $^{1}$H NMR spectrum of the commercially available product **5-P** in $C_6D_6$. The NMR yield of **5-P** from (**b**) is 94%.

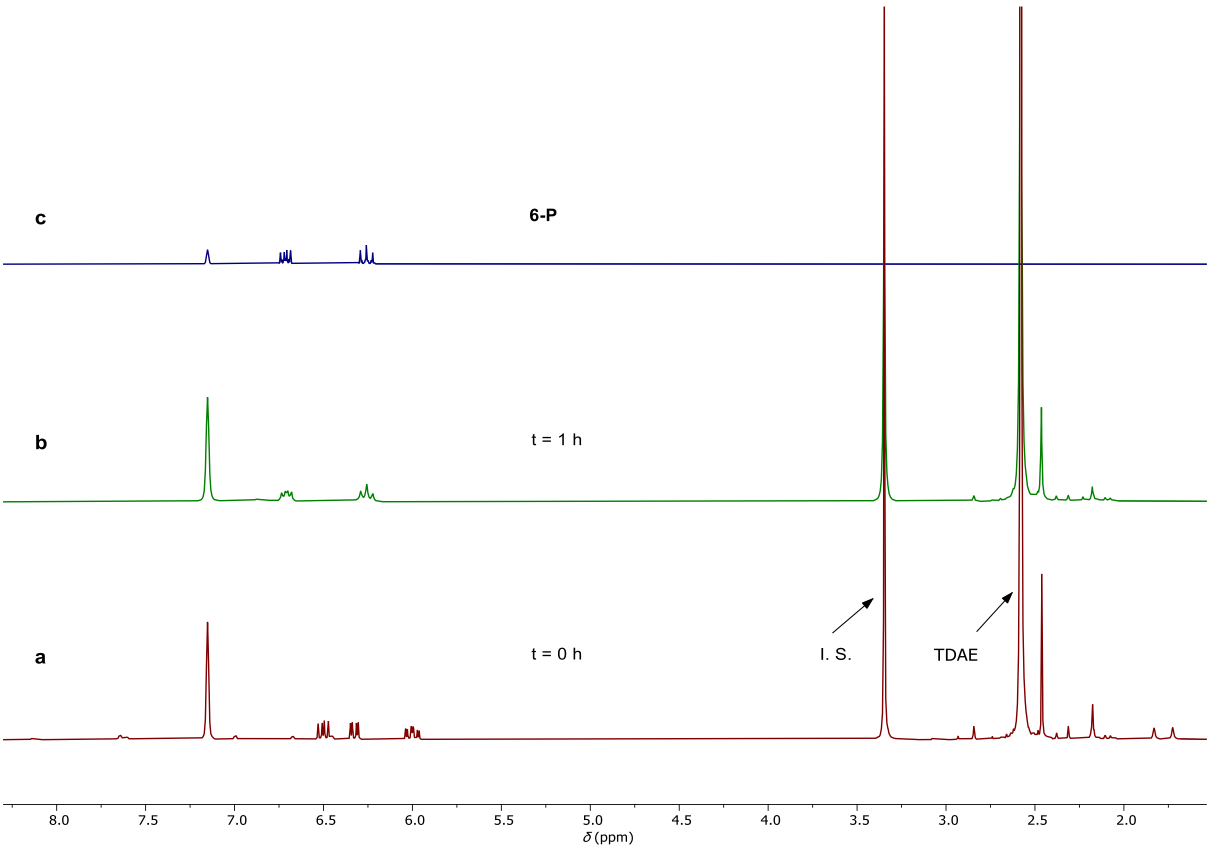


**Supplementary Fig. 113.** $^1$H NMR spectra monitoring the red-light driven dehalogenation of substrate **6** to product **6-P**. (**a**) $^1$H NMR spectrum of the reaction mixture containing substrate **6** (2-chloro-4-fluoro-benzonitrile), [Cr($L^{Mes}$)$_3$], TDAE, 1,4-dioxane (internal standard, I. S.) in $C_6D_6$. (**b**) $^1$H NMR spectrum of the reaction mixture after 1 h photoirradiation with 623 nm LED. (**c**) $^1$H NMR spectrum of the commercially available product **6-P** in $C_6D_6$. The NMR yield of **6-P** from (**b**) is 79%.

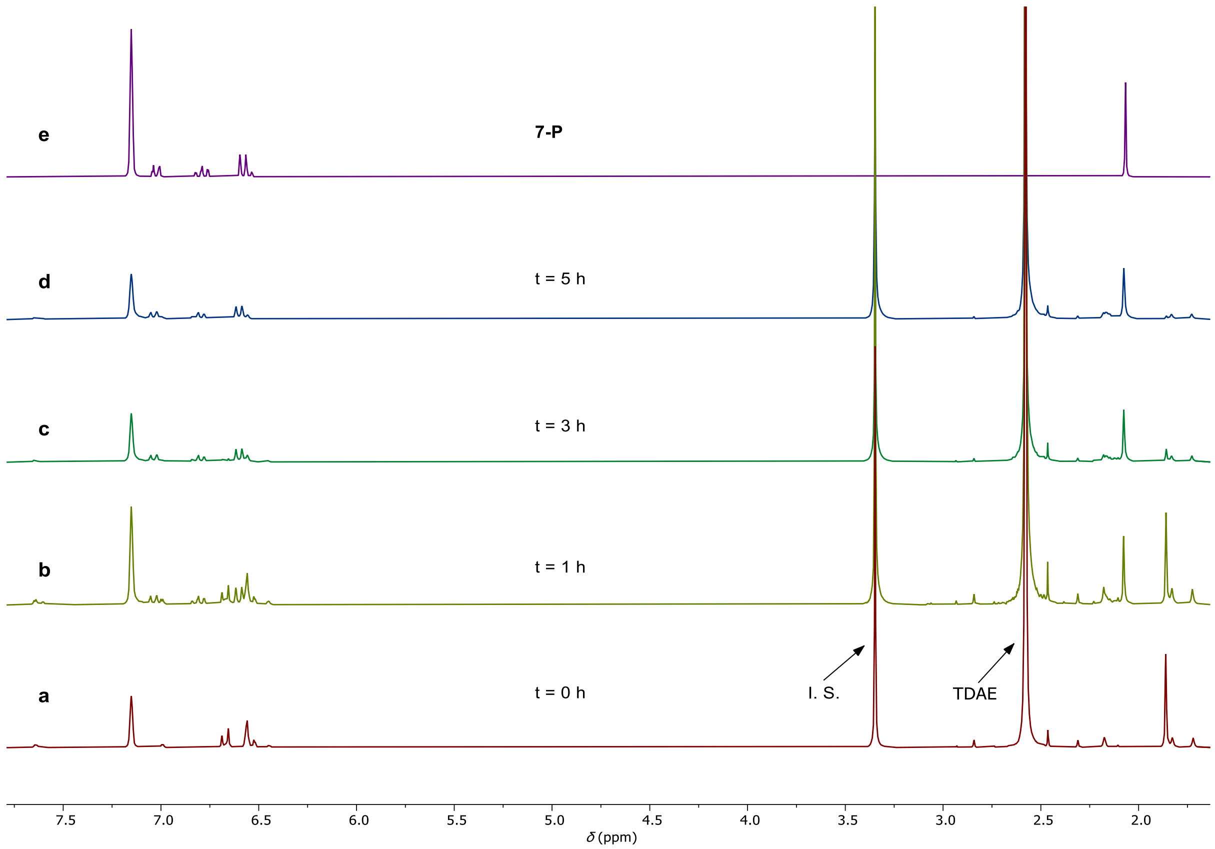


**Supplementary Fig. 114.** $^1$H NMR spectra monitoring the red light driven dehalogenation of substrate **7** to product **7-P**. (**a**) $^1$H NMR spectrum of the reaction mixture containing substrate **7** (2-methyl-4-chloro-benzonitrile), [Cr(L$^{Mes}$)$_3$], TDAE, 1,4-dioxane (internal standard, I. S.) in $C_6D_6$. $^1$H NMR spectrum of the reaction mixture after 1 h (**b**), 3 h (**c**) and 5 h (**d**) photoirradiation with 623 nm LED, respectively. (**e**) $^1$H NMR spectrum of the commercially available product **7-P** in $C_6D_6$. The NMR yield of **7-P** from (**d**) is 86%.

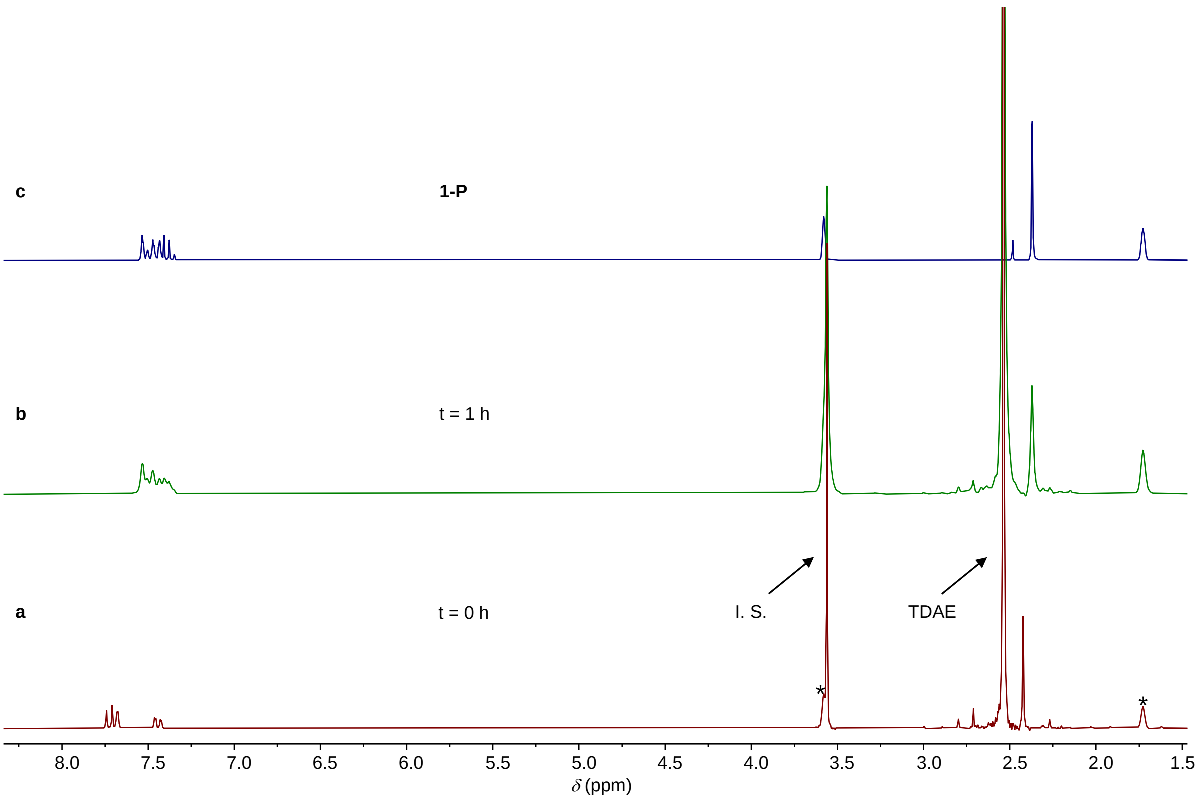


**Supplementary Fig. 115.** $^{1}H$ NMR spectra monitoring the red-light driven hydrodehalogenation of substrate **1** to product **1-P**. (**a**) $^{1}H$ NMR spectrum of the reaction mixture containing substrate **1** (4-bromo-3-methyl-benzonitrile), [Cr($L^{Mes}$)$_3$], TDAE, 1,4-dioxane (internal standard, I. S.) in THF-$d_8$ (*). (**b**) $^{1}H$ NMR spectrum of the reaction mixture after 1 h of photoirradiation with a 623 nm LED. (**c**) $^{1}H$ NMR spectrum of the commercially available product **1-P** in THF-$d_8$ (*). The NMR yield of **1-P** from (**b**) is 94%.

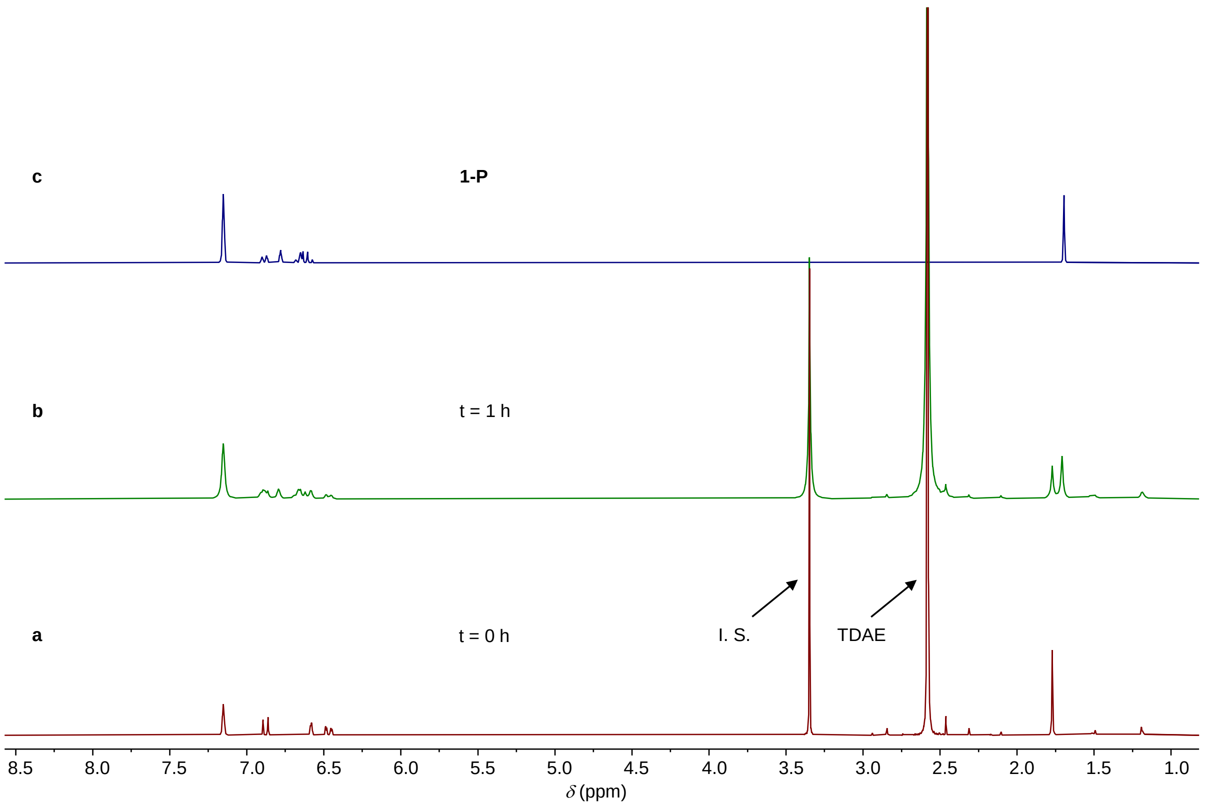


**Supplementary Fig. 116.** $^{1}$H NMR spectra monitoring the red-light driven hydrodehalogenation of substrate **1** to product **1-P**. (**a**) $^{1}$H NMR spectrum of the reaction mixture containing substrate **1** (4-bromo-3-methyl-benzonitrile), [Cr($L^{tBu}$)$_3$], TDAE, 1,4-dioxane (internal standard, I. S.) in $C_6D_6$. (**b**) $^{1}$H NMR spectrum of the reaction mixture after 1 h of photoirradiation with a 623 nm LED. (**c**) $^{1}$H NMR spectrum of the commercially available product **1-P** in $C_6D_6$. The NMR yield of **1-P** from (**b**) is 54%.

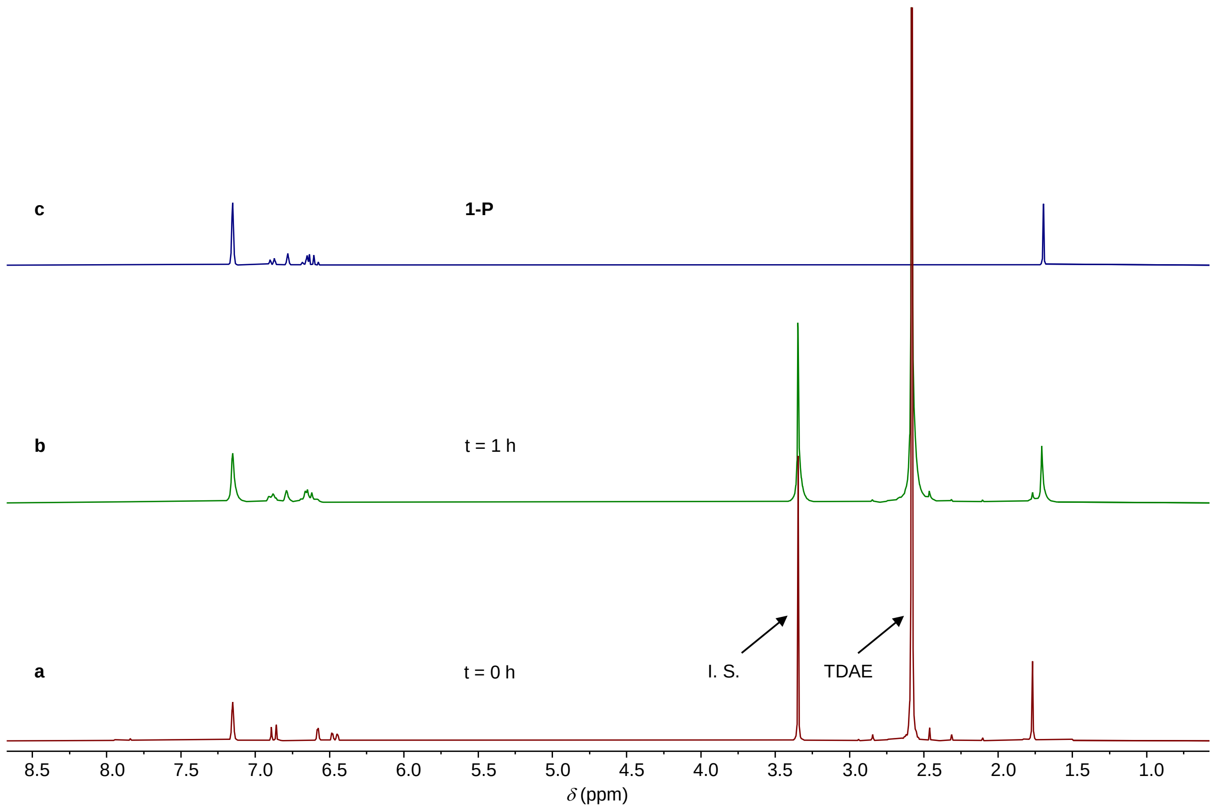


**Supplementary Fig. 117.** [1]H NMR spectra monitoring the red-light driven hydrodehalogenation of substrate **1** to product **1-P**. (**a**) [1]H NMR spectrum of the reaction mixture containing substrate **1** (4-bromo-3-methyl-benzonitrile), [Cr($L^{Pyr}$)$_3$], TDAE, 1,4-dioxane (internal standard, I. S.) in $C_6D_6$. (**b**) [1]H NMR spectrum of the reaction mixture after 1 h of photoirradiation with a 623 nm LED. (**c**) [1]H NMR spectrum of the commercially available product **1-P** in $C_6D_6$. The NMR yield of **1-P** from (**b**) is 83%.

## 6.6. Red-light induced base-promoted homolytic aromatic substitution (BHAS) reaction

A Schlenk flask was charged with chromium(0) photocatalyst ([Cr($L^{Mes}$)$_3$], 10 mol%). Then the flask was evacuated and back filled with argon (3 times). 1-(2-Iodobenzyl)-pyrrole[15] (0.03 mmol, 1.0 equiv), 2,2,6,6-tetramethylpiperidine (TMP, 2.0 equiv), and dry and deaerated $C_6D_6$ (0.6 mL) were added subsequently. The reaction mixture was deaerated through three cycles of freeze-pump-thaw, and anhydrous 1,4-dioxane (internal standard, 0.03 mmol) was added subsequently. Then the reaction mixture was transferred to an NMR tube under inert gas conditions and sealed. The NMR tube was irradiated with a 623 nm LED (Thorlabs Solis-623C, 3.8 W, with 550 nm long-pass filter). Yield was determined by NMR spectroscopy against the internal standard.

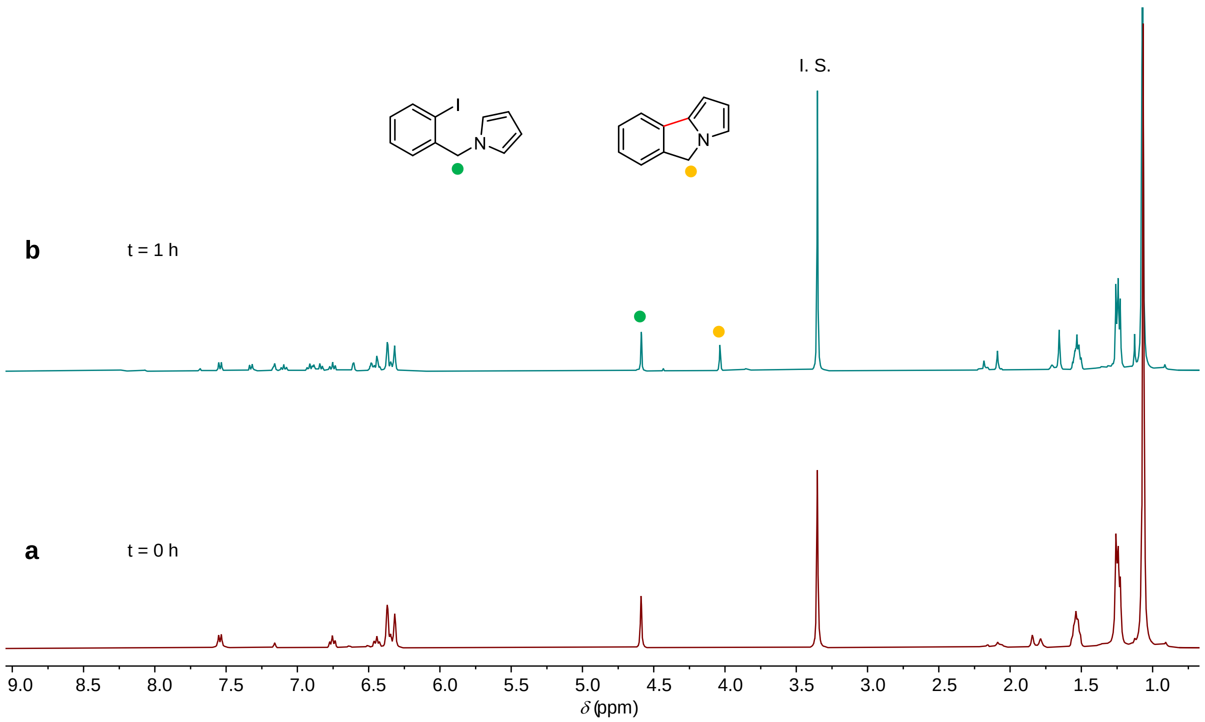


**Supplementary Fig. 118.** $^{1}H$ NMR spectra monitoring the red light driven BHAS reaction of 1-(2-iodobenzyl)-pyrrole. (**a**) $^{1}H$ NMR spectrum of the reaction mixture containing substrate 1-(2-iodobenzyl)-pyrrole (0.03 mmol, 1.0 equiv), [Cr($L^{Mes}$)$_3$] (10 mol%), TMP (2.0 equiv), 1,4-dioxane (internal standard, I. S.) in $C_6D_6$ (0.6 mL). (**b**) $^{1}H$ NMR spectrum of the reaction mixture after 1 h. The NMR yield of the C-C coupled product from (**b**) is 38%.

## 7. References


1. Suzuki, K. *et al.* Reevaluation of absolute luminescence quantum yields of standard solutions using a spectrometer with an integrating sphere and a back-thinned CCD detector. *Phys. Chem. Chem. Phys.* **11**, 9850-9860 (2009).
2. Lakowicz, J. R. Principles of fluorescence spectroscopy 3rd edition. *Springer, Springer Science+Business Media, LLC, 233 Spring Street, New York, NY 10013, USA* (2006).
3. Montalti, M., Credi, A., Prodi, L. & Gandolfi, M. T. Handbook of photochemistry 3rd edition. *CRC Press, Taylor & Francis Group, Boca Raton, FL 33487-2742* (2006).
4. Bilger, J. B., Kerzig, C., Larsen, C. B. & Wenger, O. S. A photorobust Mo(0) complex mimicking $[Os(2,2'\text{-bipyridine})_3]^{2+}$ and its application in red-to-blue upconversion. *J. Am. Chem. Soc.* **143**, 1651-1663 (2021).
5. Han, F. S., Higuchi, M. & Kurth, D. G. Diverse synthesis of novel bisterpyridines via Suzuki-type cross-coupling. *Org. Lett.* **9**, 559-562 (2007).
6. Büldt, L. A., Guo, X., Prescimone, A. & Wenger, O. S. A molybdenum(0) isocyanide analogue of $Ru(2,2'\text{-bipyridine})_3^{2+}$: a strong reductant for photoredox catalysis. *Angew. Chem. Int. Ed.* **55**, 11247-11250 (2016).
7. Büldt, L. A., Guo, X., Vogel, R., Prescimone, A. & Wenger, O. S. A tris(diisocyanide)chromium(0) complex is a luminescent analog of $Fe(2,2'\text{-bipyridine})_3^{2+}$. *J. Am. Chem. Soc.* **139**, 985-992 (2017).
8. Wegeberg, C., Häussinger, D. & Wenger, O. S. Pyrene-decoration of a chromium(0) tris(diisocyanide) enhances excited state delocalization: a strategy to improve the photoluminescence of $3d^6$ metal complexes. *J. Am. Chem. Soc.* **143**, 15800−15811 (2021).
9. Sheldrick, G. M. Crystal structure refinement with SHELXL. *Acta Cryst.* **C71**, 3-8 (2015).
10. Dolomanov, O. V., Bourhis, L. J., Gildea, R. J., Howard, J. A. K. & Puschmann, H. OLEX2: a complete structure solution, refinement and analysis program. *J. Appl. Cryst.* **42**, 339-341 (2009).
11. Sheldrick, G. M. SHELXT - Integrated space-group and crystal-structure determination. *Acta Cryst.* **A71**, 3-8 (2015).
12. Kjær, K. S. *et al.* Luminescence and reactivity of a charge-transfer excited iron complex with nanosecond lifetime. *Science* **363**, 249–253 (2019).
13. Connell, T. U. *et al.* The tandem photoredox catalysis mechanism of $[Ir(ppy)_2(dtb\text{-}bpy)]^+$ enabling access to energy demanding organic substrates. *J. Am. Chem. Soc.* **141**, 17646-17658 (2019).
14. Glaser, F. & Wenger, O. S. Red light-based dual photoredox strategy resembling the Z-scheme of natural photosynthesis. *JACS Au* **2**, 1488-1503 (2022).
15. Herr, P., Glaser, F., Büldt, L. A., Larsen, C. B. & Wenger, O. S. Long-Lived, Strongly Emissive, and Highly Reducing Excited States in Mo(0) Complexes with Chelating Isocyanides. *J. Am. Chem. Soc.* **141**, 14394-14402 (2019).
